# Application of the Hydrogen‐Bond‐Mediated Aglycone Delivery (HAD) Reaction to the Synthesis of *α*‐ and *β*‐Linked 2‐Azido‐2‐deoxyglucosides

**DOI:** 10.1002/asia.70928

**Published:** 2026-08-09

**Authors:** Alessandra Damico, Connor R. Mathus, Alexei V. Demchenko

**Affiliations:** ^1^ Department of Chemistry Saint Louis University St. Louis Missouri USA

**Keywords:** aminosugars, glycosyl donors, glycosylation, methodology, stereoselectivity

## Abstract

Controlling stereoselectivity of glycosylation reactions remains one of the central challenges in synthetic carbohydrate chemistry. In this study, we explore the impact of *O‐*picoloyl (Pico) protecting group positioning on the stereoselectivity of glycosylation with 2‐azido‐2‐deoxyglucosyl donors. Three glycosyl donors were designed and synthesized, each bearing the Pico group at a different position (C‐3, C‐4, or C‐6). The glycosyl donor performance was evaluated through H‐bond‐mediated aglycone delivery (HAD) glycosylation reaction with a series of primary and secondary glycosyl acceptors. All donors exhibited good to high yields across a range of substrates. However, the stereochemical outcome proved highly dependent on the position of the Pico group: 3‐Pico and 6‐Pico donors were *β*‐gluco selective, whereas 4‐Pico donors produced predominantly *α*‐linked products, albeit with moderate selectivity. The performance of the 6‐Pico derivative that produced glycosides with complete *β*‐gluco selectivity in all applications, was the most impressive. We then showcased how the 6‐Pico donor could be applied to the synthesis of *β*‐linked glycosaminoglycans up to a tetramer. Each glycosylation step proceeded with complete *β*‐selectivity, which opens a viable perspective to synthesizing this type of glycan sequence with glycosyl donors lacking a participating group at C‐2.

## Graphical abstract

H‐bond‐mediated aglycone delivery method has been applied to the synthesis of glucosamine glycosides.

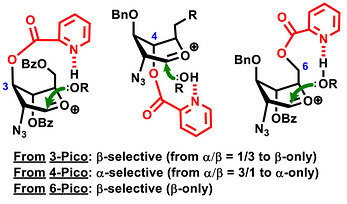

## 1 Introduction

Control over the stereoselectivity of glycosylation reactions remains a central and unresolved challenge in carbohydrate chemistry. Highly stereocontrolled formation of either *α*‐ or *β*‐glycosidic linkage is essential for accessing structurally defined oligosaccharides, glycoconjugates, or mimetics thereof to study the utility of these compounds as inhibitors, biomarkers, pharmaceuticals, and beyond [1, 2]. Yet, achieving high stereocontrol often depends on a subtle combination of factors including the substrate structure, protecting‐group pattern, and activation conditions [3, 4]. Among various strategies developed, the hydrogen‐bond‐mediated aglycone delivery (HAD) reaction pathway offers a powerful means for influencing glycosylation reactions [5, 6]. Note that the HAD reaction is not a typical neighboring or remote group participation that provides 1,X‐anti selectivity, but rather a conceptually different stereodirecting handle. Thus, our lab discovered that glycosyl donors equipped with 3‐, 4‐, or 6‐*O*‐picolinyl (Pic) or Pico protecting groups provide high *syn*‐selectivities in respect to Pic/Pico [7]. In the HAD reaction, the glycosyl acceptor (aglycone) forms an H‐bond with the Pic/Pico nitrogen of the glycosyl donor. Upon the leaving group activation and subsequent departure, the aglycone is delivered to form the glycosidic bond with high *syn*‐selectivity in respect to the remote Pic/Pico group [7].

Initially, conditions of choice were ethylthio glycosyl donor (5–50 mM) at −30°C in the presence of either DMTST [8, 9] or NIS/TfOH promoter system [10, 11]. The HAD reaction has been successfully applied to *α*‐glucosylation with 4‐Pico donor [7], *β*‐gluco with 6‐Pico donor [12], *β*‐manno with 3‐ and/or 6‐Pico donors [13], and these methods were applied to the synthesis of challenging *α*‐glucans [14] and *β*‐mannans [13, 15]. The HAD reaction was also applied to the synthesis of *β*‐glucosides with 3‐Pico‐protected glucosyl donors [12], a project that evolved into a thorough mechanistic study of the reaction pathway in the presence of excess triflic acid [16, 17]. 3‐Pico donors of the mannosamine (ManNAc) [18], mannuronic acid (ManA) [15], and ManNAcA [19] series have also been developed and applied to the synthesis of the repeating unit of *S. pneumoniae* capsular polysaccharide type 4 (CP4) [18], *β*‐(1→3)‐linked ManA tetrasaccharide [15], and the repeating unit of *Staphylococcus aureus* CP8 [19], all featuring *β*‐manno linkages [20]. We have also investigated altrosamine donors [21], and introduced a catalytic method for chemoselective removal of Pico with FeCl_3_ in the presence of all other common protecting groups [22]. These featured applications of the HAD reaction demonstrated how excellent stereocontrol can be achieved even in the absence of the neighboring group participation, establishing remote protecting groups as active stereodirecting handles rather than passive bystanders.

In application to the synthesis of aminosugars, 2‐azido‐2‐deoxy derivatives have become valuable glycosyl donors owing to their balance of reactivity, stability, and synthetic versatility. The azido group serves as a precursor to amino or acetamido functionalities (key motifs in many biologically active glycans) [23, 24, 25], while maintaining compatibility with diverse activation systems and many protecting group manipulations. The lack of this internal directing effect, while removing an inherent bias, also creates an opportunity to evaluate external stereodirecting mechanisms, such as those provided by Pico groups. Despite significant progress in the design of glycosyl donors capable of HAD, systematic evaluation of how the position of the Pico group affects the stereochemical preference of 2‐azido‐2‐deoxyglucosyl donors remains unexplored.

**FIGURE 1:**
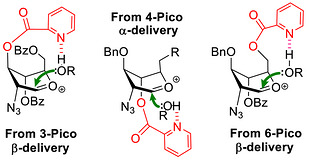
Hydrogen‐bond‐mediated aglycone delivery (HAD) with 2‐azido‐2‐deoxyglucosyl donors.

Given that H‐bonding geometry and donor conformation are inherently position‐dependent, a comparative analysis of differently substituted glycosyl donors can offer a critical insight into how remote interactions govern the outcome of the glycosylation reaction. To address this gap, reported herein is the design, synthesis, and investigation of three 2‐azido‐2‐deoxyglucosyl donors bearing the Pico substituent at the C‐3, C‐4, and C‐6 positions. The anticipated stereodirecting effect is shown in Figure 1. Thus, based on our previous observations 3‐Pico and 6‐Pico donors should be *β*‐selective because the Pico substituent in each of these compounds is projecting above the pyranose ring. In contrast, 4‐Pico donor should be *α*‐selective because the Pico substituent is projecting below the pyranose ring.

Each donor was then examined under universal reaction conditions with representative primary and secondary acceptors to assess the influence of Pico positioning on both the yield and stereoselectivity. The study reported herein provides a systematic framework to evaluate how subtle variations in donor architecture translate into measurable differences in stereoselectivity, offering a foundation for the rational design of new systems and platforms for stereocontrolled glycosylation. Ultimately, this study offers a new approach to the synthesis of glycans containing glucosamine residues, which are ubiquitous in nature albeit remain challenging targets for chemists [25].

## 2 Results and Discussion

**SCHEME 1:**
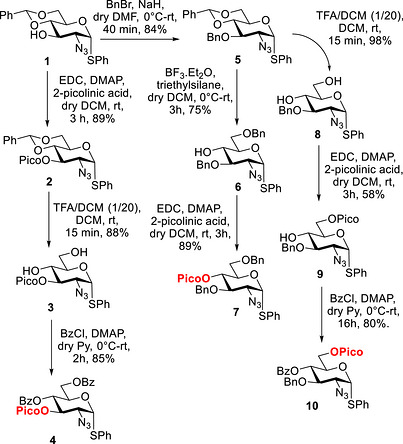
Synthesis of regioselectively protected glycosyl donors **4**, **7**, and **10**.

The synthesis of all glycosyl donors originated from D‐mannose, which was converted into the known phenylthiol glucosamine derivative **1** using well‐established synthetic manipulations [26]. Compound **1** then served as the universal precursor for the preparation of all three Pico‐substituted glycosyl donors of interest as depicted in Scheme 1. With the C‐3 position directly accessible, we reacted precursor **1** with picolinic acid in the presence of EDC and DMAP to afford 3‐Pico intermediate **2** in 89% yield. We note that our attempts to glycosidate compound **2** were largely unsuccessful, and its reaction under standard HAD reaction conditions at 5.0 mM concentration was extremely slow and gave only traces of the desired product even after 20 h. We believe that this is due to the deactivating effect of the benzylidene acetal that is known to disarm glycosyl donors via a combination of torsional and electron withdrawal effects [27, 28, 29]. Hence, benzylidene acetal in compound **2** was removed with TFA in DCM to afford diol **3** in 88% yield. Subsequent di‐*O*‐benzoylation of the latter with BzCl in pyridine afforded the desired 3‐Pico glycosyl donor **4** in 85% yield.

To obtain other glycosyl donors of these series, we benzylated compound number **1** with BnBr in the presence of NaH in DMF to afford 3‐*O*‐benzylated derivative **5** in 84% yield. From this point, to prepare 4‐Pico donor **7**, we carried out regioselective reductive benzylidene opening using boron trifluoride diethyl etherate and triethylsilane [30] to afford 4‐OH intermediate **6** in 75% yield. The latter was then picoloylated to afford the desired 4‐Pico donor **7** in 89% yield.

On the other hand, for the synthesis of 6‐Pico donor **10**, intermediate **5** was first subjected to hydrolytic benzylidene cleavage to afford diol **8** in 98% yield. The resulting compound was then subjected to regioselective mono‐picoloylation at the C‐6 position that furnished compound **9** in 58% yield. The remaining hydroxyl was then benzoylated to afford the desired 6‐Pico donor **10** in 80% yield.

**FIGURE 2:**
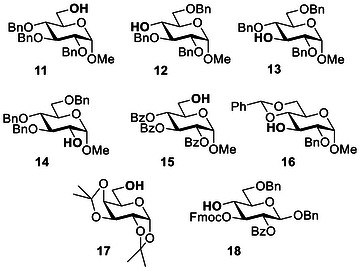
Glycosyl acceptors **11**–**18** used in this study.

With all three 2‐azido‐2‐deoxyglucosyl donors in hand, we began evaluating their performance in glycosylation in the presence of NIS/TfOH promoter system under activation conditions that were previously deemed standard for the HAD reactions with other substrates. In general, these reaction conditions are commonly employed for the activation of *S*‐phenyl donors [10]. Each donor was subjected to glycosidation with representative primary and secondary glycosyl acceptors **11–18** depicted in Figure 2 [31], and the key results of this study are summarized in Table 1.

**TABLE 1:** Glycosidation of donors **4**, **7**, and **10** with acceptors **11**–**18**.

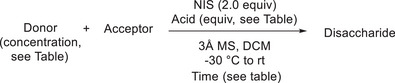				

Entries	Donors (concentration)	Acceptors	Acid additive, time	Product, yield, *α/β* ratio
1	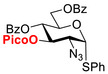 **4** (50 mM)	**11**	TfOH (0.5 equiv) 2 h	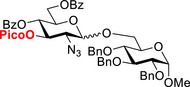 **19**: 60%, *α*/*β* = 1/3.0
2	**4** (50 mM)	**11**	TfOH (0.2 equiv) 5 h	**19**: 69%, *α*/*β* = 1/4.8
3	**4** (50 mM)	**11**	TMSOTf (0.2 equiv) 5 h	**19**: 69%, *α*/*β* = 1/3.0
4	**4** (50 mM)	**15**	TfOH (0.2 equiv) 8 h	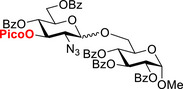 **20**: 48%, *α*/*β* = 1/3.0
5	**4** (50 mM)	**14**	TfOH (0.2 equiv) 16 h	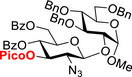 **21**: 30%, *β* only
6	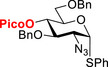 **7** (50 mM)	**11**	TfOH (0.2 equiv) 1 h	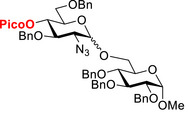 **22**: 96%, *α*/*β* = 6.0/1
7	**7** (25 mM)	**11**	TfOH (0.2 equiv) 5 h	**22**: 70%, *α*/*β* = 6.0/1
8	**7** (10 mM)	**11**	TfOH (0.2 equiv) 16 h	**22**: 59%, *α* only
9	**7** (50 mM)	**12**	TfOH (0.2 equiv) 24 h	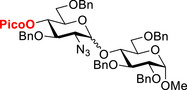 **23**: 52%, *α*/*β* = 3.8/1
10	**7** (50 mM)	**13**	TfOH (0.2 equiv) 16 h	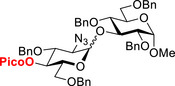 **24**: 81%, *α*/*β* = 3.0/1
11	**7** (50 mM)	**14**	TfOH (0.2 equiv) 5 h	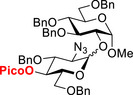 **25**: 77%, *α*/*β* = 2.7/1
12	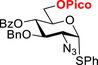 **10** (50 mM)	**11**	TfOH (0.2 equiv) 1 h	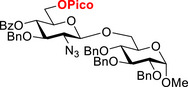 **26**: 90%, *β* only
13	**10** (50 mM)	**12**	TfOH (0.2 equiv) 24 h	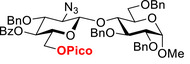 **27**: 38%, *β* only
14	**10** (50 mM)	**13**	TfOH (0.2 equiv) 16 h	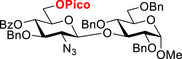 **28**: 45%, *β* only
15	**10** (50 mM)	**14**	TfOH (0.2 equiv) 16 h	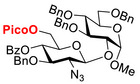 **29**: 54%, *β* only
16	**10** (50 mM)	**16**	TfOH (0.2 equiv) 16 h	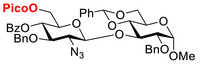 **30**: 66%, *β* only
17	**10** (50 mM)	**17**	TfOH (0.2 equiv) 16 h	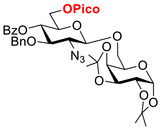 **31**: 82%, *β* only
18	**10** (50 mM)	**18**	TfOH (0.2 equiv) 24 h	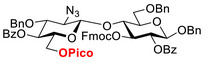 **32**: 24%, *β* only

The glycosylation behavior of donor **4**, bearing the Pico substituent at the C‐3 position, was first examined under standard reaction conditions (2.0 equiv of NIS, 0.5 equiv of TfOH, 50 mM concentration). Glycosylation of the primary tetra‐*O*‐benzylated acceptor **11** [31] proceeded smoothly to afford disaccharide **19** in 60% yield in 2 h with moderate *β*‐selectivity (*α*/*β* = 1/3.0, entry 1). Considering that excess acid is able to protonate the nitrogen of the Pico group, hence disrupting the HAD pathway [16], the amount of TfOH was reduced to 0.2 equiv. Under these milder reaction conditions, the reaction proceeded slower (5 h) and provided disaccharide **19** in 69% yield with improved stereoselectivity (*α*/*β* = 1/4.8, entry 2). This result is in line with our previous observations that partial protonation of the Pico group may influence HAD‐assisted stereoselectivity [16, 17]. Substitution of TfOH with TMSOTf afforded comparable reaction rate and yield albeit lower stereoselectivity, confirming that TfOH is the preferred promoter for this reaction (entry 3).

In contrast, when the reaction was conducted under high‐dilution conditions (5 mM), often beneficial for Pico‐directed reactions due to the reduced probability of the intermolecular nucleophilic attack [7], no product formation was observed even after 72 h. This outcome indicated that donor **4** requires higher effective concentration to achieve efficient activation. Therefore, further glycosylations were performed at 50 mM with 0.2 equiv of TfOH as the benchmark reaction conditions. Under these conditions, reaction with the less reactive *tetra*‐*O*‐benzoylated primary acceptor **15** [32] furnished disaccharide **20** in 48% yield after 8 h with identical stereoselectivity (*α*/*β* = 1/3.0, entry 4). Expectedly, no product was obtained at 5 mM concentration even after 72 h (result is not shown).

To further explore the reactivity profile of donor **4**, its performance with secondary 2‐OH glycosyl acceptor **14** [31] was also evaluated. This transformation proceeded sluggishly due to intrinsically low reactivity of secondary acceptors [33]. As a result, the corresponding disaccharide **21** was isolated in 30% yield in 16 h, albeit with complete *β*‐selectivity (entry 5). Both unreacted donor and acceptor have also been recovered, and the exclusive formation of the *β*‐anomer could be attributed to the known inverse correlation between reactivity and selectivity, whereat slower reactions often allow to achieve enhanced stereocontrol [34]. We also note that none of glycosylations with donor **4** proceeded to full conversion, which is evidenced by moderate yields of products in entries 1–5.

We then investigated glycosyl donor **7** bearing the Pico substituent at the C‐4 position under the same glycosylation reaction conditions as those used for donor **4**. According to the HAD pathway, relocation of the Pico group to C‐4 is expected to favor formation of the *α*‐anomer [14], as the directing group offers the bottom face delivery in this case (Figure 1). We note that donor **7** was also expected to be more reactive due to electron rich benzyl ether protecting groups [35]. Indeed, the reaction under the previously established reaction conditions in 50 mM concentration was swift and efficient. As a result, the corresponding disaccharide **22** was obtained in 96% yield in 1 h. The stereoselectivity was good, but not exceptional, and expectedly the *α*‐anomer predominated (*α*/*β* = 6.0/1, entry 6). Notably, the two anomers exhibited very different affinity to silica gel, which enabled a very straightforward separation of anomers, which were isolated and characterized individually. In attempt to enhance the stereoselectivity of this reaction, we investigated the effect of dilution. The reaction between donor **7** and acceptor **11** was repeated at 25, 10, and 5 mM concentration of the donor. At 25 mM, the reaction proceeded slowly, which negatively impacted the yield of disaccharide **22** that declined to 70% albeit no change in stereoselectivity was observed (*α*/*β* = 6.0/1, entry 7). At 10 mM concentration, the glycosylation was even slower (16 h), and disaccharide **22** was obtained in a moderate yield of 59%, albeit with complete *α*‐selectivity (entry 8). As in previous experiments, no disaccharide formation was detected even after 72 h when the reaction was performed at 5 mM concentration of the donor.

Following this optimization, the scope of the reaction with donor **7** was expanded to secondary acceptors **12–14** (Figure 2). These reactions provided the corresponding disaccharides **23‐**‐**25** in 52%–81% yields and modest stereoselectivities (*α*/*β* = 2.7–3.8/1, entries 9–11). Glycosylations with secondary acceptors or those conducted under high‐dilution conditions were all incomplete, and a significant amount of the acceptor was recovered. The *α*‐ and *β*‐anomers generated in these glycosylations were separable in all cases, enabling complete characterization of individual compounds.

We next investigated donor **10**, in which the Pico substituent occupies the primary position. As in previous cases, the initial glycosylation was performed with the primary acceptor **11** under the optimized NIS/TfOH‐promoted conditions, affording the corresponding disaccharide **26** in 90% yield after 1 h with exclusive formation of the *β*‐anomer (entry 12). Encouraged by this result, the study was extended to secondary acceptors **12–14** to further evaluate the scope of donor **10**. Remarkably, the reactions maintained complete *β*‐selectivity across all secondary acceptors tested. These glycosylations required longer reaction times (16–24 h), and the corresponding disaccharide **27–29** were isolated in 38%–54% yields (entries 13–15). The consistent complete stereoselectivity achieved with all glycosyl acceptors suggests that positioning the Pico group at the primary position is especially effective in facilitating the HAD pathway in application to glucosamine donors. Despite the favorable stereochemical outcome, none of the glycosylations with secondary acceptors proceeded to full conversion. A noticeable amount of unreacted acceptor was consistently recovered. In all cases, reactions were terminated once no further change in the TLC profile was observed over time. One inherent drawback of the HAD reaction is that it cannot typically be “pushed” by excess promoter without intervening with the HAD pathway and loosing stereoselectivity [7].

To further evaluate the compatibility of our reaction conditions with other protecting groups, such as acid‐labile acetal/ketal protecting groups or base‐labile Fmoc group, we investigated glycosyl acceptors **16**–**18** (Figure 2). First, 3‐OH glucosyl acceptor **16** [36] bearing a 4,6‐*O*‐benzylidene acetal was glycosylated under the optimized reaction conditions. The corresponding disaccharide **30** was obtained in 66% yield in 16 h, again with exclusive *β*‐selectivity (Table 1, entry 16). Similarly to several examples discussed above, the reaction did not reach full conversion and was terminated when no further progress was observed. The scope was further expanded to include diacetone galactose acceptor **17**. Gratifyingly, the desired disaccharide **31** was obtained in 82% yield in 16 h with complete *β*‐selectivity (Table 1, entry 17), further supporting the compatibility of the methodology with acceptors bearing acid‐labile protecting groups.

Finally, we investigated a much more hindered 4‐OH glycosyl acceptor **18** [37] carrying a base‐sensitive bulky Fmoc protecting group at C‐3. Although the reaction proceeded with complete *β*‐selectivity, only partial conversion was observed after 24 h, resulting in isolation of the desired disaccharide **32** in 24% yield (Table 1, entry 18). A substantial amount of acceptor was recovered, while the donor was only partially consumed and underwent competing decomposition pathways. The diminished reactivity can be attributed to the sterically demanding nature of the acceptor in combination with the intrinsically lower nucleophilicity of the secondary 4‐OH group [33]. In addition, purification proved particularly challenging, as product **32** and unreacted donor **10** displayed very similar chromatographic behavior in multiple solvent systems. Consequently, initial chromatography on silica gel afforded a mixture, requiring subsequent purification by size exclusion chromatography on Sephadex LH‐20 to obtain the pure product, which further contributed to the poor yield.

As previously observed, several reactions failed to reach full conversion, resulting in moderate to low isolated yields. This behavior likely reflects a combination of factors, including the moderate reactivity commonly associated with 2‐azido‐2‐deoxy glycosyl donors [38, 39] and the use of low promoter amounts, which were found to be essential for maintaining the HAD‐mediated stereocontrolling pathway [16]. Given the well‐established influence of protecting‐group pattern on glycosyl donor reactivity, subtle electronic and conformational effects may also contribute to the observed differences in reaction efficiency.

**SCHEME 2:**
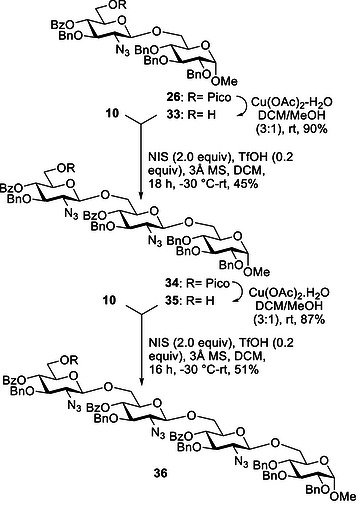
Application of 6‐Pico donor **10** in the synthesis of PNAG glycan fragments.

Having established the reactivity trends and stereochemical behavior of picoloylated 2‐azido‐2‐deoxyglucosyl donors, we next sought to demonstrate the synthetic utility of the optimized glycosylation methodology. Among all systems examined, the reaction between donor **10** (6‐Pico) and acceptor **11** that produced disaccharide **26** in 90% yield (entry 12, Table 1) was selected as the most suitable coupling for a proof‐of‐concept oligosaccharide synthesis, as it displayed the most favorable combination of stereoselectivity, yield, and reaction rate under the optimized conditions. To demonstrate the practical utility of this reaction, we chose to synthesize fragments of poly‐*N*‐acetylglucosamine (PNAG), a surface polysaccharide broadly expressed in both Gram‐positive and Gram‐negative bacteria, where it plays key roles in biofilm formation and cell adhesion [40]. In light of the biological importance of PNAG and its potential as a vaccine candidate or an immunotherapeutic target, we developed a synthetic approach to exemplify the applicability of the new methodology to the construction of structurally complex and biologically important glycans in the absence of the neighboring group participation. Thus, disaccharide **26** was selectively deprotected at the C‐6 position to remove the Pico substituent with Cu(OAc)_2_─H_2_O in DCM/MeOH while preserving the remaining protecting groups [22], thereby providing the corresponding disaccharide acceptor **33** in 90% yield (Scheme 2).

A subsequent glycosylation of acceptor **33** under the standard reaction conditions with donor **10** furnished trisaccharide **34** in 45% yield with complete *β*‐selectivity. Another iteration of selective deprotection of 6‐*O*‐Pico group produced trisaccharide acceptor **35** in 87% yield. Glycosylation of the latter with donor **10** produced the desired tetrasaccharide **36** in 51% yield, again with complete *β*‐selectivity. Owing to its involvement in bacterial virulence and immune evasion, PNAG represents an important pharmaceutical target, and a streamlined access to well‐defined synthetic glycan fragments is crucial for the development of vaccine candidates and immunological probes [40]. This application therefore highlights the practical relevance and robustness of the developed donor system for the stereoselective construction of complex *β*‐linked architectures.

## 3 Conclusions

In summary, this study has provided valuable insight into how the stereoselectivity of glycosylation reactions involving 2‐azido‐2‐deoxy sugars of the D‐gluco configuration can be varied through the strategic positioning of the Pico protecting group. This class of donors, highly relevant in carbohydrate chemistry due to their involvement in numerous biological processes and drug‐targeting applications, presents a significant stereochemical challenge, as the absence of neighboring group participation makes the control of selectivity particularly demanding. Within this context, the possibility of achieving controlled formation of *α*‐ and *β*‐glycosidic linkages on demand becomes especially attractive.

The results demonstrated that the position of the Pico group exerts a clear influence on the stereochemical outcome, directing the formation of the desired anomer with varying efficiency. In particular, substitution at C‐6 led to complete *β*‐selectivity, whereas the C‐3–substituted donor displayed moderate selectivity with the primary acceptors, but a notable increase with secondary acceptors, likely due to a slower and more controlled reaction process. In contrast, the C‐4–substituted donor showed only moderate selectivity with all acceptors; however, reaction concentration proved to be an additional determining factor, as dilution to 10 mM resulted in exclusive *α*‐selectivity, albeit with a significant decrease in yield. These findings are consistent with the general conventions of the HAD pathway, and further investigation of 4‐Pico substituted glucosamine donors may help to enhance *α*‐selectivity to achieve improved results on a par with other known approaches and strategies [41, 42, 43, 44, 45].

While we managed to achieve reproducible results and predictable reaction outcomes in terms of the stereoselectivity, some reaction yields were low. Unfortunately, the HAD reactions cannot be “pushed” by excess promoter without intervening with the HAD pathway. Having previously investigated NIS‐promoted reactions in the presence of larger amounts of triflic acid we observed relaxed stereoselectivity [7]. In some cases, such as with 3‐Pico protected glucosyl donors, we did obtain enhanced *β*‐selectivity, but low‐temperature NMR monitoring showed that these reactions proceed via a glycosyl triflate protonated at the Pico nitrogen rather than the HAD pathway [16, 17].

Based on these findings, optimized reaction conditions were established and subsequently applied to the stereoselective assembly of a PNAG tetrasaccharide fragment, demonstrating the practical utility of the developed methodology in the construction of biologically relevant *β*‐linked oligosaccharides. Overall, this study expands the understanding of remote protecting group effects in glycosylation chemistry and highlights the potential of picoloyl‐protected donors as versatile tools for the rational design of oligosaccharide targets.

## 4 Experimental Section

### 4.1 General Methods

The reactions were performed using commercial reagents. The ACS‐grade solvents used for reactions were purified and dried in accordance with standard procedures. Column chromatography was performed on silica gel 60 (70–230 mesh), reactions were monitored by TLC on Kieselgel 60 F254. The compounds were detected by examination under UV light and by charring with 5% sulfuric acid in methanol. Solvents were removed under reduced pressure at <40°C. CH_2_Cl_2_ and ClCH_2_CH_2_Cl (1,2‐DCE) were distilled from CaH_2_ directly prior to application. Molecular sieves (3Å), used for reactions, were crushed and activated in vacuo at 390°C for 8 h in the first instance and then for 2‐3 h at 390°C directly prior to application. Optical rotations were measured at “Jasco P‐2000” polarimeter. ^1^H NMR spectra were recorded in CDCl_3_ at 400 or 700 MHz, ^13^C NMR spectra were recorded at 101 or 176 MHz. The ^1^H NMR chemical shifts were referenced to tetramethylsilane (TMS, δ H = 0 ppm) or CDCl_3_ (δ H = 7.26 ppm) for ^1^H NMR spectra for solutions in CDCl_3_. The ^13^C NMR chemical shifts were referenced to the central signal of CDCl_3_ (δ C = 77.00 ppm) for solutions in CDCl_3_. Figures showing all spectra for all compounds are available in the Supporting Information. Mass analysis was performed using Agilent 6230 ESI TOF mass spectrometer.

### 4.2 Synthesis of Monosaccharide Building Blocks: Glycosyl Donors **4**, **7**, **10** and Glycosyl Acceptors **11**–**18**. For the Spectra of All New Compounds, Refer to Figures S1–S18

#### 4.2.1 Phenyl 2‐Azido‐4,6‐*O*‐benzylidene‐2‐deoxy‐3‐*O*‐picoloyl‐1‐thio‐*α*‐D‐glucopyranoside (**2**)

Picolinic acid (0.44 g, 3.56 mmol), EDC (0.68 g, 3.56 mmol), and DMAP (43 mg, 0.36 mmol) were added to a solution of compound **1** [26] (0.69 g, 1.78 mmol) in dry CH_2_Cl_2_ (18 mL), and the resulting mixture was stirred under argon for 3 h at rt. After that, the reaction mixture was diluted with CH_2_Cl_2_ (∼20 mL) and washed with water (10 mL), sat. aq. NaHCO_3_ (10 mL) and brine (10 mL). The organic phase was separated, dried with Na_2_SO_4_, and concentrated under reduced pressure. The residue was purified by column chromatography on silica gel (ethyl acetate‐hexane gradient elution) to afford the title compound as a white amorphous solid in 89% yield (0.78 g, 1.58 mmol). Analytical data for **2**: R*_f_* 0.20 (ethyl acetate/hexane, 3/7, *v*/*v*); [*α*]^23^_D_ +79.4 (*c* = 1.0, CHCl_3_); ^1^H NMR (400 MHz, CDCl_3_): *δ* 8.81–8.75 (m, 1H, aromatic), 8.24–8.17 (m, 1H, aromatic), 7.89–7.81 (m, 1H, aromatic), 7.56–7.45 (m, 3H, aromatic), 7.44–7.38 (m, 2H, aromatic), 7.37–7.27 (m, 6H, aromatic), 5.81 (dd, 1H, *J*_3,4_ = 9.9 Hz, H‐3), 5.71 (d, 1H, *J*_1,2_ = 5.6 Hz, H‐1), 5.52 (s, 1H, >C*H*Ph), 4.62–4.51 (m, 1H, *J*_5,6a_ = 4.9 Hz, *J*_5,6b_ = 9.9 Hz, H‐5), 4.34 (dd, 1H, *J*_2,3_ = 10.3 Hz, H‐2), 4.25 (dd, 1H, *J*_6a,6b_ = 10.5 Hz, H‐6a), 3.96 (dd, 1H, *J*_4,5_ = 9.09 Hz, H‐4), 3.79 (dd, 1H, H‐6b) ppm; ^13^C NMR (101 MHz, CDCl_3_): *δ* 164.0, 149.9, 147.2, 137.1, 136.7, 132.7, 132.6 (× 2), 129.2 (× 2), 129.1, 128.2 (× 2), 128.1, 127.3, 126.2 (× 2), 125.9, 101.7, 87.9, 79.3, 71.7, 68.5, 64.0, 62.3 ppm; ESI‐TOF [M + Na]^+^: calcd for [C_25_H_22_N_4_O_5_SNa] 513.1209; found: 513.1205.

#### 4.2.2 Phenyl 2‐Azido‐2‐deoxy‐3‐*O*‐picoloyl‐1‐thio‐*α*‐D‐glucopyranoside (**3**)

A solution of trifluoroacetic acid in CH_2_Cl_2_ (2.0 mL, 1/20, *v*/*v*) was added dropwise to a solution of compound **2** (0.78 g, 1.58 mmol) in wet CH_2_Cl_2_ (10 mL), and the resulting mixture was stirred for 15 min at rt. The reaction mixture was neutralized with triethylamine and concentrated under reduced pressure. The residue was purified by column chromatography on silica gel (methanol—dichloromethane gradient elution) to afford the title compound in 88% yield (0.56 g, 1.39 mmol). Analytical data for **3**: R*_f_* 0.50 (methanol/dichloromethane, 1/9, *v*/*v*); [*α*]^23^_D_ +72.7 (*c* = 1.0, CHCl_3_); ^1^H NMR (400 MHz, CDCl_3_): *δ* 8.68 (m, 1H, aromatic), 8.11 (m, 1H, aromatic), 7.85 (m, 1H, aromatic), 7.56–7.48 (m, 3H, aromatic), 7.38–7.29 (m, 3H, aromatic), 5.68 (d, 1H, *J*_1,2_ = 5.4 Hz, H‐1), 5.54 (dd, 1H, *J*_3,4_ = 10.5 Hz, H‐3), 4.38 (m, 1H, *J*_5,6a_ = 6.9 Hz, *J*_5,6b_ = 9.6 Hz, H‐5), 4.29 (d, 1H, 4‐OH), 4.25 (dd, 1H, *J*_2,3_ = 10.6 Hz, H‐2), 4.17 (dd, 1H, *J*_4,5_ = 10.5 Hz, H‐4), 3.97 (m, 1H, *J*_6a,6b_ = 11.5 Hz, H‐6a), 3.86 (m, 1H, H‐6b), 2.64 (t, 1H, 6‐OH) ppm; ^13^C NMR (101 MHz, CDCl_3_): *δ* 164.3, 149.1, 146.9, 137.6, 133.2, 132.4 (× 2), 129.2 (× 2), 127.9, 127.4, 125.7, 87.4, 76.4, 72.7, 68.2, 61.9, 61.0 ppm; ESI‐TOF [M + Na]^+^: calcd for [C_18_H_18_N_4_O_5_SNa] 425.0896; found: 425.0895.

#### 4.2.3 Phenyl 2‐Azido‐4,6‐di‐*O*‐benzoyl‐2‐deoxy‐3‐*O*‐picoloyl‐1‐thio‐*α*‐D‐glucopyranoside (**4**)

Benzoyl chloride (0.48 mL, 4.17 mmol) and DMAP (85 mg, 0.69 mmol) were added to a solution of compound **3** (0.56 g, 1.39 mmol) in pyridine (13 mL), and the resulting mixture was stirred under argon for 2 h at rt. After that, the reaction was quenched with MeOH (∼5 mL), the volatiles were removed under reduced pressure, and the residue was co‐evaporated with toluene. The resulting residue was diluted with CH_2_Cl_2_ (∼20 mL) and washed with 1 N aq. HCl (10 mL), sat. aq. NaHCO_3_ (10 mL) and brine (10 mL). The organic phase was separated, dried with Na_2_SO_4_, and concentrated under reduced pressure. The residue was purified by column chromatography on silica gel (ethyl acetate‐hexane gradient elution) to give the title compound as a colorless amorphous solid in 85% yield (0.72 g, 1.18 mmol). Analytical data for **4**: R*_f_* 0.50 (ethyl acetate/hexane, 1/1, *v*/*v*); [*α*]^23^_D_ +86.3 (*c* = 1.0, CHCl_3_); ^1^H NMR (400 MHz, CDCl_3_): *δ* 8.72 (m, 1H, aromatic), 8.03 (m, 1H, aromatic), 7.96 (m, 4H, aromatic), 7.74 (m, 1H, aromatic), 7.56–7.47 (m, 4H, aromatic), 7.42–7.33 (m, 5H, aromatic), 7.23–7.16 (m, 3H, aromatic), 5.92 (dd, 1H, *J*_3,4_ = 9.9 Hz, H‐3), 5.81 (d, 1H, *J*_1,2_ = 5.3 Hz, H‐1), 5.65 (dd, 1H, *J*_4,5_ = 9.3 Hz, H‐4), 4.99 (m, 1H, *J*_5,6a_ = 5.4 Hz, *J*_5,6b_ = 10.0 Hz, H‐5), 4.56–4.47 (m, 2H, *J*_6a,6b_ = 10.5 Hz, H‐6a, 6b), 4.45 (dd, 1H, *J*_2,3_ = 9.9 Hz, H‐2) ppm; ^13^C NMR (101 MHz, CDCl_3_): *δ* 166.1, 165.3, 163.9, 150.1, 146.7, 137.0, 133.5, 132.9, 132.4, 132.1 (× 2), 129.9 (× 2), 129.6 (× 2), 129.5, 129.1 (× 2), 128.6, 128.4 (× 2), 128.3 (× 2), 128.0, 127.2, 125.5, 86.8, 72.8, 69.8, 69.1, 63.0, 61.8 ppm; ESI‐TOF [M + Na]^+^: calcd for [C_32_H_26_N_4_O_7_SH] 611.1595; found: 611.1595.

#### 4.2.4 Phenyl 2‐Azido‐3,6‐di‐*O*‐benzyl‐2‐deoxy‐4‐*O*‐picoloyl‐1‐thio‐*α*‐D‐glucopyranoside (**7**)

Picolinic acid (0.29 g, 2.33 mmol), EDC (0.45 g, 2.33 mmol), and DMAP (28 mg, 0.23 mmol) were added to a solution of compound **6** [26] (0.56 g, 1.17 mmol) in dry CH_2_Cl_2_ (12 mL), and the resulting mixture was stirred under argon for 3 h at rt. After that, the reaction mixture was diluted with CH_2_Cl_2_ (∼20 mL) and washed with water (10 mL), sat. aq. NaHCO_3_ (10 mL), and brine (10 mL). The organic phase was separated, dried with Na_2_SO_4_, and concentrated under reduced pressure. The residue was purified by column chromatography on silica gel (ethyl acetate—hexane gradient elution) to afford the title compound as a white amorphous solid in 89% yield (0.61 g, 1.04 mmol). Analytical data for **7**: R*_f_* 0.60 (ethyl acetate/hexane, 1/1, *v*/*v*); [*α*]^23^_D_ +81.7 (*c* = 1.0, CHCl_3_); ^1^H NMR (400 MHz, CDCl_3_): *δ* 8.77 (m, 1H, aromatic), 8.06 (m, 1H, aromatic), 7.82 (m, 1H, aromatic), 7.57–7.46 (m, 3H, aromatic), 7.29–7.07 (m, 13H, aromatic), 5.64 (d, 1H, *J*_1,2_ = 3.7 Hz, H‐1), 5.55–5.47 (m, 1H, *J*_4,5_ = 10.3 Hz, H‐4), 4.84 (d, 1H, ^2^ *J* = 10.8 Hz, C*H*Ph), 4.77–4.71 (m, 1H, *J*_5,6a_ = 6.1 Hz, *J*_5,6b_ = 10.7 Hz, H‐5), 4.69 (d, 1H, ^2^ *J* = 10.8 Hz, C*H*Ph), 4.43 (m, 2H, *^2^J* = 11.7 Hz, 2 x C*H*Ph), 4.07 (m, 2H, H‐2, 3), 3.63 (m, 2H, *J*_6a,6b_ = 11.0 Hz, H‐6a, 6b) ppm; ^13^C NMR (101 MHz, CDCl_3_): *δ* 164.0, 149.9, 147.3, 137.6, 137.1, 137.0, 132.9, 132.5 (× 2), 129.1 (× 2), 128.2 (× 2), 128.1 (× 4), 127.9, 127.8, 127.7 (× 2), 127.4, 127.1, 125.7, 87.1, 79.3, 75.4, 73.5, 72.3, 70.2, 68.7, 63.8 ppm; ESI‐TOF [M + Na]^+^: calcd for [C_32_H_30_N_4_O_5_SNa] 605.1835; found: 605.1825.

#### 4.2.5 Phenyl 2‐Azido‐3‐*O*‐benzyl‐2‐deoxy‐6‐*O*‐picoloyl‐1‐thio‐*α*‐D‐glucopyranoside (**9**)

Picolinic acid (0.19 g, 1.52 mmol), EDC (0.29 g, 1.52 mmol), and DMAP (25 mg, 0.20 mmol) were added to a solution of compound **8** [26] (0.50 g, 1.01 mmol) in dry CH_2_Cl_2_ (10 mL), and the resulting mixture was stirred under argon for 3 h at rt. After that, the reaction mixture was diluted with CH_2_Cl_2_ (∼20 mL) and washed with water (10 mL), sat. aq. NaHCO_3_ (10 mL), and brine (10 mL). The organic phase was separated, dried with Na_2_SO_4_, and concentrated under reduced pressure. The residue was purified by column chromatography on silica gel (methanol—dichloromethane gradient elution) to afford the title compound as a white amorphous solid in 58% yield (0.29 g, 0.58 mmol). Analytical data for **2**: R*_f_* 0.50 (methanol/dichloromethane, 1/9, *v*/*v*); [*α*]^23^_D_ +73.1 (*c* = 1.0, CHCl_3_); ^1^H NMR (400 MHz, CDCl_3_): *δ* 8.75 (m, 1H, aromatic), 8.03 (m, 1H, aromatic), 7.81 (m, 1H, aromatic), 7.53–7.45 (m, 3H, aromatic), 7.44–7.29 (m, 5H, aromatic), 7.24–7.19 (m, 3H, aromatic), 5.60 (d, 1H, *J*_1,2_ = 5.4 Hz, H‐1), 4.97–4.88 (m, 2H, ^2^ *J* = 10.9 Hz, 2 x C*H*Ph), 4.86 (dd, 1H, *J*_5,6a_ = 4.7 Hz, *J*_6a,6b_ = 12.1 Hz, H‐6a), 4.53–4.46 (m, 2H, H‐5, H‐6b), 3.90 (dd, 1H, *J*_2,3_ = 9.9 Hz, H‐2), 3.73 (dd, 1H, *J*_3,4_ = 9.0 Hz, H‐3), 3.69–3.63 (m, 1H, H‐4), 3.24 (d, 1H, 4‐OH) ppm; ^13^C NMR (101 MHz, CDCl_3_): *δ* 165.3, 150.0, 147.3, 137.7, 137.0, 133.3, 131.9 (× 2), 129.1 (× 2), 128.7 (× 2), 128.3 (× 2), 128.1, 127.6, 127.1, 125.4, 86.9, 81.1, 75.7, 71.2, 71.0, 64.4, 63.5 ppm; ESI‐TOF [M + Na]^+^: calcd for [C_25_H_24_N_4_O_5_SNa] 515.1365; found: 515.1356.

#### 4.2.6 Phenyl 2‐Azido‐4‐*O*‐benzoyl‐3‐*O*‐benzyl‐2‐deoxy‐6‐*O*‐picoloyl‐1‐thio‐*α*‐D‐glucopyranoside (**10**)

Benzoyl chloride (0.10 mL, 0.87 mmol) and DMAP (35 mg, 0.29 mmol) were added to a solution of compound **9** (0.29 g, 0.58 mmol) in pyridine (5.0 mL), and the resulting mixture was stirred under argon for 16 h at rt. After that, the reaction was quenched with MeOH (∼3 mL), the volatiles were removed under reduced pressure, and the residue was co‐evaporated with toluene. The resulting residue was diluted with CH_2_Cl_2_ (∼20 mL) and washed with 1 N aq. HCl (10 mL), sat. aq. NaHCO_3_ (10 mL), and brine (10 mL). The organic phase was separated, dried with Na_2_SO_4_, and concentrated under reduced pressure. The residue was purified by column chromatography on silica gel (ethyl acetate‐hexane gradient elution) to give the title compound as a colorless amorphous solid in 80% yield (0.28 g, 0.46 mmol). Analytical data for **10**: R*_f_* 0.40 (ethyl acetate/hexane, 1/1, *v*/*v*); [*α*]^23^_D_ +74.8 (*c* = 1.0, CHCl_3_); ^1^H NMR (400 MHz, CDCl_3_): *δ* 8.58 (m, 1H, aromatic), 7.93 (m, 2H, aromatic), 7.87 (m, 1H, aromatic), 7.61 (m, 1H, aromatic), 7.45 (m, 1H, aromatic), 7.40 (m, 2H, aromatic), 7.36–7.26 (m, 3H, aromatic), 7.08–6.96 (m, 8H, aromatic), 5.53 (d, 1H, *J*_1,2_ = 5.3 Hz, H‐1), 5.35 (dd, 1H, *J*_4,5_ = 10.2 Hz, H‐4), 4.78 (m, 1H, *J*_5,6a_ = 6.3 Hz, *J*_5,6b_ = 11.6 Hz, H‐5), 4.70 (d, 1H, ^2^ *J* = 10.8 Hz, C*H*Ph), 4.54 (d, 1H, ^2^ *J* = 10.8 Hz, C*H*Ph), 4.49 (m, 1H, H‐6a), 4.38 (dd, 1H, *J*_6a,6b_ = 12.1 Hz, H‐6b), 3.98 (dd, 1H, *J*_2,3_ = 10.2 Hz, H‐2), 3.91 (dd, 1H, *J*_3,4_ = 10.2 Hz, H‐3) ppm; ^13^C NMR (101 MHz, CDCl_3_): *δ* 165.3, 164.5, 149.9, 147.5, 136.9, 136.8, 133.6, 132.6, 132.3 (× 2), 129.9 (× 2), 129.1 (× 3), 128.6 (× 2), 128.4 (× 2), 128.3 (× 2), 128.0, 127.8, 126.9, 125.4, 86.7, 79.2, 75.6, 71.5, 69.1, 63.9, 63.6 ppm; ESI‐TOF [M + Na]^+^: calcd for [C_32_H_28_N_4_O_6_SNa] 619.1627; found: 619.1618.

**Methyl 2,3,4‐tri‐*O*‐benzyl‐*α*‐D‐glucopyranoside (11)** was synthesized as reported previously, and its analytical data were in accordance with that previously described [31].

**Methyl 2,3,6‐tri‐*O*‐benzyl‐*α*‐D‐glucopyranoside (12)** was synthesized as reported previously, and its analytical data were in accordance with that previously described [31].

**Methyl 2,4,6‐tri‐*O*‐benzyl‐*α*‐D‐glucopyranoside (13)** was synthesized as reported previously, and its analytical data were in accordance with that previously described [31, 46].

**Methyl 3,4,6‐tri‐*O*‐benzyl‐*α*‐D‐glucopyranoside (14)** was synthesized as reported previously, and its analytical data were in accordance with that previously described [31].

**Methyl 2,3,4‐tri‐*O*‐benzoyl‐*α*‐D‐glucopyranoside (15)** was synthesized as reported previously, and its analytical data were in accordance with that previously described [32].

**Methyl 2‐*O*‐benzyl‐4,6‐*O*‐benzylidene‐*α*‐D‐glucopyranoside (16)** was synthesized as reported previously, and its analytical data were in accordance with that previously described [36].

**Benzyl 2‐*O*‐benzoyl‐6‐*O*‐benzyl‐3‐*O*‐fluorenylmethoxycarbonyl‐*β*‐D‐glucopyranoside (18)** was synthesized as reported previously and its analytical data were in accordance with that previously described [37].

### 4.3 Synthesis of Disaccharides **19**–**32**. For the Spectra of All New Compounds, Refer to Figures S19–S89

#### 4.3.1 A General Procedure for Glycosylation in the Presence of NIS/TfOH

A mixture of a glycosyl donor (0.034–0.035 mmol), glycosyl acceptor (0.023–0.024 mmol), and freshly activated molecular sieves (3 Å, 0.060 g) in dichloromethane (0.7 mL) was stirred under argon for 1 h at rt. The mixture was then cooled to −30°C, *N*‐iodosuccinimide (NIS, 0.068–0.070 mmol) and trifluoromethanesulfonic acid (TfOH, 0.6 µL, 0.0068–0.0070 mmol) were added. The external cooling was removed, resulting mixture was allowed to warm to ambient temperature, and stirred for the time listed in Table 1. After that, the solids were filtered off through a pad of celite and washed successively with CH_2_Cl_2_. The combined filtrate (∼40 mL) was washed with water (10 mL), 10% sodium thiosulfate (Na_2_S_2_O_3_, 10 mL), and water (2 × 10 mL). The organic phase was separated, dried with Na_2_SO_4_, and concentrated under reduced pressure. The residue was purified by column chromatography on silica gel (ethyl acetate—hexane gradient elution or acetone—toluene gradient elution) to afford respective disaccharide derivatives. Anomeric ratios (or anomeric purity) were determined by comparison of the integral intensities of the relevant signals in ^1^H NMR spectra. When both anomers were separable, the *α*/*β* ratio was determined by comparison of the weights of the individual anomers.

#### 4.3.2 Methyl 6‐*O*‐(2‐Azido‐4,6‐di‐*O*‐benzoyl‐2‐deoxy‐3‐*O*‐picoloyl‐D‐glucopyranosyl)‐2,3,4‐tri‐*O*‐benzyl‐*α*‐D‐glucopyranoside (**19**)

The title compound was obtained as a colorless amorphous solid from glycosyl donor **4** and acceptor **11** [31] in 60% yield (*α*/*β* = 1/3.0) or 69% yield (*α*/*β* = 1/4.8) or 69% (*α*/*β* = 1/3.0). Analytical data for **19**: R*_f_* 0.40 (acetone/toluene, 1/9, *v*/*v*); ^1^H NMR (400 MHz, CDCl_3_): *δ* 8.72 (m, 1H, aromatic), 8.07–7.92 (m, 3H, aromatic), 7.88 (m, 2H, aromatic), 7.82–7.72 (m, 1H, aromatic), 7.58–7.20 (m, 25H, aromatic), 5.65–5.50 (m, 2H, H‐3′, 4′), 5.03–4.89 (m, 2H, 2 x C*H*Ph), 4.86–4.73 (m, 2H, 2 x C*H*Ph), 4.69–4.59 (m, 3H, H‐1, 2 x C*H*Ph), 4.54 (dd, 1H, *J*_6a′,6b′_ = 12.2 Hz, H‐6a′), 4.47–4.37 (m, 2H, *J*_1′,2′_ = 8.5 Hz, H‐1′, 6b′), 4.13 (dd, 1H, *J*_6a,6b_ = 11.9 Hz, H‐6a), 4.01 (dd, 1H, *J*_3,4_ = 9.3 Hz, H‐3), 3.92 (m, 1H, *J*_5′,6a′_ = 4.7 Hz, *J*_5′,6b′_ = 10.2 Hz, H‐5′), 3.85 (m, 2H, H‐2′, 5), 3.76 (dd, 1H, *J*_6a,6b_ = 11.0 Hz, H‐6b), 3.56–3.49 (m, 2H, H‐2, 4), 3.38 (s, 3H, OCH_3_) ppm; ^13^C NMR (101 MHz, CDCl_3_): *δ* 166.0, 165.2, 164.0, 150.1, 146.7, 138.7, 138.3, 138.1, 137.0, 133.4, 133.1, 129.8 (× 2), 129.6 (× 2), 128.6, 128.5 (× 2), 128.4 (× 4), 128.3 (× 4), 128.1 (× 2), 127.9 (× 3), 127.8 (× 3), 127.6, 127.2, 125.5, 102.4, 98.1, 82.0, 79.8, 77.7, 77.2, 75.7, 74.8, 73.4 (× 2), 72.1, 69.7, 69.5, 68.9, 63.9, 62.9, 55.2 ppm; ESI‐TOF [M + Na]^+^: calcd for [C_54_H_52_N_4_O_13_Na] 987.3429; found: 987.3427.

#### 4.3.3 Methyl 6‐*O*‐(2‐Azido‐4,6‐di‐*O*‐benzoyl‐2‐deoxy‐3‐*O*‐picoloyl‐D‐glucopyranosyl)‐2,3,4‐tri‐*O*‐benzoyl‐*α*‐D‐glucopyranoside (**20**)

The title compound was obtained as a colorless amorphous solid from glycosyl donor **4** and acceptor **15** [32] in 48% yield (*α*/*β* = 1/3.0) under regular reaction conditions. Analytical data for *β* **‐20**: R*_f_* 0.50 (ethyl acetate/hexane, 1/1, *v*/*v*); ^1^H NMR (400 MHz, CDCl_3_): *δ* 8.73 (m, 1H, aromatic), 8.07–7.82 (m, 11H, aromatic), 7.77 (m, 1H, aromatic), 7.60–7.23 (m, 17H, aromatic), 6.16 (m, 1H, *J*_2,3_ = 9.6 Hz, H‐2, 3), 5.75–5.41 (m, 3H, H‐3′, 4, 4′), 5.31–5.21 (m, 1H, *J*_1,2_ = 3.6 Hz, H‐1), 4.74 (d, 1H, *J*_1′,2′_ = 7.9 Hz, H‐1′), 4.56 (m, 2H, H‐6a, 6a′), 4.47–4.26 (m, 2H, H‐5, 6b′), 4.14 (dd, 1H, *J*_6a,6b_ = 11.5 Hz, H‐6b), 4.02 (m, 1H, H‐5), 3.91–3.84 (m, 1H, H‐2′), 3.50 (s, 3H, OCH_3_) ppm; ^13^C NMR (101 MHz, CDCl_3_): *δ* 166.0, 165.7 (× 2), 165.4, 165.2, 163.9, 150.1, 146.8, 137.0, 133.4, 133.3, 133.1 (× 3), 133.0, 129.9, 129.8 (× 4), 129.7 (× 4), 129.6 (× 2), 129.5, 129.2, 129.1, 128.8, 128.6, 128.5, 128.4 (× 6), 128.3 (× 2), 128.2 (× 2), 127.2, 125.5, 102.7, 96.9, 73.2, 72.1, 72.0, 70.2, 69.7, 69.5, 68.9, 64.0, 55.7 ppm; ESI‐TOF [M + Na]^+^: calcd for [C_54_H_46_N_4_O_16_Na] 1029.2807; found: 1029.2812.

#### 4.3.4 Methyl 2‐*O*‐(2‐Azido‐4,6‐di‐*O*‐benzoyl‐2‐deoxy‐3‐*O*‐picoloyl‐*β*‐D‐glucopyranosyl)‐3,4,6‐tri‐*O*‐benzyl‐*α*‐D‐glucopyranoside (**21**)

The title compound was obtained as a colorless amorphous solid from glycosyl donor **4** and acceptor **14** [31] in 30% yield (*β* only) under regular reaction conditions. Analytical data for **21**: R*_f_* 0.60 (acetone/toluene, 1/9, *v*/*v*); [*α*]^23^_D_ +1.60 (*c* = 1.0, CHCl_3_); ^1^H NMR (700 MHz, CDCl_3_): *δ* 8.67 (m, 1H, aromatic), 8.09–8.04 (m, 1H, aromatic), 7.97–7.92 (m, 2H, aromatic), 7.91–7.87 (m, 2H, aromatic), 7.73 (m, 1H, aromatic), 7.52 (m, 2H, aromatic), 7.43–7.24 (m, 17H, aromatic), 7.20–7.15 (m, 2H, aromatic), 5.60 (dd, 1H, *J*_3′,4′_ = 9.8 Hz, H‐3′), 5.53 (dd, 1H, *J*_4′,5′_ = 9.7 Hz, H‐4′), 5.03 (d, 1H, ^2^ *J* = 10.5 Hz, C*H*Ph), 5.01 (d, 1H, *J*_1,2_ = 3.6 Hz, H‐1), 4.82 (m, 3H, *J*_1′,2′_ = 10.0 Hz, H‐1′, 2 x C*H*Ph), 4.64 (dd, 1H, *J*_6a′,6b′_ = 12.4 Hz, H‐6a′), 4.61 (d, 1H, C*H*Ph), 4.58–4.49 (m, 3H, H‐6b′, 2 x C*H*Ph), 4.07 (m, 1H, H‐5), 4.05 (dd, 1H, *J*_3,4_ = 10.2 Hz, H‐3), 3.94 (dd, 1H, *J*_2′,3′_ = 10.1 Hz, H‐2′), 3.82‐3.78 (m, 2H, *J*_2,3_ = 10.2 Hz, *J*_4,5_ = 10.0 Hz, H‐2, 4), 3.74 (dd, 1H, *J*_5,6a_ = 3.7 Hz, *J*_5,6b_ = 10.6 Hz, H‐6a), 3.72–3.65 (m, 2H, H‐5, 6b), 3.40 (s, 3H, OCH_3_) ppm; ^13^C NMR (176 MHz, CDCl_3_): *δ* 165.0, 164.9, 138.0, 137.7, 137.5, 133.0 (× 2), 129.4, 129.3 (× 2), 128.5, 128.3, 128.1, 128.0 (× 3), 127.9 (× 8), 127.7, 127.5 (× 3), 127.3 (× 3), 127.2 (× 5), 124.8, 102.3, 98.8, 80.6, 80.5, 77.7, 75.1, 74.5, 73.0, 72.8, 71.5, 69.4, 69.1, 68.1, 63.8, 63.1, 54.7, 29.2 ppm; ESI‐TOF [M + Na]^+^: calcd for [C_54_H_52_N_4_O_13_Na] 987.3429; found: 987.3428.

#### 4.3.5 Methyl 6‐*O*‐(2‐Azido‐3,6‐di‐*O*‐benzyl‐2‐deoxy‐4‐*O*‐picoloyl‐D‐glucopyranosyl)‐2,3,4‐tri‐*O*‐benzyl‐*α*‐D‐glucopyranoside (**22**)

The title compound was obtained as a colorless amorphous solid from glycosyl donor **7** and acceptor **11** [31] in 96% yield (*α*/*β* = 6.0/1, 50 mM) or 70% yield (*α*/*β* = 6.0/1, 25 mM) or 59% (*α* only, 10 mM) under regular and high dilution reaction conditions, respectively. Analytical data for *α*‐**22**: R*_f_* 0.60 (ethyl acetate/ hexane, 3.5/6.5, *v*/*v*); [*α*]^23^_D_ +48.9 (*c* = 1.0, CHCl_3_); ^1^H NMR (700 MHz, CDCl_3_): *δ* 8.77 (m, 1H, aromatic), 7.99 (m, 1H, aromatic), 7.80 (m, 1H, aromatic), 7.50 (m, 1H, aromatic), 7.43–7.28 (m, 17H, aromatic), 7.27–7.09 (m, 10H, aromatic), 5.49 (dd, 1H, *J*_4,5_ = 10.2 Hz, H‐4′), 5.14 (d, 1H, *J*_1,2_ = 3.6 Hz, H‐1′), 5.03 (d, 1H, ^2^ *J* = 11.0 Hz, C*H*Ph), 4.98 (d, 1H, ^2^ *J* = 11.0 Hz, C*H*Ph), 4.87–4.79 (m, 3H, 3 x C*H*Ph), 4.73–4.66 (m, 3H, 3 x C*H*Ph), 4.65 (d, 1H, *J*_1,2_ = 3.5 Hz, H‐1), 4.45 (dd, 2H, ^2^ *J* = 11.9 Hz, C*H_2_* Ph), 4.19 (dd, 1H, *J*_3′,4′_ = 10.0 Hz, H‐3′), 4.14 (m, 1H, *J*_5′,6a′_ = 4.6 Hz, *J*_5′,6b′_ = 10.2 Hz, H‐5′), 4.05 (dd, 1H, *J*_3,4_ = 9.4 Hz, H‐3), 3.89 (dd, 1H, *J*_6a,6b_ = 11.2 Hz, *J*_5,6a_ = 4.2 Hz, H‐6a), 3.84–3.77 (m, 2H, H‐5, 6b), 3.63 (dd, 1H*, J*_2,3_ = 9.8 Hz, H‐2), 3.61–3.49 (m, 4H, H‐2′, 4, 6a, 6b), 3.42 (s, 3H, OCH_3_) ppm; ^13^C NMR (176 MHz, CDCl_3_): *δ* 164.0, 149.8, 147.5, 138.7, 138.3, 138.1, 137.7, 137.4, 136.9, 128.4 (× 6), 128.2, 128.1 (× 5), 128.0 (× 2), 127.9, 127.8 (× 2), 127.7 (× 3), 127.6 (× 2), 127.4, 127.0, 125.5, 98.0, 97.9, 82.0, 80.1, 77.6, 75.8, 75.0, 74.6, 73.5, 73.4, 72.3, 70.2, 69.0, 68.6, 66.2, 63.2, 55.2, 30.0, 22.7, 14.4 ppm. Analytical data for *β*‐**22**: R*_f_* 0.25 (ethyl acetate/ hexane, 3.5/6.5, *v*/*v*); [*α*]^23^_D_ −9.4 (*c* = 1.0, CHCl_3_); ^1^H NMR (700 MHz, CDCl_3_): *δ* 8.75 (m, 1H, aromatic), 8.02 (m, 1H, aromatic), 7.81 (m, 1H, aromatic), 7.49 (m, 1H, aromatic), 7.38–7.29 (m, 12H, aromatic), 7.21–7.11 (m, 8H, aromatic), 5.34 (dd, 1H*, J*_4′,5′_ = 10.0 Hz, H‐4′), 5.00 (d, 1H, ^2^ *J* = 11.0 Hz, C*H*Ph), 4.96 (d, 1H, ^2^ *J* = 11.0 Hz, C*H*Ph), 4.86–4.79 (m, 3H, 3 x C*H*Ph), 4.70–4.65 (m, 3H, 3 x C*H*Ph), 4.64 (d, 1H*, J*_1,2_ = 3.6 Hz, H‐1), 4.46 (s, 2H, C*H*_2_Ph), 4.30 (d, 1H*, J*_1′,2′_ = 8.1 Hz, H‐1′), 4.15 (dd, 1H, *J*_6a,6b_ = 11.0 Hz, *J*_5,6a_ = 4.5 Hz, H‐6a), 4.03 (dd, 1H*, J*_3,4_ = 9.3 Hz, H‐3), 3.84 (m, 1H*, J*_5′,6a′_ = 4.5 Hz, *J*_5′,6b′_ = 10.9 Hz, H‐5′), 3.79 (dd, 1H, H‐6a′), 3.76 (dd, 1H, H‐6b′), 3.72 (dd, 1H*, J*_3′,4′_ = 10.0 Hz, H‐3′), 3.64‐3.56 (m, 5H, H‐2, 2′, 4, 5, 6b), 3.40 (s, 3H, OCH_3_) ppm; ^13^C NMR (176 MHz, CDCl_3_): *δ* 164.1, 149.8, 147.3, 138.8, 138.4, 138.1, 137.7, 137.4, 136.9, 128.5 (× 4), 128.4 (× 2), 128.2 (× 4), 128.1 (× 2), 128.0 (× 2), 127.9 (× 3), 127.8 (× 2), 127.7 (× 2), 127.6, 127.5 (× 2), 127.4, 127.1, 102.0, 98.2, 82.1, 80.4, 79.8, 77.7, 75.4, 75.1, 74.9, 73.5, 73.4 (× 2), 72.3, 69.6, 69.4, 68.5, 66.0, 55.2, 29.7 ppm; ESI‐TOF [M + Na]^+^: calcd for [C_54_H_56_N_4_O_11_Na] 959.3843; found: 959.3845.

#### 4.3.6 Methyl 4‐*O*‐(2‐Azido‐3,6‐di‐*O*‐benzyl‐2‐deoxy‐4‐*O*‐picoloyl‐D‐glucopyranosyl)‐2,3,6‐tri‐*O*‐benzyl‐*α*‐D‐glucopyranoside (**23**)

The title compound was obtained as a colorless amorphous solid from glycosyl donor **7** and acceptor **12** [31] in 52% yield (*α*/*β* = 3.8/1) under regular reaction conditions. Analytical data for *α*‐**23**: R*_f_* 0.40 (ethyl acetate/ hexane, 3.5/6.5, *v*/*v*); [*α*]^23^_D_ +51.1 (*c* = 1.0, CHCl_3_); ^1^H NMR (700 MHz, CDCl_3_): *δ* 8.77 (m, 1H, aromatic), 8.00 (m, 1H, aromatic), 7.81 (m, 1H, aromatic), 7.52–7.48 (m, 1H, aromatic), 7.39–7.25 (m, 13H, aromatic), 7.21 (m, 1H, aromatic), 7.18–7.11 (m, 8H, aromatic), 5.74 (d, 1H*, J*_1′,2′_ = 3.9 Hz, H‐1′), 5.48 (dd, 1H*, J*_4′,5′_ = 10.2 Hz, H‐4′), 5.13 (d, 1H, ^2^ *J* = 10.8 Hz, C*H*Ph), 4.91 (d, 1H, ^2^ *J* = 10.8 Hz, C*H*Ph), 4.78 (m, 2H, 2 x C*H*Ph), 4.66 (m, 2H, 2 x C*H*Ph), 4.63 (d, 1H, *J*_1,2_ = 3.4 Hz, H‐1), 4.56–4.51 (m, 2H, 2 x C*H*Ph), 4.38 (d, 1H, C*H*Ph), 4.26 (d, 1H, C*H*Ph), 4.20 (dd, 1H*, J*_3′,4′_ = 10.3 Hz, H‐3′), 4.13–4.08 (m, 2H, *J*_3,4_ = 10.2 Hz, H‐3, 5′), 3.94 (dd, 1H, *J*_4,5_ = 10.2 Hz, H‐4), 3.84–3.80 (m, 2H, H‐5, 6a), 3.73 (dd, 1H, *J*_6a,6b_ = 12.9 Hz, H‐6b), 3.60 (dd, 1H, *J*_2,3_ = 10.2 Hz, H‐2), 3.46–3.37 (m, 6H, H‐2′, 6a′, 6b′, OCH_3_) ppm; ^13^C NMR (176 MHz, CDCl_3_): *δ* 163.4, 149.3, 147.0, 138.2, 137.8, 137.4, 137.2, 136.9, 136.4, 128.0 (× 2), 127.9 (× 2), 127.7 (× 6), 127.6 (× 2), 127.5 (× 3), 127.2 (× 3), 126.9 (× 4), 126.8 (× 2), 126.5, 125.1, 97.5, 97.2, 81.2, 80.0, 77.1, 74.5, 74.4, 74.3, 73.0, 72.8 (× 2), 72.0, 69.1, 69.0 (× 2), 68.0, 62.5, 54.8, 29.2 ppm. Analytical data for *β*‐**23**: R*_f_* 0.20 (ethyl acetate/ hexane, 3.5/6.5, *v*/*v*); [*α*]^23^_D_ −9.03 (*c* = 1.0, CHCl_3_); ^1^H NMR (700 MHz, CDCl_3_): *δ* 8.73 (m, 1H, aromatic), 7.95 (m, 1H, aromatic), 7.76 (m, 1H, aromatic), 7.45 (m, 1H, aromatic), 7.37 (m, 4H, aromatic), 7.31–7.22 (m, 20H, aromatic), 7.18–7.07 (m, 8H, aromatic), 5.31 (dd, 1H*, J*_4′,5′_ = 9.1 Hz, H‐4′), 4.96 (d, 1H, ^2^ *J* = 11.0 Hz, C*H*Ph), 4.82–4.73 (m, 3H, 3 x C*H*Ph), 4.69–4.58 (m, 3H, 3 x C*H*Ph), 4.59 (d, 1H, *J*_1,2_ = 3.7 Hz, H‐1), 4.52 (d, 1H, ^2^ *J* = 12.1 Hz, C*H*Ph), 4.36–4.31 (m, 2H, *J*_1′,2′_ = 9.8 Hz, H‐1′, C*H*Ph), 4.18 (d, 1H, ^2^ *J* = 12.1 Hz, C*H*Ph), 3.98 (dd, 1H, *J*_3,4_ = 10.0 Hz, H‐3), 3.93 (dd, 1H, *J*_5,6a_ = 4.2 Hz, *J*_6a,6b_ = 11.0 Hz, H‐6a), 3.89 (dd, 1H, *J*_4,5_ = 10.0 Hz, H‐4), 3.79 (m, 1H, *J*_5,6b_ = 10.0 Hz, H‐5), 3.71 (dd, 1H, H‐6b), 3.57–3.41 (m, 5H, H‐2, 2′, 3′, 5′, 6a′), 3.40–3.34 (m, 4H, H‐6b′, OCH_3_) ppm; ^13^C NMR (176 MHz, CDCl_3_): *δ* 163.6, 149.3, 138.8, 137.8, 137.6, 137.4, 137.0, 136.3, 128.1 (× 2), 127.9 (× 2), 127.7 (× 2), 127.6 (× 4), 127.5 (× 6), 127.4, 127.3, 127.2 (× 2), 127.0 (× 2), 126.8, 126.7, 126.5, 124.9, 100.4, 97.9, 80.1, 79.6, 78.6, 75.0, 74.6, 73.1, 73.0 (× 2), 72.8, 72.3, 69.2 (× 2), 67.2, 66.1, 54.8, 29.2, 22.3, 13.6 ppm; ESI‐TOF [M + Na]^+^: calcd for [C_54_H_56_N_4_O_11_Na] 959.3843; found: 959.3842.

#### 4.3.7 Methyl 3‐*O*‐(2‐Azido‐3,6‐di‐*O*‐benzyl‐2‐deoxy‐4‐*O*‐picoloyl‐D‐glucopyranosyl)‐2,4,6‐tri‐*O*‐benzyl‐*α*‐D‐glucopyranoside (**24**)

The title compound was obtained as a colorless amorphous solid from glycosyl donor **7** and acceptor **13** [31] in 81% yield (*α*/*β* = 3.0/1) under regular reaction conditions. Analytical data for *α*‐**24**: R*_f_* 0.50 (ethyl acetate/ hexane, 3.5/6.5, *v*/*v*); [*α*]^23^_D_ +30.3 (*c* = 1.0, CHCl_3_); ^1^H NMR (700 MHz, CDCl_3_): *δ* 8.74 (m, 1H, aromatic), 7.85 (m, 1H, aromatic), 7.75 (m, 1H, aromatic), 7.46 (m, 1H, aromatic), 7.42–7.32 (m, 6H, aromatic), 7.30–7.23 (m, 7H, aromatic), 7.23–7.09 (m, 15H, aromatic), 5.60–5.54 (m, 2H, *J*_1′,2′_ = 3.0 Hz, *J*_4′,5′_ = 9.4 Hz, H‐1′, 4′), 4.92 (d, 1H, ^2^ *J* = 10.8 Hz, C*H*Ph), 4.80 (d, 1H, ^2^ *J* = 10.8 Hz, C*H*Ph), 4.73 (d, 1H, *J*_1,2_ = 3.6 Hz, H‐1), 4.71–4.64 (m, 3H, 3 x C*H*Ph), 4.60‐4.55 (m, 2H, H‐5′, C*H*Ph), 4.52 (m, 2H, 2 x C*H*Ph), 4.43 (d, 1H, ^2^ *J* = 11.4 Hz, C*H*Ph), 4.30 (d, 1H, ^2^ *J* = 11.6 Hz, C*H*Ph), 4.24 (dd, 1H, *J*_3′,4′_ = 9.3 Hz, H‐3′), 4.19 (dd, 1H, *J*_3,4_ = 9.1 Hz, H‐3), 3.82–3.72 (m, 3H, H‐4, 5, 6a), 3.67 (dd, 1H, *J*_6a,6b_ = 11.6 Hz, H‐6b), 3.55 (dd, 1H*, J*_2,3_ = 9.1 Hz, H‐2), 3.48 (dd, 1H, *J*_2′,3′_ = 9.3 Hz, H‐2′), 3.40 (dd, 1H, *J*_5′,6a′_ = 3.5 Hz, *J*_6a′,6b′_ = 11.2 Hz, H‐6a′), 3.33 (s, 3H, OCH_3_), 3.19 (dd, 1H, *J*_5,6b_ = 10.1 Hz, H‐6b′) ppm; ^13^C NMR (176 MHz, CDCl_3_): *δ* 163.3, 149.3, 147.3, 137.4, 137.2, 137.1, 137.0, 136.2, 128.2 (× 2), 128.0 (× 3), 127.9 (× 4), 127.7 (× 2), 127.6 (× 4), 127.5 (× 7), 127.4, 127.2, 126.9, 126.7, 126.3, 124.7, 97.3, 97.1, 78.4, 77.4 (× 2), 76.1, 74.1, 73.5, 73.2, 72.9, 72.1, 71.6, 69.2, 68.2, 67.7, 67.3, 62.5, 54.6 ppm. Analytical data for *β*‐**24**: R*_f_* 0.25 (ethyl acetate/ hexane, 3.5/6.5, *v*/*v*); [*α*]^23^_D_ −8.0 (*c* = 1.0, CHCl_3_); ^1^H NMR (700 MHz, CDCl_3_): *δ* 8.77 (m, 1H, aromatic), 8.00 (m, 1H, aromatic), 7.81 (m, 1H, aromatic), 7.52–7.48 (m, 1H, aromatic), 7.39–7.25 (m, 13H, aromatic), 7.21 (m, 1H, aromatic), 7.18–7.11 (m, 8H, aromatic), 5.74 (d, 1H*, J*_1′,2′_ = 3.9 Hz, H‐1′), 5.48 (dd, 1H*, J*_4′,5′_ = 10.2 Hz, H‐4′), 5.13 (d, 1H, ^2^ *J* = 10.8 Hz, C*H*Ph), 4.91 (d, 1H, ^2^ *J* = 10.8 Hz, C*H*Ph), 4.78 (m, 2H, 2 x C*H*Ph), 4.66 (dd, 2H, ^2^ *J* = 11.0 Hz, C*H*_2_Ph), 4.63 (d, 1H, *J*_1,2_ = 3.4 Hz, H‐1), 4.56–4.51 (m, 2H, 2 x C*H*Ph), 4.38 (d, 1H, C*H*Ph), 4.26 (d, 1H, C*H*Ph), 4.20 (dd, 1H*, J*_3′,4′_ = 10.3 Hz, H‐3′), 4.13–4.08 (m, 2H, *J*_3,4_ = 10.2 Hz, H‐3, 5′), 3.94 (dd, 1H, *J*_4,5_ = 10.2 Hz, H‐4), 3.84–3.80 (m, 2H, H‐5, 6a), 3.73 (dd, 1H, *J*_6a,6b_ = 12.9 Hz, H‐6b), 3.60 (dd, 1H, *J*_2,3_ = 10.2 Hz, H‐2), 3.46–3.37 (m, 6H, H‐2′, 6a′, 6b′, OCH_3_) ppm; ^13^C NMR (176 MHz, CDCl_3_): *δ* 163.4, 149.3, 147.0, 138.2, 137.8, 137.4, 137.2, 136.9, 136.4, 128.0, 127.9, 127.7 (× 5), 127.6, 127.5 (× 2), 127.2 (× 2), 126.9 (× 4), 126.8 (× 2), 126.5, 125.1, 97.5, 97.2, 81.2, 80.0, 77.1, 74.5, 74.4, 74.3, 73.0, 72.8 (× 2), 72.0, 69.1, 69.0 (× 2), 68.0, 62.5, 54.8, 29.2 ppm; ESI‐TOF [M + Na]^+^: calcd for [C_54_H_56_N_4_O_11_Na] 959.3843; found: 959.3843.

#### 4.3.8 Methyl 2‐*O*‐(2‐Azido‐3,6‐di‐*O*‐benzyl‐2‐deoxy‐4‐*O*‐picoloyl‐D‐glucopyranosyl)‐3,4,6‐tri‐*O*‐benzyl‐*α*‐D‐glucopyranoside (**25**)

The title compound was obtained as a colorless amorphous solid from glycosyl donor **7** and acceptor **14** [31] in 77% yield (*α*/*β* = 2.7/1) under regular reaction conditions. Analytical data for *α*‐**25**: R*_f_* 0.65 (ethyl acetate/ hexane, 3.5/6.5, *v*/*v*); [*α*]^23^_D_ +84.5 (*c* = 1.0, CHCl_3_); ^1^H NMR (400 MHz, CDCl_3_): *δ* 8.75 (m, 1H, aromatic), 7.68 (m, 1H, aromatic), 7.60 (m, 1H, aromatic), 7.45 (m, 1H, aromatic), 7.39–7.06 (m, 26H, aromatic), 5.55 (dd, 1H, *J*_4′,5′_ = 9.7 Hz, H‐4′), 5.11 (d, 1H*, J*_1′,2′_ = 3.6 Hz, H‐1′), 4.96 (d, 1H*, J*_1,2_ = 3.1 Hz, H‐1), 4.95 (d, 1H, ^2^ *J* = 11.5 Hz, C*H*Ph), 4.87 (d, 1H, ^2^ *J* = 11.5 Hz, C*H*Ph), 4.77 (dd, 2H, ^2^ *J* = 11.0 Hz, C*H*_2_Ph), 4.63 (dd, 2H, C*H*_2_Ph), 4.50–4.38 (m, 3H, 3 x C*H*Ph), 4.36–4.26 (m, 2H, H‐5′, C*H*Ph), 4.19 (dd, 1H*, J*_3′,4′_ = 9.7 Hz, H‐3′), 4.04 (dd, 1H, *J*_3,4_ = 9.3 Hz, H‐3), 3.88 (dd, 1H, *J*_2,3_ = 9.3 Hz, H‐2), 3.81 (m, 1H*, J*_5,6a_ = 3.7 Hz, *J*_5,6b_ = 10.0 Hz, H‐5), 3.75 (dd, 1H*, J*_6a,6b_ = 11.0 Hz, H‐6a), 3.67 (m, 2H, H‐4, 6b), 3.57 (dd, 1H, *J*_2′,3′_ = 9.7 Hz, H‐2′), 3.49–3.40 (m, 4H, H‐6a′, OCH_3_), 3.33 (dd, 1H, *J*_6a′,6b′_ = 11.1 Hz, H‐6b′) ppm; ^13^C NMR (101 MHz, CDCl_3_): *δ* 163.6, 149.9, 147.5, 138.5, 138.1, 137.9, 137.7, 137.3, 136.7, 128.4 (× 2), 128.3 (× 4), 128.2 (× 5), 128.1 (× 2), 127.9 (× 3), 127.8 (× 2), 127.7 (× 3), 127.6 (× 3), 127.4, 127.3, 126.8, 125.2, 96.5, 95.0, 80.7, 78.1, 77.5, 75.7, 74.9, 74.6, 73.5, 73.4, 71.8, 70.2, 69.1, 68.4, 68.0, 62.7, 55.3 ppm. Analytical data for *β*‐**25**: R*_f_* 0.30 (ethyl acetate/ hexane, 3.5/6.5, *v*/*v*); [*α*]^23^_D_ −2.8 (*c* = 1.0, CHCl_3_); ^1^H NMR (700 MHz, CDCl_3_): *δ* 8.74 (m, 1H, aromatic), 8.01 (m, 1H, aromatic), 7.80 (m, 1H, aromatic), 7.48 (m, 1H, aromatic), 7.40 (m, 2H, aromatic), 7.35–7.24 (m, 20H, aromatic), 7.19–7.10 (m, 11H, aromatic), 5.39 (dd, 1H*, J*_4′,5′_ = 9.6 Hz, H‐4′), 5.07 (d, 1H, ^2^ *J* = 10.7 Hz, C*H*Ph), 4.96 (d, 1H, *J*_1,2_ = 3.5 Hz, H‐1), 4.83–4.77 (m, 3H, 3 x C*H*Ph), 4.64 (dd, 2H, ^2^ *J* = 11.6 Hz, C*H*_2_Ph), 4.54 (d, 1H*, J*_1′,2′_ = 8.2 Hz, H‐1′), 4.50 (dd, 2H, ^2^ *J* = 11.4 Hz, C*H*_2_Ph), 4.46–4.39 (m, 2H, 2 x C*H*_2_Ph), 4.03 (dd, 1H, *J*_3,4_ = 9.4 Hz, H‐3), 3.82‐3.72 (m, 5H, H‐2, 3′, 5, 5′, 6a), 3.70–3.65 (m, 3H, H‐2′, 4, 6b), 3.61–3.56 (m, 2H, H‐6a′, 6b′), 3.39 (s, 3H, OCH_3_) ppm; ^13^C NMR (101 MHz, CDCl_3_): *δ* 163.6, 149.3, 146.9, 138.3, 137.7, 137.5, 137.1, 136.8, 136.4, 127.9 (× 5), 127.7 (× 6), 127.4 (× 4), 127.2 (× 5), 127.1, 127.0 (× 2), 126.6, 125.1, 102.2, 99.0, 80.6 (× 2), 80.2, 77.6, 75.0, 74.6, 74.5, 73.0 (× 2), 72.6, 71.8, 69.4, 68.8, 68.0, 65.3, 54.7, 29.2 (× 2) ppm; ESI‐TOF [M + Na]^+^: calcd for [C_54_H_56_N_4_O_11_Na] 959.3843; found: 959.3845.

#### 4.3.9 Methyl 6‐*O*‐(2‐Azido‐4‐*O*‐benzoyl‐3‐*O*‐benzyl‐2‐deoxy‐6‐*O*‐picoloyl‐*β‐*D‐glucopyranosyl)‐2,3,4‐tri‐*O*‐benzyl‐*α*‐D‐glucopyranoside (**26**)

The title compound was obtained as a colorless amorphous solid from glycosyl donor **10** and acceptor **11** [31] in 90% yield (*β* only) under regular reaction conditions. Analytical data for **26**: R*_f_* 0.35 (ethyl acetate/hexane, 3.5/6.5, *v*/*v*); [*α*]^23^_D_ −11.5 (*c* = 1.0, CHCl_3_); ^1^H NMR (700 MHz, CDCl_3_): *δ* 8.66 (m, 1H, aromatic), 8.02 (m, 1H, aromatic), 7.98–7.93 (m, 2H, aromatic), 7.72 (m, 1H, aromatic), 7.57 (m, 1H, aromatic), 7.46–7.39 (m, 3H, aromatic), 7.39–7.27 (m, 12H, aromatic), 7.19–7.12 (m, 4H, aromatic), 5.40 (dd, 1H, *J*_4′,5′_ = 10.0, H‐4′), 5.00 (d, 1H, *^2^J* = 10.9 Hz, C*H*Ph), 4.94 (d, 1H, ^2^ *J* = 10.9 Hz, C*H*Ph), 4.85–4.75 (m, 3H, 3 x C*H*Ph), 4.69–4.61 (m, 4H, H‐1, 3 x C*H*Ph), 4.56–4.49 (m, 2H, H‐6a′, 6b′), 4.31 (d, 1H*, J*_1′,2′_ = 7.7 Hz, H‐1′), 4.12 (dd, 1H, *J*_6a,6b_ = 10.9 Hz, H‐6a), 4.00 (dd, 1H, *J*_3,4_ = 9.3 Hz, H‐3), 3.88 (m, 1H, *J*_5′,6a′_ = 5.2 Hz, *J*_5′,6b′_ = 10.0 Hz, H‐5′), 3.80 (m, 1H, *J*_5,6a_ = 4.3 Hz, *J*_5,6b_ = 10.1 Hz, H‐5), 3.74 (dd, 1H, H‐6b), 3.67–3.54 (m, 4H, H‐2, 2′, 3′, 4), 3.39 (s, 3H, OCH_3_) ppm; ^13^C NMR (176 MHz, CDCl_3_): 164.6, 164.0, 149.4, 146.9, 138.3, 137.9, 137.6, 136.5, 136.4, 133.0, 129.3 (× 2), 128.7, 128.0 (× 5), 127.9 (× 2), 127.8 (× 2), 127.7 (× 7), 127.5 (× 2), 127.4, 127.3 (× 2), 127.2, 127.1, 126.4, 124.8, 101.6, 97.7, 81.6, 79.7, 79.3, 77.1, 75.2, 74.7, 74.4, 73.0, 71.3, 70.5, 69.1, 68.0, 65.5, 63.6, 54.8, 29.2, 29.1 ppm; ESI‐TOF [M + Na]^+^: calcd for [C_54_H_54_N_4_O_12_Na] 973.3636; found: 973.3630.

#### 4.3.10 Methyl 4‐*O*‐(2‐Azido‐4‐*O*‐benzoyl‐3‐*O*‐benzyl‐2‐deoxy‐6‐*O*‐picoloyl‐*β‐*D‐glucopyranosyl)‐2,3,6‐tri‐*O*‐benzyl‐*α*‐D‐glucopyranoside (**27**)

The title compound was obtained as a colorless amorphous solid from glycosyl donor **10** and acceptor **12** [31] in 38% yield (*β* only) under regular reaction conditions. Analytical data for **27**: R*_f_* 0.45 (acetone/toluene, 1/9, *v*/*v*); [*α*]^23^_D_ −8.2 (*c* = 1.0, CHCl_3_); ^1^H NMR (700 MHz, CDCl_3_): *δ* 8.65 (m, 1H, aromatic), 7.93 (m, 3H, aromatic), 7.63–7.55 (m, 2H, aromatic), 7.46–7.23 (m, 14H, aromatic), 7.21–7.11 (m, 6H, aromatic), 5.33 (dd, 1H, *J*_4′,5′_ = 10.0 Hz, H‐4′), 4.98 (d, 1H, ^2^ *J* = 11.1 Hz, C*H*Ph), 4.85–4.74 (m, 3H, 3 x C*H*Ph), 4.71 (d, 1H, ^2^ *J* = 11.1 Hz, C*H*Ph), 4.66–4.60 (m, 2H, *J*_1,2_ = 3.0 Hz, H‐1, C*H*Ph), 4.58 (d, 1H, ^2^ *J* = 11.1 Hz, C*H*Ph), 4.45 (d, 1H, ^2^ *J* = 11.0 Hz, C*H*Ph), 4.35 (dd, 1H*, J*_5′,6a′_ = 3.6 Hz, *J*_6a′,6b′_ = 12.0 Hz, H‐6a′), 4.26 (m, 2H, *J*_1′,2′_ = 8.0 Hz, H‐1′, 6b), 4.00 (dd, 1H, *J*_5,6a_ = 4.6 Hz, *J*_6a,6b_ = 12.6 Hz, H‐6a), 3.98 (dd, 1H, *J*_4,5_ = 9.0 Hz, H‐4), 3.90 (dd, 1H*, J*_3,4_ = 9.0 Hz, H‐3), 3.80 (m, 1H, H‐5), 3.74 (dd, 1H, H‐6b), 3.51–3.46 (m, 2H, H‐2, 5′), 3.44 (dd, 1H, *J*_2′,3′_ = 9.8 Hz, H‐2′), 3.41 (s, 3H, OCH_3_), 3.30 (dd, 1H*, J*_3′,4′_ = 9.8 Hz, H‐3′) ppm; ^13^C NMR (176 MHz, CDCl_3_): *δ* 164.6, 163.7, 149.4, 146.9, 138.6, 137.7, 137.5, 136.6, 136.3, 132.9, 129.3 (× 2), 128.8, 128.2 (× 2), 128.0 (× 4), 127.9 (× 2), 127.8 (× 2), 127.6 (× 2), 127.5 (× 5), 127.4, 127.3, 127.1 (× 2), 126.7, 126.2, 124.8, 100.4, 97.9, 79.8, 79.4, 78.4, 74.8, 74.5, 72.9 (× 2), 70.8, 70.6, 69.1, 67.6, 65.9, 63.4, 54.9, 29.2 ppm; ESI‐TOF [M + Na]^+^: calcd for [C_54_H_54_N_4_O_12_Na] 973.3636; found: 973.3635.

#### 4.3.11 Methyl 3‐*O*‐(2‐Azido‐4‐*O*‐benzoyl‐3‐*O*‐benzyl‐2‐deoxy‐6‐*O*‐picoloyl‐*β‐*D‐glucopyranosyl)‐2,4,6‐tri‐*O*‐benzyl‐*α*‐D‐glucopyranoside (**28**)

The title compound was obtained as a colorless amorphous solid from glycosyl donor **10** and acceptor **13** [31] in 45% yield (*α*/*β* = 1/25.0) under regular reaction conditions. Analytical data for **28**: R*_f_* 0.30 (acetone/toluene, 1/9, *v*/*v*); [*α*]^23^_D_ −6.6 (*c* = 1.0, CHCl_3_); ^1^H NMR (700 MHz, CDCl_3_): *δ* 8.61 (m, 1H, aromatic), 7.94–7.89 (m, 3H, aromatic), 7.59 (m, 1H, aromatic), 7.54 (m, 1H, aromatic), 7.49–7.45 (m, 2H, aromatic), 7.40–7.30 (m, 12H, aromatic), 7.18–7.08 (m, 11H, aromatic), 5.42 (dd, 1H, *J*_4′,5′_ = 9.4 Hz, H‐4′), 5.06 (d, 1H, *J*_1′,2′_ = 8.0 Hz, H‐1′), 4.96 (d, 1H, ^2^ *J* = 11.0 Hz, C*H*Ph), 4.87 (d, 1H, ^2^ *J* = 11.7 Hz, C*H*Ph), 4.78 (d, 1H, ^2^ *J* = 11.0 Hz, C*H*Ph), 4.61 (dd, 2H, ^2^ *J* = 11.7 Hz, C*H*_2_Ph), 4.57–4.54 (m, 2H, *J*_1,2_ = 3.4 Hz, H‐1, C*H*Ph), 4.47 (d, 1H, ^2^ *J* = 11.7 Hz, C*H*Ph), 4.44–4.39 (m, 3H, H‐6a′, 6b′, C*H*Ph), 4.31 (dd, 1H*, J*_3,4_ = 9.7 Hz, H‐3), 3.85 (m, 1H, *J*_5′,6a′_ = 4.8 Hz, *J*_5′,6b′_ = 10.0 Hz, H‐5′), 3.70–3.64 (m, 3H, *J*_2,3_ = 9.7 Hz, H‐2, 6a, 6b), 3.62–3.58 (m, 3H, H‐2′, 4, 5), 3.55 (dd, 1H, *J*_3′,4′_ = 9.4 Hz, H‐3′), 3.28 (s, 3H, OCH_3_) ppm; ^13^C NMR (176 MHz, CDCl_3_): *δ* 164.7, 163.8, 149.3, 146.9, 137.8, 137.5, 137.4, 136.7, 136.1, 132.8, 129.3 (× 2), 128.1 (× 4), 127.9 (× 4), 127.8 (× 2), 127.7 (× 3), 127.6 (× 2), 127.5 (× 2), 127.4 (× 2), 127.3, 127.2, 127.0, 126.1, 124.8, 101.5, 96.9, 80.4, 79.6, 78.9, 75.1, 74.7, 74.2, 73.0, 72.9, 70.8, 70.6, 69.2, 68.0, 66.0, 63.6, 54.6, 29.1 ppm; ESI‐TOF [M + Na]^+^: calcd for [C_54_H_54_N_4_O_12_Na] 973.3636; found: 973.3635.

#### 4.3.12 Methyl 2‐*O*‐(2‐Azido‐4‐*O*‐benzoyl‐3‐*O*‐benzyl‐2‐deoxy‐6‐*O*‐picoloyl‐*β‐*D‐glucopyranosyl)‐3,4,6‐tri‐*O*‐benzyl‐*α*‐D‐glucopyranoside (**29**)

The title compound was obtained as a colorless amorphous solid from glycosyl donor **10** and acceptor **14** [31] in 54% yield (*β* only) under regular reaction conditions. Analytical data for **29**: R*_f_* 0.50 (ethyl acetate/hexane, 3.5/6.5, *v*/*v*); [*α*]^23^_D_ −4.5 (*c* = 1.0, CHCl_3_); ^1^H NMR (400 MHz, CDCl_3_): *δ* 8.64 (m, 1H, aromatic), 8.04 (m, 1H, aromatic), 7.97 (m, 2H, aromatic), 7.70 (m, 1H, aromatic), 7.57 (m, 1H, aromatic), 7.47–7.07 (m, 25H, aromatic), 5.42 (dd, 1H, *J*_4′,5′_ = 10.4 Hz, H‐4′), 5.06 (d, 1H, ^2^ *J* = 10.7 Hz, C*H*Ph), 4.96 (d, 1H*, J*_1,2_ = 3.5 Hz, H‐1), 4.80 (dd, 3H, ^2^ *J* = 10.7 Hz, 3 x C*H*Ph), 4.60 (m, 4H*, J*_1′,2′_ = 10.0 Hz, *J*_6a′,6b′_ = 11.2 Hz, H‐1′, 6a′, C*H*_2_Ph), 4.54–4.40 (m, 3H, H‐6b′, C*H*_2_Ph), 4.02 (dd, 1H*, J*_3,4_ = 9.3 Hz, H‐3), 3.88 (m, 1H, *J*_5′,6a′_ = 5.8 Hz, *J*_5′,6b′_ = 9.4 Hz, H‐5′), 3.82–3.60 (m, 7H, H‐2, 2′, 3′, 4, 5, 6a, 6b), 3.37 (s, 3H, OCH_3_) ppm; ^13^C NMR (101 MHz, CDCl_3_): *δ* 165.1, 164.4, 149.9, 138.7, 138.1, 138.0, 136.8, 133.5, 129.8, 129.0, 128.5, 128.4 (× 3), 128.3 (× 7), 127.9 (× 2), 127.8 (× 4), 127.7 (× 2), 127.6 (× 2), 127.5, 125.3, 102.8, 99.3, 81.3, 81.0, 80.9, 78.1, 75.5, 75.3, 75.0, 73.4, 72.0, 70.9, 69.8, 68.6, 65.7, 63.7, 55.2, 31.9, 29.7, 22.7, 14.1 ppm; ESI‐TOF [M + Na]^+^: calcd for [C_54_H_54_N_4_O_12_Na] 973.3636; found: 973.3633.

#### 4.3.13 Methyl 3‐*O*‐(2‐Azido‐4‐*O*‐benzoyl‐3‐*O*‐benzyl‐2‐deoxy‐6‐*O*‐picoloyl‐*β‐*D‐glucopyranosyl)‐2‐*O*‐benzyl‐4,6‐*O*‐benzylidene‐*α*‐D‐glucopyranoside (**30**)

The title compound was obtained as a colorless amorphous solid from glycosyl donor **10** and acceptor **16** [36] in 66% yield (*β* only) under regular reaction conditions. Analytical data for **30**: R*_f_* 0.40 (acetone/toluene, 1/9, *v*/*v*); [*α*]^23^_D_ −32.3 (*c* = 1.0, CHCl_3_); ^1^H NMR (400 MHz, CDCl_3_): *δ* 8.56 (m, 1H, aromatic), 7.90–7.81 (m, 3H, aromatic), 7.57–7.45 (m, 2H, aromatic), 7.41–7.26 (m, 11H, aromatic), 7.17–7.05 (m, 8H, aromatic), 5.51 (s, 1H, >C*H*Ph), 5.33 (dd, 1H, *J*_4′,5′_ = 10.0 Hz, H‐4′), 4.86 (d, 1H, ^2^ *J* = 11.8 Hz, C*H*Ph), 4.77 (d, 1H, *J*_1′,2′_ = 8.1 Hz, H‐1′), 4.69 (d, 1H, ^2^ *J* = 11.0 Hz, C*H*Ph), 4.55 (m, 2H, 2 x C*H*Ph), 4.44 (d, 1H, *J*_1,2_ = 3.7 Hz, H‐1), 4.36 (dd, 1H, *J*_5′,6a′_ = 3.5, *J*_6a′,6b′_ = 12.1 Hz, H‐6a′), 4.28 (dd, 1H, *J*_5′,6b′_ = 5.2 Hz, H‐6b′), 4.20 (dd, 1H, *J*_3,4_ = 9.3 Hz, H‐3), 4.16–4.11 (dd, 1H, *J*_5,6a_ = 4.4, *J*_6a,6b_ = 12.2 Hz, H‐6a), 3.72–3.46 (m, 7H, H‐2, 2′, 3′, 4, 5, 5′, 6b), 3.25 (s, 3H, OCH_3_) ppm; ^13^C NMR (101 MHz, CDCl_3_): *δ* 165.2, 164.3, 149.7, 147.4, 137.9, 137.2 (× 2), 136.7, 133.3, 129.8 (× 2), 129.2, 128.9, 128.5 (× 2), 128.4 (× 4), 128.2 (× 2), 128.1 (× 3), 128.0 (× 2), 127.8, 126.6, 125.9 (× 2), 125.4, 102.1, 101.1, 98.5, 80.2, 80.1, 79.3, 77.4, 75.0, 73.7, 71.4, 70.9, 68.8, 66.1, 64.1, 62.4, 55.3 ppm; ESI‐TOF [M + Na]^+^: calcd for [C_47_H_46_N_4_O_12_Na] 881.3010; found: 881.3010.

#### 4.3.14 6‐*O*‐(2‐Azido‐4‐*O*‐benzoyl‐3‐*O*‐benzyl‐2‐deoxy‐6‐*O*‐picoloyl‐*β‐*D‐glucopyranosyl)‐1,2:3,4‐di‐*O*‐isopropylidene‐*α*‐D‐galactopyranose (**31**)

The title compound was obtained as a colorless amorphous solid from glycosyl donor **10** and acceptor **17** in 82% yield (*β* only) under regular reaction conditions. Analytical data for **31**: R*_f_* 0.40 (acetone/toluene, 1/9, *v*/*v*); [*α*]^23^_D_ −72.1 (*c* = 1.0, CHCl_3_); ^1^H NMR (400 MHz, CDCl_3_): *δ* 8.62–8.58 (m, 1H, aromatic), 7.99–7.82 (m, 3H, aromatic), 7.68 (m, 1H, aromatic), 7.51–7.45 (m, 1H, aromatic), 7.36–7.30 (m, 3H, aromatic), 7.06 (m, 5H, aromatic), 5.47 (d, 1H, *J*_1,2_ = 5.0 Hz, H‐1), 5.33 (dd, 1H, *J*_4′,5′_ = 10.1 Hz, H‐4′), 4.71 (d, 1H, ^2^ *J* = 11.0 Hz, C*H*Ph), 4.56–4.49 (m, 3H, H‐1′, 4, C*H*Ph), 4.46 (m, 2H, H‐6a′, 6b′), 4.24 (dd, 1H, *J*_2,3_ = 7.9 Hz, H‐2), 4.16 (dd, 1H, *J*_3,4_ = 7.9 Hz, H‐3), 4.01–3.94 (m, 2H, H‐5, 6a), 3.86 (m, 1H, *J*_5′,6a′_ = 4.9, *J*_5′,6b′_ = 10.1 Hz, H‐5′), 3.75 (dd, 1H, *J*_5,6b_ = 6.6, *J*_6a,6b_ = 11.0 Hz, H‐6b), 3.57–3.48 (m, 2H, H‐2′, 3′), 1.48, 1.33, 1.27, 1.24 (4 s, 12H, 4 x CH_3_) ppm; ^13^C NMR (101 MHz, CDCl_3_): *δ* 165.2, 164.5, 149.8, 147.5, 137.2, 136.9, 133.4, 129.8 (× 2), 129.2, 128.5 (× 2), 128.3 (× 2), 128.1 (× 2), 127.8, 126.9, 125.2, 109.3, 108.7, 102.5, 96.3, 80.0, 75.1, 71.7, 71.2, 70.9, 70.6, 70.3, 69.0, 67.8, 66.2, 64.1, 26.0, 25.9, 24.9, 24.3 ppm; ESI‐TOF [M + Na]^+^: calcd for [C_38_H_42_N_4_O_12_Na] 769.2697; found: 769.2697.

#### 4.3.15 Benzyl 4‐*O*‐(2‐Azido‐4‐*O*‐benzoyl‐3‐*O*‐benzyl‐2‐deoxy‐6‐*O*‐picoloyl‐*β‐*D‐glucopyranosyl)‐2‐*O*‐benzoyl‐6‐*O*‐benzyl‐3‐*O*‐fluorenylmethoxycarbonyl‐*β*‐D‐glucopyranoside (**32**)

The title compound was obtained as a colorless amorphous solid from glycosyl donor **10** and acceptor **18** [37] in 24% yield (*β* only) under regular reaction conditions. Analytical data for **32**: R*_f_* 0.30 (acetone/toluene 1/9, *v*/*v*); [*α*]^23^_D_ −29.6 (*c* = 1.0, CHCl_3_); ^1^H NMR (CDCl_3_, 400 MHz): *δ* 8.50 (m, 1H, aromatic), 7.93–7.71 (m, 6H, aromatic), 7.53–7.21 (m, 16H, aromatic), 7.18–6.94 (m, 15H, aromatic), 5.28 (dd, 1H, *J*_2,3_ = 9.8 Hz, H‐2), 5.10 (m, 2H, *J*_3,4_ = 9.8, *J*_4′,5′_ = 10.2 Hz, H‐3, 4′), 4.82 (d, 1H, ^2^ *J* = 12.6 Hz, C*H*Ph), 4.71 (d, 1H, ^2^ *J* = 12.1 Hz, C*H*Ph), 4.63 (d, 1H, ^2^ *J* = 11.0 Hz, C*H*Ph), 4.60–4.53 (m, 2H, H‐1, C*H*Ph), 4.53–4.46 (m, 2H, 2 x C*H*Ph), 4.38–4.27 (m, 2H, H‐6a′, Fmoc), 4.18 (d, 1H, *J*_1′,2′_ = 8.1 Hz, H‐1′), 4.08–4.02 (m, 2H, H‐4, Fmoc), 3.99–3.90 (m, 2H, H‐6b′, Fmoc), 3.89–3.79 (m, 2H, H‐6a, 6b), 3.59–3.50 (m, 2H, H‐5, 5′), 3.39 (dd, 1H, *J*_2′,3′_ = 10.0 Hz, H‐2′), 3.31 (dd, 1H, *J*_3′,4′_ = 10.1 Hz, H‐3′) ppm; ^13^C NMR (176 MHz, CDCl_3_): *δ* 165.1, 165.0, 164.2, 154.7, 149.7, 147.3, 143.2, 143.0, 141.0, 140.9, 138.0, 137.2, 137.1, 136.7, 133.4, 133.2, 129.9 (× 2), 129.7 (× 2), 129.3, 129.1, 128.6 (× 2), 128.4 (× 2), 128.3 (× 4), 128.1 (× 2), 128.0 (× 2), 127.9, 127.8, 127.7, 127.6 (× 2), 127.5, 127.4, 126.9 (× 2), 126.7, 125.6, 124.9, 124.6, 119.7 (× 2), 101.3, 99.2, 80.2, 77.2 (× 2), 75.9, 75.0, 74.6, 73.5, 71.7, 71.6, 71.1, 70.5, 69.8, 67.5, 65.8, 63.8, 46.3, 29.7 ppm; ESI‐TOF [M + Na]^+^: calcd for [C_68_H_60_N_4_O_15_Na] 1195.3953; found: 1195.3967.

### 4.4 Synthesis of PNAG Tetrasaccharide Fragment 36. For the Spectra of All New Compounds, Refer to Figures S90–S103)

#### 4.4.1 Methyl 6‐*O*‐(2‐Azido‐4‐*O*‐benzoyl‐3‐*O*‐benzyl‐2‐deoxy‐*β‐*D‐glucopyranosyl)‐2,3,4‐tri‐*O*‐benzyl‐*α*‐D‐glucopyranoside (**33**)

Cu(OAc)_2_–H_2_O (14.3 mg, 0.079 mmol) was added to a solution of compound **26** (50 mg, 0.053 mmol) in CH_2_Cl_2_/MeOH (4.0 mL, 3/1, *v*/*v*) and the resulting mixture was stirred for 10 min at rt. After that, the reaction mixture was diluted with CH_2_Cl_2_ (∼10 mL) and washed with water (5 mL), sat. aq. NaHCO_3_ (5 mL) and water (5 mL). The organic phase was separated, dried with Na_2_SO_4_, and concentrated under reduced pressure. The residue was purified by column chromatography on silica gel (ethyl acetate–hexane gradient elution) to obtain the title compound as a colorless amorphous solid in 90% yield (40 mg, 0.047 mmol). Analytical data for **33**: R*_f_* 0.40 (ethyl acetate/hexane, 1/1, *v*/*v*); [*α*]^23^_D_ −16.4 (*c* = 1.0, CHCl_3_); ^1^H NMR (400 MHz, CDCl_3_): *δ* 7.97 (m, 2H, aromatic), 7.60 (m, 1H, aromatic), 7.45 (m, 2H, aromatic), 7.39–7.26 (m, 15H, aromatic), 7.25–7.05 (m, 5H, aromatic), 5.19 (dd, 1H, *J*_4′,5′_ = 9.26 Hz, H‐4′), 4.98 (dd, 2H, ^2^ *J* = 10.9 Hz, C*H*_2_Ph), 4.80 (dd, 3H, ^2^ *J* = 11.2 Hz, C*H*_2_Ph), 4.66 (dd, 3H, ^2^ *J* = 11.3 Hz, 3 x C*H*Ph), 4.62 (d, 1H, *J*_1,2_ = 4.4 Hz, H‐1), 4.29 (d, 1H, *J*_1′,2′_ = 7.8 Hz, H‐1′), 4.09 (dd, 1H, *J*_6a,6b_ = 11.3 Hz, H‐6a), 4.01 (dd, 1H, *J*_3,4_ = 9.2 Hz, H‐3), 3.86–3.73 (m, 2H, *J*_5,6a_ = 4.5 Hz, *J*_5,6b_ = 10.7 Hz, H‐5, 6b), 3.72–3.51 (m, 6H, H‐2, 2′, 3′, 4, 6a′, 6b′), 3.44 (m, 1H, *J*_5′,6a′_ = 5.3 Hz, *J*_5′,6b′_ = 10.2 Hz, H‐5′), 3.39 (s, 3H, OCH_3_), 2.40 (s, 1H, 6‐OH) ppm; ^13^C NMR (101 MHz, CDCl_3_): *δ* 165.8, 138.8, 138.5, 138.1, 137.2, 133.7, 129.9 (× 2), 129.0, 128.6 (× 2), 128.5 (× 2), 128.4 (× 2), 128.3 (× 2), 128.2 (× 2), 128.1 (× 2), 128.0 (× 2), 127.9 (× 2), 127.8 (× 2), 127.7, 127.6, 102.2, 98.3, 82.1, 80.2, 79.8, 77.6, 75.7, 75.0, 74.8, 74.6, 73.4, 70.9, 69.7, 68.7, 66.1, 61.5, 55.3 ppm; ESI‐TOF [M + Na]^+^: calcd for [C_48_H_51_N_3_O_11_Na] 868.3421; found: 868.3421.

#### 4.4.2 Methyl *O*‐(2‐Azido‐4‐*O*‐benzoyl‐3‐*O*‐benzyl‐2‐deoxy‐6‐*O*‐picoloyl‐*β*‐D‐glucopyranosyl)‐(1→6)‐*O*‐(2‐azido‐4‐*O*‐benzoyl‐3‐*O‐*benzyl‐2‐deoxy‐*β*‐D‐glucopyranosyl)‐(1→6)‐2,3,4‐tri‐*O*‐benzyl‐*α*‐D‐glucopyranoside (**34**)

A mixture containing glycosyl donor **10** (30 mg, 0.051 mmol), disaccharide acceptor **33** (30 mg, 0.035 mmol), and molec. sieves (3 Å, 90 mg) in CH_2_Cl_2_ (1.0 mL) was stirred under argon for 1 h at rt. The mixture was cooled to −30°C, NIS (23 mg, 0.101 mmol) and TfOH (0.8 µL, 0.010 mmol) were added, the external cooling was removed, and the resulting mixture was stirred under argon for 18 h at rt. After that, the solids were filtered off through a pad of Celite, rinsed successively with CH_2_Cl_2_, and the combined filtrate was concentrated under reduced pressure. The residue was redissolved in CH_2_Cl_2_ (∼20 mL) and washed with water (8 mL), 10% aq. Na_2_S_2_O_3_ (8 mL), and water (2 × 8 mL). The organic phase was separated, dried with Na_2_SO_4_, and concentrated under reduced pressure. The residue was purified by column chromatography on silica gel (ethyl acetate–hexane gradient elution) to obtain the title compound as a colorless amorphous solid in 45% yield (21 mg, 0.016 mmol, *β* only). Analytical data for **34**: R*_f_* 0.40 (ethyl acetate/hexane, 3.5/6.5, *v*/*v*); [*α*]^23^_D_ −15.8 (*c* = 1.0, CHCl_3_); ^1^H NMR (700 MHz, CDCl_3_): *δ* 8.66 (m, 1H, aromatic), 8.00–7.84 (m, 5H, aromatic), 7.76 (m, 1H, aromatic), 7.60–7.52 (m, 2H, aromatic), 7.44‐7.24 (m, 20H, aromatic), 7.16–7.04 (m, 10H, aromatic), 5.28 (dd, 1H, *J*_4″,5″_ = 10.1 Hz, H‐4″), 5.10 (dd, 1H, *J*_4′,5′_ = 9.5 Hz, H‐4′), 4.98 (dd, 2H, ^2^ *J* = 10.9 Hz, 2 x C*H*Ph), 4.84–4.78 (m, 2H, 2 x C*H*Ph), 4.76–4.64 (m, 5H, *J*_1,2_ = 3.4 Hz, H‐1, 4 x C*H*Ph), 4.59 (d, 1H, ^2^ *J* = 10.9 Hz, C*H*Ph), 4.52–4.43 (m, 2H, *J*_1″,2″_ = 7.6 Hz, H‐1″, C*H*Ph), 4.43–4.32 (m, 3H, H‐1′, H‐6a″, H‐6b″), 4.29 (dd, 1H, *J*_6a,6b_ = 10.9 Hz, H‐6a), 4.02 (dd, 1H, *J*_3,4_ = 9.3 Hz, H‐3), 3.93 (dd, 1H, *J*_6a′,6b′_ = 10.7 Hz, H‐6′), 3.88–3.82 (m, 2H, *J*_5,6a_ = 3.7 Hz, *J*_5,6b_ = 7.6 Hz, H‐5, 6b), 3.80–3.68 (m, 3H, H‐5′, 5″, 6b′), 3.65–3.55 (m, 4H, H‐2, 2′, 3′, 4), 3.49–3.38 (m, 5H, H‐2″, 3″, OCH_3_) ppm; ^13^C NMR (176 MHz, CDCl_3_): *δ* 165.3, 165.1, 164.4, 149.9, 138.8, 138.5, 138.1, 137.0, 133.6, 133.5, 129.8 (× 2), 129.0, 129.1, 128.5 (× 6), 128.4 (× 2), 128.3 (× 4), 128.2 (× 2), 128.1 (× 2), 128.0 (× 2), 127.9 (× 4), 127.8 (× 2), 127.7, 127.6, 126.9, 125.3, 102.7, 101.9, 98.2, 82.0, 80.3, 79.8, 79.7, 77.7, 77.2, 75.7, 75.1, 75.0, 74.9, 73.9, 73.5, 71.8, 70.9, 70.7, 69.7, 69.2, 68.2, 66.1, 65.9, 63.8, 55.2, 31.9, 29.7 (× 2), 29.4, 22.7, 14.1 ppm; ESI‐TOF [M + Na]^+^: calcd for [C_74_H_73_N_7_O_17_Na] 1354.4961; found: 1354.4960.

#### 4.4.3 Methyl *O*‐(2‐Azido‐4‐*O*‐benzoyl‐3‐*O*‐benzyl‐2‐deoxy‐*β*‐D‐glucopyranosyl)‐(1→6)‐*O*‐(2‐azido‐4‐*O*‐benzoyl‐3‐*O‐*benzyl‐2‐deoxy‐*β*‐D‐glucopyranosyl)‐(1→6)‐2,3,4‐tri‐*O*‐benzyl‐*α*‐D‐glucopyranoside (**35**)

Cu(OAc)_2_─H_2_O (3.7 mg, 0.020 mmol) was added to a solution of compound **34** (18 mg, 0.013 mmol) in CH_2_Cl_2_/MeOH (2.0 mL, 3/1, *v*/*v*) and the resulting mixture was stirred for 10 min at rt. After that, the reaction mixture was diluted with CH_2_Cl_2_ (∼10 mL) and washed with water (5 mL), sat. aq. NaHCO_3_ (5 mL), and water (5 mL). The organic phase was separated, dried with Na_2_SO_4_, and concentrated under reduced pressure. The residue was purified by column chromatography on silica gel (ethyl acetate–hexane gradient elution) to obtain the title compound as a colorless amorphous solid in 87% yield (14.3 mg, 0.012 mmol). Analytical data for **35**: R*_f_* 0.70 (ethyl acetate/hexane, 1/1, *v*/*v*); [*α*]^23^_D_ −35.5 (*c* = 1.0, CHCl_3_); ^1^H NMR (700 MHz, CDCl_3_): *δ* 8.02–7.95 (m, 2H, aromatic), 7.95–7.88 (m, 2H, aromatic), 7.65–7.55 (m, 2H, aromatic), 7.51–7.24 (m, 19H, aromatic), 7.16–7.12 (m, 6H, aromatic), 7.12–7.05 (m, 3H, aromatic), 5.16 (dd, 1H, *J*_4′,5′_ = 9.1 Hz, H‐4′), 5.05 (dd, 1H, *J*_4″,5″_ = 9.5 Hz, H‐4″), 4.98 (dd, 2H, ^2^ *J* = 11.0 Hz, C*H*_2_Ph), 4.87–4.57 (m, 8H, H‐1, 7 x C*H*Ph), 4.53 (d, 1H, ^2^ *J* = 11.1 Hz, C*H*Ph), 4.38 (d, 1H, *J*_1″,2″_ = 8.0 Hz, H‐1″), 4.36–4.21 (m, 2H, H‐1′, 6a), 4.03 (dd, 1H, *J*_3,4_ = 9.3 Hz, H‐3), 3.97 (dd, 1H, *J*_6a′,6b′_ = 10.6 Hz, H‐6a′), 3.84 (dd, 2H, *J*_5,6a_ = 3.4 Hz, *J*_5,6b_ = 10.7 Hz, H‐5, 6b), 3.78–3.68 (m, 2H, H‐5′, 6b′), 3.66–3.53 (m, 5H, H‐2, 2′, 3′, 4, 6a″), 3.50–3.38 (m, 5H, H‐5″, 6b″, OCH_3_), 3.32 (m, 2H, *J*_3″,4″_ = 9.8 Hz, H‐2″, 3″), 2.59 (s, 1H, 6″‐OH) ppm; ^13^C NMR (176 MHz, CDCl_3_): *δ* 165.9, 165.6, 138.7, 138.5, 138.1, 137.2, 137.0, 133.7, 129.8 (× 2), 129.1, 128.8, 128.6, 128.5 (× 3), 128.4 (× 3), 128.3 (× 6), 128.2 (× 4), 128.1 (× 2), 127.9 (× 6), 127.8, 127.7 (× 3), 127.5, 102.2, 101.9, 98.2, 82.0, 80.1, 79.7, 79.6, 77.7, 77.1, 75.7, 75.1, 74.9, 74.2, 73.6, 73.4, 71.5, 70.5, 69.7, 69.2, 68.3, 66.1, 65.8, 60.9, 55.2, 29.7, 14.1 ppm; ESI‐TOF [M + Na]^+^: calcd for [C_68_H_70_N_6_O_16_Na] 1249.4746; found: 1249.4746.

#### 4.4.4 Methyl *O*‐(2‐Azido‐4‐*O*‐benzoyl‐3‐*O*‐benzyl‐2‐deoxy‐6‐*O*‐picoloyl‐*β*‐D‐glucopyranosyl)‐(1→6)‐*O*‐(2‐azido‐4‐*O*‐benzoyl‐3‐*O*‐benzyl‐2‐deoxy‐*β*‐D‐glucopyranosyl)‐(1→6)‐*O*‐(2‐azido‐4‐*O*‐benzoyl‐3‐*O‐*benzyl‐2‐deoxy‐*β*‐D‐glucopyranosyl)‐(1→6)‐2,3,4‐tri‐*O*‐benzyl‐*α*‐D‐glucopyranoside (**36**)

A mixture containing glycosyl donor **10** (11 mg, 0.018 mmol), disaccharide acceptor **35** (14 mg, 0.012 mmol), and molec. sieves (3 Å, 33 mg) in CH_2_Cl_2_ (0.5 mL) was stirred under argon for 1 h at rt. The mixture was cooled to −30°C, NIS (5.6 mg, 0.025 mmol) and TfOH (0.2 µL, 0.002 mmol) were added, the external cooling was removed, and the resulting mixture was stirred under argon for 18 h at rt. After that, the solids were filtered off through a pad of Celite, rinsed successively with CH_2_Cl_2_, and the combined filtrate was concentrated under reduced pressure. The residue was redissolved in CH_2_Cl_2_ (∼20 mL) and washed with water (8 mL), 10% aq. Na_2_S_2_O_3_ (8 mL), and water (2 × 8 mL). The organic phase was separated, dried with Na_2_SO_4_, and concentrated under reduced pressure. The residue was purified by column chromatography on silica gel (ethyl acetate–hexane gradient elution) to obtain the title compound as a colorless amorphous solid in 51% yield (10 mg, 0.006 mmol, *β* only). Analytical data for **36**: R*_f_* = 0.40 (ethyl acetate/hexane, 3.5/6.5, *v*/*v*); [*α*]^23^_D_ −29.9 (*c* = 1.0, CHCl_3_); ^1^H NMR (700 MHz, CDCl_3_): *δ* 8.68 (m, 1H, aromatic), 8.05–8.00 (m, 2H, aromatic), 7.96 (m, 2H, aromatic), 7.91 (m, 2H, aromatic), 7.78 (m, 1H, aromatic), 7.61–7.52 (m, 3H, aromatic), 7.48–7.41 (m, 4H, aromatic), 7.41–7.37 (m, 6H, aromatic), 7.37–7.35 (m, 1H, aromatic), 7.33 (m, 5H, aromatic), 7.32–7.30 (m, 1H, aromatic), 7.30–7.25 (m, 2H, aromatic), 7.18–7.08 (m, 10H, aromatic), 7.08–7.05 (m, 1H, aromatic), 5.33 (dd, 1H, *J*_4‴,5‴_ = 10.5 Hz, H‐4‴), 5.23 (dd, 1H, *J*_4′,5′_ = 10.5 Hz, H‐4′), 5.00 (dd, 2H, ^2^ *J* = 11.0 Hz, C*H*_2_Ph), 4.91 (dd, 1H, *J*_4″,5″_ = 10.0 Hz, H‐4″), 4.83 (dd, 3H, ^2^ *J* = 11.2 Hz, 3 x C*H*Ph), 4.78 (d, 1H, ^2^ *J* = 11.0 Hz, C*H*Ph), 4.74 (d, 1H, ^2^ *J* = 11.1 Hz, C*H*Ph), 4.70–4.60 (m, 5H, H‐1, 4 x C*H*Ph), 4.53 (d, 1H, *J*_1‴,2‴_ = 8.1 Hz, H‐1‴), 4.52–4.45 (m, 3H, *J*_1″,2″_ = 7.9 Hz, H‐1′′, 6a‴, C*H*Ph), 4.42 (dd, 1H, *J*_6a‴,6b‴_ = 12.1 Hz, H‐6b‴), 4.37 (d, 1H, *J*_1′,2′_ = 7.8 Hz, H‐1′), 4.29 (dd, 1H, *J*_6a,6b_ = 12.0 Hz, H‐6a), 4.10 (dd, 1H, *J*_6a′,6b′_ = 11.2 Hz, H‐6a′), 4.05 (dd, 1H, *J*_3,4_ = 9.6 Hz, H‐3), 3.91–3.80 (m, 5H, H‐5, 5‴, 6a″, 6b, 6b′), 3.77 (m, 1H, *J*_5′,6a′_ = 7.1 Hz, *J*_5′,6b′_ = 10.2 Hz, H‐5′), 3.70–3.56 (m, 7H, H‐2, 2′, 3′, 3‴, 4, 5″, 6b″), 3.45–3.33 (m, 5H, H‐2‴, 3″, OCH_3_), 3.27 (dd, 1H*, J*_2″,3″_ = 9.9 Hz, H‐2″) ppm; ^13^C NMR (176 MHz, CDCl_3_): *δ* 165.3, 165.2 (× 2), 164.4, 149.9, 147.4, 138.8, 138.4, 138.1, 137.4, 137.1 (× 2), 137.0, 133.6 (× 2), 133.3, 129.8 (× 4), 129.7 (× 2), 129.3, 129.2, 129.0, 128.6, 128.5 (× 3), 128.4 (× 3), 128.3 (× 4), 128.2 (× 6), 128.1 (× 4), 127.9 (× 5), 127.8 (× 3), 127.7 (× 2), 127.5, 126.8, 125.4, 102.5, 102.2, 102.0, 98.2, 82.0, 80.2, 79.8 (× 2), 79.6, 77.7, 75.7, 75.2, 75.1, 74.9, 74.8, 74.0, 73.8, 73.5, 71.7, 71.6, 70.8, 70.7, 69.7, 69.2, 68.8, 68.3, 66.1, 65.9 (× 2), 63.9, 55.2, 31.9, 29.6, 22.7, 14.1 ppm; ESI‐TOF [M + Na]^+^: calcd for [C_94_H_92_N_10_O_22_Na] 1736.6364; found: 1736.6296.

## Author Contributions

The study was written through contributions of all authors. All authors have given approval to the final version of the study.

## Conflicts of Interest

The authors declare no conflicts of interest.

## Supporting information




**NMR spectra for all new compounds**.
**Supporting File 1**: asia70928‐sup‐0001‐SuppMat.pdf.

## Data Availability

The data that supports the findings of this study are available in the Supporting Information of this article.

## References

[1] G. Paulíková , J. Houser , M. Kašáková , et al., “Fucosylated Inhibitors of Recently Identified Bangle Lectin from Photorhabdus Asymbiotica,” Scientific Reports 9 (2019): 14904, 10.1038/s41598-019-51357-9. 31624296 PMC6797808

[2] E. Fujdiarová , J. Houser , P. Dobeš , et al., “Heptabladed *β*‐Propeller Lectins PLL2 and PHL from Photorhabdus spp. Recognize O ‐Methylated Sugars and Influence the Host Immune System,” FEBS Journal 288 (2021): 1343–1365, 10.1111/febs.15457. 32559333

[3] D. Crich , “Mechanism of a Chemical Glycosylation Reaction,” Accounts of Chemical Research 43 (2010): 1144–1153, 10.1021/ar100035r. 20496888

[4] L. K. Mydock and A. V. Demchenko , “Mechanism of Chemical O‐Glycosylation: From Early Studies to Recent Discoveries,” Organic & Biomolecular Chemistry 8 (2010): 497–510, 10.1039/B916088D. 20090962

[5] M. P. Mannino and A. V. Demchenko , “Hydrogen‐bond‐mediated Aglycone Delivery (HAD) and Related Methods in Carbohydrate Chemistry,” in Carbohydrate Chemistry: Chemical and Biological Approaches (Specialist Periodical Reports), eds. A. P. Rauter , T. K. Lindhorst , and Y. Queneau , (RSC, 2021), 93–116.

[6] A. Khanam and P. Kumar Mandal , “Influence of Remote Picolinyl and Picoloyl Stereodirecting Groups for the Stereoselective Glycosylation,” Asian Journal of Organic Chemistry 10 (2021): 296–314, 10.1002/ajoc.202000558.

[7] J. P. Yasomanee and A. V. Demchenko , “Effect of Remote Picolinyl and Picoloyl Substituents on the Stereoselectivity of Chemical Glycosylation,” Journal of the American Chemical Society 134 (2012): 20097–20102, 10.1021/ja307355n. 23167454

[8] M. Ravenscroft , R. M. G. Roberts , and J. G. Tillett , “The Reaction of some Cyclic and Open‐chain Disulphides with Methyl Trifluoromethanesulphonate,” Journal of the Chemical Society, Perkin Transactions 2 11 (1982): 1569, 10.1039/p29820001569.

[9] F. Andersson , P. Fúgedi , P. J. Garegg , and M. Nashed , “Synthesis of 1,2–linked Glycosides Using Dimethyl(methylthio) Sulfonium Triplate as Promoter and Thioglycosides as Glycosyl Donors,” Tetrahedron Letters 27 (1986): 3919–3922, 10.1016/S0040-4039(00)83917-9.

[10] P. Konradsson , D. R. Mootoo , R. E. McDevitt , and B. Fraser‐Reid , “Iodonium Ion Generated In Situ from N‐iodosuccinimide and Trifluoromethanesulphonic Acid Promotes Direct Linkage of ‘Disarmed’ pent‐4‐enyl Glycosides,” Journal of the Chemical Society, Chemical Communications 1990, 270–272, 10.1039/C39900000270.

[11] G. H. Veeneman , S. H. van Leeuwen , and J. H. van Boom , “Iodonium Ion Promoted Reactions at the Anomeric Centre. II an Efficient Thioglycoside Mediated Approach Toward the Formation of 1,2‐trans Linked Glycosides and Glycosidic Esters,” Tetrahedron Letters 31 (1990): 1331–1334, 10.1016/S0040-4039(00)88799-7.

[12] M. P. Mannino , J. P. Yasomanee , and A. V. Demchenko , “Investigation of the H‐Bond‐Mediated Aglycone Delivery Reaction in Application to the Synthesis of *β*‐glucosides,” Carbohydrate Research 470 (2018): 1–7, 10.1016/j.carres.2018.09.003. 30286335 PMC6215728

[13] S. G. Pistorio , J. P. Yasomanee , and A. V. Demchenko , “Hydrogen‐Bond‐Mediated Aglycone Delivery: Focus on *β*‐Mannosylation,” Organic Letters 16 (2014): 716–719, 10.1021/ol403396j. 24471826

[14] J. P. Yasomanee and A. V. Demchenko , “Hydrogen Bond Mediated Aglycone Delivery: Synthesis of Linear and Branched α‐Glucans,” Angewandte Chemie International Edition 53 (2014): 10453–10456, 10.1002/anie.201405084. 25115776

[15] C. Alex , S. Visansirikul , and A. V. Demchenko , “A Versatile Approach to the Synthesis of Glycans Containing Mannuronic Acid Residues,” Organic & Biomolecular Chemistry 19 (2021): 2731–2743, 10.1039/D1OB00188D. 33687051

[16] M. P. Mannino and A. V. Demchenko , “Synthesis of *β*‐Glucosides With 3‐ O ‐Picoloyl‐Protected Glycosyl Donors in the Presence of Excess Triflic Acid: a Mechanistic Study,” Chemistry—A European Journal 26 (2020): 2927–2937, 10.1002/chem.201905277. 31886924

[17] M. P. Mannino and A. V. Demchenko , “Synthesis of *β*‐Glucosides with 3‐ O ‐Picoloyl‐Protected Glycosyl Donors in the Presence of Excess Triflic Acid: Defining the Scope,” Chemistry—A European Journal 26 (2020): 2938–2946, 10.1002/chem.201905278. 31886911

[18] C. Alex , S. Visansirikul , and A. V. Demchenko , “A Versatile Approach to the Synthesis of Mannosamine Glycosides,” Organic & Biomolecular Chemistry 18 (2020): 6682–6695, 10.1039/D0OB01640C. 32813001

[19] C. Alex and A. V. Demchenko , “Direct Synthesis of Glycans Containing Challenging ManNAcA Residues,” Journal of Organic Chemistry 87 (2022): 271–280, 10.1021/acs.joc.1c02351. 34928148

[20] C. Alex and A. V. Demchenko , “Recent Advances in Stereocontrolled Mannosylation: Focus on Glycans Comprising Acidic and/or Amino Sugars,” Chemical Record 21 (2021): 3278–3294, 10.1002/tcr.202100201. 34661961

[21] M. Novakova , A. Das , C. Alex , and A. V. Demchenko , “Synthesis and Glycosidation of Building Blocks of D‐altrosamine,” Frontiers in Chemistry 10 (2022): 945779, 10.3389/fchem.2022.945779. 36226114 PMC9548543

[22] S. A. Geringer , M. P. Mannino , M. D. Bandara , and A. V. Demchenko , “Picoloyl Protecting Group in Synthesis: Focus on a Highly Chemoselective Catalytic Removal,” Organic & Biomolecular Chemistry 18 (2020): 4863–4871, 10.1039/D0OB00803F. 32608450 PMC7656231

[23] K. Skarbek and M. J. Milewska , “Biosynthetic and Synthetic Access to Amino Sugars,” Carbohydrate Research 434 (2016): 44–71, 10.1016/j.carres.2016.08.005. 27592039

[24] R. A. Dwek , “Glycobiology: Toward Understanding the Function of Sugars,” Chemical Reviews 96 (1996): 683–720, 10.1021/cr940283b. 11848770

[25] A. F. G. Bongat and A. V. Demchenko , “Recent Trends in the Synthesis of O‐Glycosides of 2‐Amino‐2‐deoxysugars,” Carbohydrate Research 342 (2007): 374–406, 10.1016/j.carres.2006.10.021. 17125757

[26] X. Y. Zhou , L. X. Li , Z. Zhang , et al., “Chemical Synthesis and Antigenic Evaluation of Inner Core Oligosaccharides From Acinetobacter baumannii Lipopolysaccharide,” Angewandte Chemie International Edition 61 (2022): e202204420, 10.1002/anie.202204420. 35543248

[27] G. Anilkumar , L. G. Nair , L. Olsson , J. K. Daniels , and B. Fraser‐Reid , “α‐Glucosaminide Synthesis: Exercising Stereocontrol at C1 or C2 via Torsional Effects or DeShong Nucleophiles,” Tetrahedron Letters 41 (2000): 7605–7608, 10.1016/S0040-4039(00)01329-0.

[28] B. Fraser‐Reid , Z. Wu , C. W. Andrews , E. Skowronski , and J. P. Bowen , “Torsional Effects in Glycoside Reactivity: Saccharide Couplings Mediated by Acetal Protecting Groups,” Journal of the American Chemical Society 113 (1991): 1434–1435, 10.1021/ja00004a066.

[29] H. H. Jensen , L. U. Nordstrøm , and M. Bols , “The Disarming Effect of the 4,6‐Acetal Group on Glycoside Reactivity: Torsional or Electronic?,” Journal of the American Chemical Society 126 (2004): 9205–9213, 10.1021/ja047578j. 15281809

[30] Y. C. Yeh , H. J. Kim , and H.‐W. Liu , “Mechanistic Investigation of 1,2‐Diol Dehydration of Paromamine Catalyzed by the Radical S ‐Adenosyl‐ l ‐Methionine Enzyme AprD4,” Journal of the American Chemical Society 143 (2021): 5038–5043, 10.1021/jacs.1c00076. 33784078 PMC8045149

[31] S. C. Ranade , S. Kaeothip , and A. V. Demchenko , “Glycosyl Alkoxythioimidates as Complementary Building Blocks for Chemical Glycosylation,” Organic Letters 12 (2010): 5628–5631, 10.1021/ol1023079. 21087037 PMC3005820

[32] F. Zhang , W. Zhang , Y. Zhang , D. P. Curran , and G. Liu , “Synthesis and Applications of a Light‐Fluorous Glycosyl Donor,” Journal of Organic Chemistry 74 (2009): 2594–2597, 10.1021/jo9000993. 19216499 PMC2754202

[33] S. van der Vorm , T. Hansen , J. M. A. van Hengst , H. S. Overkleeft , G. A. van der Marel , and J. D. C. Codee , “Acceptor Reactivity in Glycosylation Reactions,” Chemical Society Reviews 48 (2019): 4688–4706. 31287452 10.1039/c8cs00369f

[34] W. Pilgrim and P. V. Murphy , “SnCl 4 ‐ and TiCl 4 ‐Catalyzed Anomerization of Acylated O—And S ‐Glycosides: Analysis of Factors That Lead to Higher α:*Β* Anomer Ratios and Reaction Rates,” Journal of Organic Chemistry 75 (2010): 6747–6755, 10.1021/jo101090f. 20836488

[35] B. Fraser‐Reid , Z. Wu , U. E. Udodong , and H. Ottosson , “Armed/Disarmed Effects in Glycosyl Donors: Rationalization and Sidetracking,” Journal of Organic Chemistry 55 (1990): 6068–6070, 10.1021/jo00312a004.

[36] D. B. Konrad , K. P. Rühmann , H. Ando , et al., “A Concise Synthesis of Tetrodotoxin,” Science 377 (2022): 411–415, 10.1126/science.abn0571. 35862530

[37] F. Pooladian , A. Das , J. W. Wise , and A. V. Demchenko , “Synthesis of Regioselectively Protected Building Blocks of Benzyl *β*‐d‐glucopyranoside,” Carbohydrate Research 544 (2024): 109250, 10.1016/j.carres.2024.109250. 39214041 PMC11391699

[38] B. Hagen , S. Ali , H. S. Overkleeft , G. A. van der Marel , and J. D. C. Codée , “Mapping the Reactivity and Selectivity of 2‐Azidofucosyl Donors for the Assembly of N ‐Acetylfucosamine‐Containing Bacterial Oligosaccharides,” Journal of Organic Chemistry 82 (2017): 848–868, 10.1021/acs.joc.6b02593. 28051314 PMC5332126

[39] A. B. Ingle , C. S. Chao , W. C. Hung , and K. K. T. Mong , “Tuning Reactivity of Glycosyl Imidinium Intermediate for 2‐Azido‐2‐deoxyglycosyl Donors in α‐Glycosidic Bond Formation,” Organic Letters 15 (2013): 5290–5293, 10.1021/ol402519c. 24107114

[40] X. Lu , G. Li , J. Pang , et al., “Antibodies Targeting a Conserved Surface Polysaccharide Are Protective Against a Wide Range of Microbial Pathogens Producing *β*‐1–6‐Linked Poly‐N‐Acetylglucosamine (PNAG),” Engineering 38 (2024): 69–76, 10.1016/j.eng.2023.09.012.

[41] A. V. Demchenko , “1,2‐cis O‐Glycosylation: Methods, Strategies, Principles,” Current Organic Chemistry 7 (2003): 35–79, 10.2174/1385272033373175.

[42] A. E. Christina , G. A. van der Marel , and J. D. C. Codee , “Recent Developments in the Construction of Cis‐Glycosidic Linkages,” in Modern Synthetic Methods in Carbohydrate Chemistry, eds. D. B. Werz and S. Vidal (Wiley‐VCH Verlag GmbH & Co. KGaA, 2014), 97–124.

[43] S. S. Nigudkar and A. V. Demchenko , “Stereocontrolled 1,2‐cis Glycosylation as the Driving Force of Progress in Synthetic Carbohydrate Chemistry,” Chemical Science 6 (2015): 2687–2704, 10.1039/C5SC00280J. 26078847 PMC4465199

[44] R. A. Mensink and T. J. Boltje , “Advances in Stereoselective 1,2‐ Cis Glycosylation Using C‐2 Auxiliaries,” Chemistry—A European Journal 23 (2017): 17637–17653, 10.1002/chem.201700908. 28741787

[45] D. Takahashi , K. Inaba , and K. Toshima , “Recent Advances in Boron‐mediated Aglycon Delivery (BMAD) for the Efficient Synthesis of 1,2‐cis Glycosides,” Carbohydrate Research 518 (2022): 108579, 10.1016/j.carres.2022.108579. 35598560

[46] G. Shrestha , G. A. Kashiwagi , K. J. Stine , and A. V. Demchenko , “Streamlined Access to Carbohydrate Building Blocks: Methyl 2,4,6‐tri‐O‐benzyl‐α‐d‐glucopyranoside,” Carbohydrate Research 511 (2022): 108482, 10.1016/j.carres.2021.108482. 34856429 PMC8792249

