# Supplementary material for: Application of the Hydrogen‐Bond‐Mediated Aglycone Delivery (HAD) Reaction to the Synthesis of α‐ and β‐Linked 2‐Azido‐2‐deoxyglucosides
Source: Chem Asian J. 2026 Aug 9;21(15):e70928. doi: 10.1002/asia.70928 (PMC13454313; doi:10.1002/asia.70928)
Supplement: Supplementary file 1 [file ASIA-21-e70928-s001.pdf]

Supporting Information

Application of the Hydrogen-bond-mediated Aglycone Delivery (HAD) Reaction to the Synthesis of α- and β-Linked 2-Azido-2-deoxyglucosides

*Alessandra Damico, Connor R. Mathus, and Alexei V. Demchenko**

§ Department of Chemistry, Saint Louis University, 3501 Laclede Ave, St. Louis, Missouri, 63103, USA
e-mail: alexei.demchenko@slu.edu

Contents

Spectra of New Monosaccharides 2
Spectra of New Disaccharides 20
Spectra of PNAG tetrasaccharide fragment 36 and all intermediates 91

Spectra of New Monosaccharides

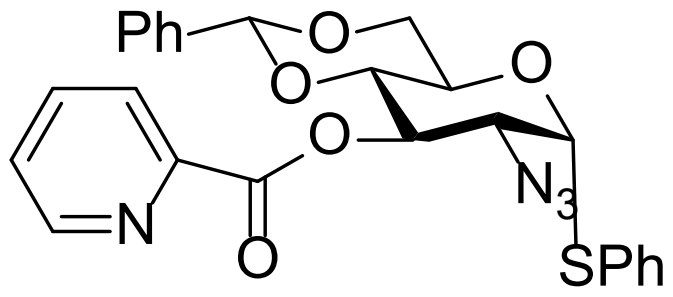

2

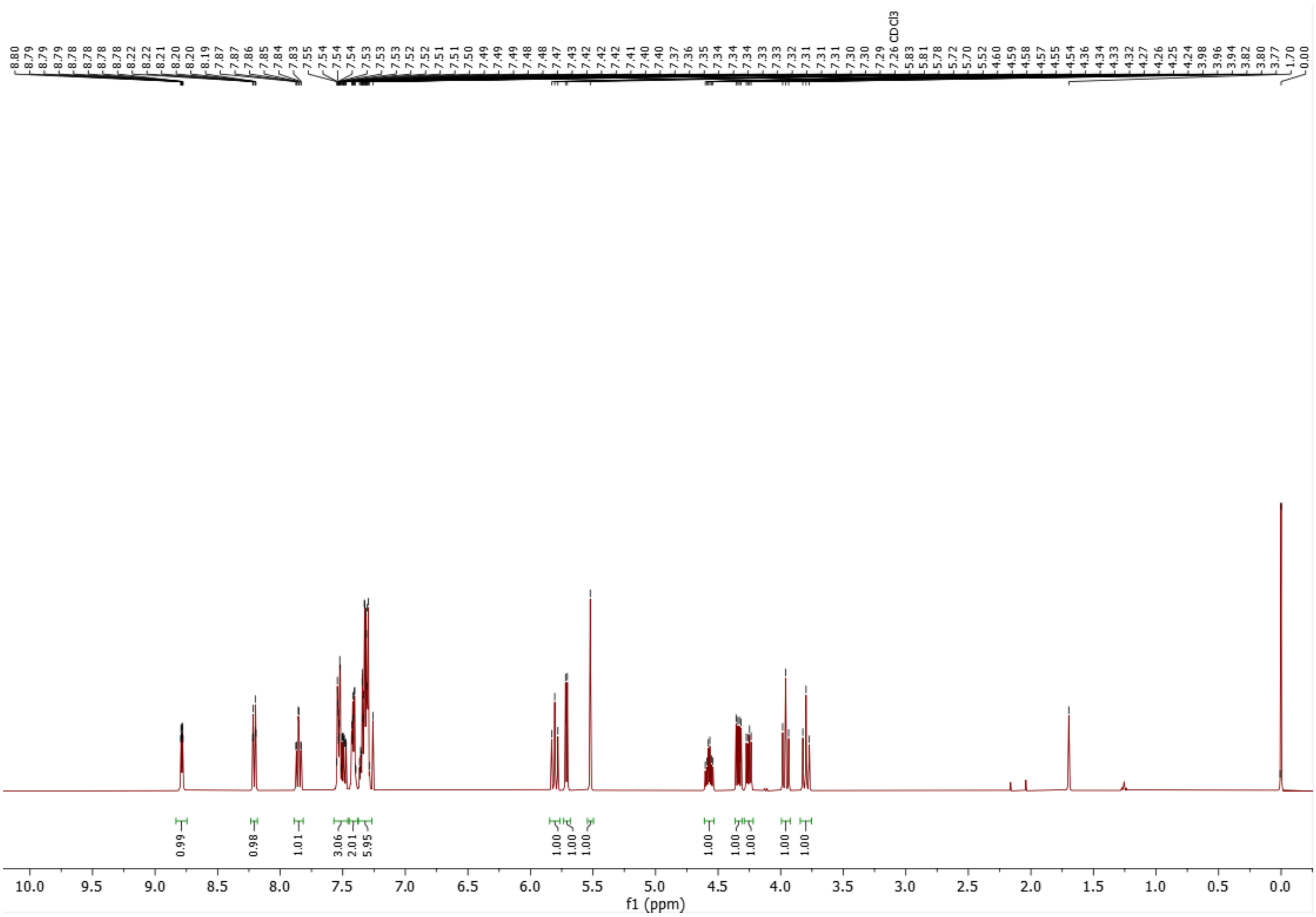

Figure S1: ^{1}H NMR (400 MHz, $CDCl_3$)

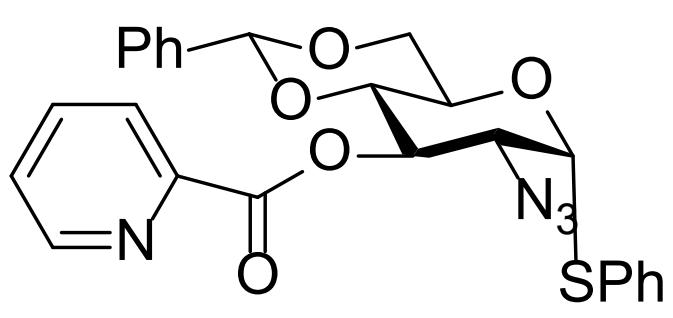

2

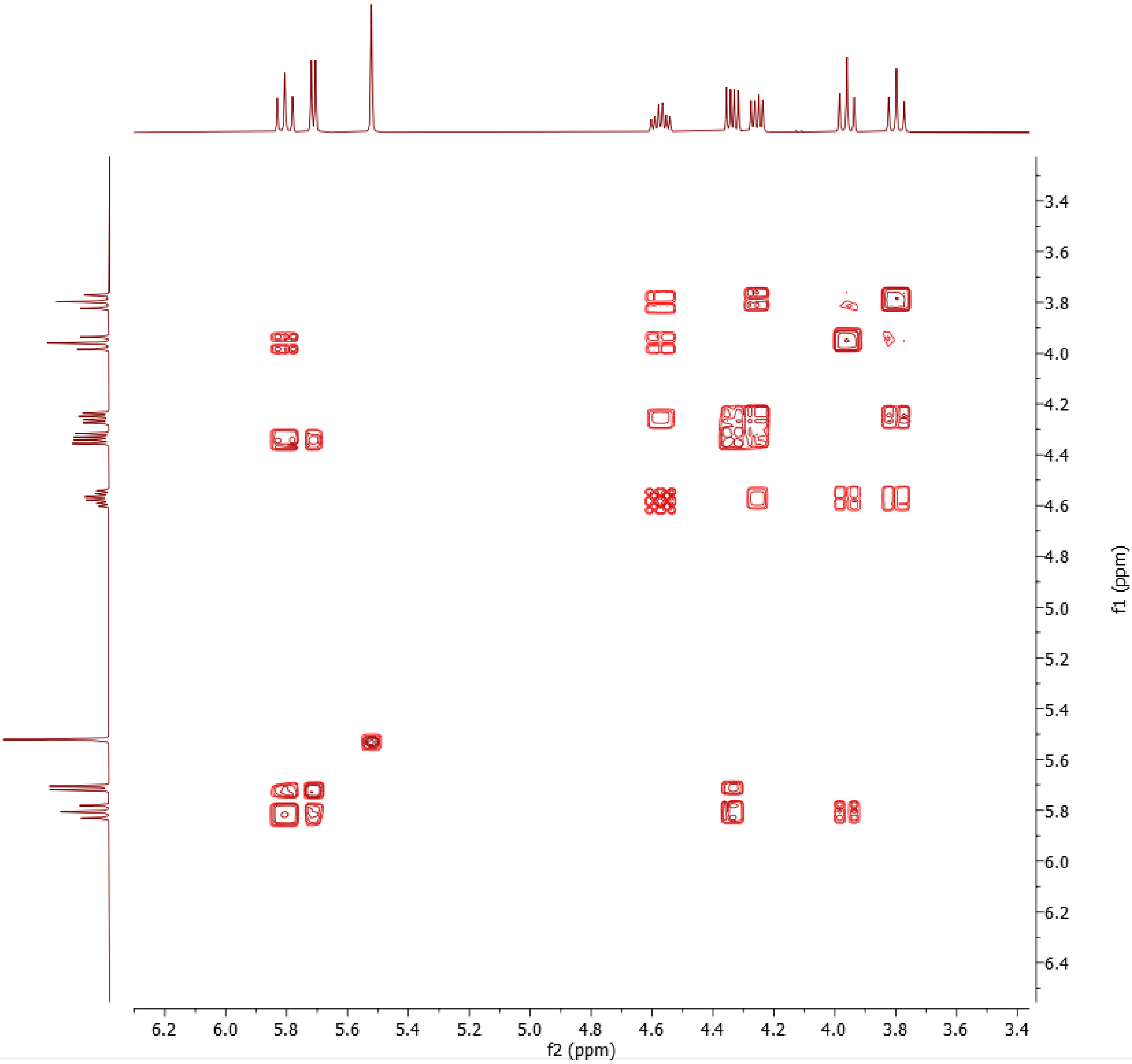

Figure S2: COSY NMR (400 MHz, $CDCl_3$)

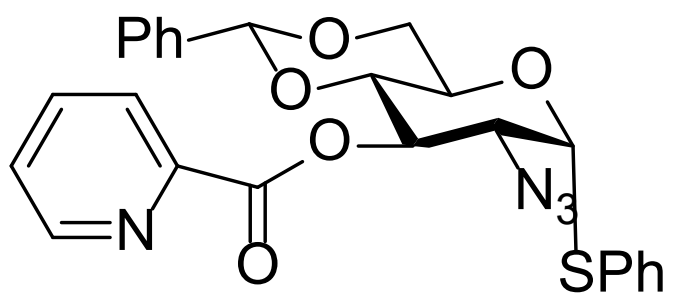

2

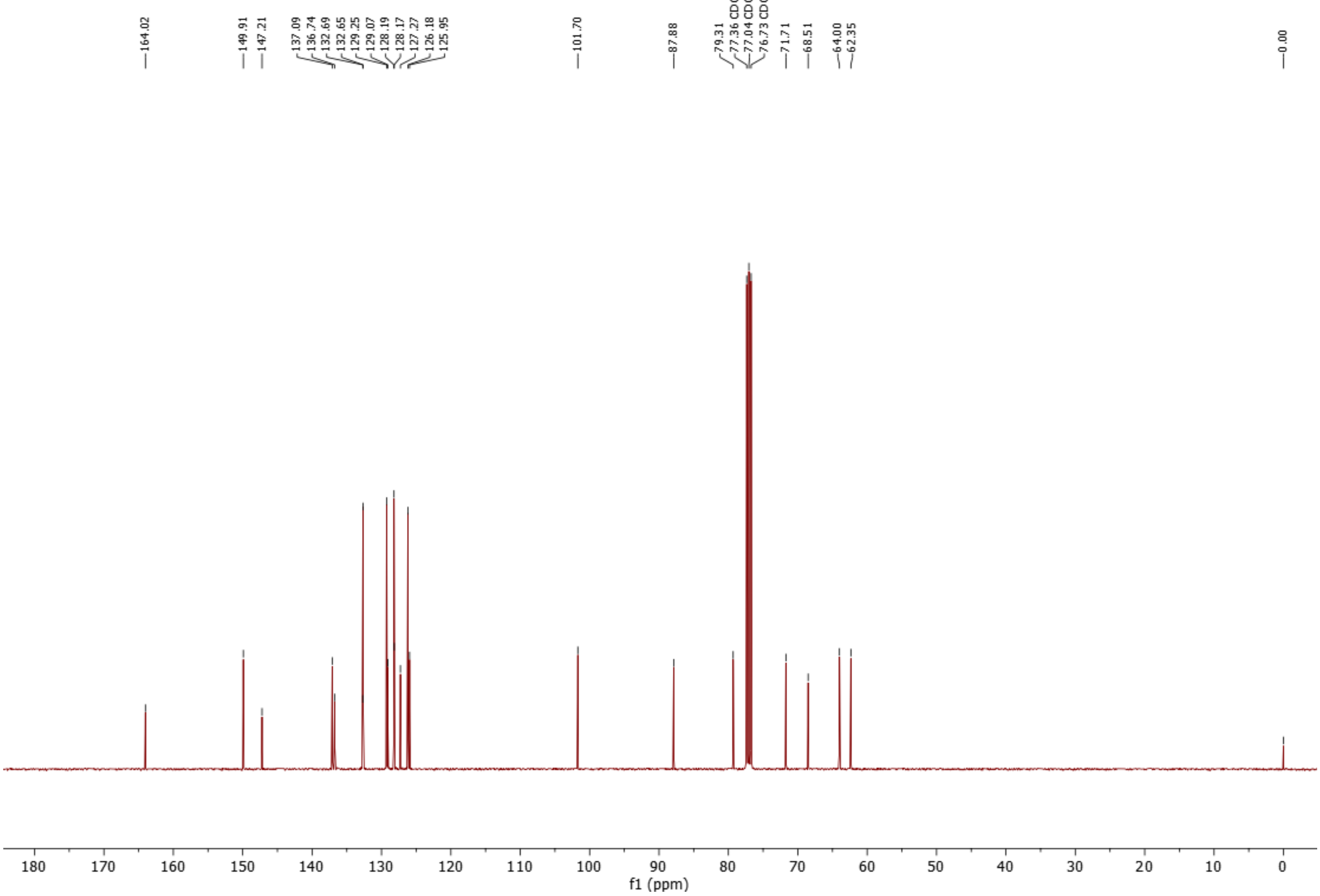

Figure S3: ^{13}C NMR (101 MHz, $CDCl_3$)

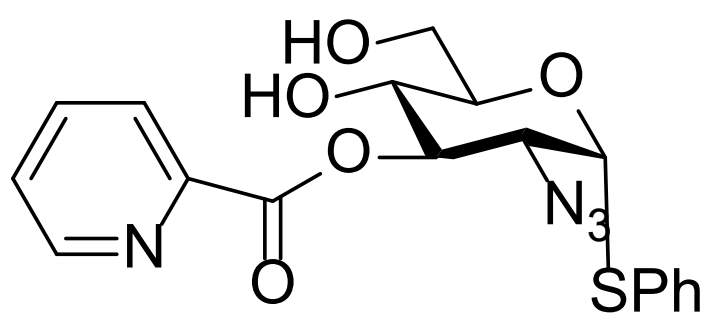

3

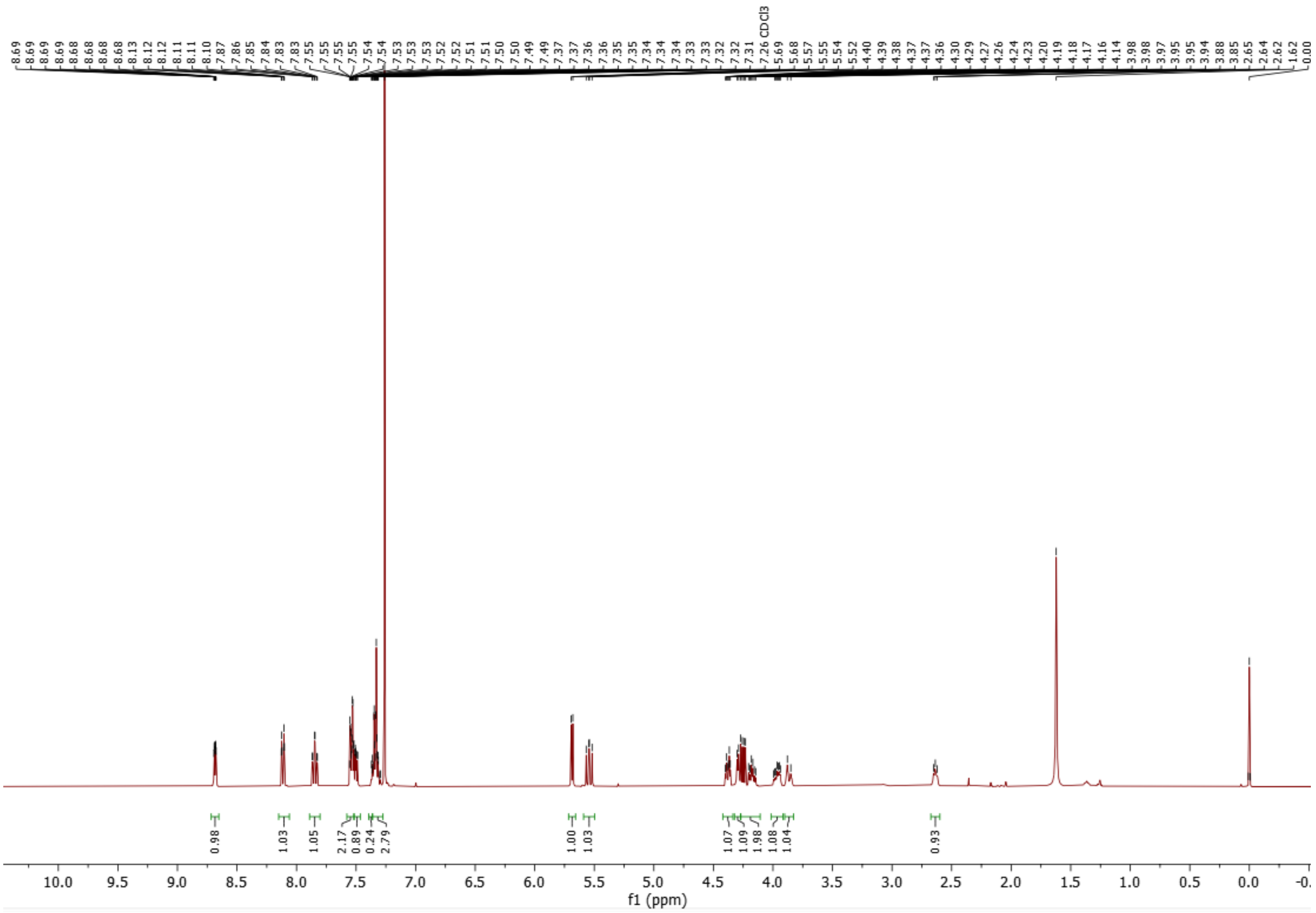

Figure S4: ^{1}H NMR (400 MHz, $CDCl_3$)

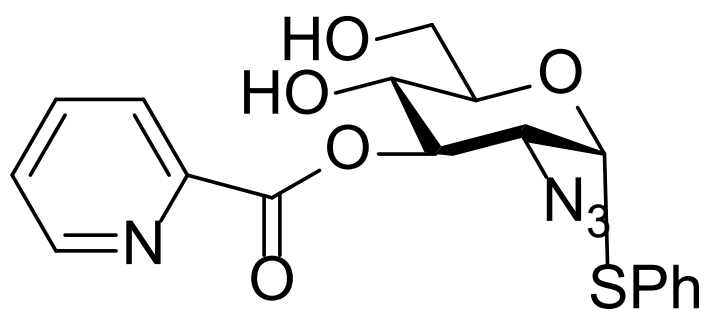

3

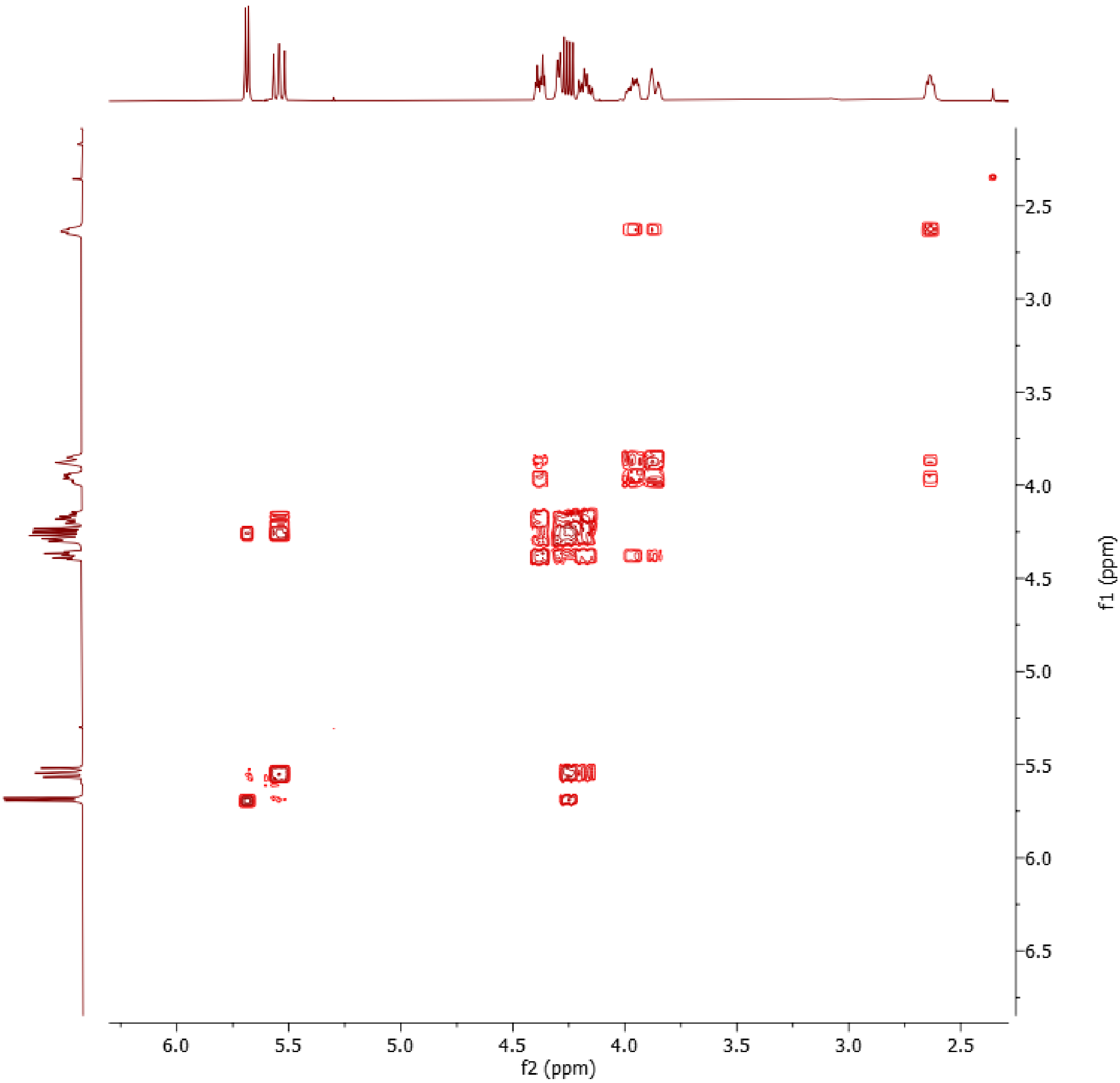

Figure S5: COSY NMR (400 MHz, $CDCl_3$)

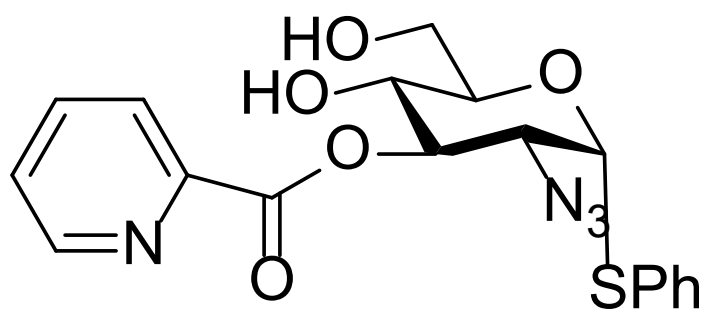

3

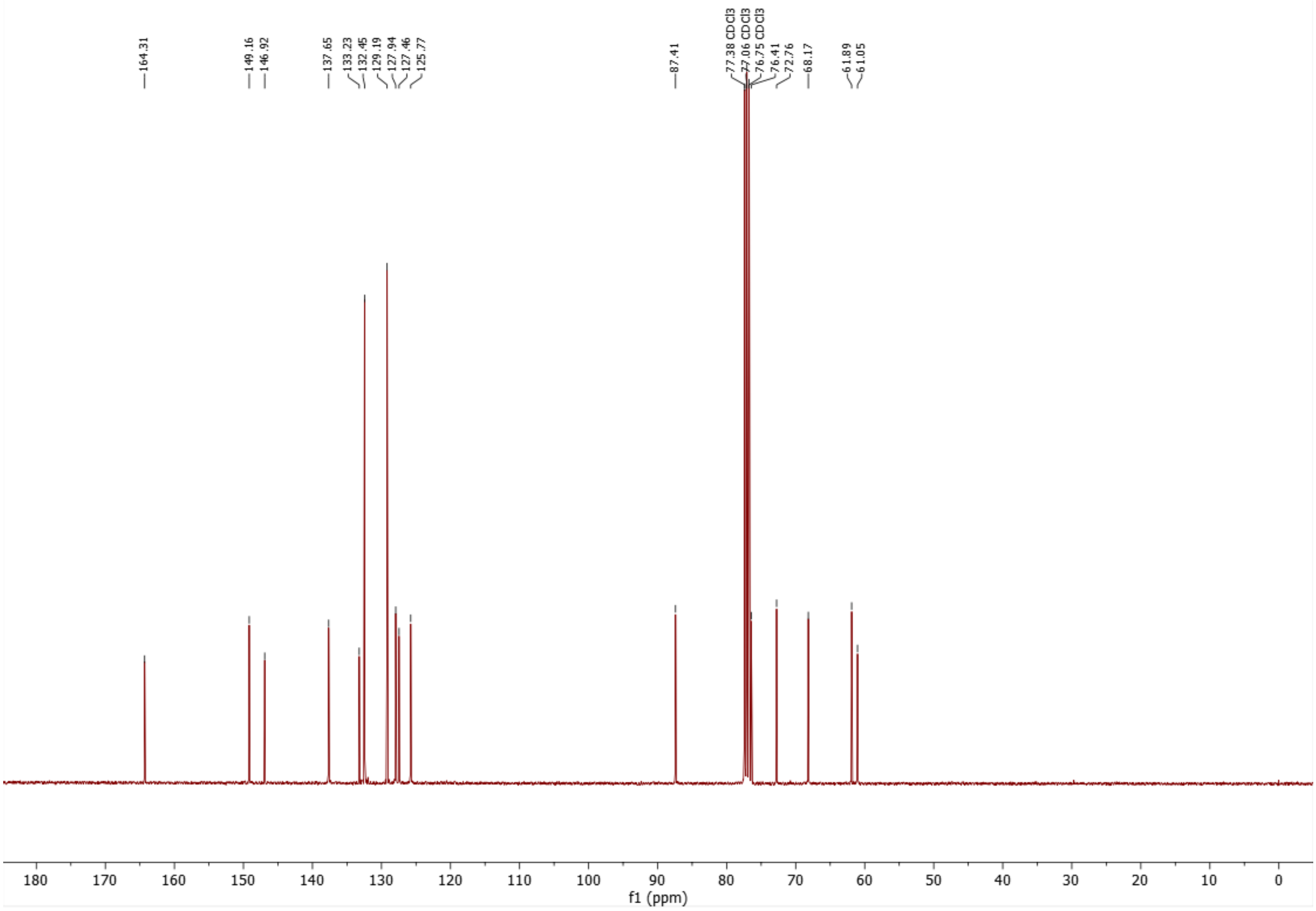

Figure S6: ^{13}C NMR (101 MHz, $CDCl_3$)

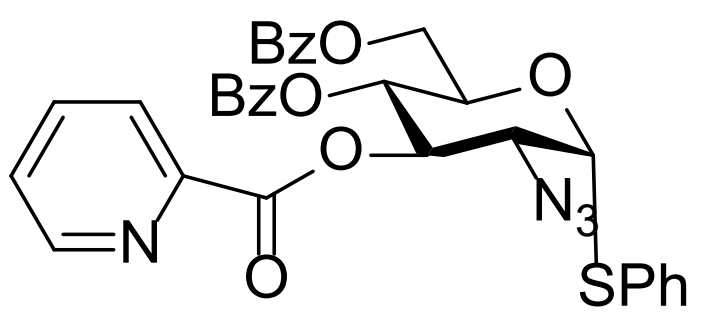

4

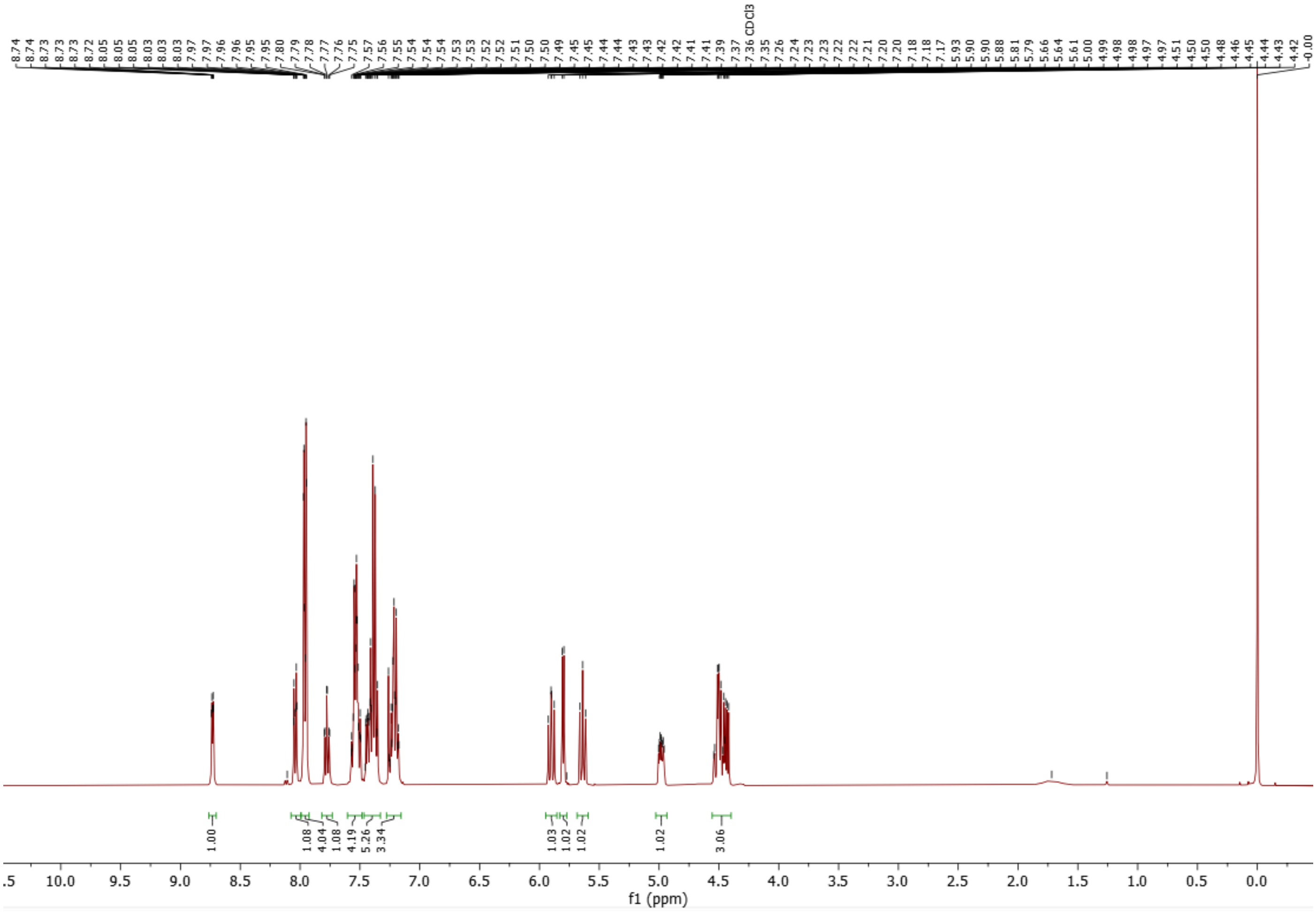

Figure S7: ^{1}H NMR (400 MHz, $CDCl_3$)

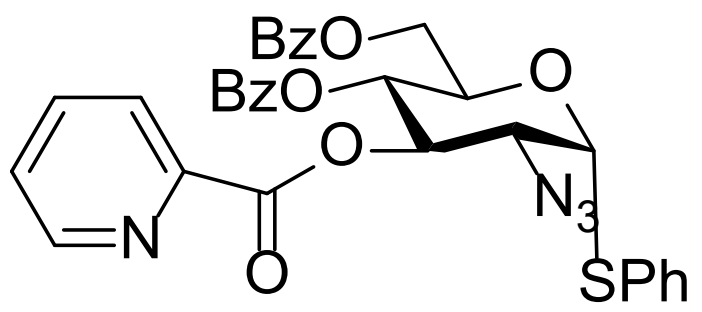

4

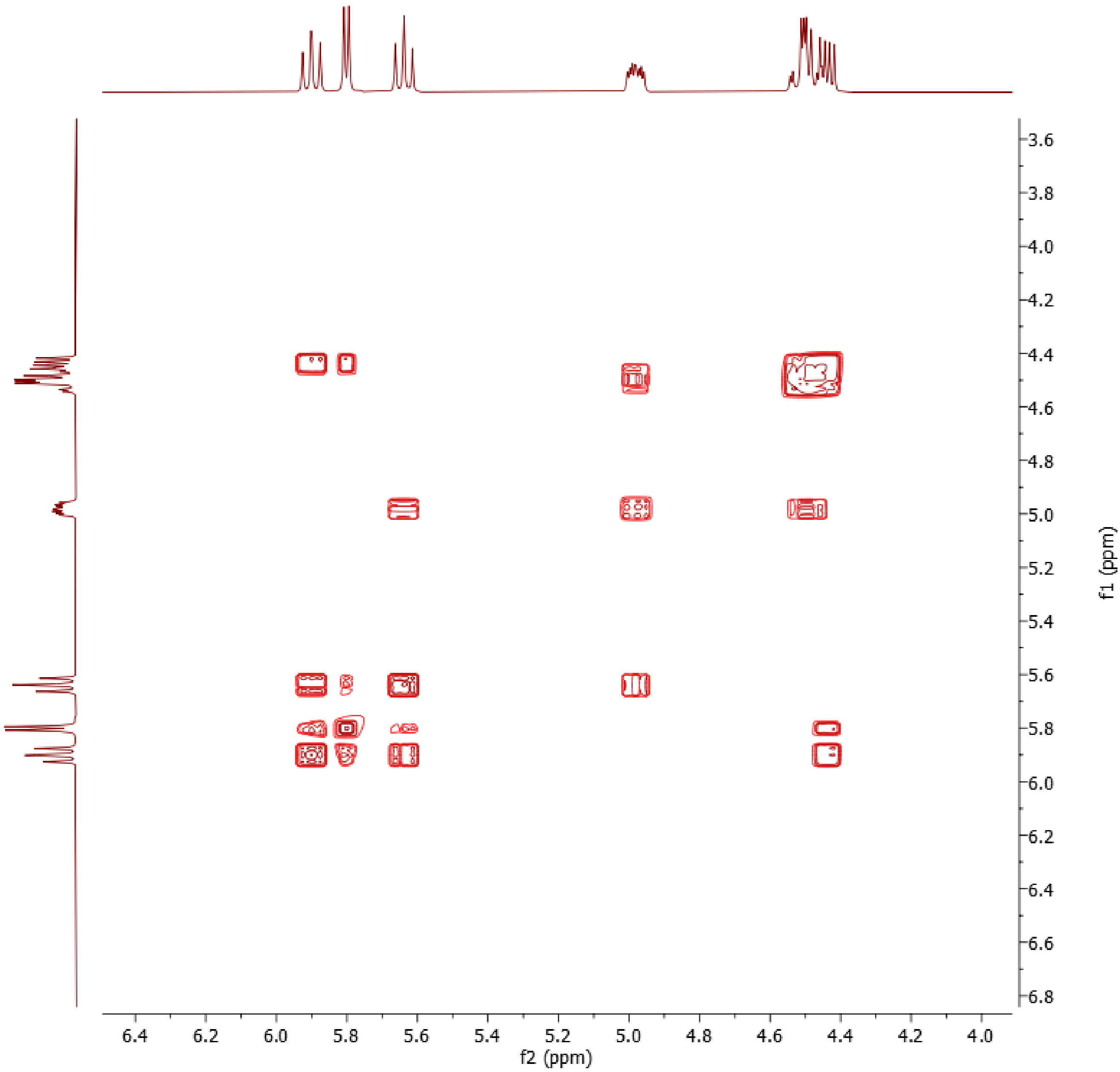

Figure S8:COSY NMR (400 MHz, $CDCl_3$)

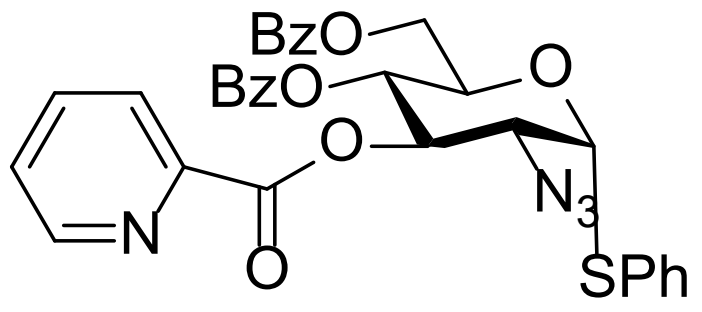

4

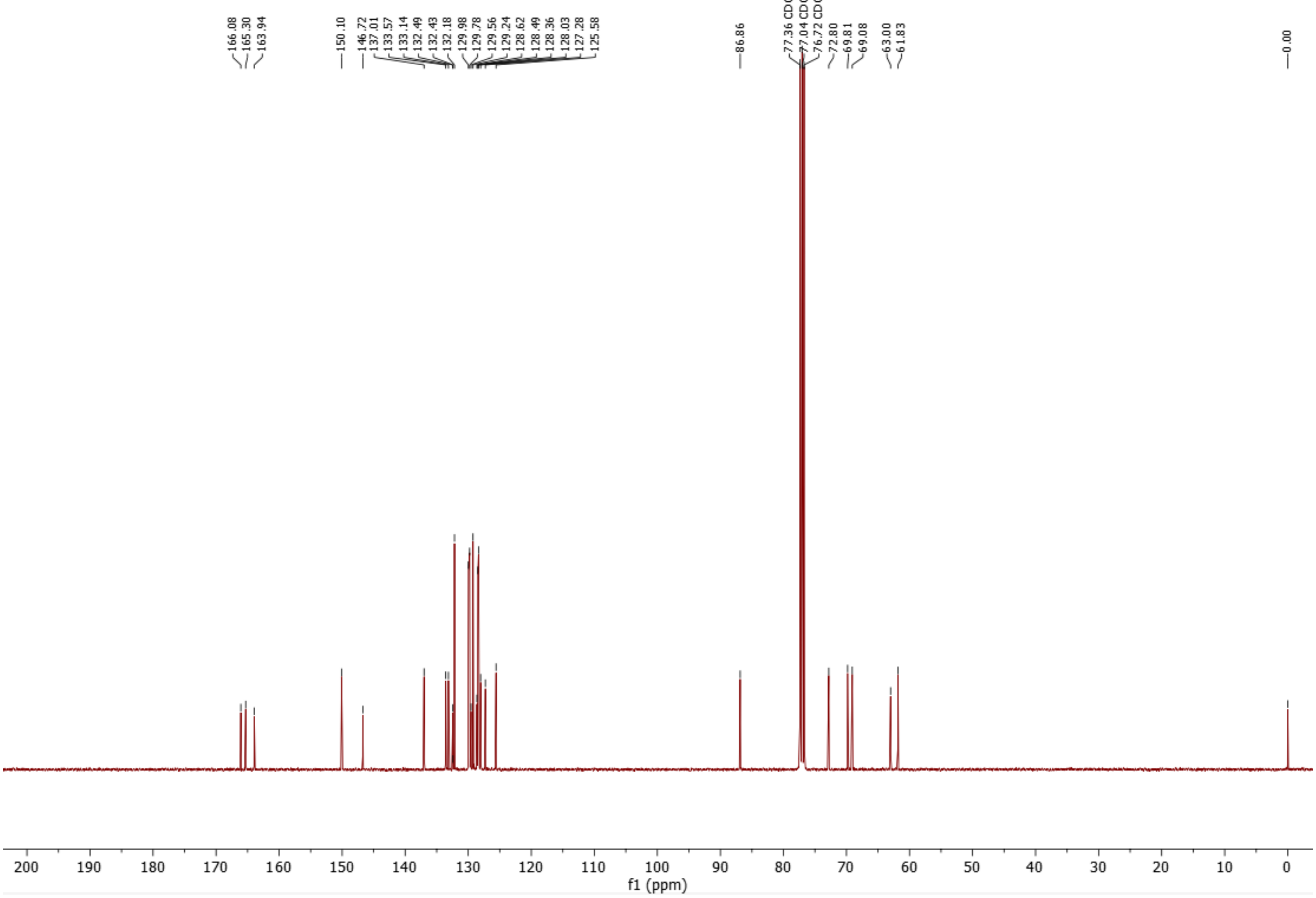

Figure S9: ^{13}C NMR (101 MHz, $CDCl_3$)

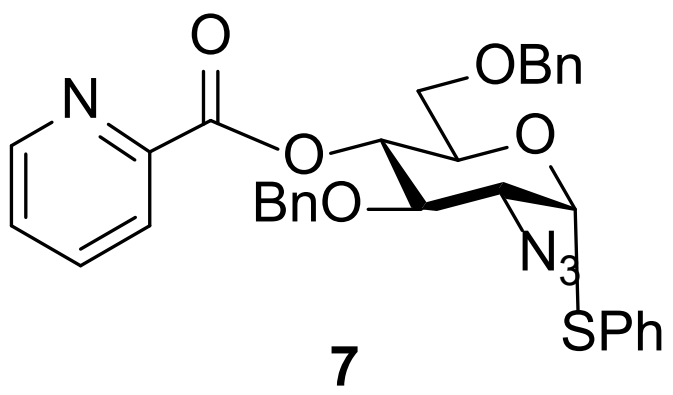

7

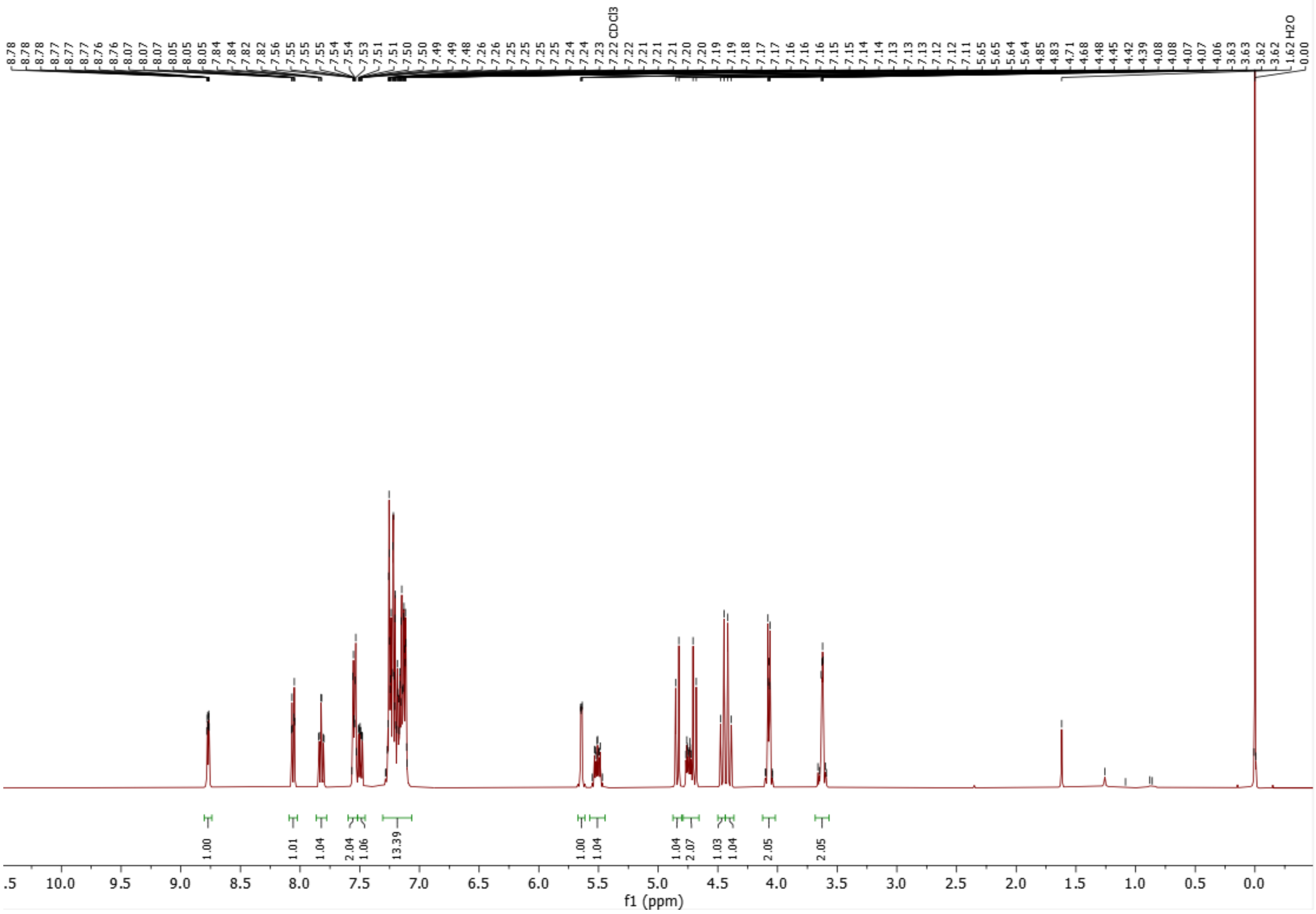

Figure S10: ^{1}H NMR (400 MHz, $CDCl_3$)

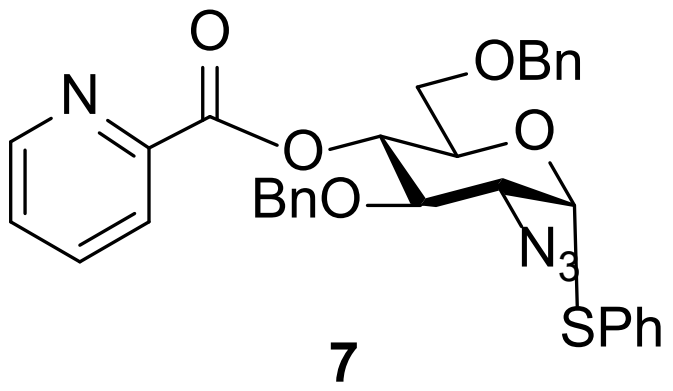

7

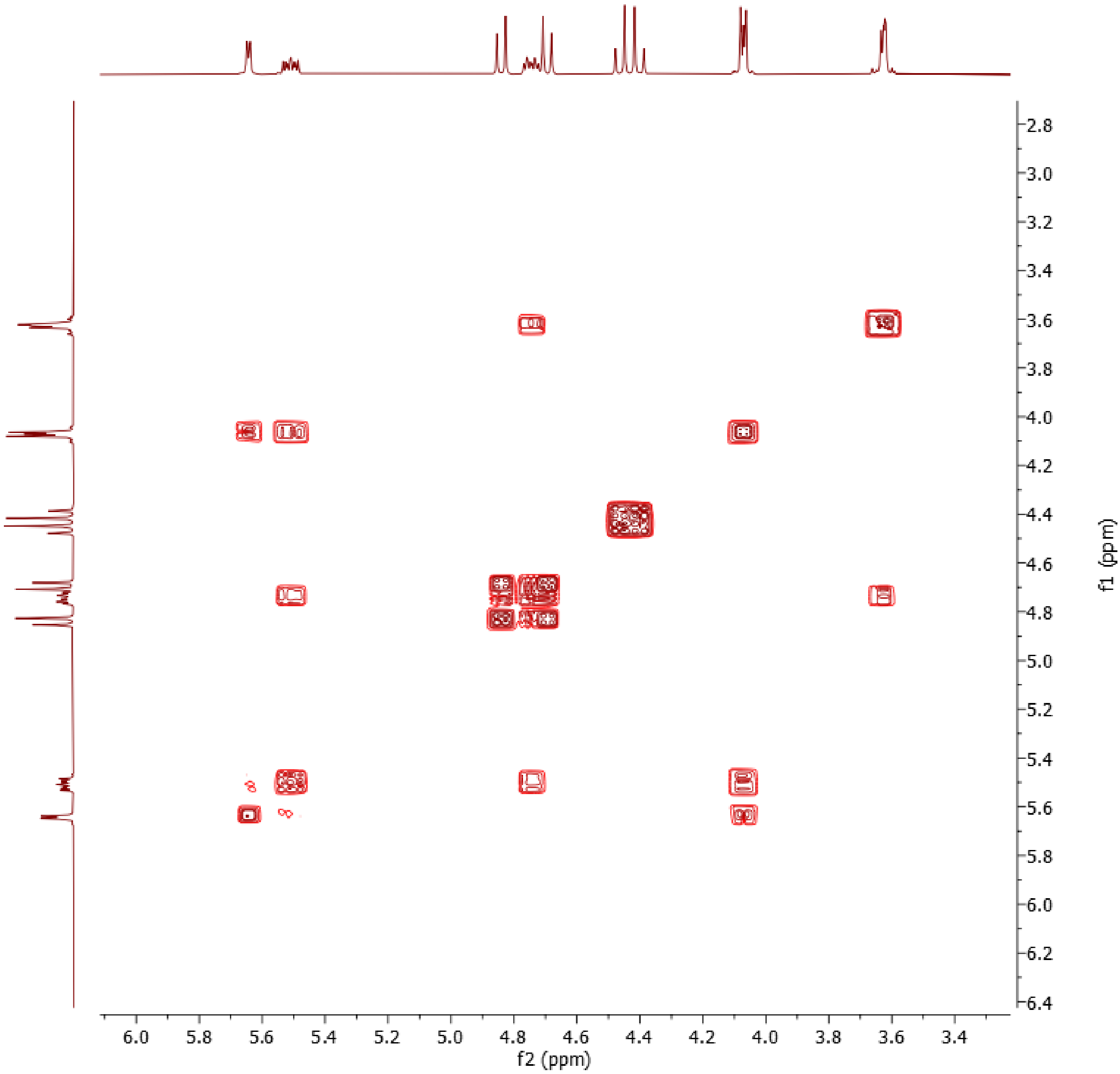

Figure S11: COSY NMR (400 MHz, $CDCl_3$)

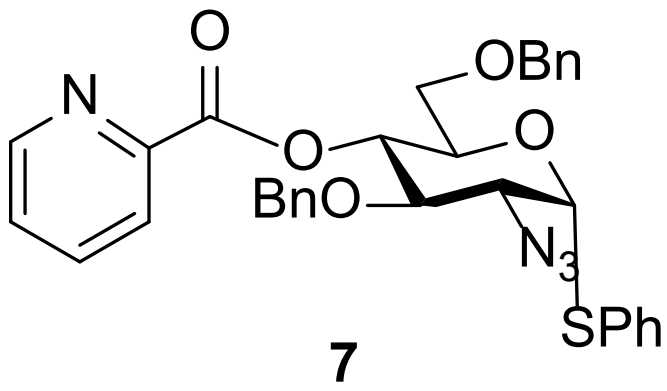

7

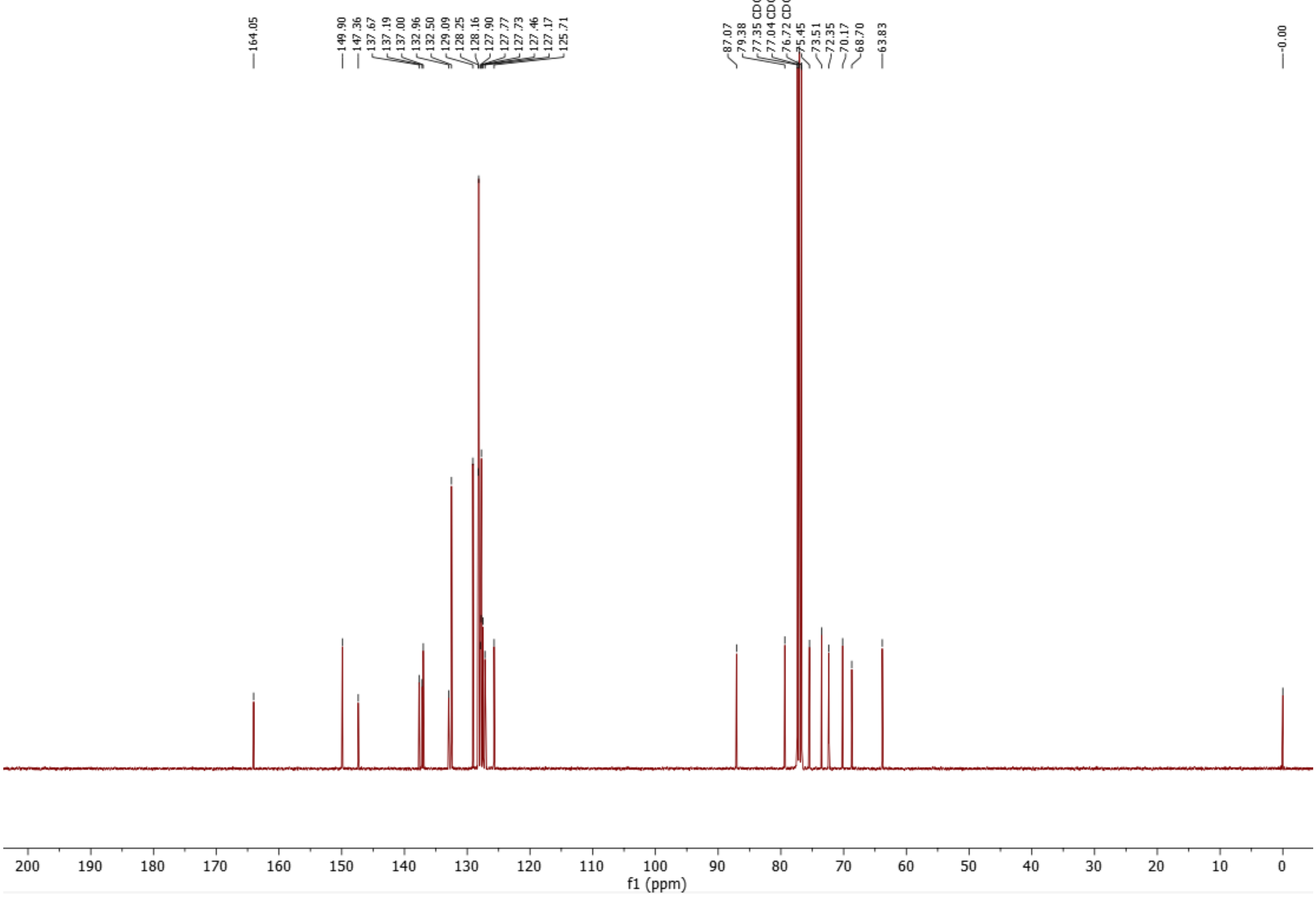

Figure S12: ^{13}C NMR (101 MHz, $CDCl_3$)

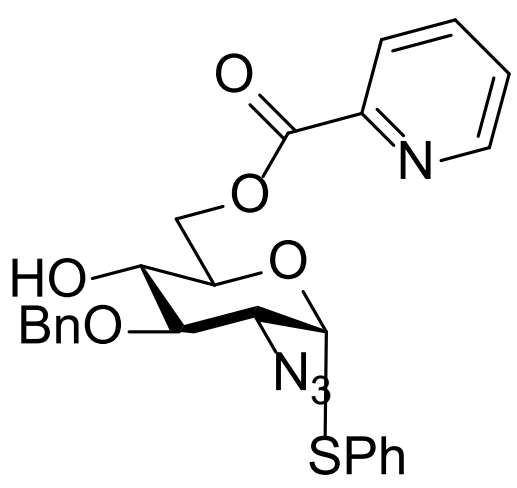

9

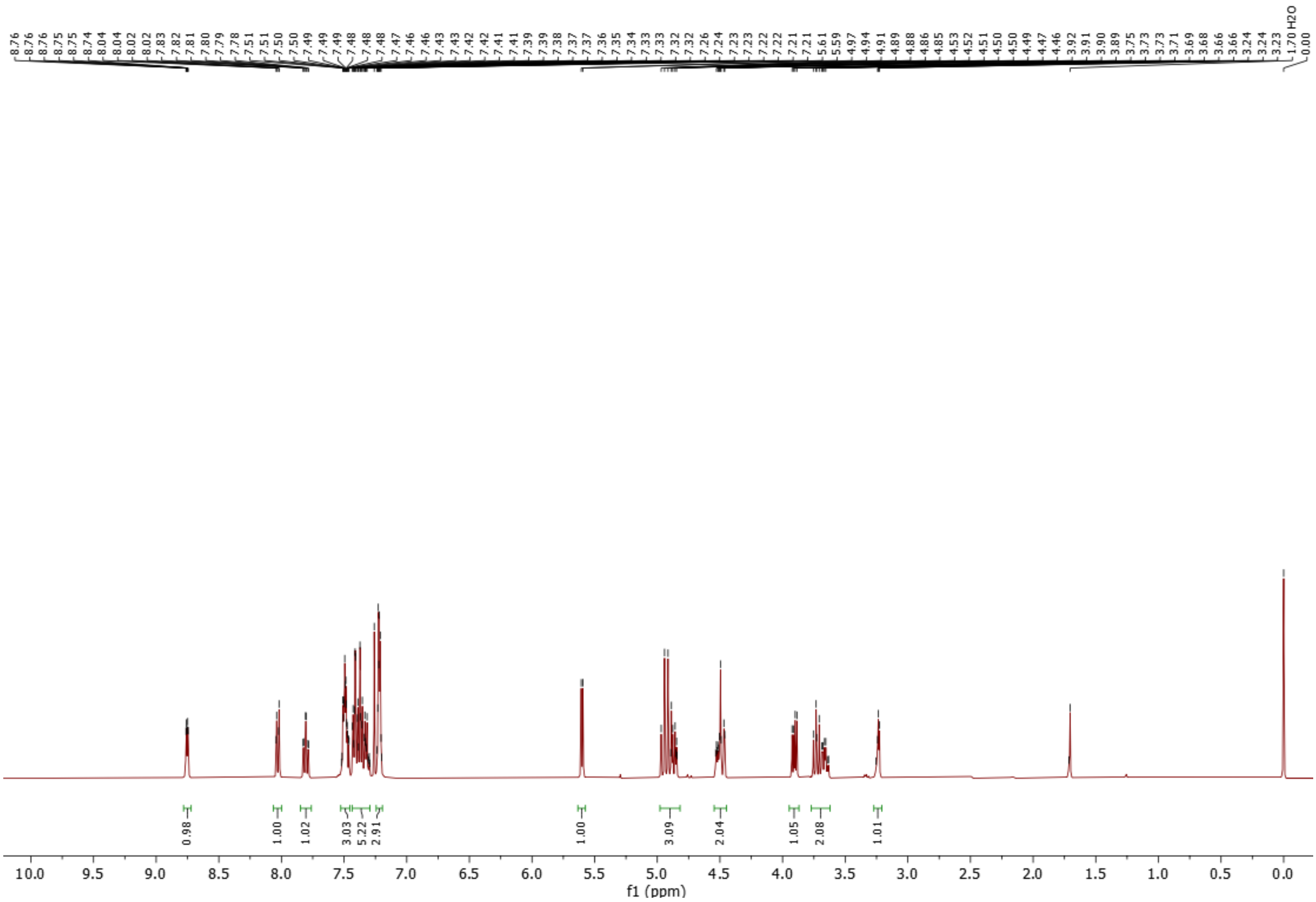

Figure S13: ^{1}H NMR (400 MHz, $CDCl_3$)

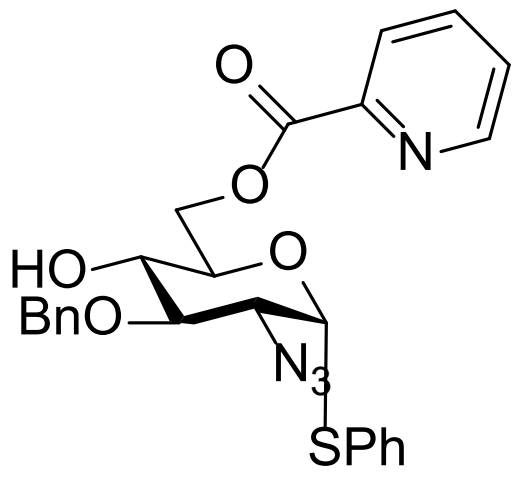

9

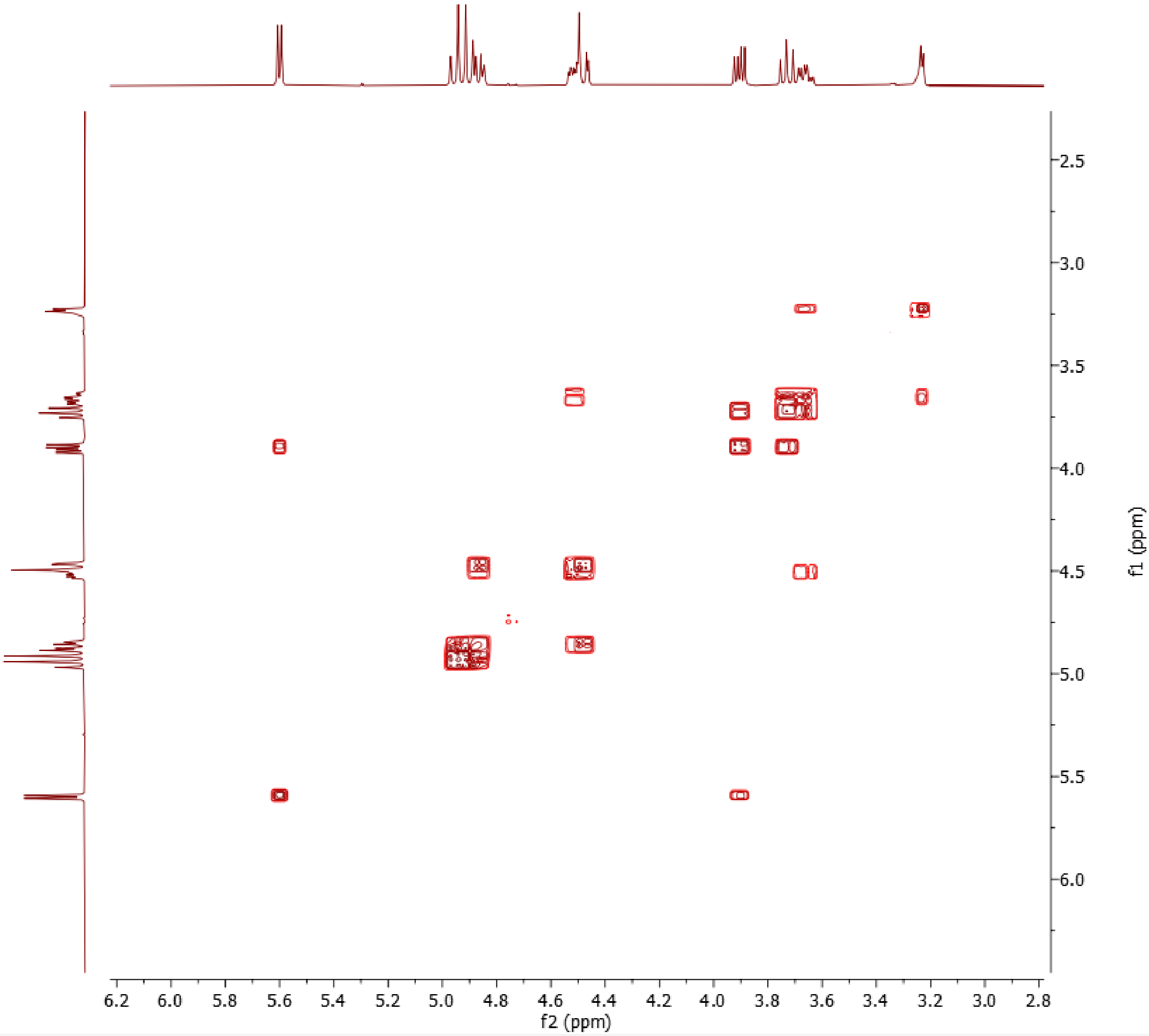

Figure S14: COSY NMR (400 MHz, $CDCl_3$)

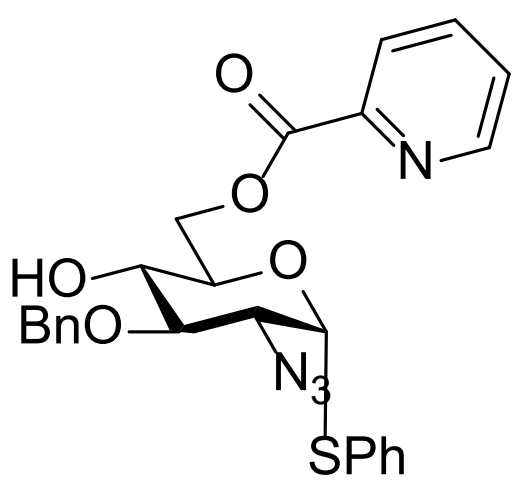

9

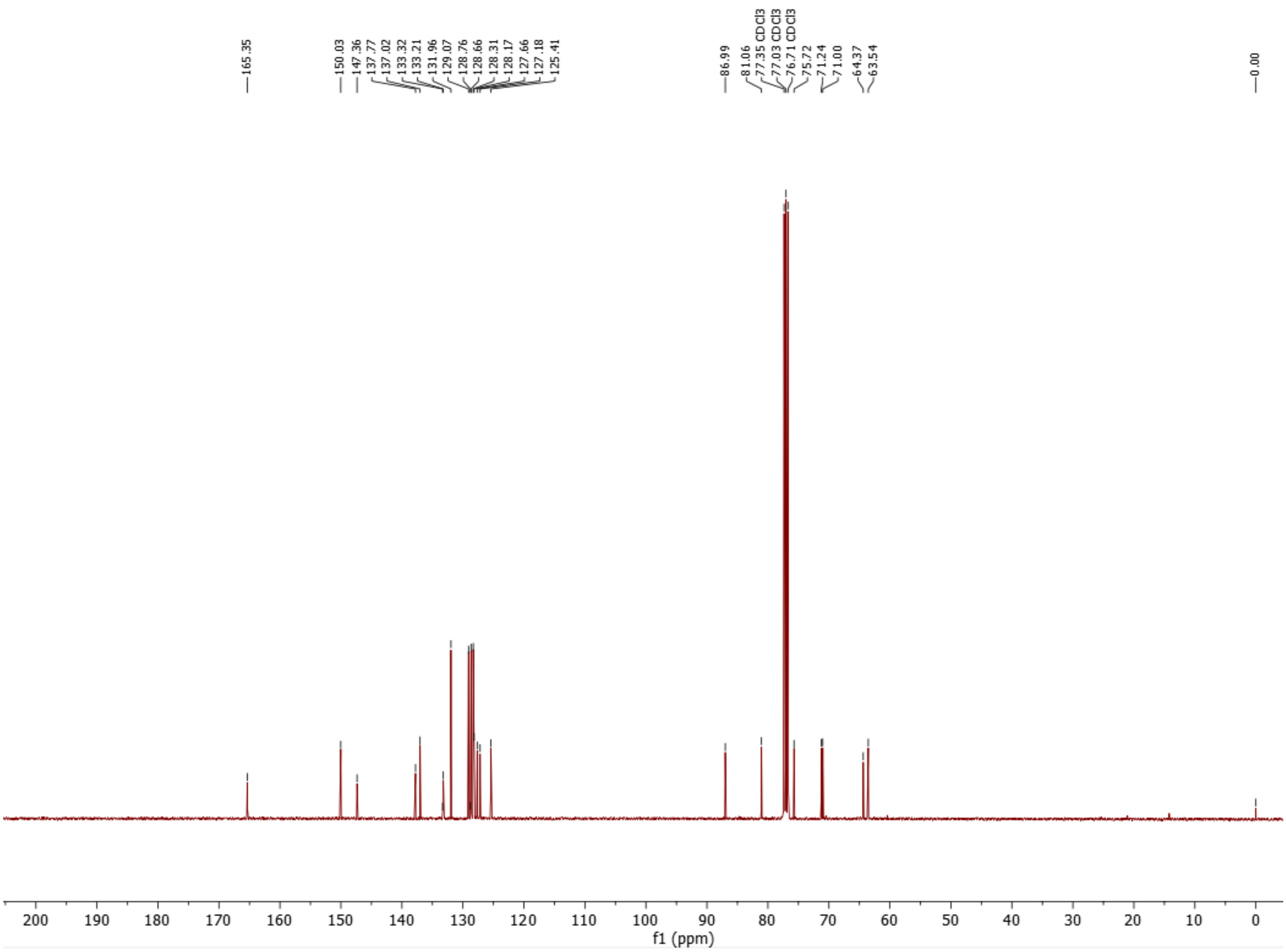

Figure S15: ^{13}C NMR (101 MHz, $CDCl_3$)

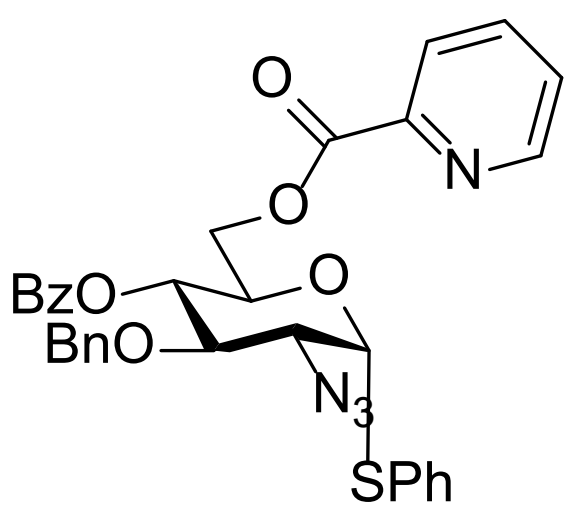

10

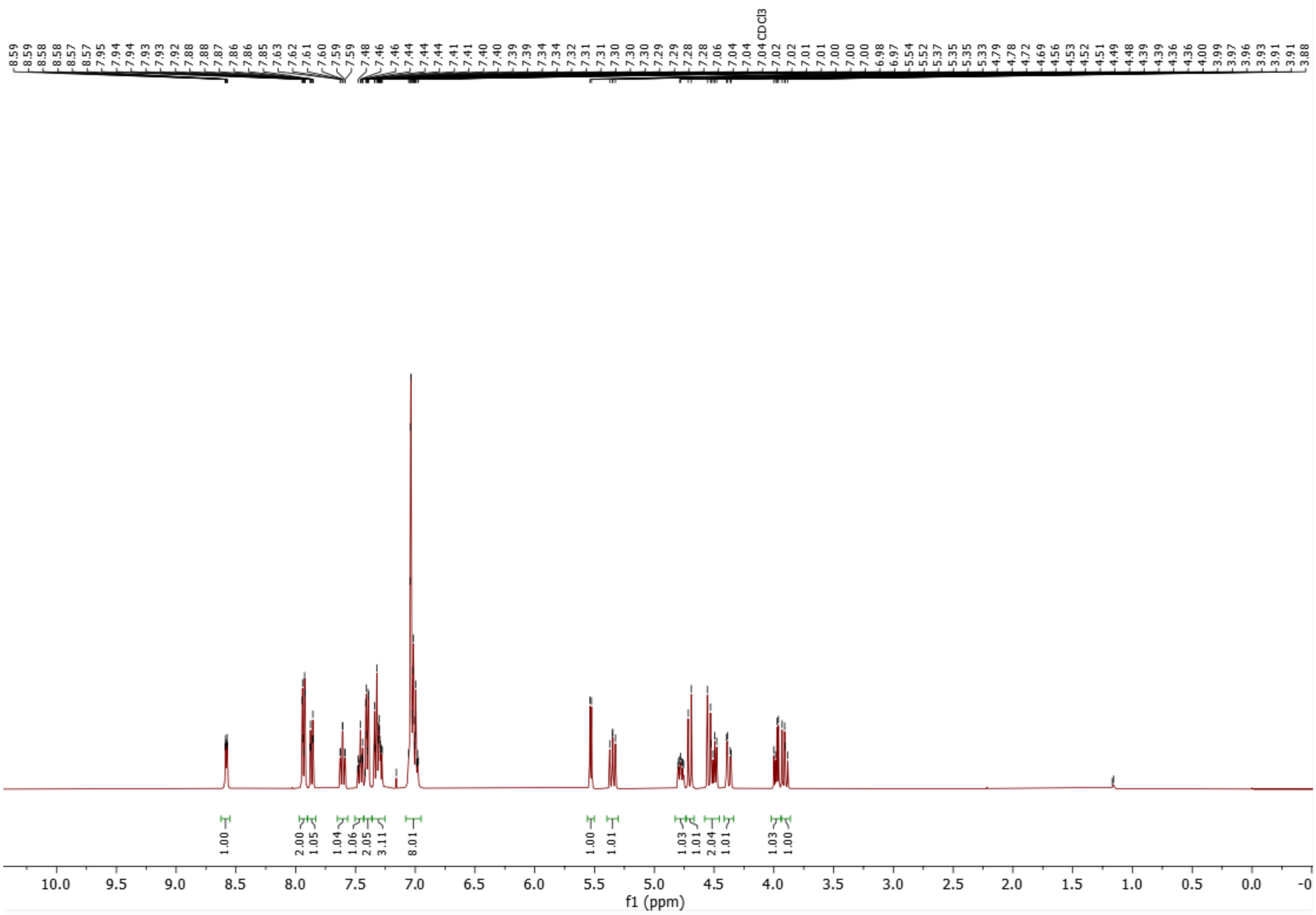

Figure S16: ^{1}H NMR (400 MHz, $CDCl_3$)

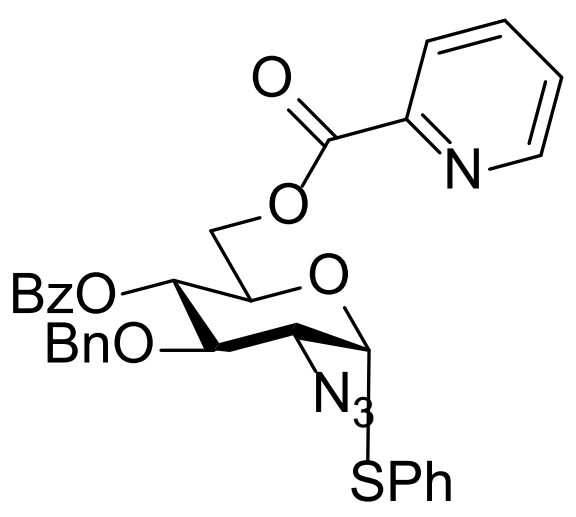

10

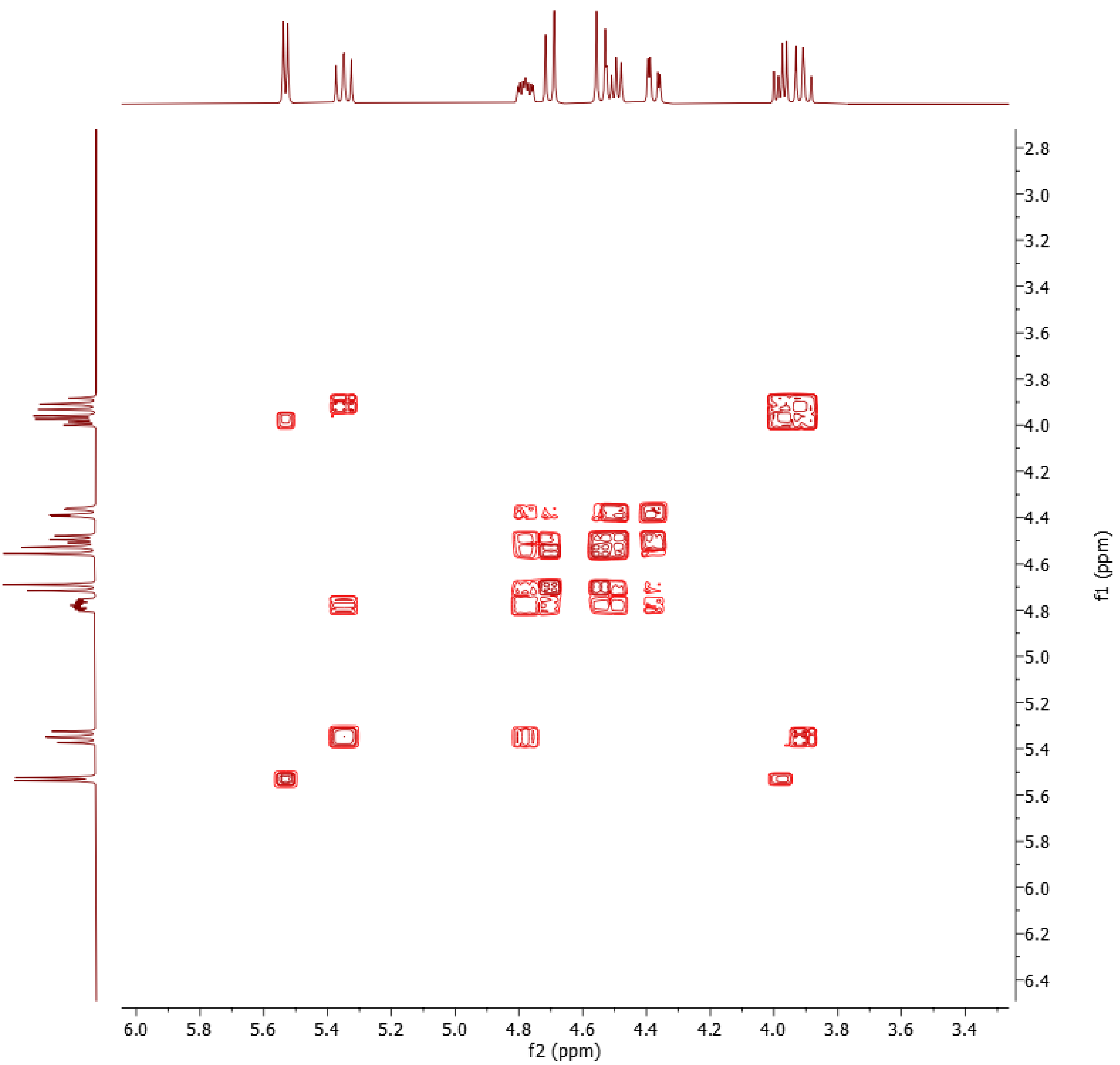

Figure S17: COSY NMR (400 MHz, $CDCl_3$)

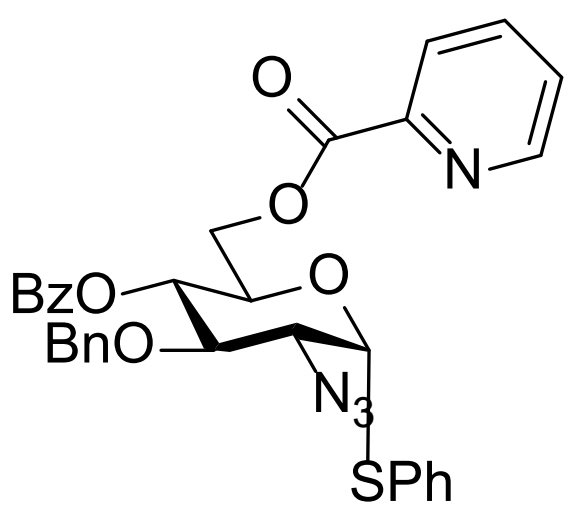

10

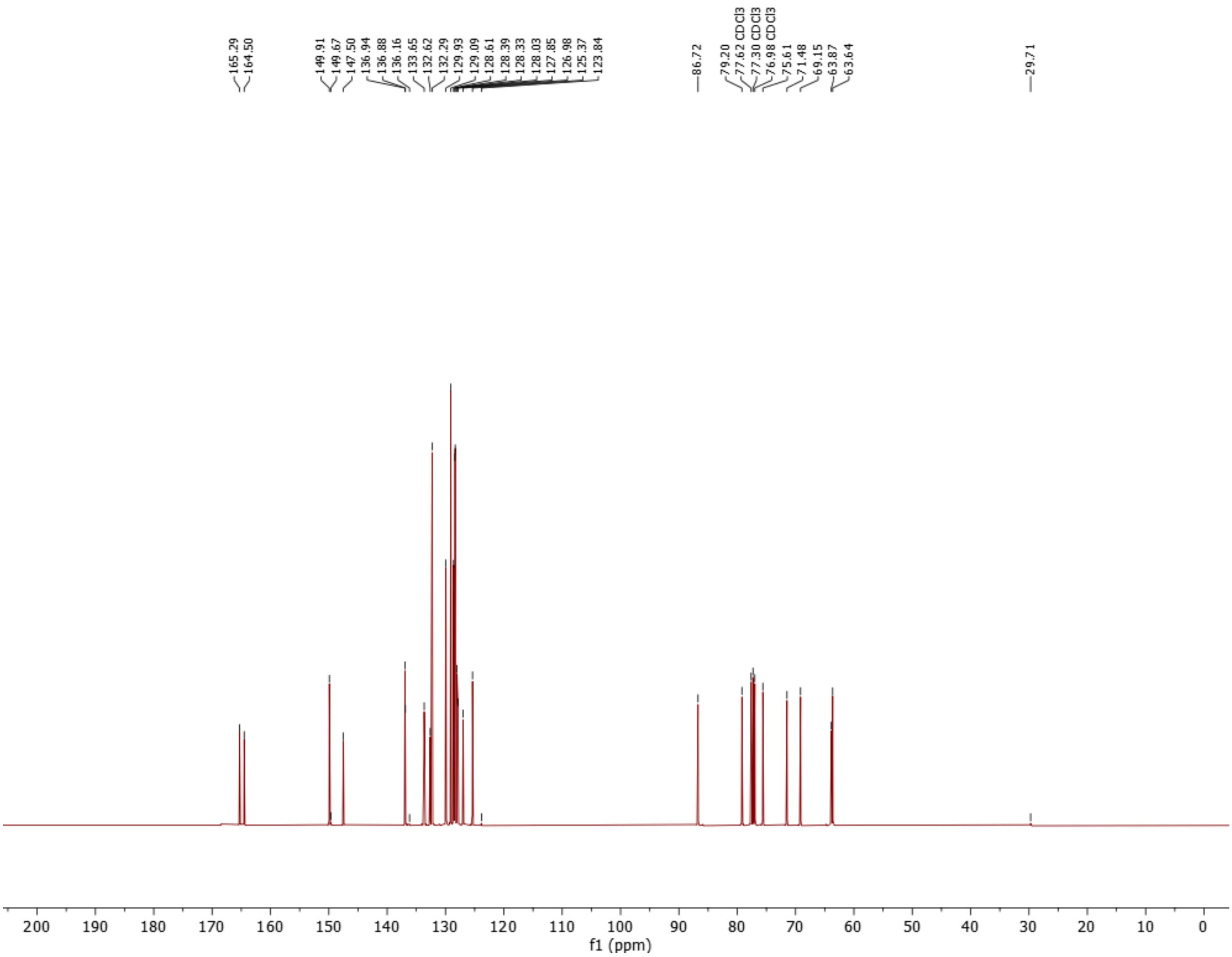

Figure S18: ^{13}C NMR (101 MHz, $CDCl_3$)

Spectra of New Disaccharides

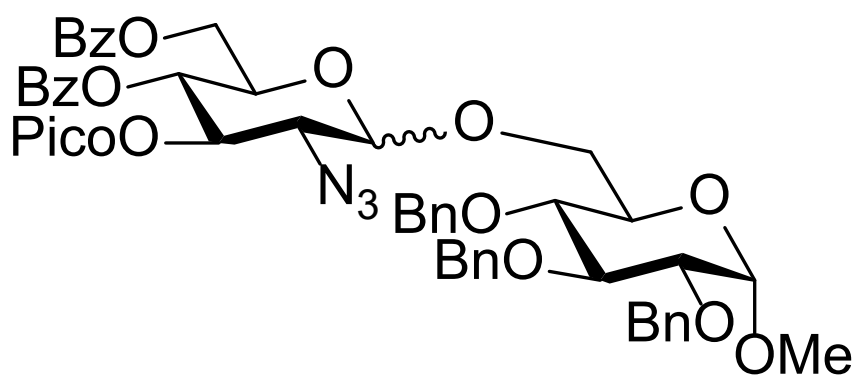

19

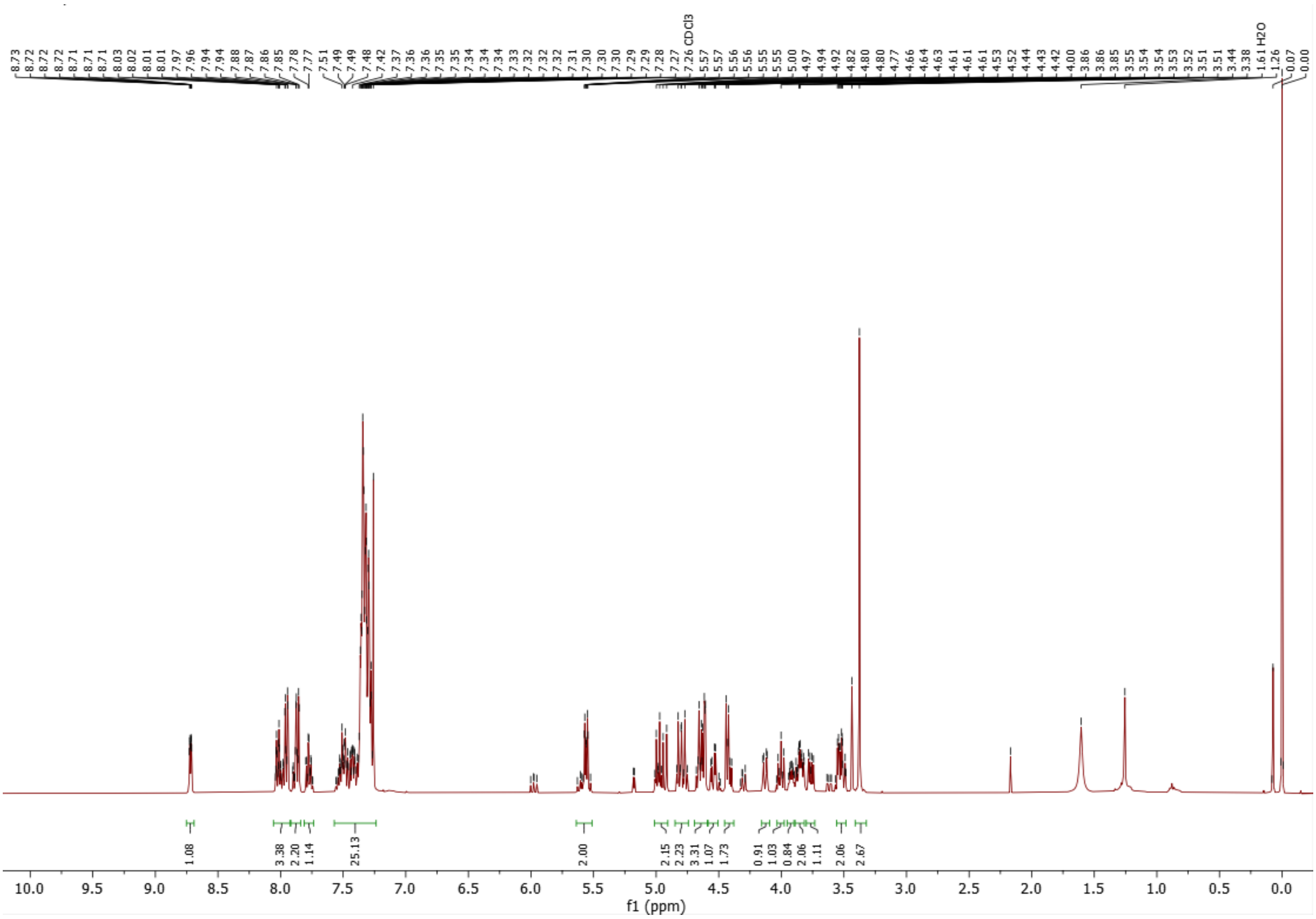

Figure S19: ^{1}H NMR (400 MHz, $CDCl_3$)

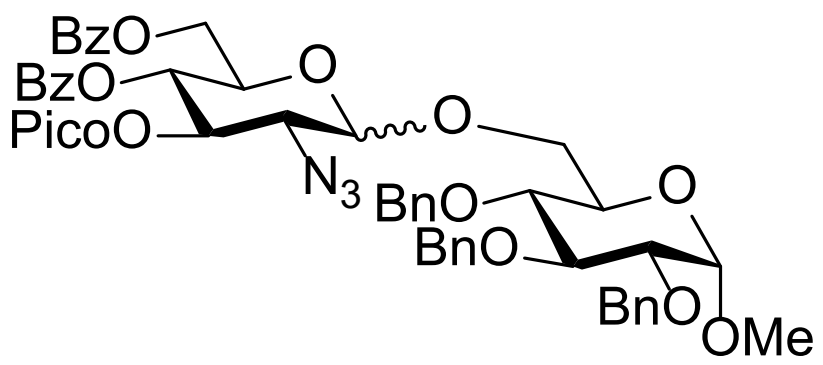

19

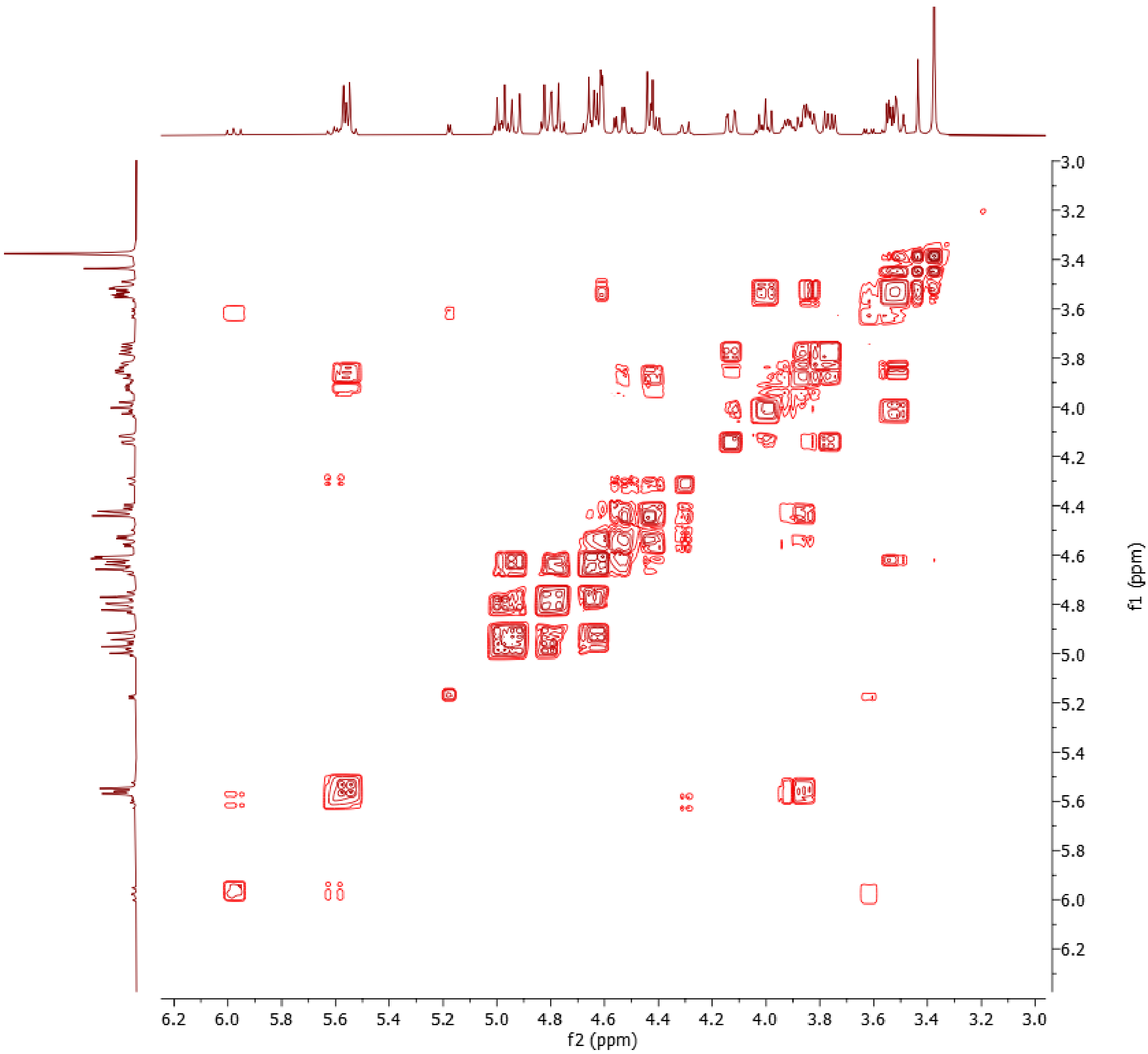

Figure S20: COSY NMR (400 MHz, $CDCl_3$)

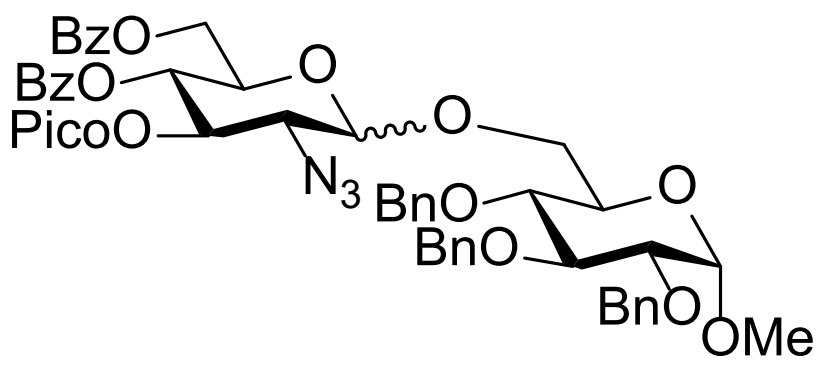

19

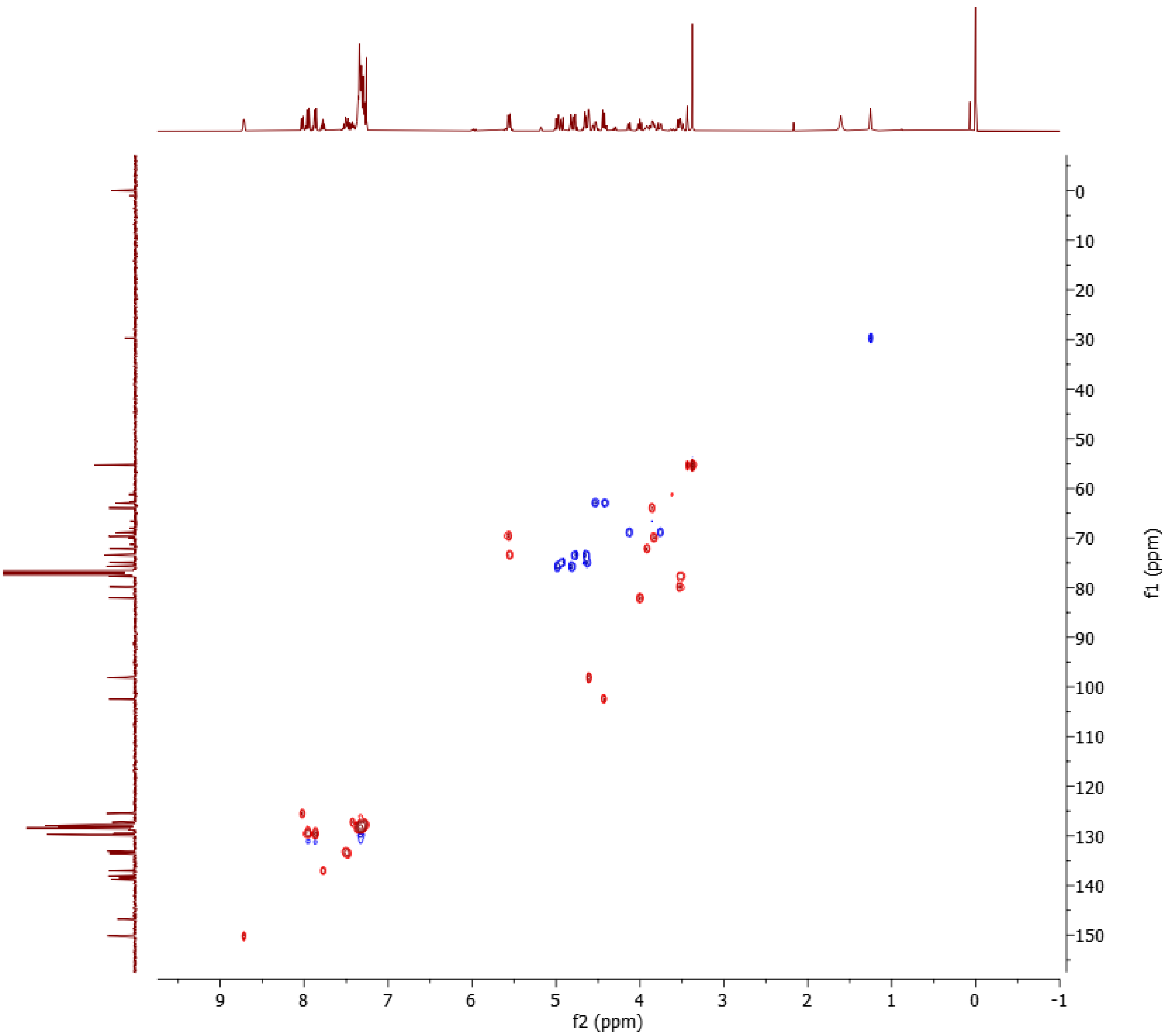

Figure S21: ^{1}H NMR and $^{13}C(^{1}H)$ NMR HSQC Spectrum (400 MHz, 101 MHz, $CDCl_3$)

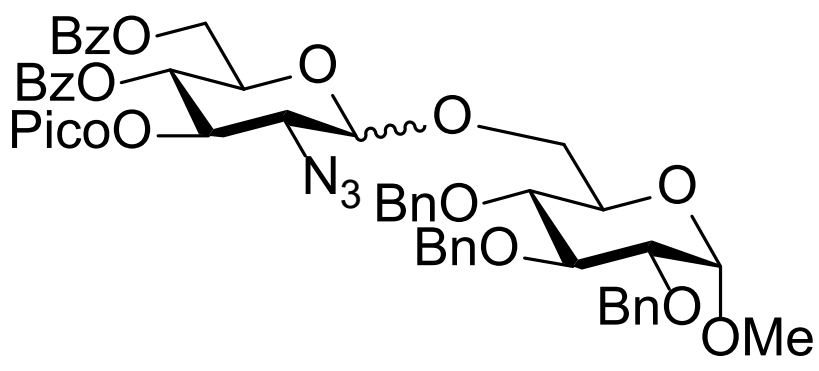

19

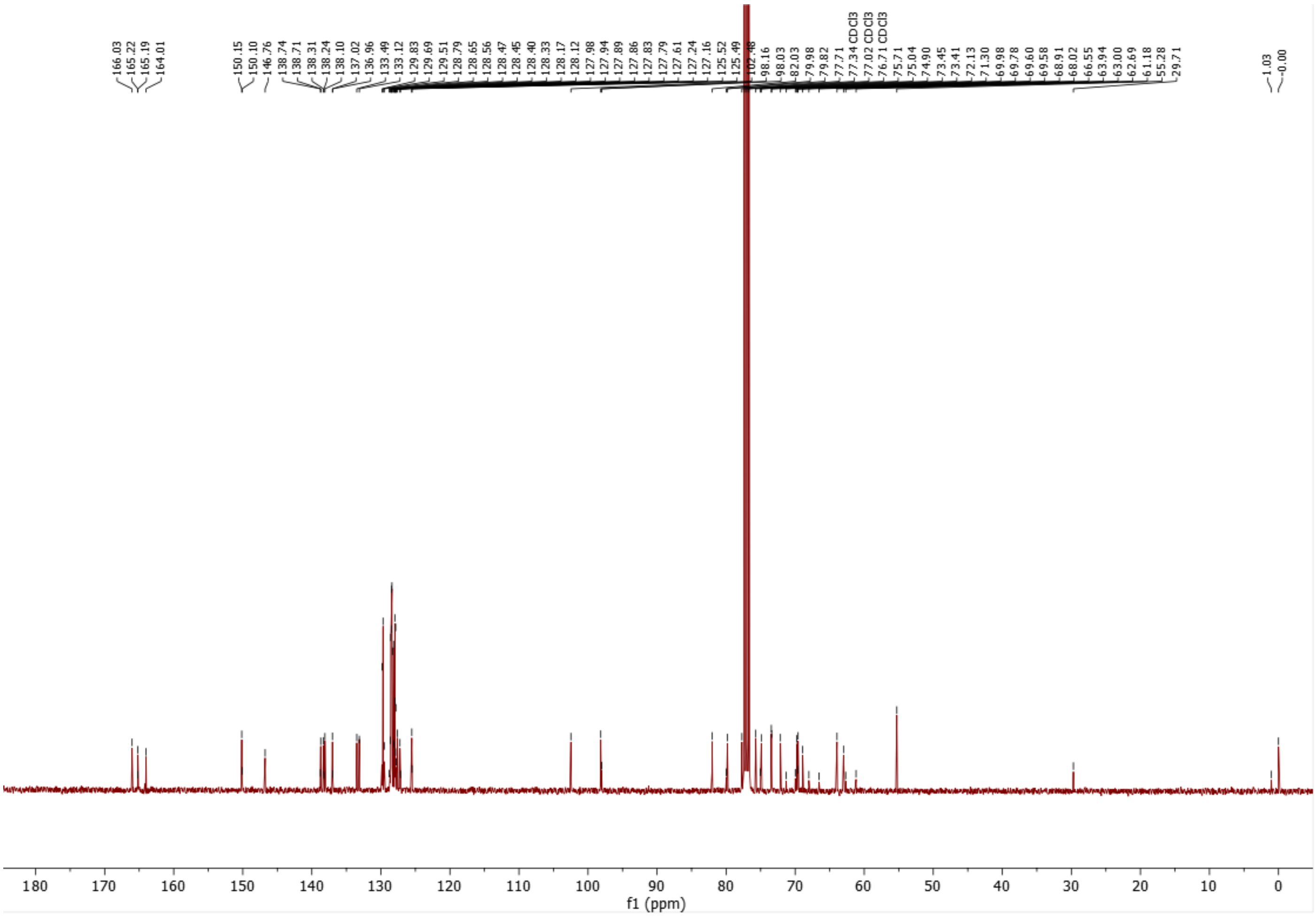

Figure S22: ^{13}C NMR (101 MHz, $CDCl_3$)

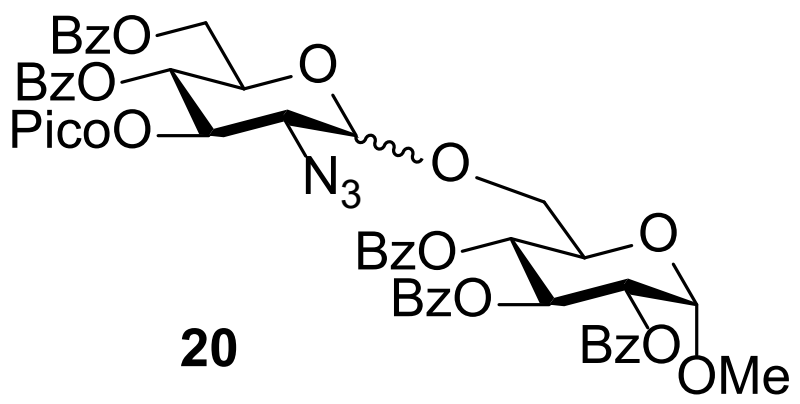

20

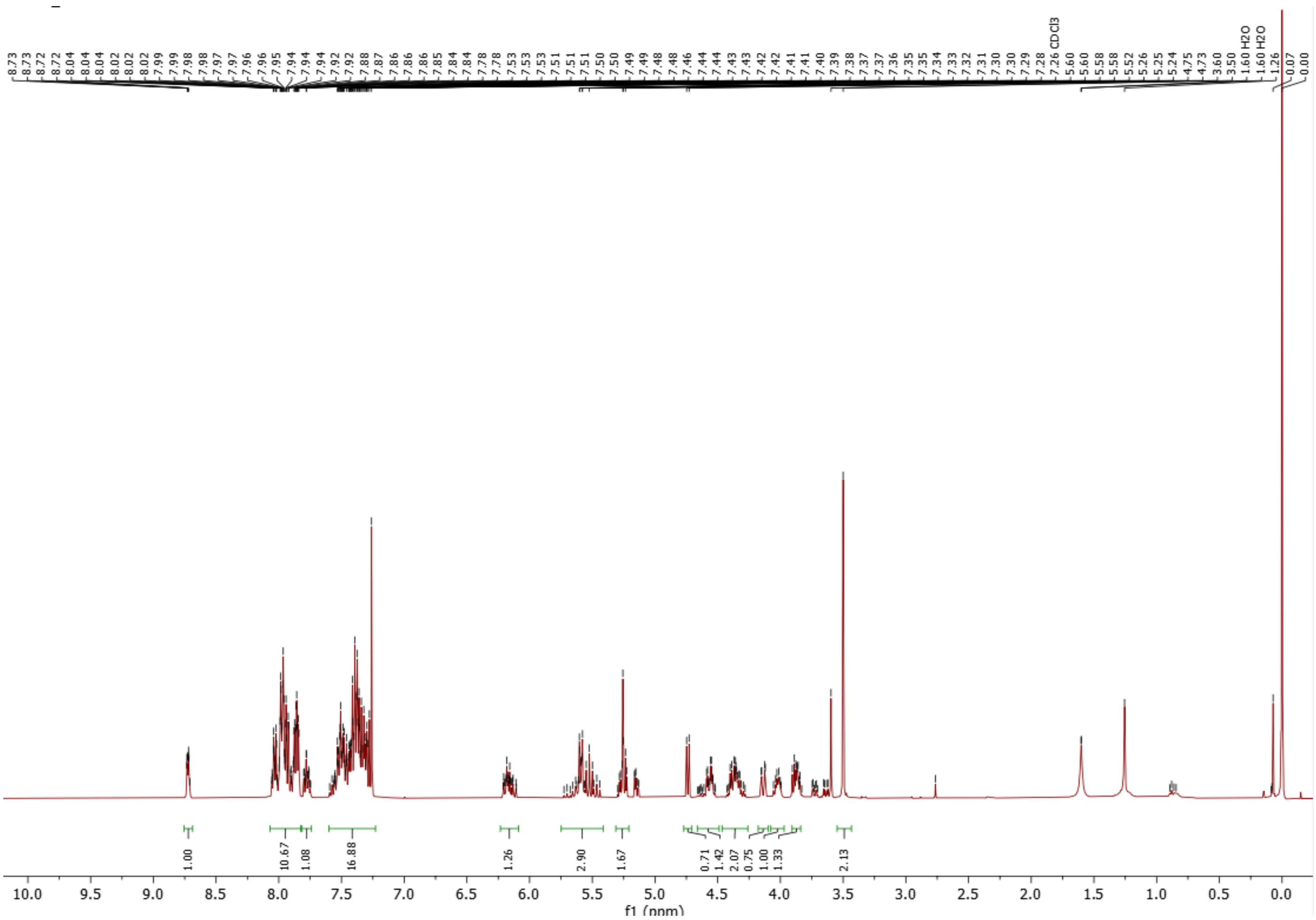

Figure S23: ^{1}H NMR (400 MHz, $CDCl_3$)

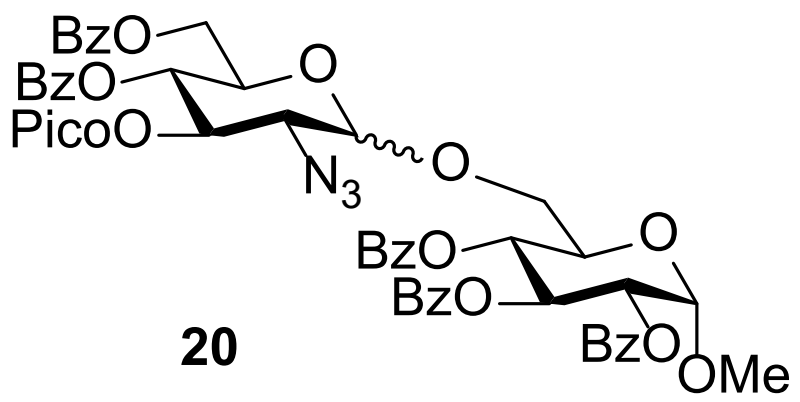

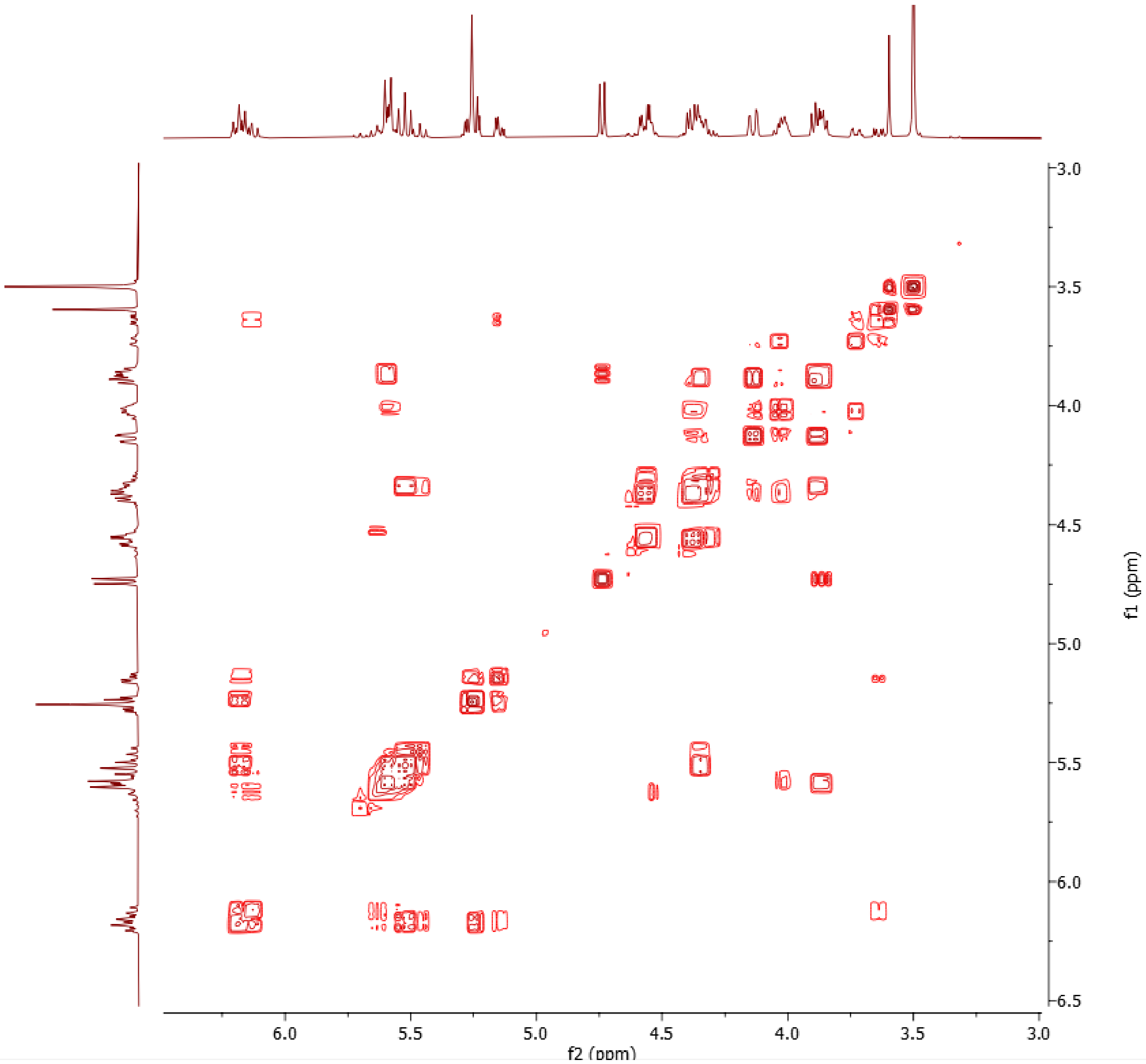

Figure S24: COSY NMR (400 MHz, $CDCl_3$)

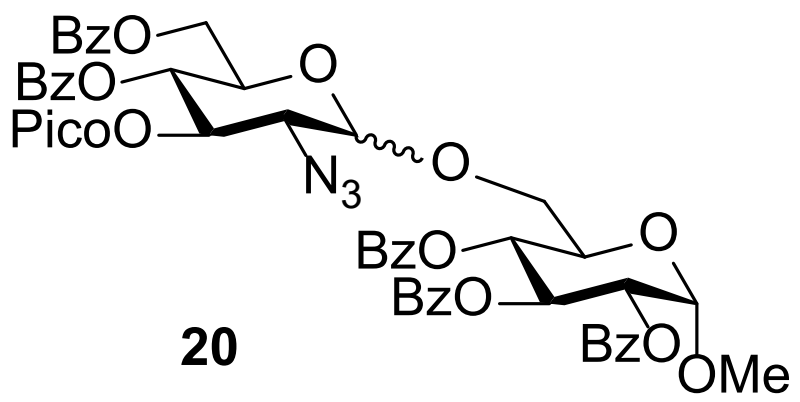

20

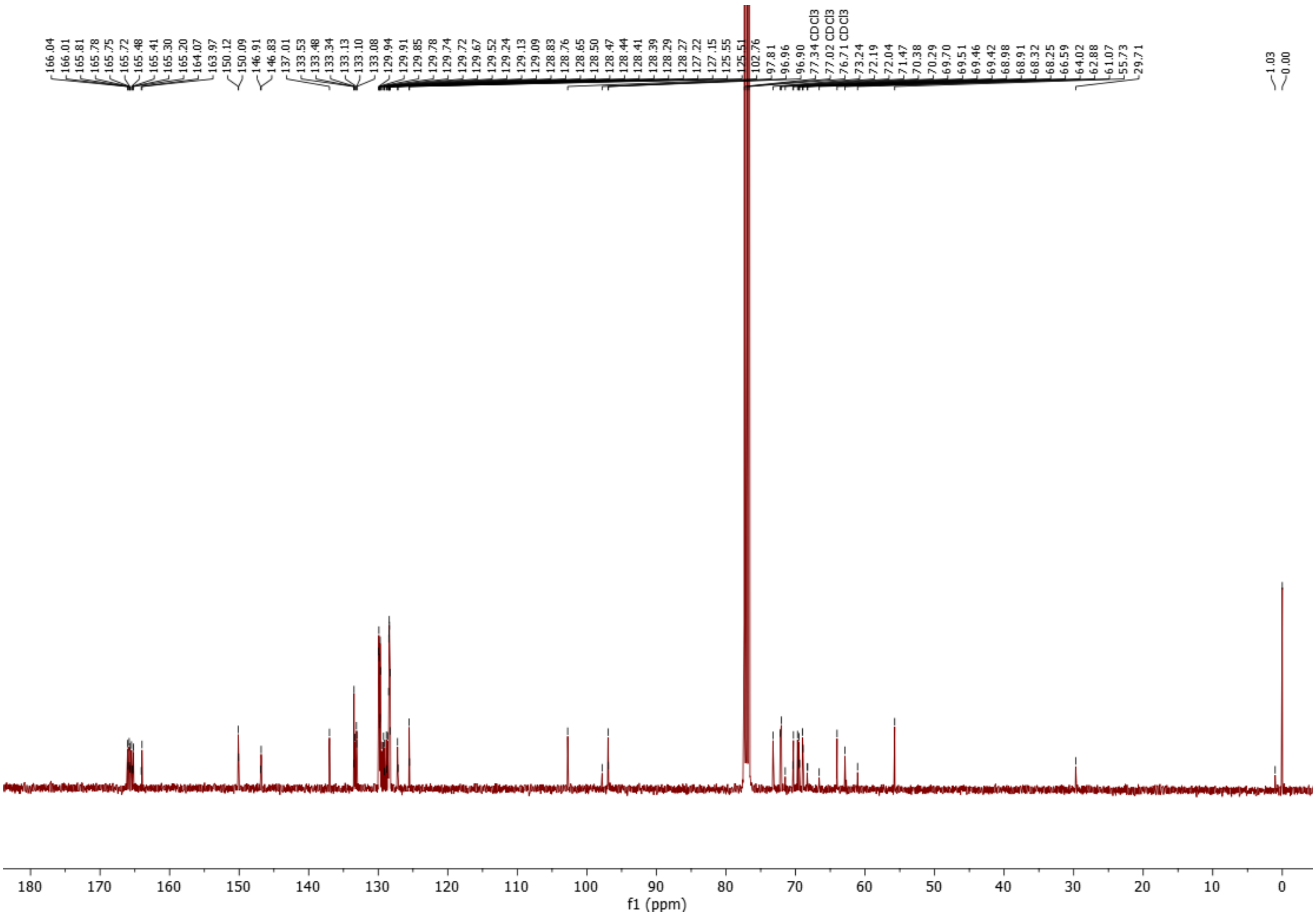

Figure S25: ^{13}C NMR (101 MHz, $CDCl_3$)

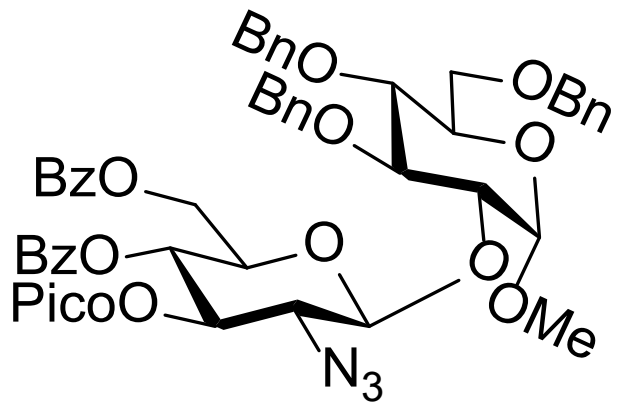

21

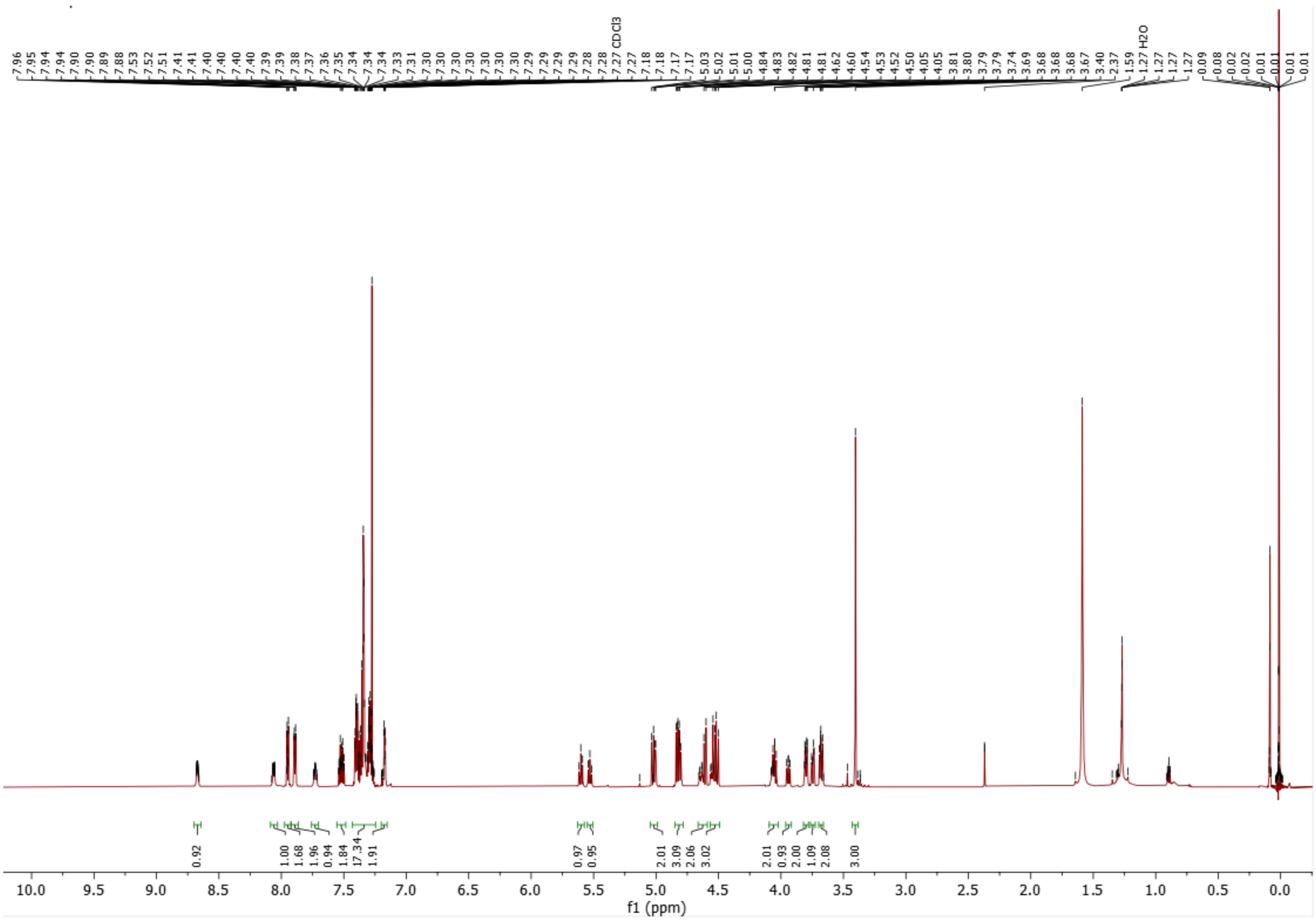

Figure S26: ^{1}H NMR (700 MHz, $CDCl_3$)

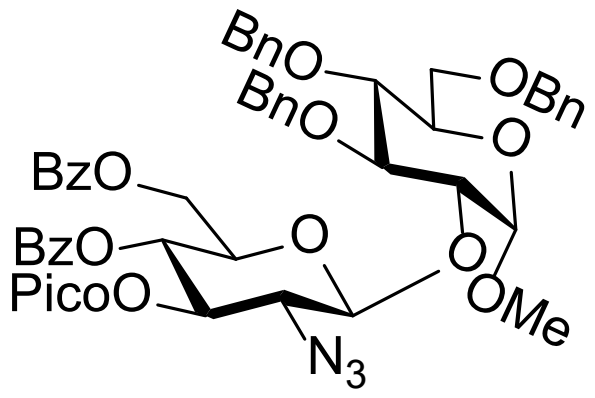

21

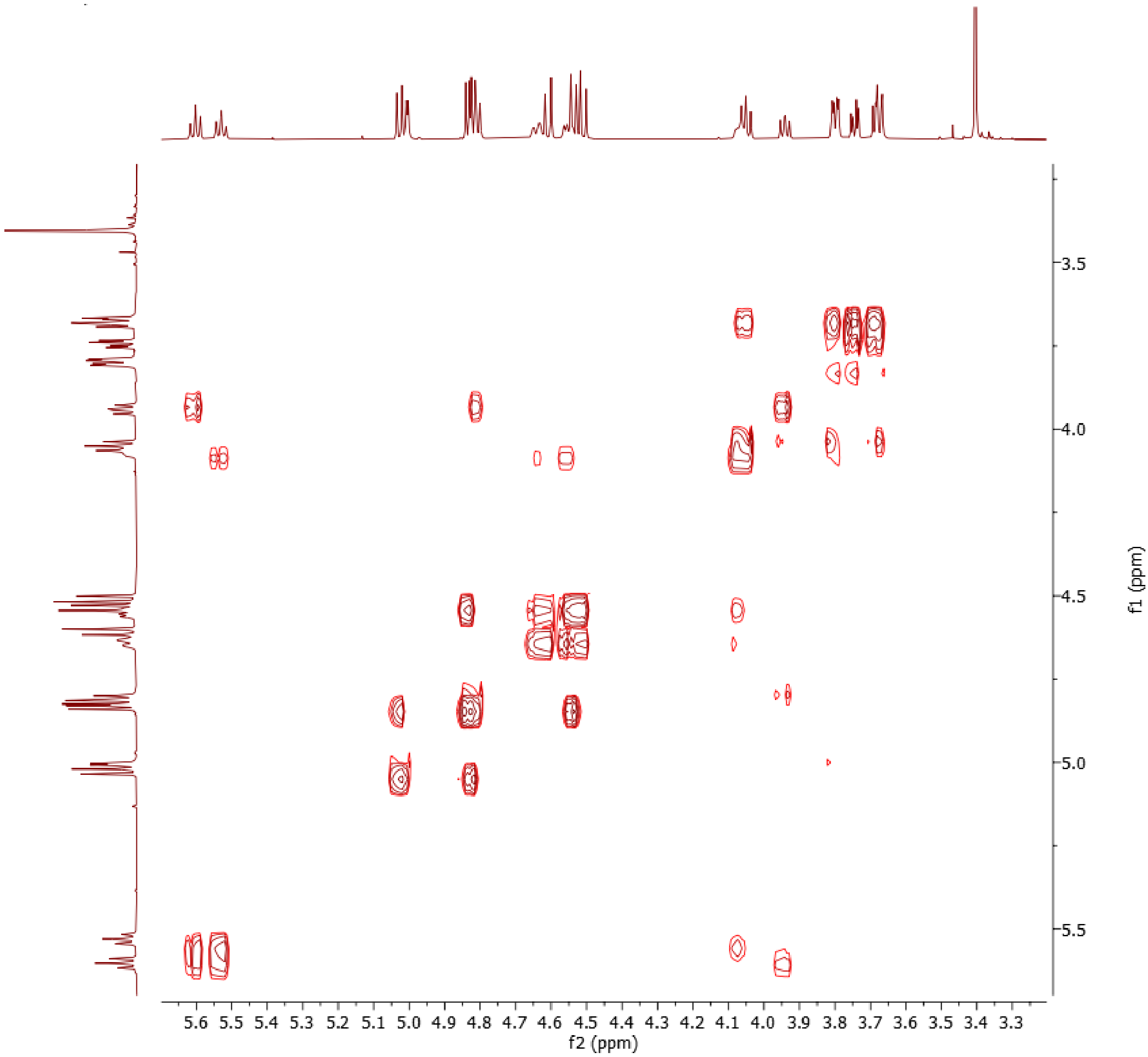

Figure S27: COSY NMR (700 MHz, $CDCl_3$)

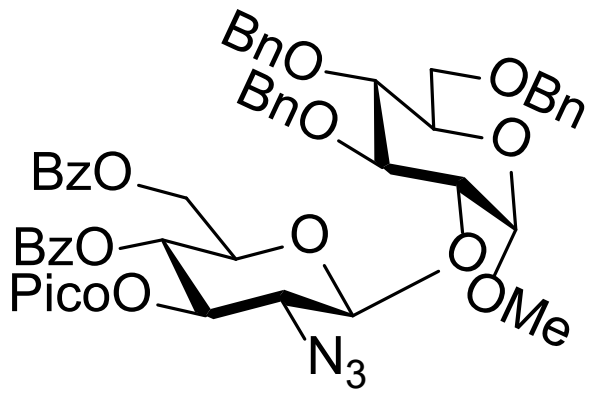

21

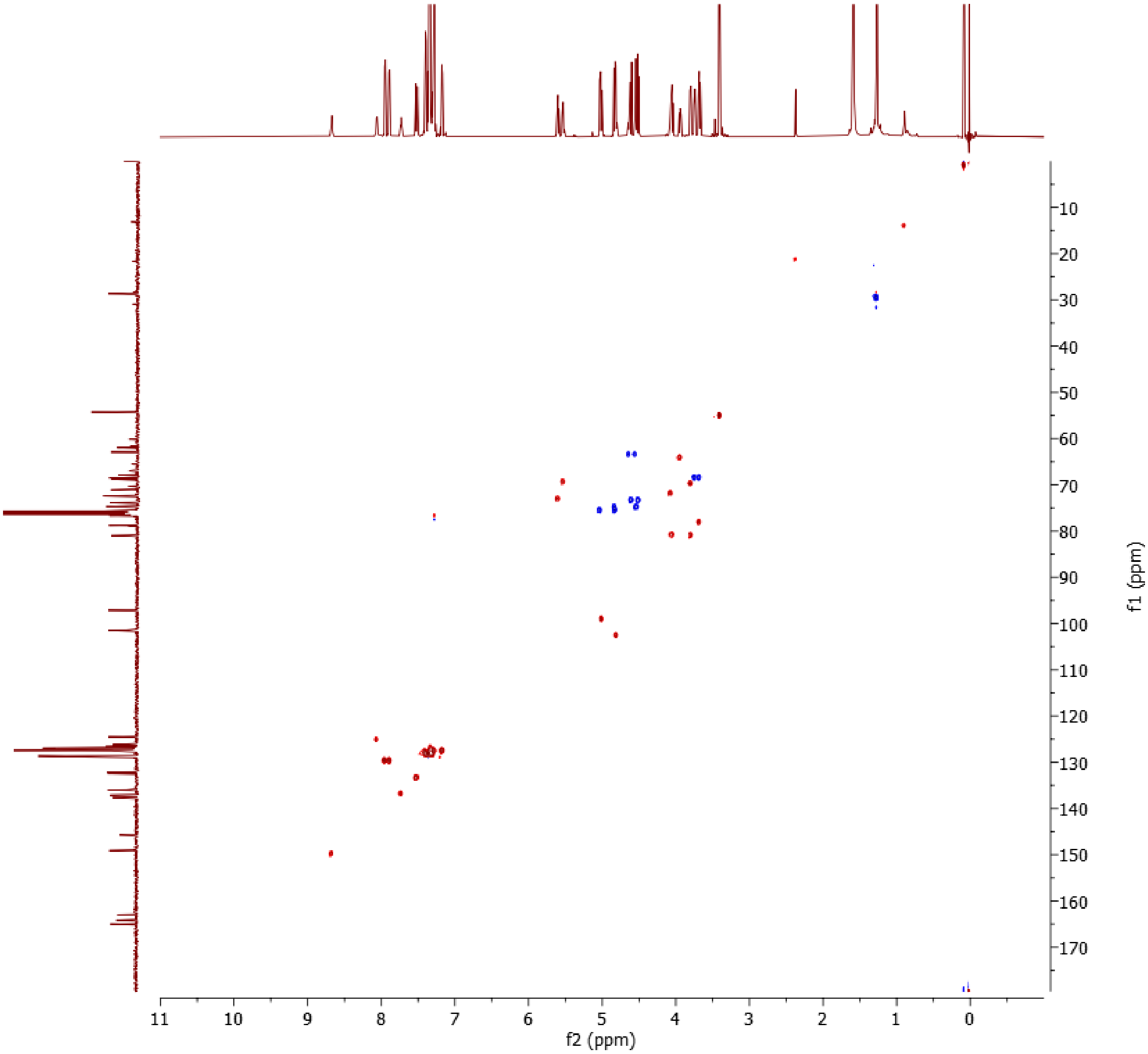

Figure S28: ^{1}H NMR and ^{13}C(^{1}H) NMR HSQC Spectrum (700 MHz, 176 MHz, $CDCl_3$)

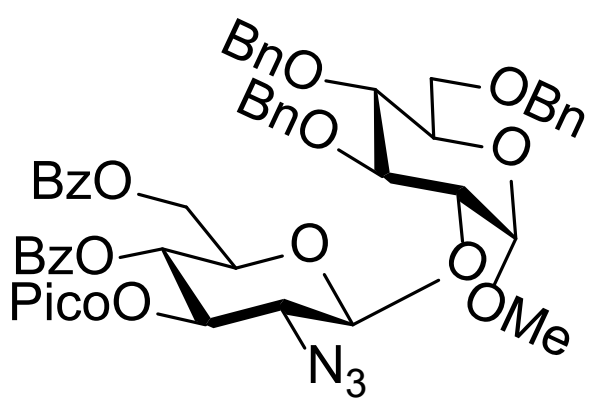

21

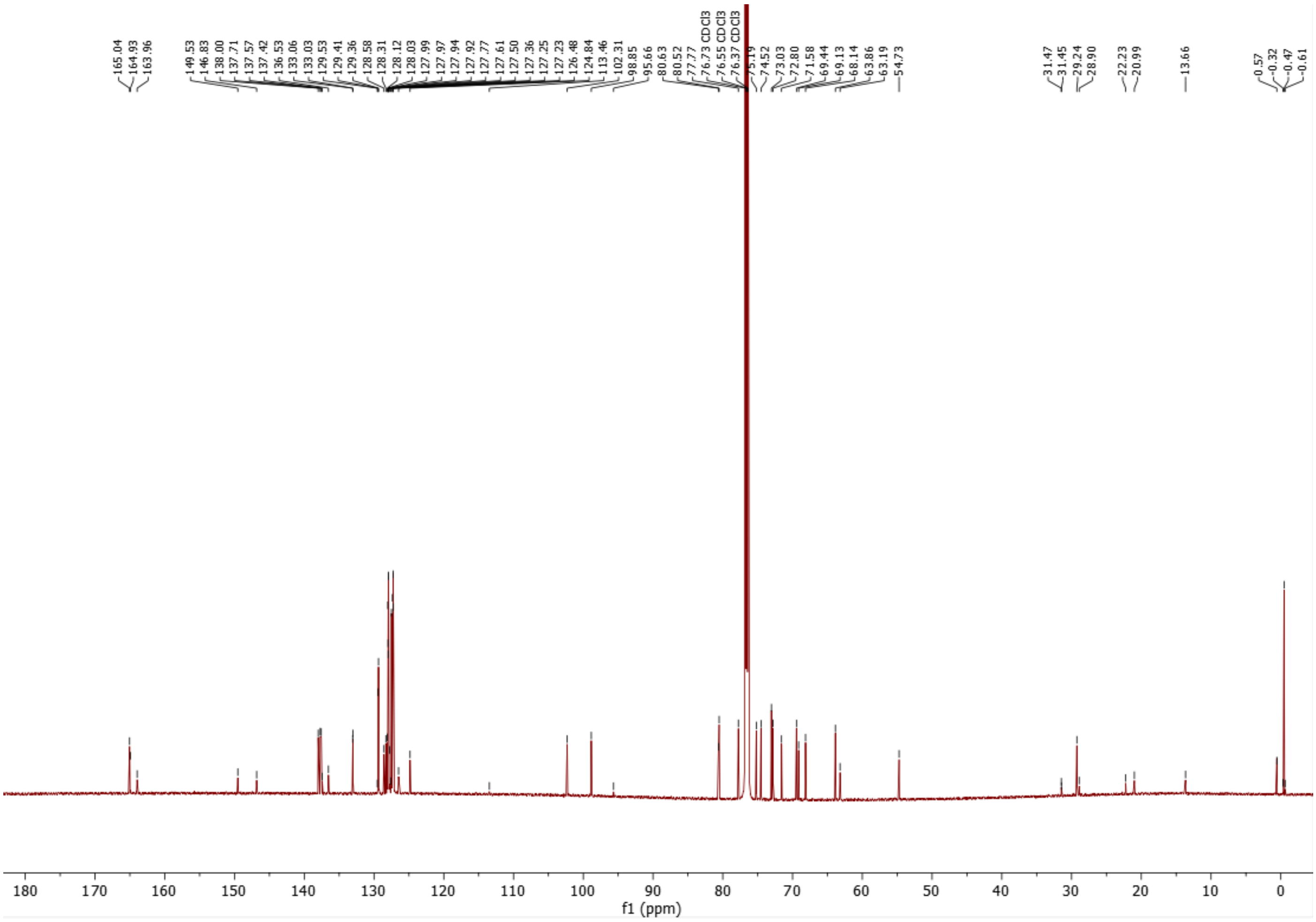

Figure S29: ^{13}C NMR (176 MHz, $CDCl_3$)

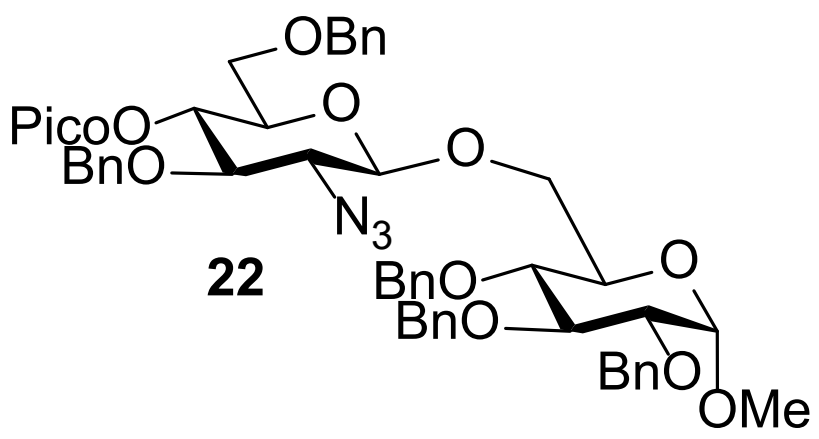

22

β-only

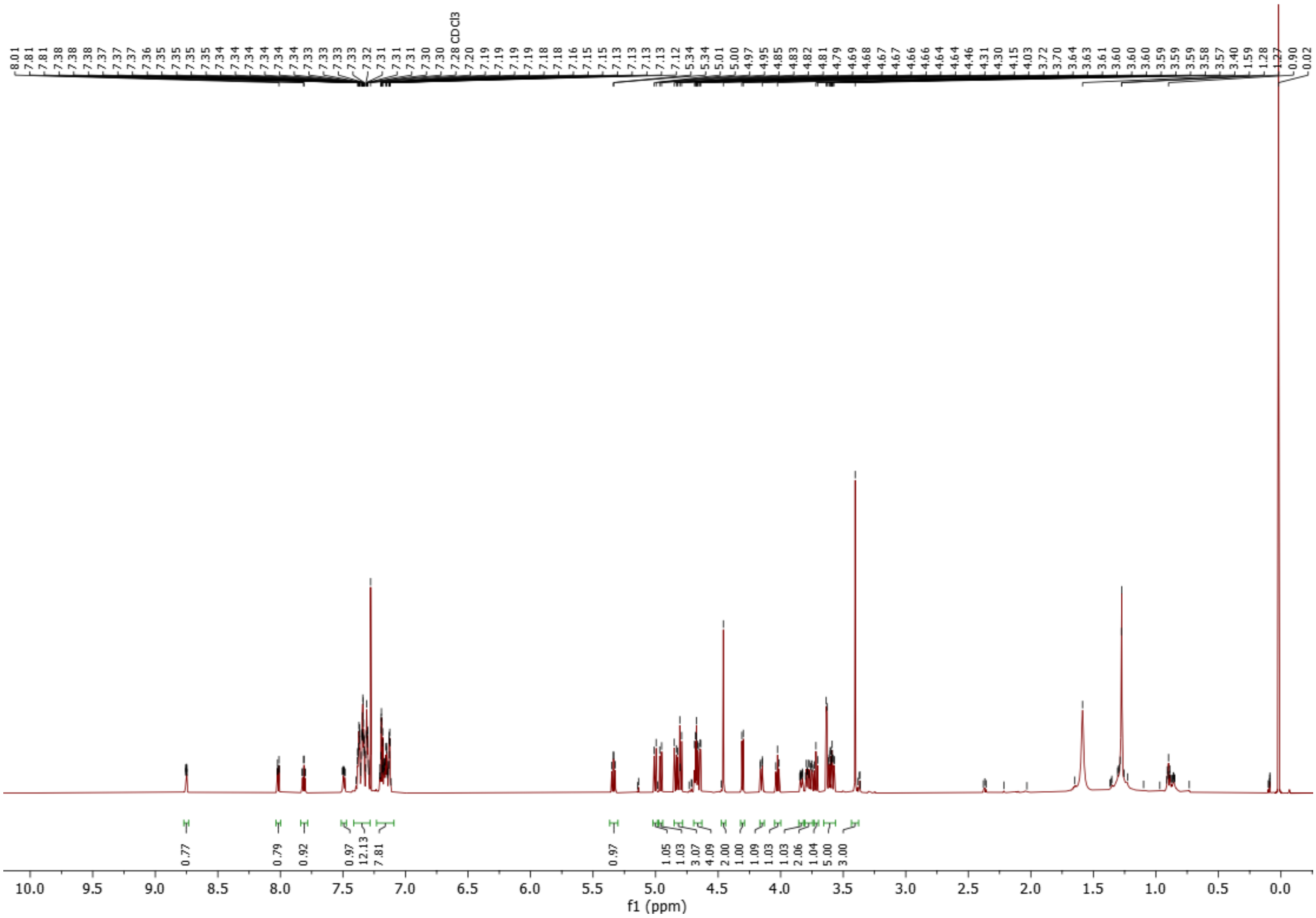

Figure S30: ^{1}H NMR (700 MHz, $CDCl_3$)

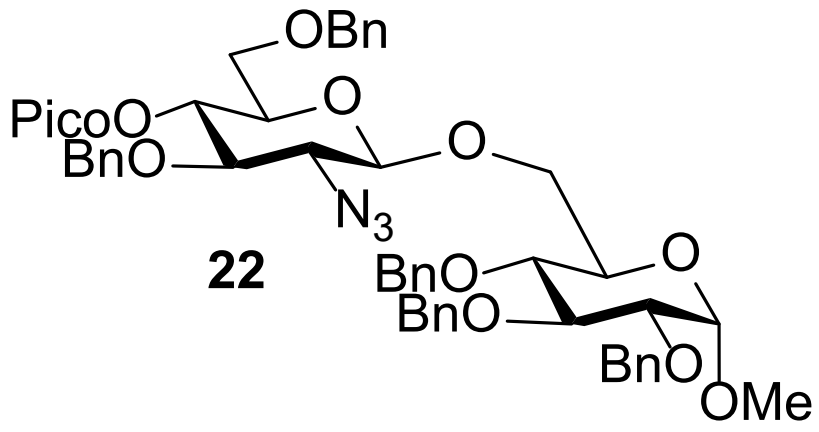

β-only

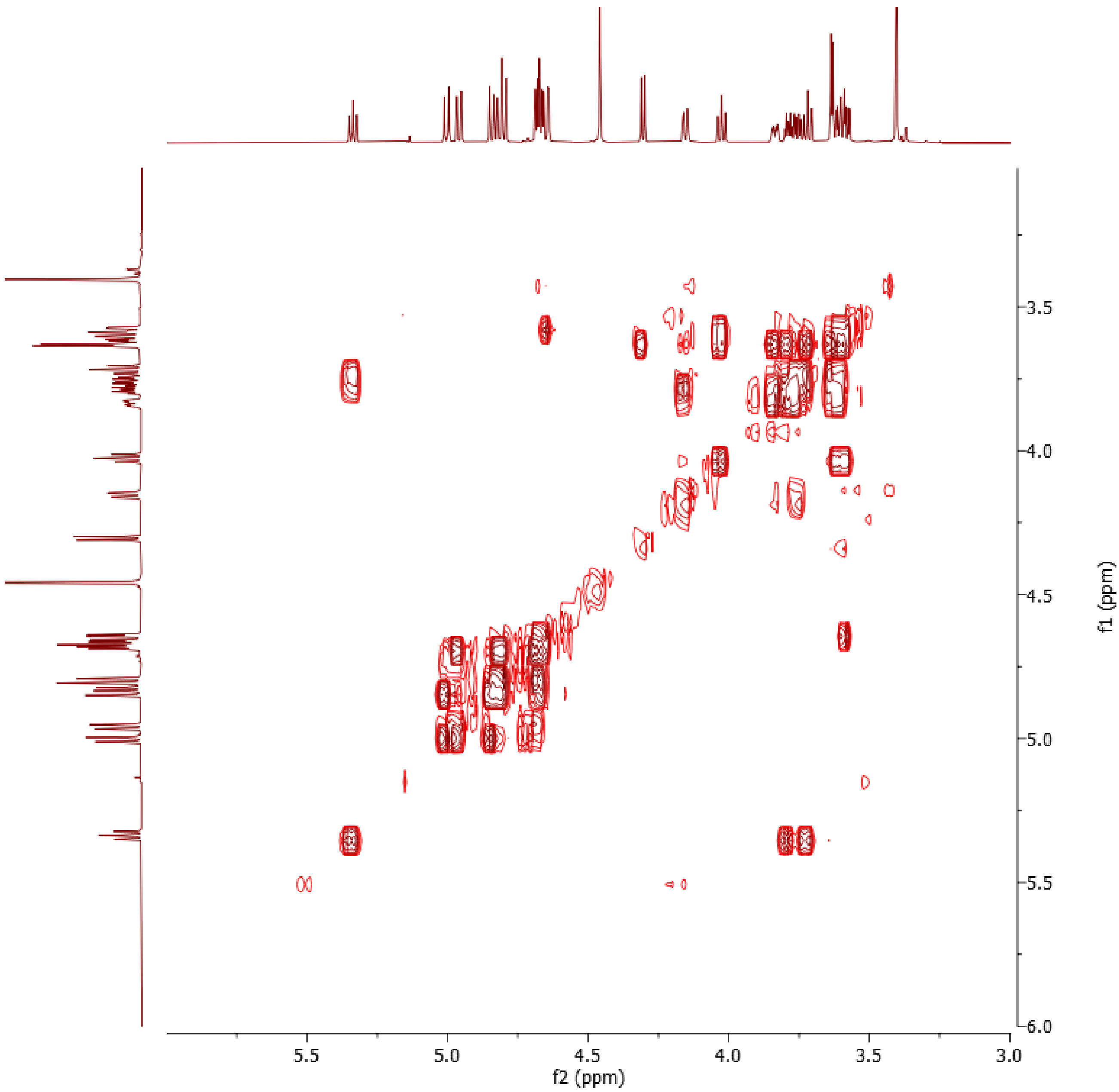

Figure S31: COSY NMR (700 MHz, $CDCl_3$)

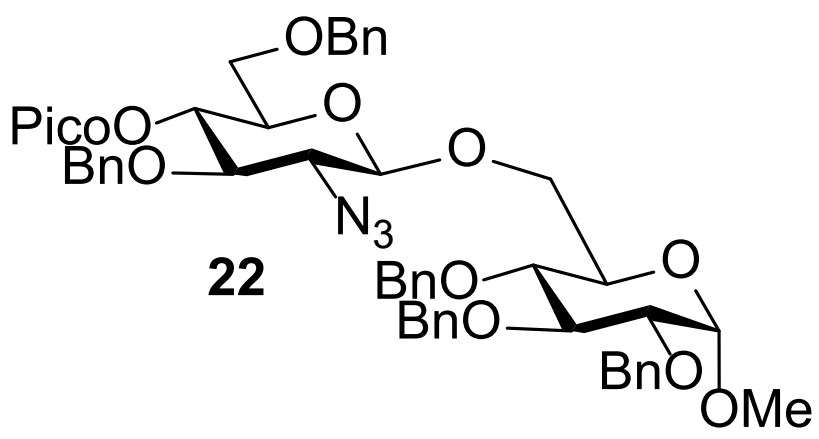

β-only

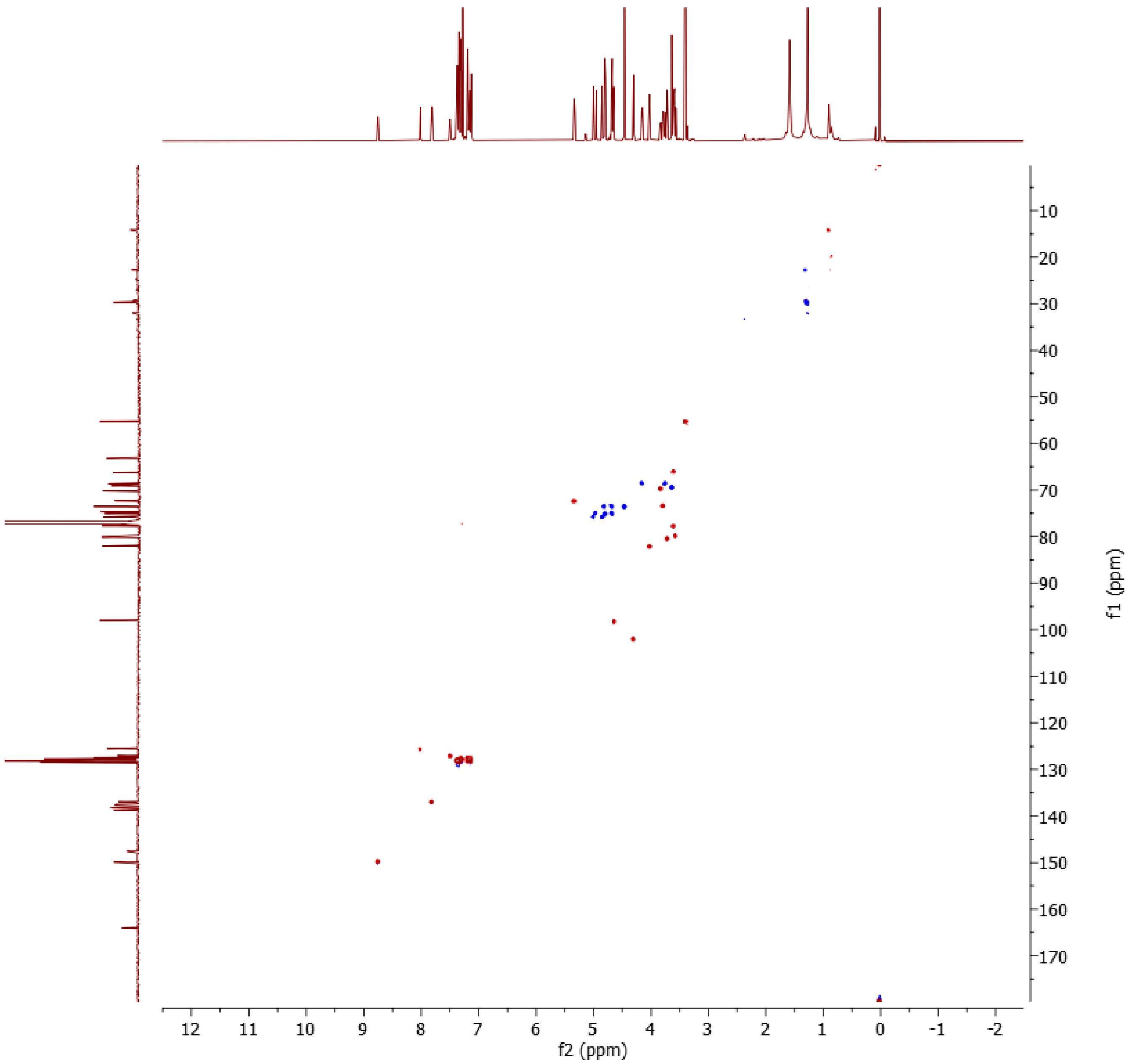

Figure S32: ^{1}H NMR and ^{13}C(^{1}H) NMR HSQC Spectrum (700 MHz, 176 MHz, $CDCl_3$)

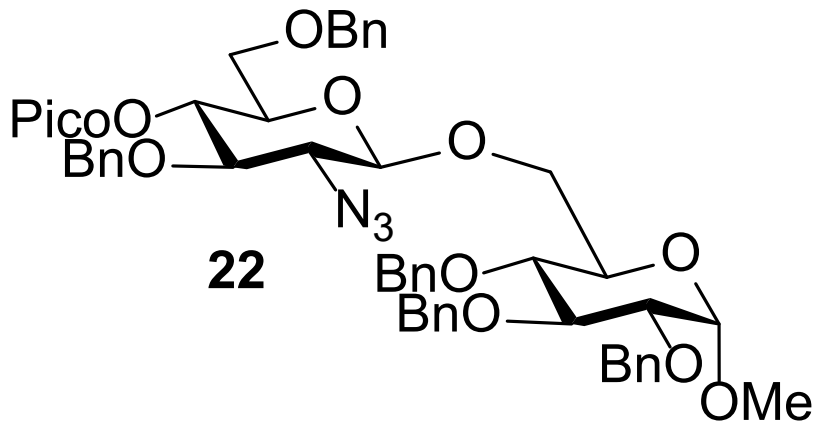

22

β-only

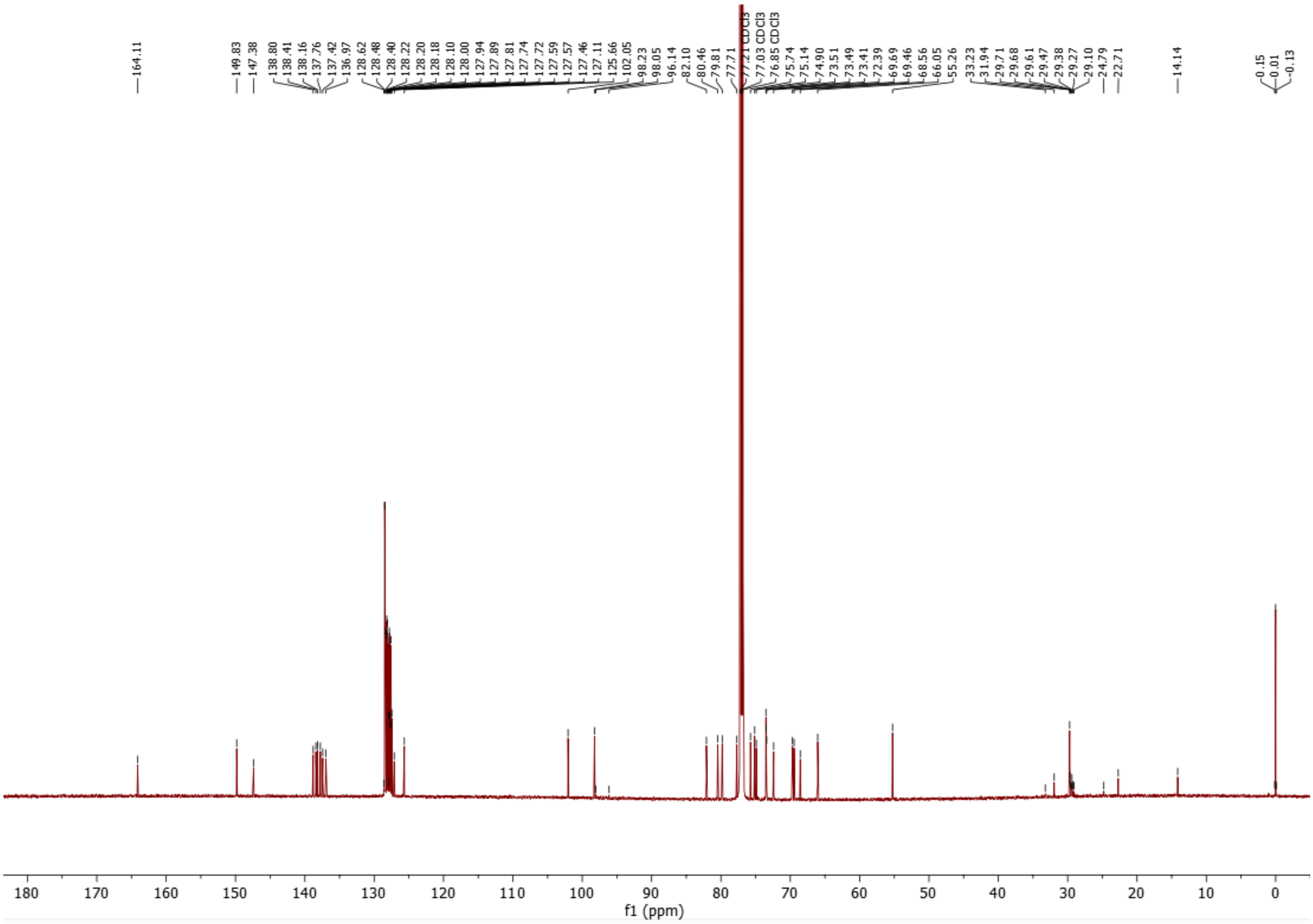

Figure S33: ^{13}C NMR (176 MHz, $CDCl_3$)

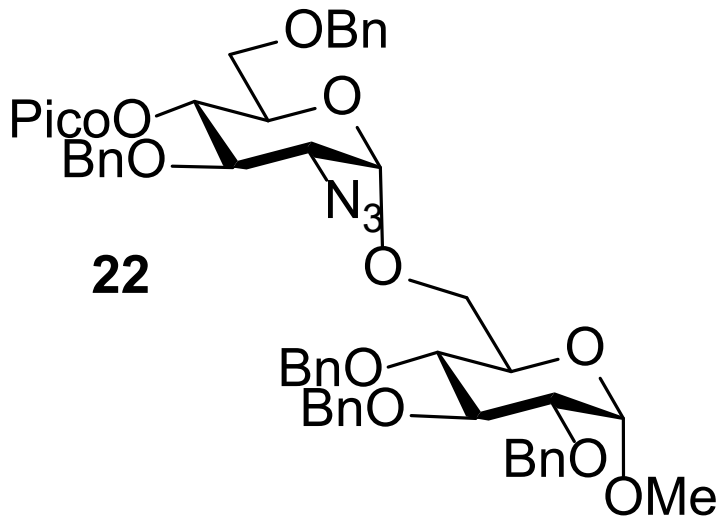

22

α-only

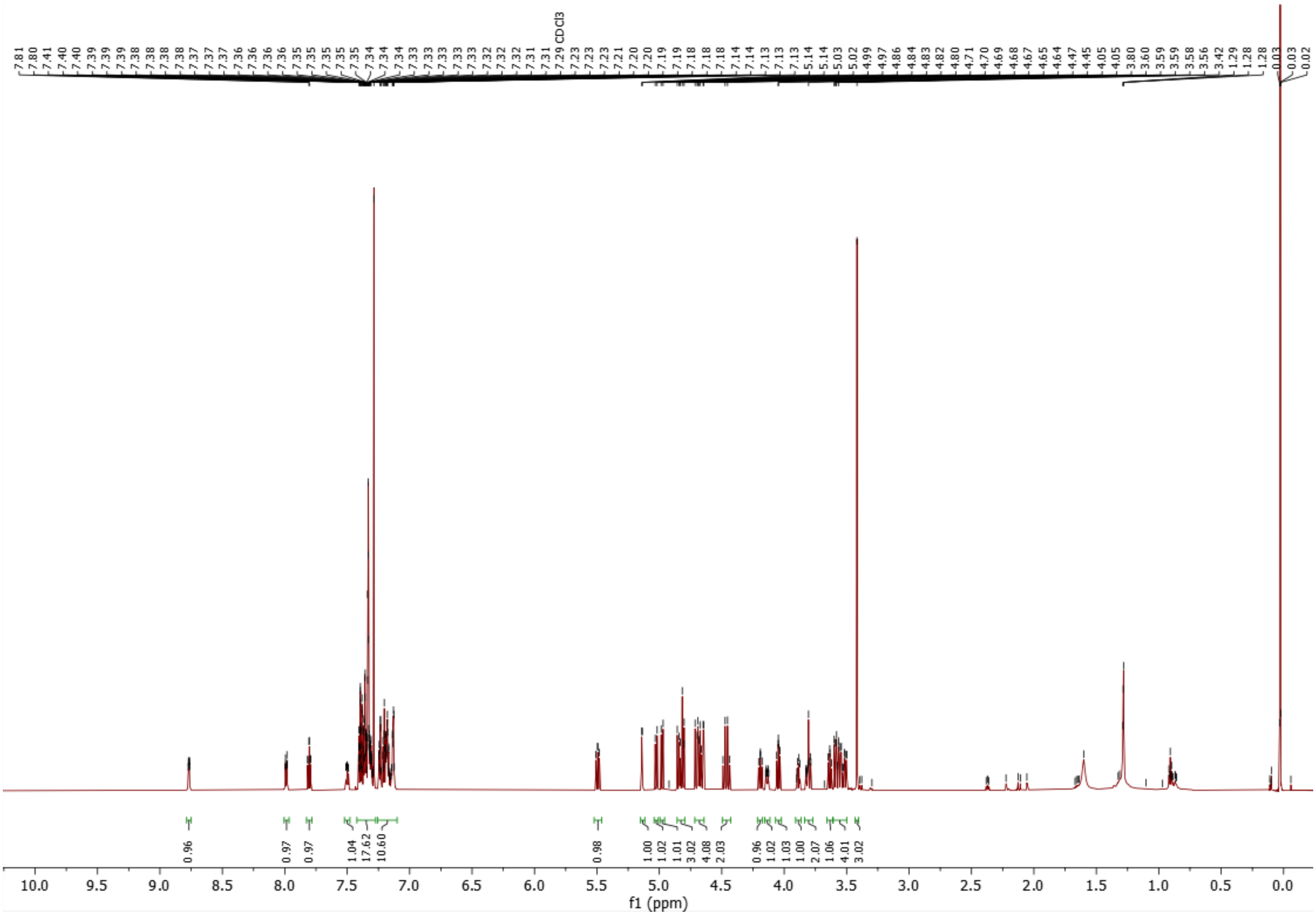

Figure S34: ^{1}H NMR (700 MHz, $CDCl_3$)

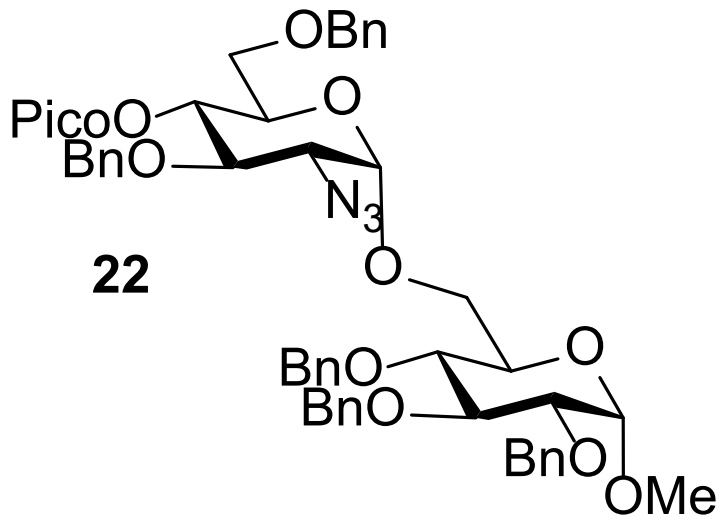

22

α-only

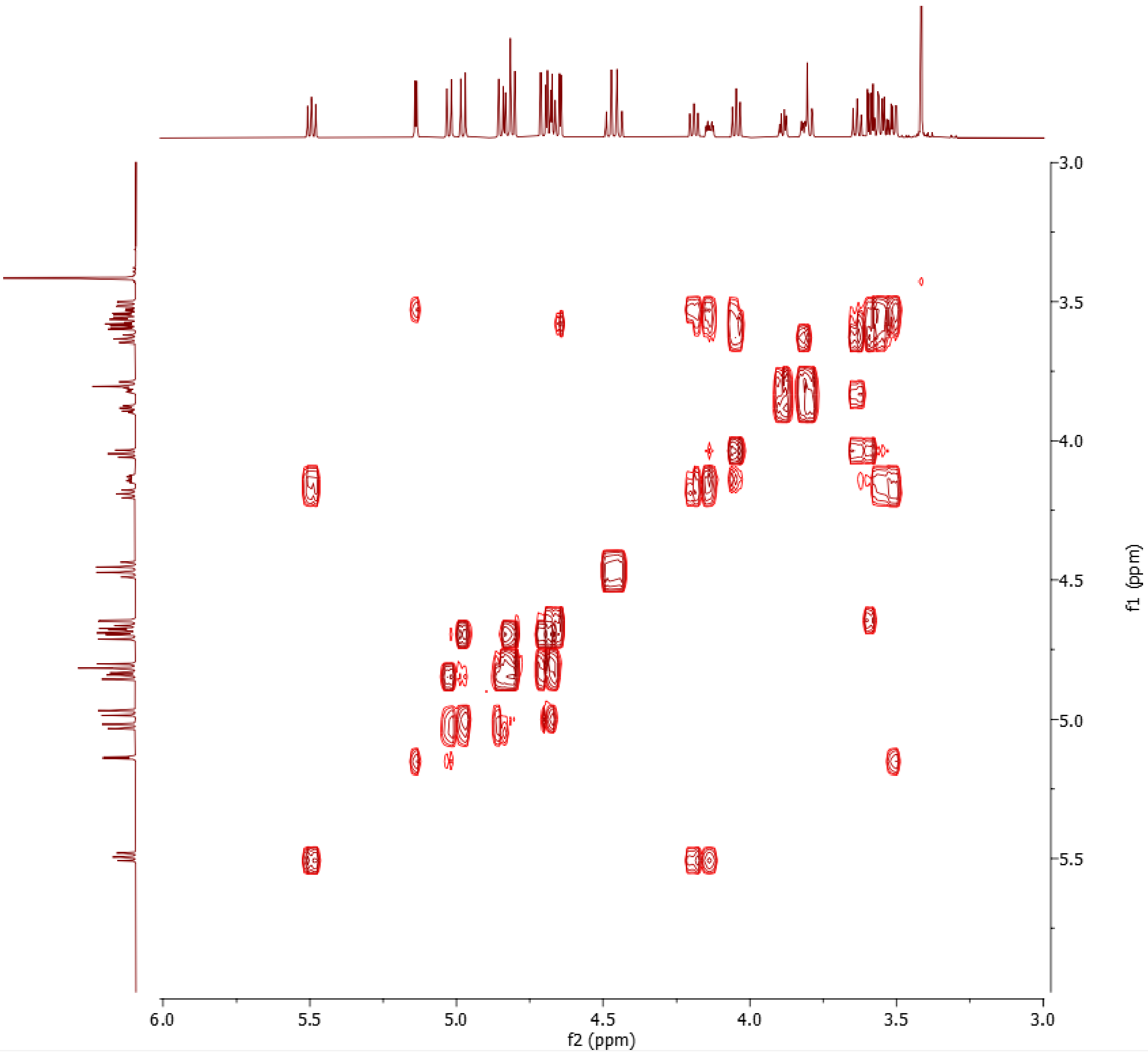

Figure S35: COSY NMR (700 MHz, $CDCl_3$)

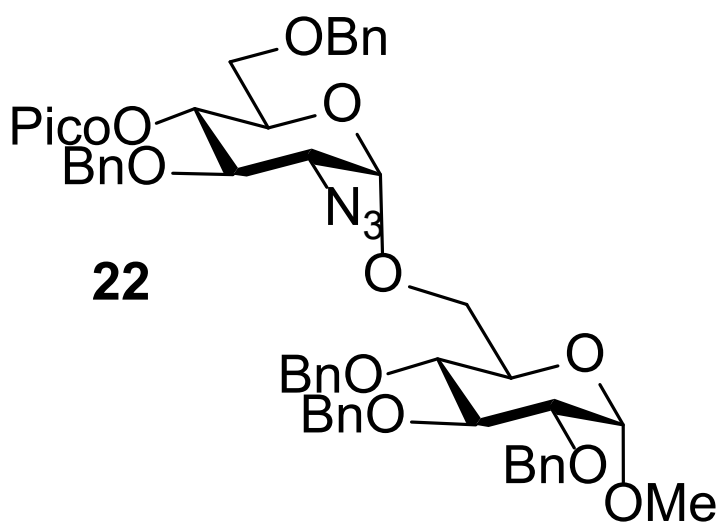

22

α-only

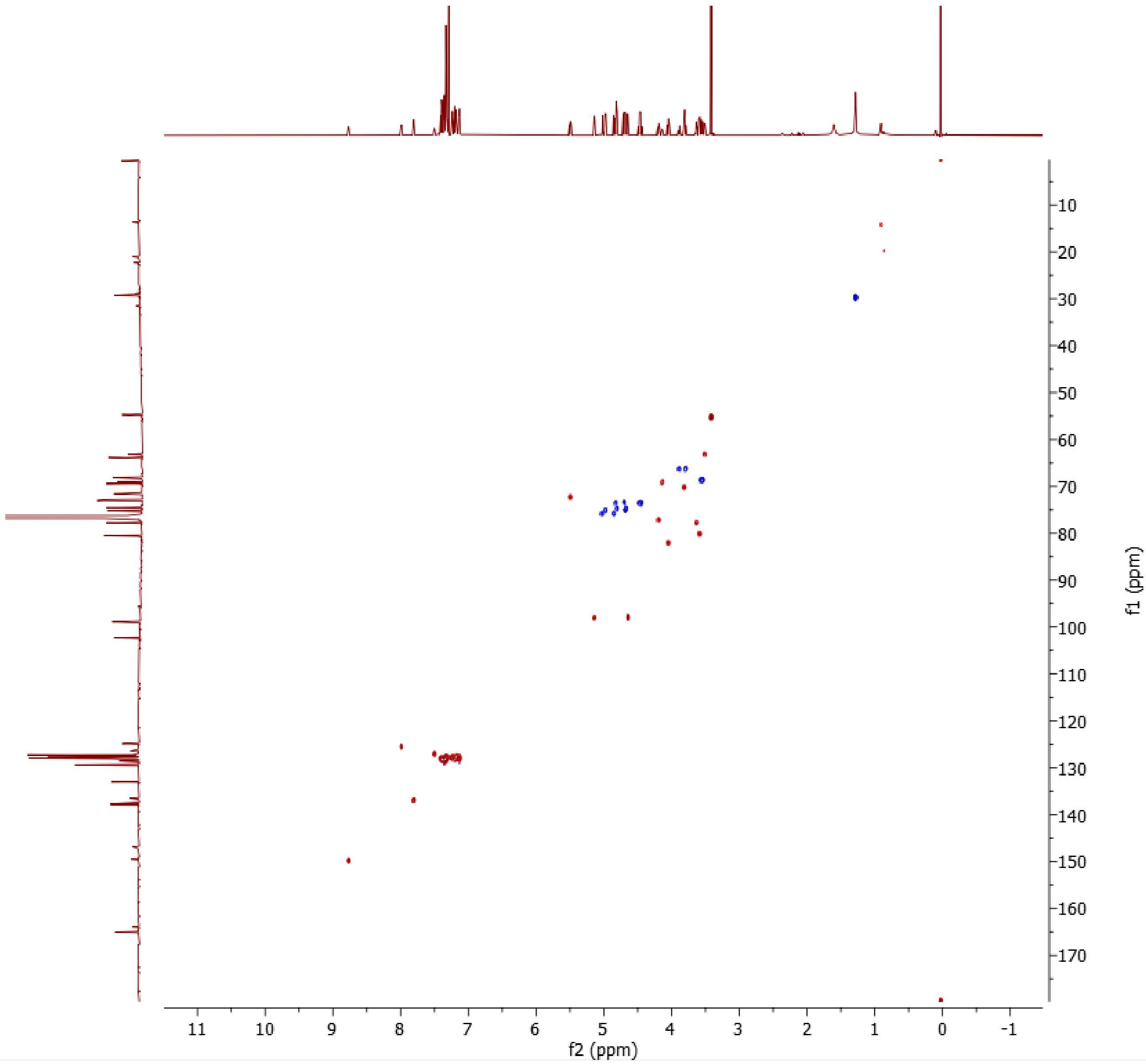

Figure S36: ^{1}H NMR and ^{13}C(^{1}H) NMR HSQC Spectrum (700 MHz, 176 MHz, $CDCl_3$)

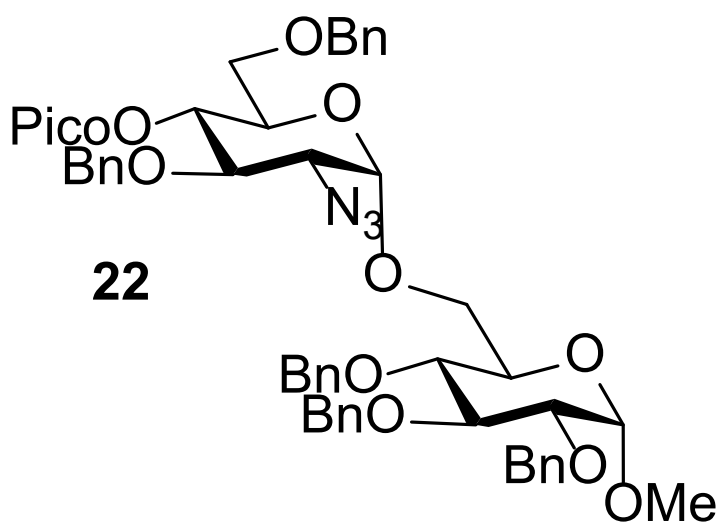

22

α-only

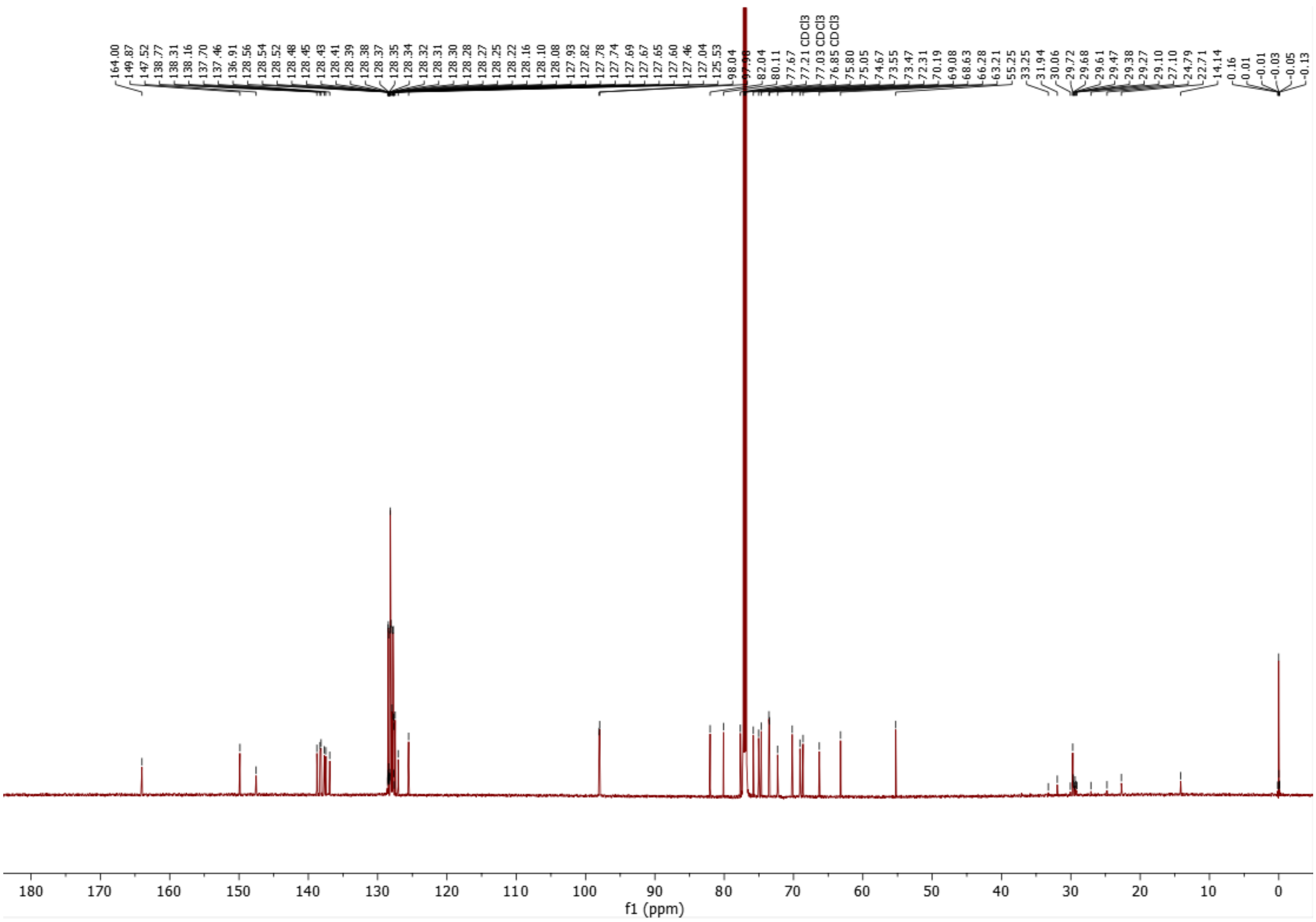

Figure S37: ^{13}C NMR (176 MHz, $CDCl_3$)

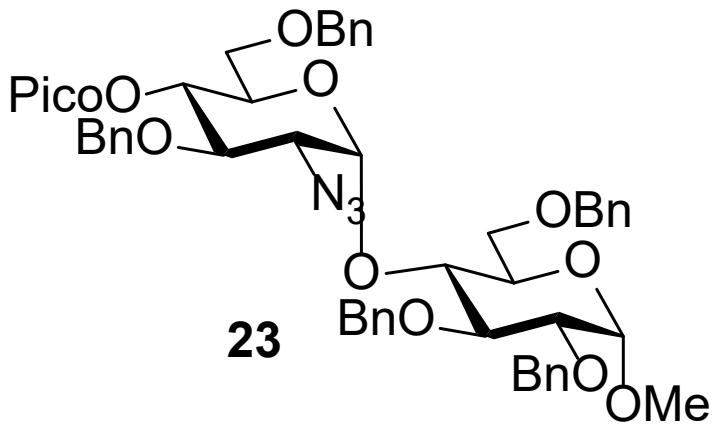

23

α-only

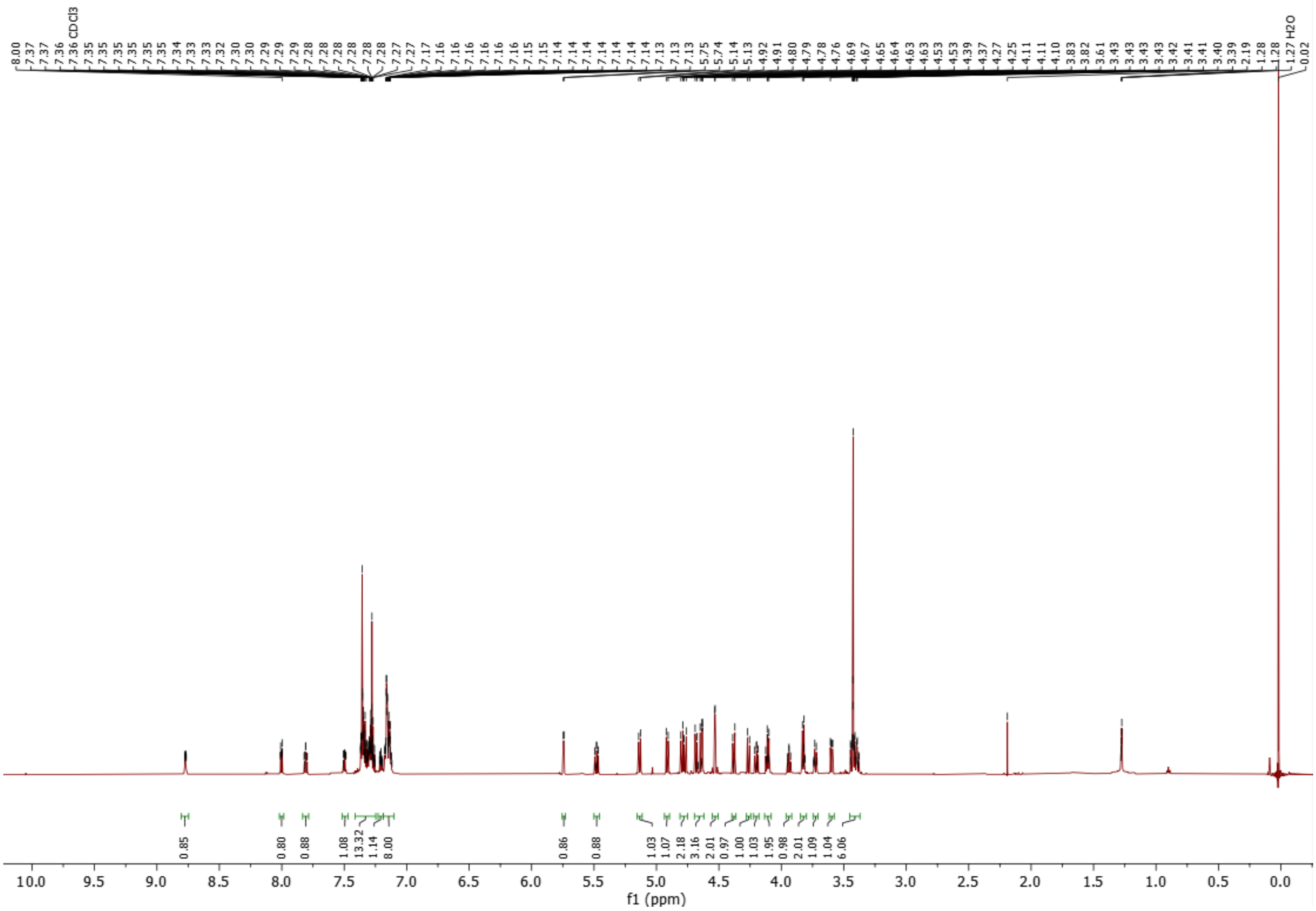

Figure S38: ^{1}H NMR (700 MHz, $CDCl_3$)

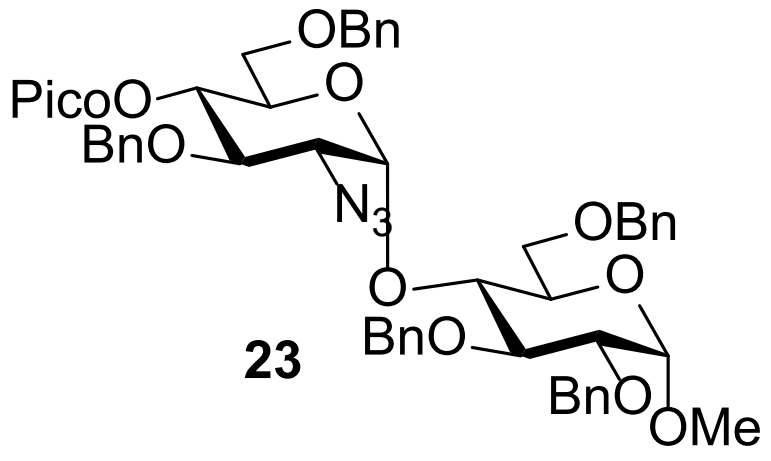

23

α-only

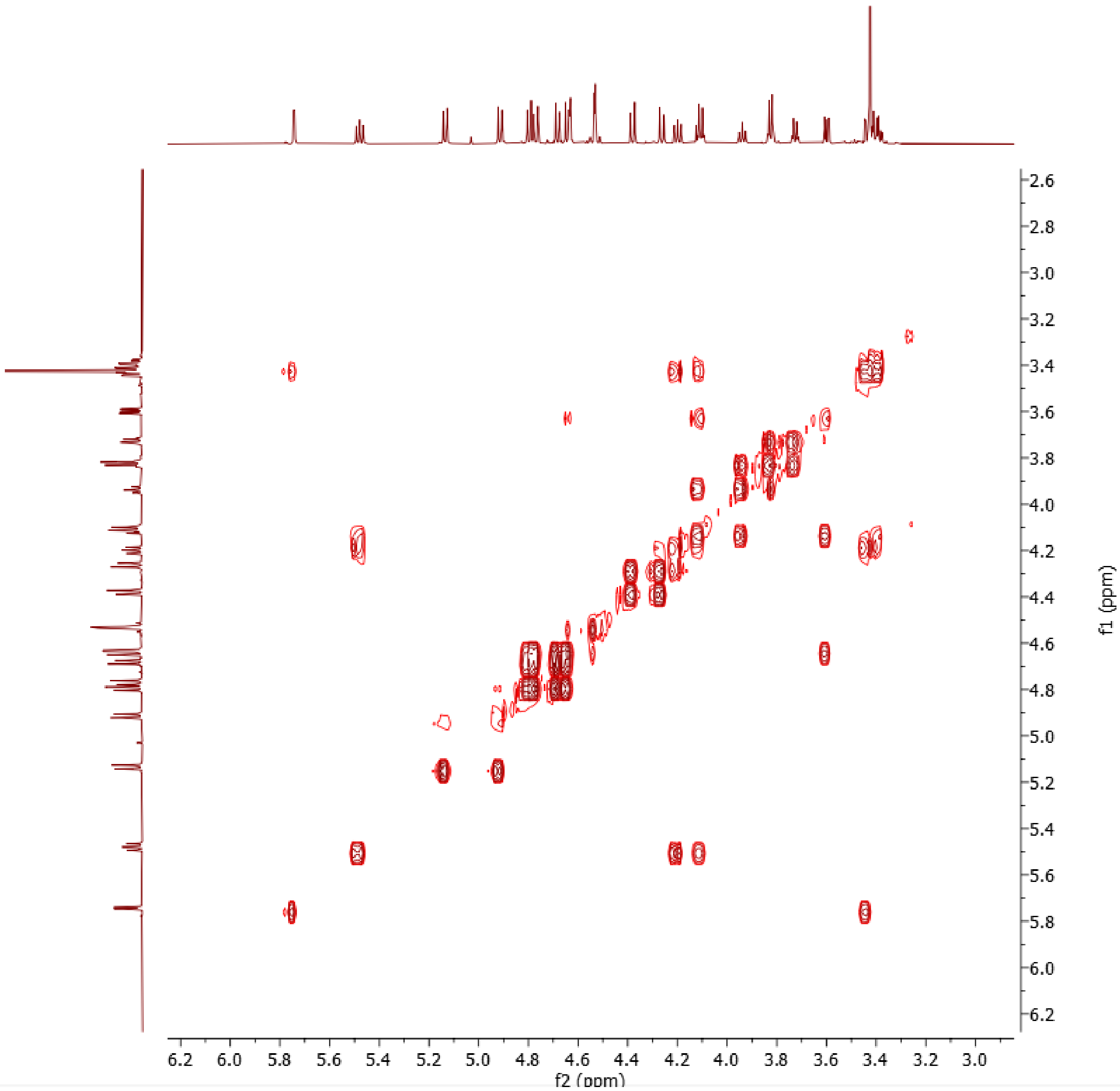

Figure S39: COSY NMR (700 MHz, $CDCl_3$)

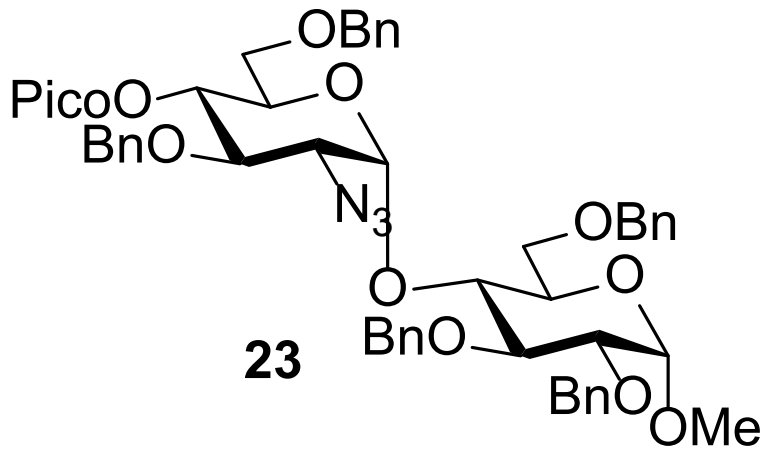

23

α-only

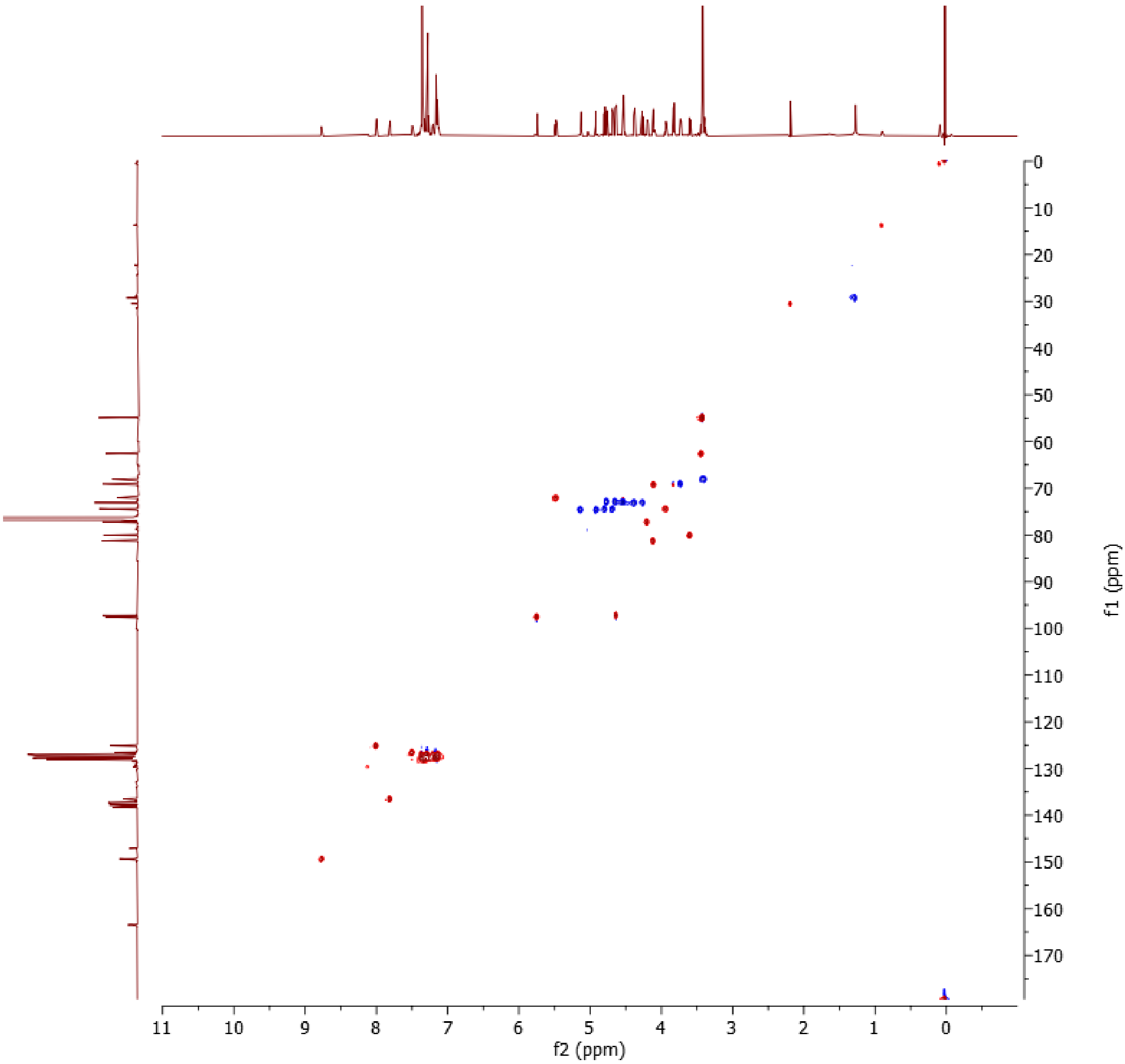

Figure S40: ^{1}H NMR and $^{13}C(^{1}H)$ NMR HSQC Spectrum (700 MHz, 176 MHz, $CDCl_3$)

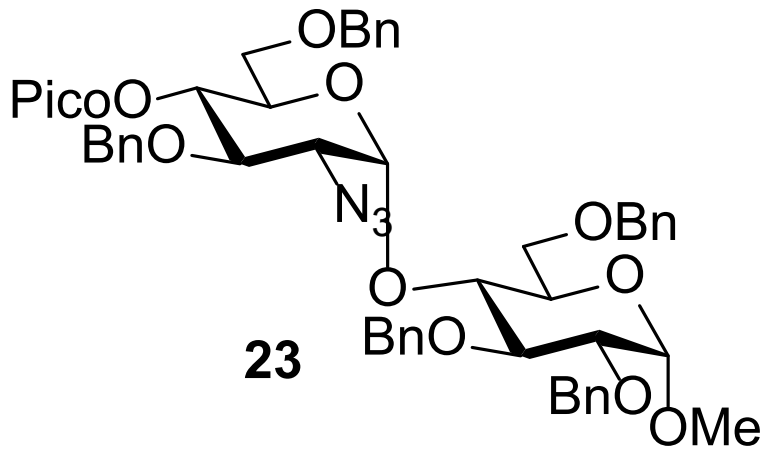

23

α-only

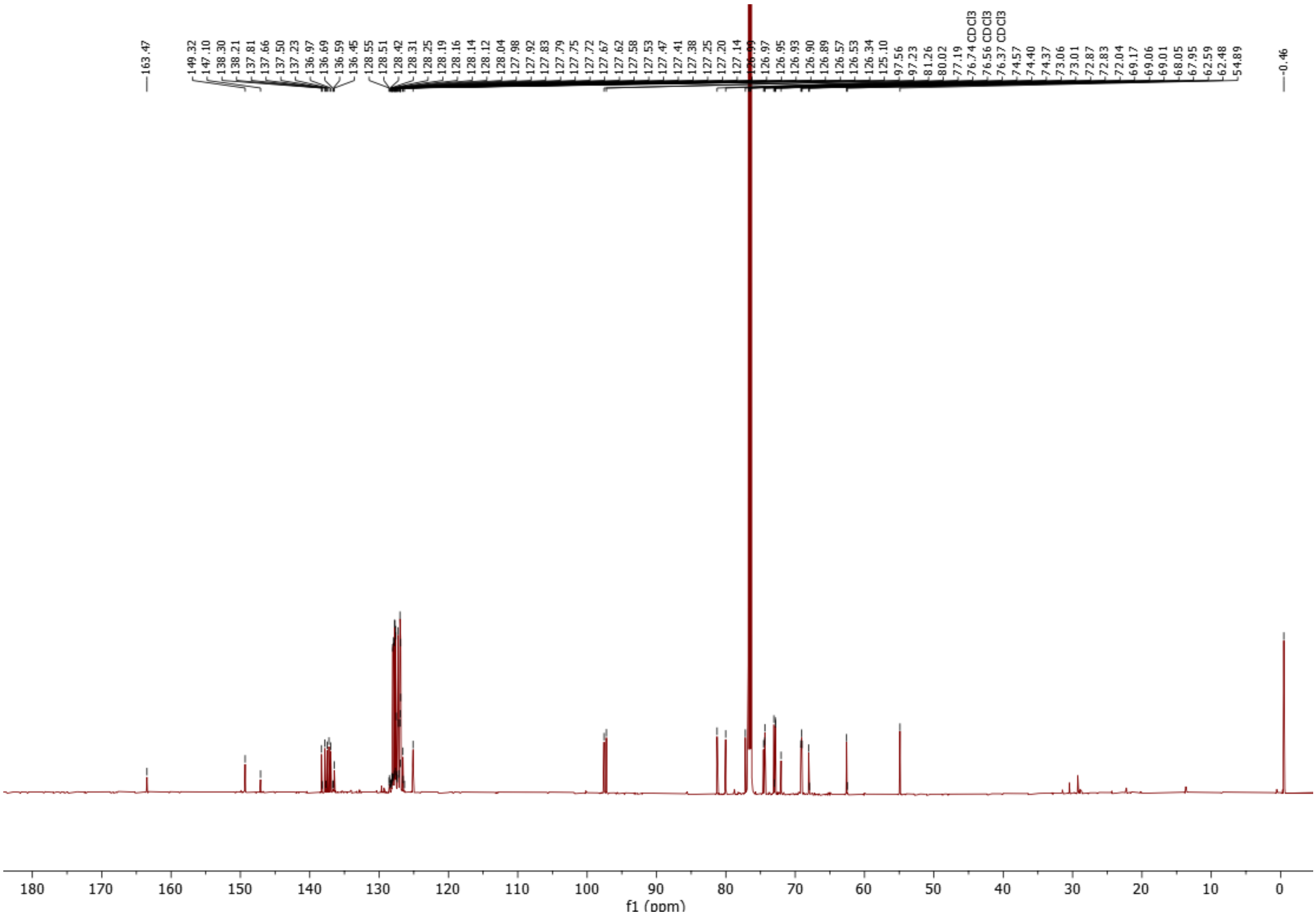

Figure S41: ^{13}C NMR (176 MHz, $CDCl_3$)

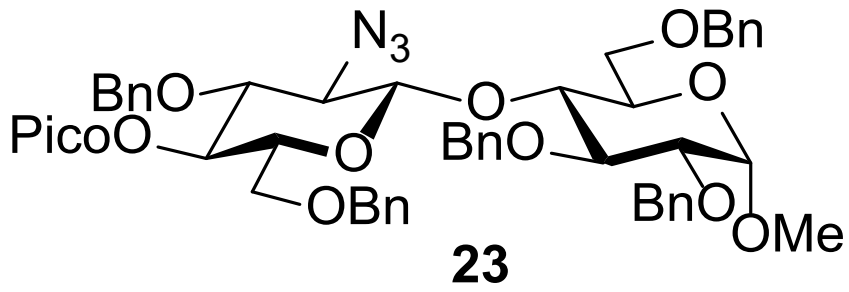

23

β-only

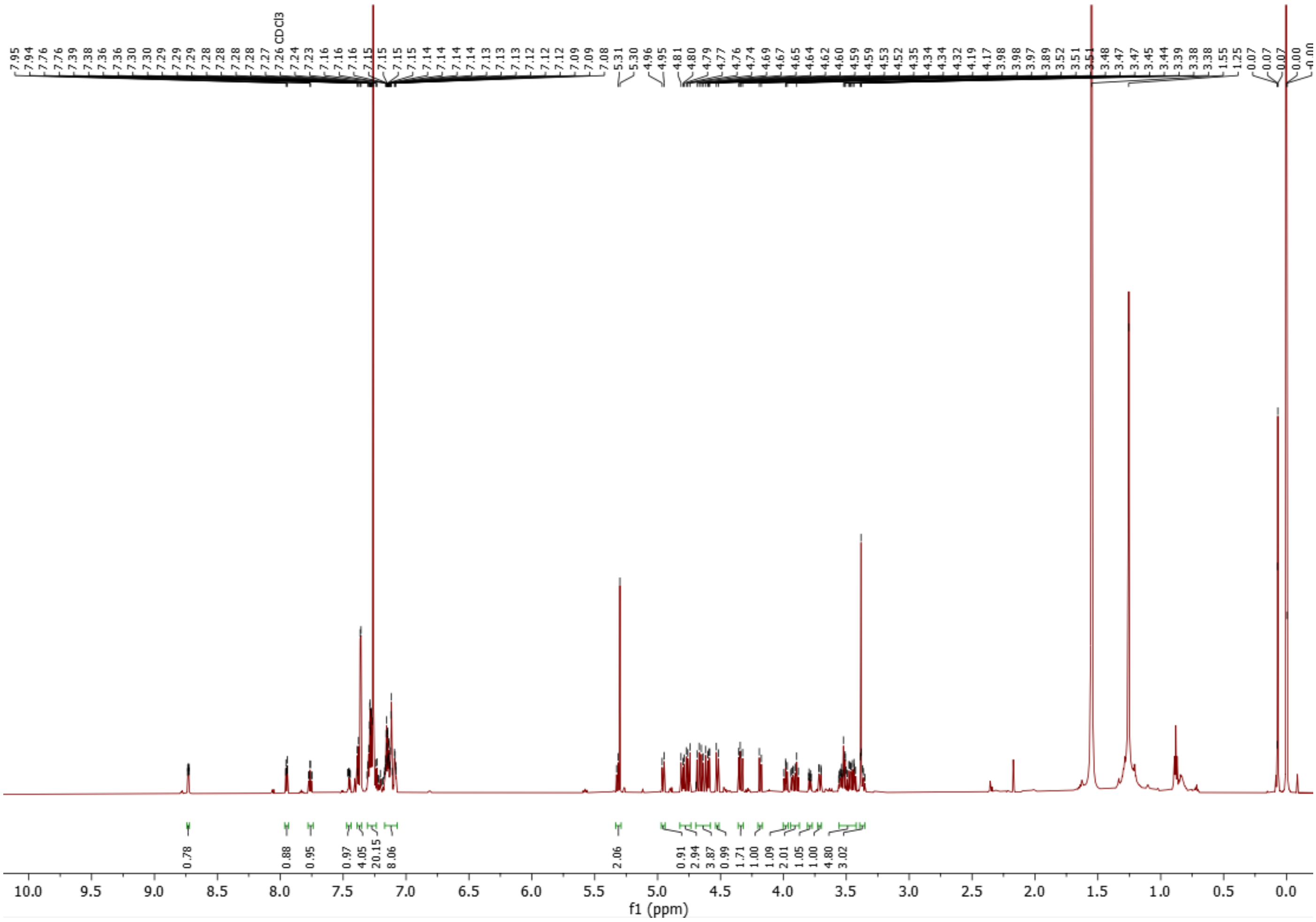

Figure S42: ^{1}H NMR (700 MHz, $CDCl_3$)

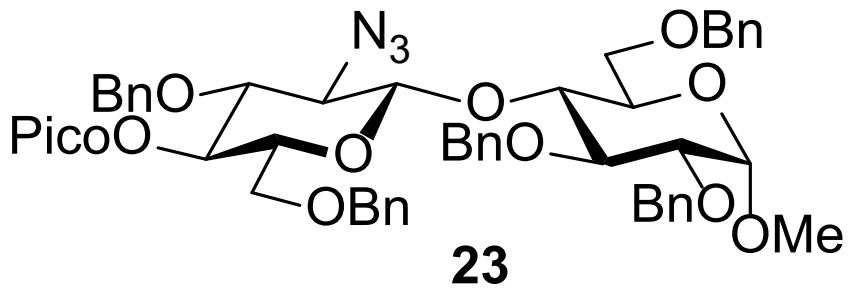

23

β-only

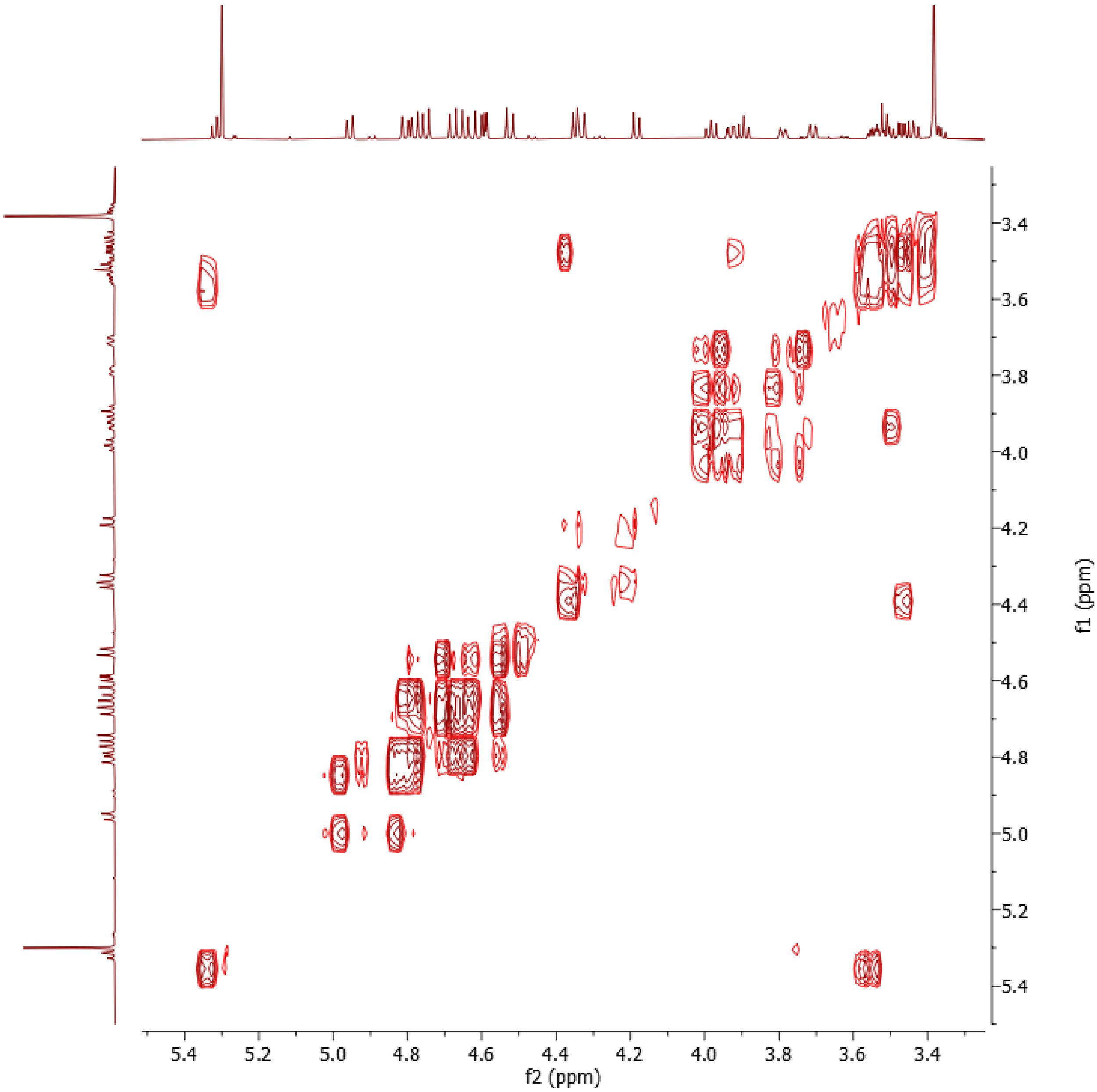

Figure S43: COSY NMR (700 MHz, $CDCl_3$)

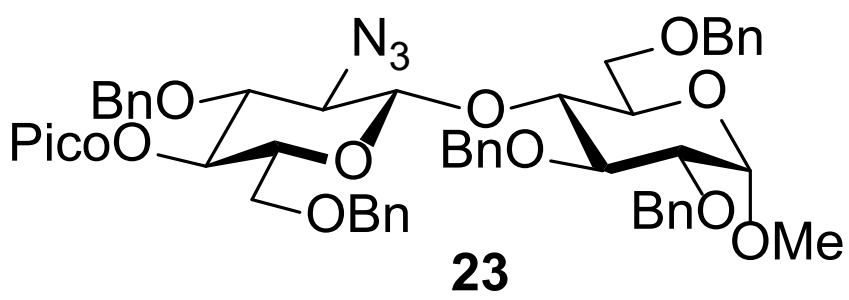

23

β-only

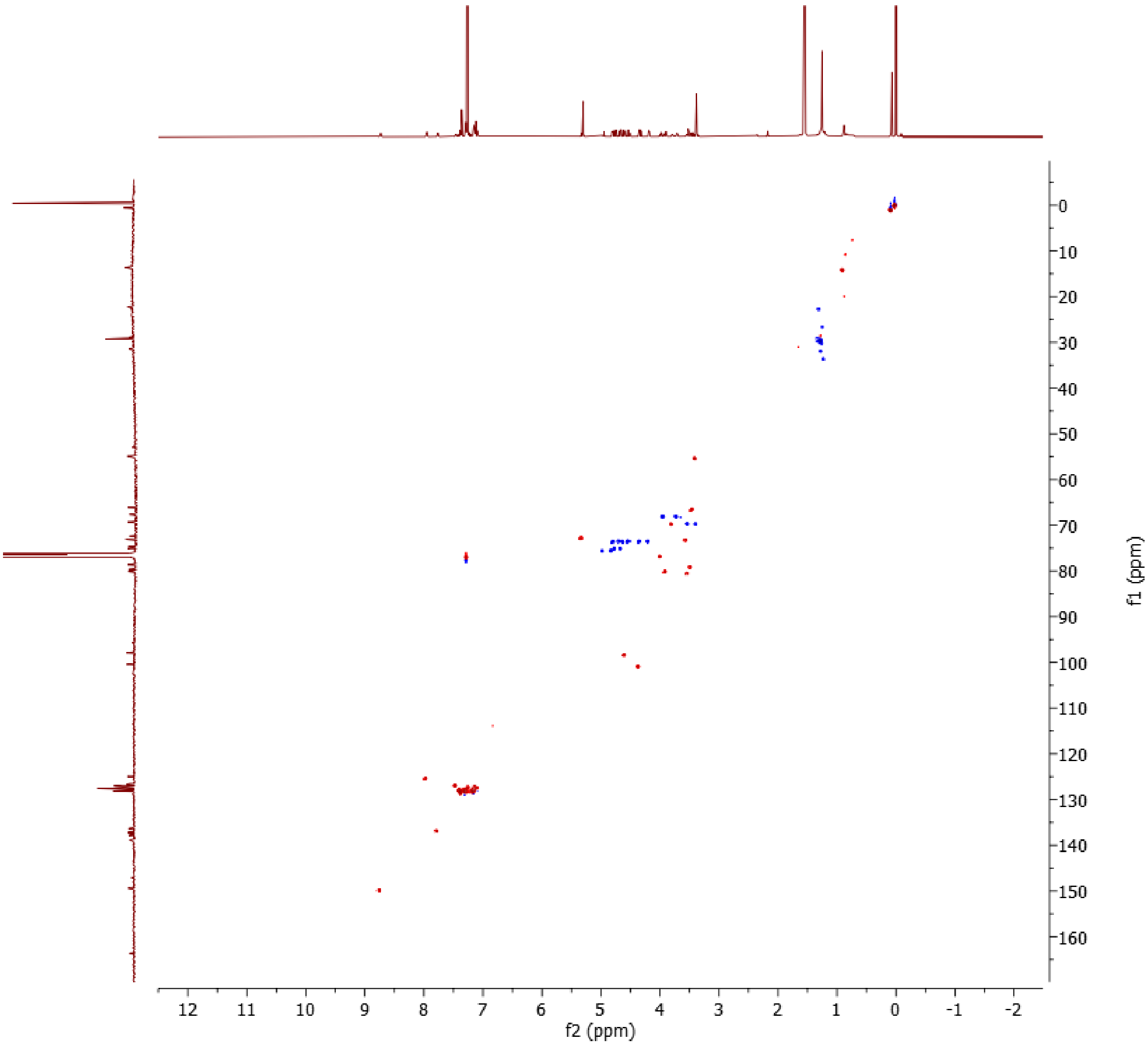

Figure S44: ^{1}H NMR and $^{13}C(^{1}H)$ NMR HSQC Spectrum (700 MHz, 176 MHz, $CDCl_3$)

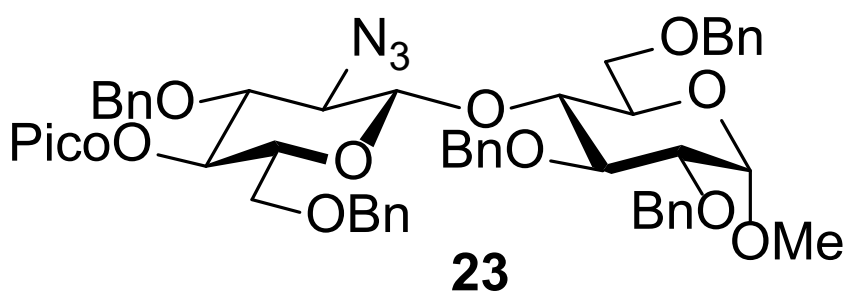

23

β-only

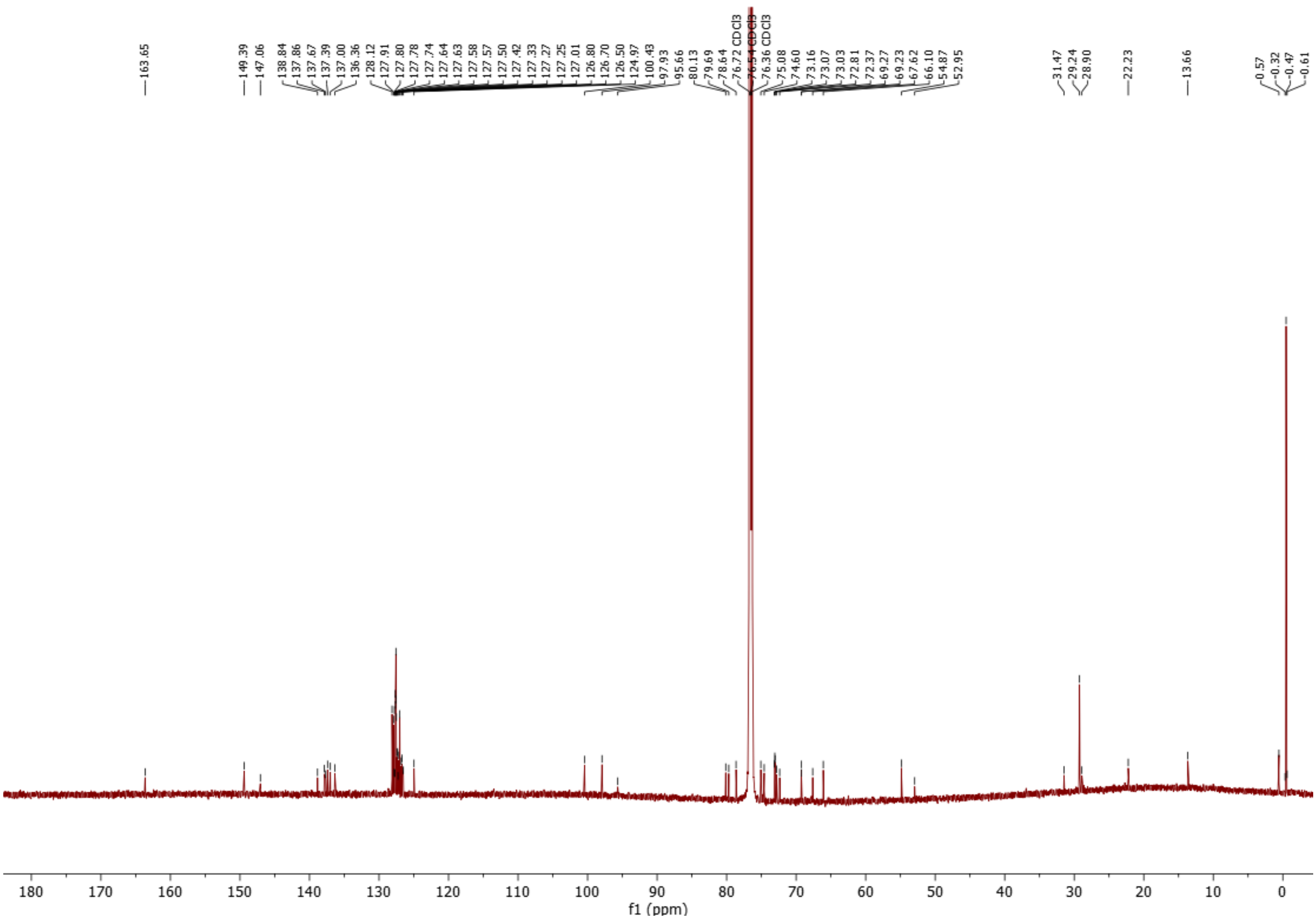

Figure S45: ^{13}C NMR (176 MHz, $CDCl_3$)

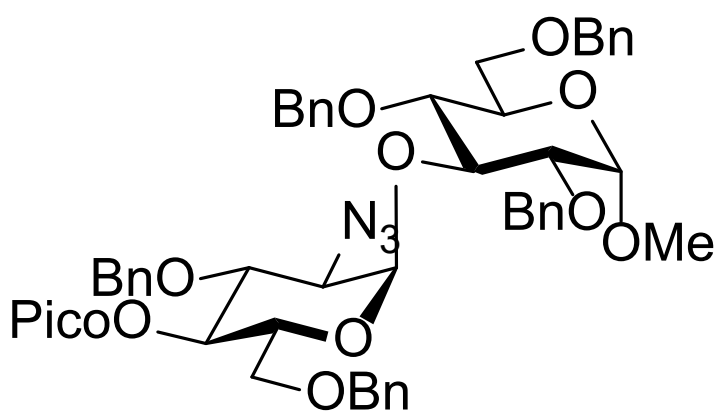

24

α-only

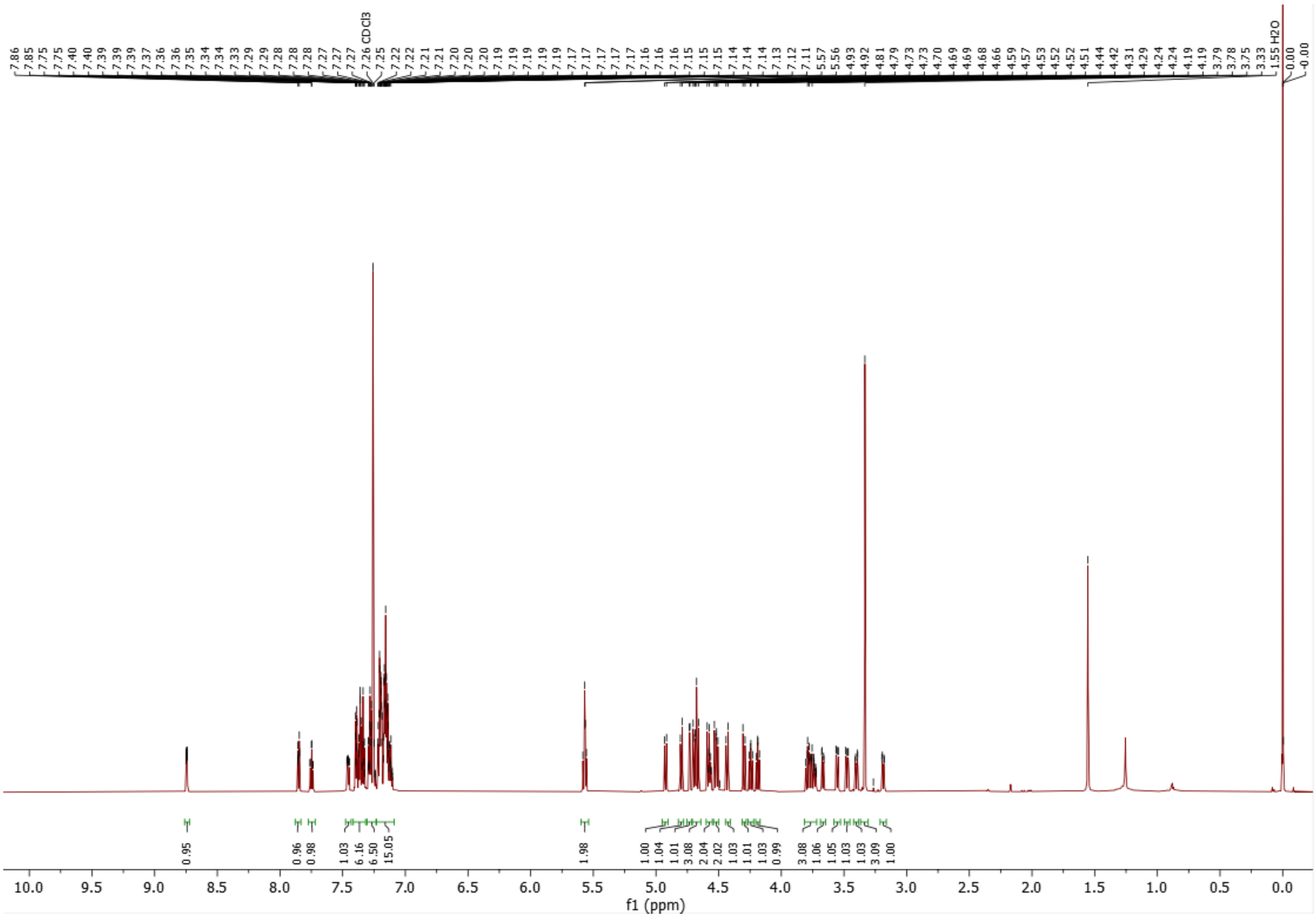

Figure S46: ^{1}H NMR (700 MHz, $CDCl_3$)

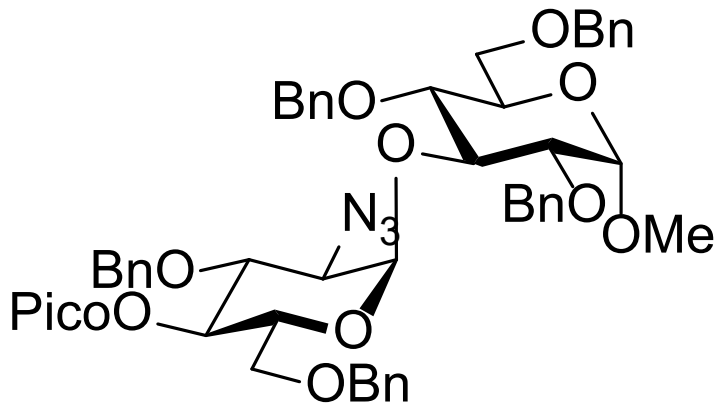

24

α-only

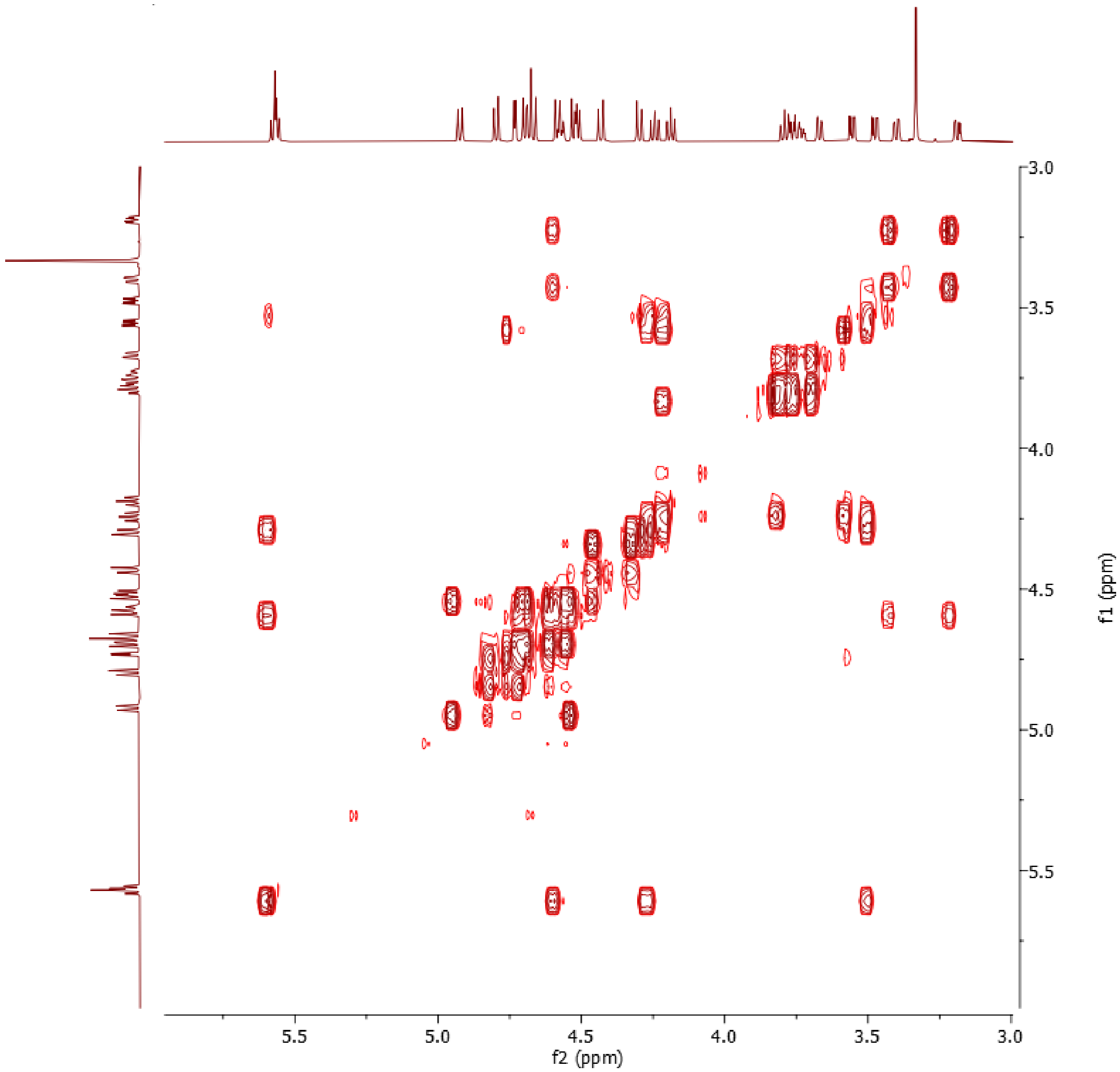

Figure S47: COSY NMR (700 MHz, $CDCl_3$)

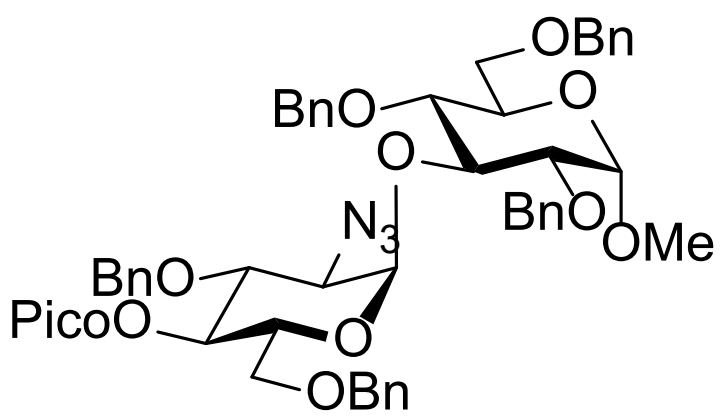

24

α-only

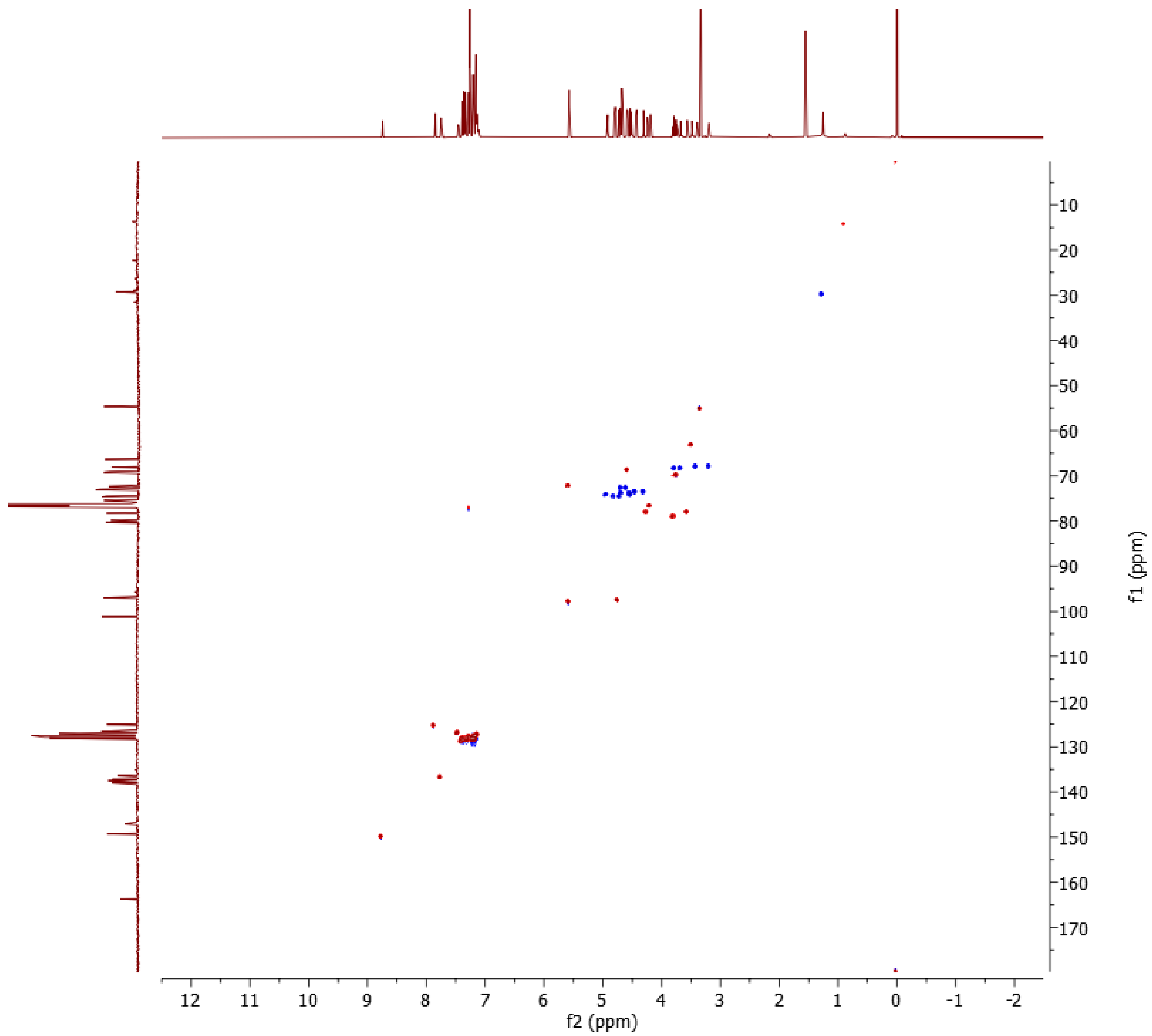

Figure S48: ^{1}H NMR and ^{13}C(^{1}H) NMR HSQC Spectrum (700 MHz, 176 MHz, $CDCl_3$)

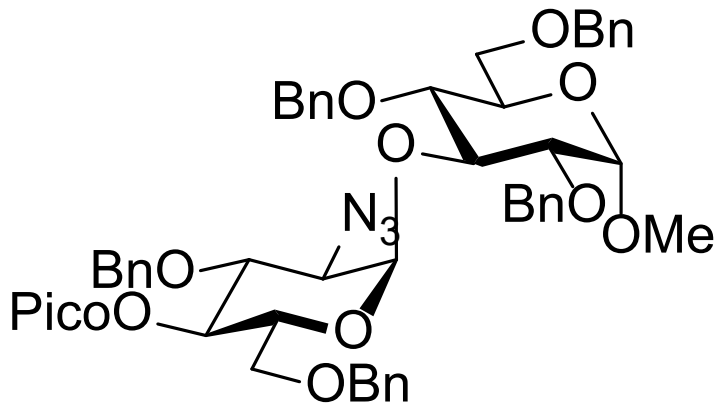

24

α-only

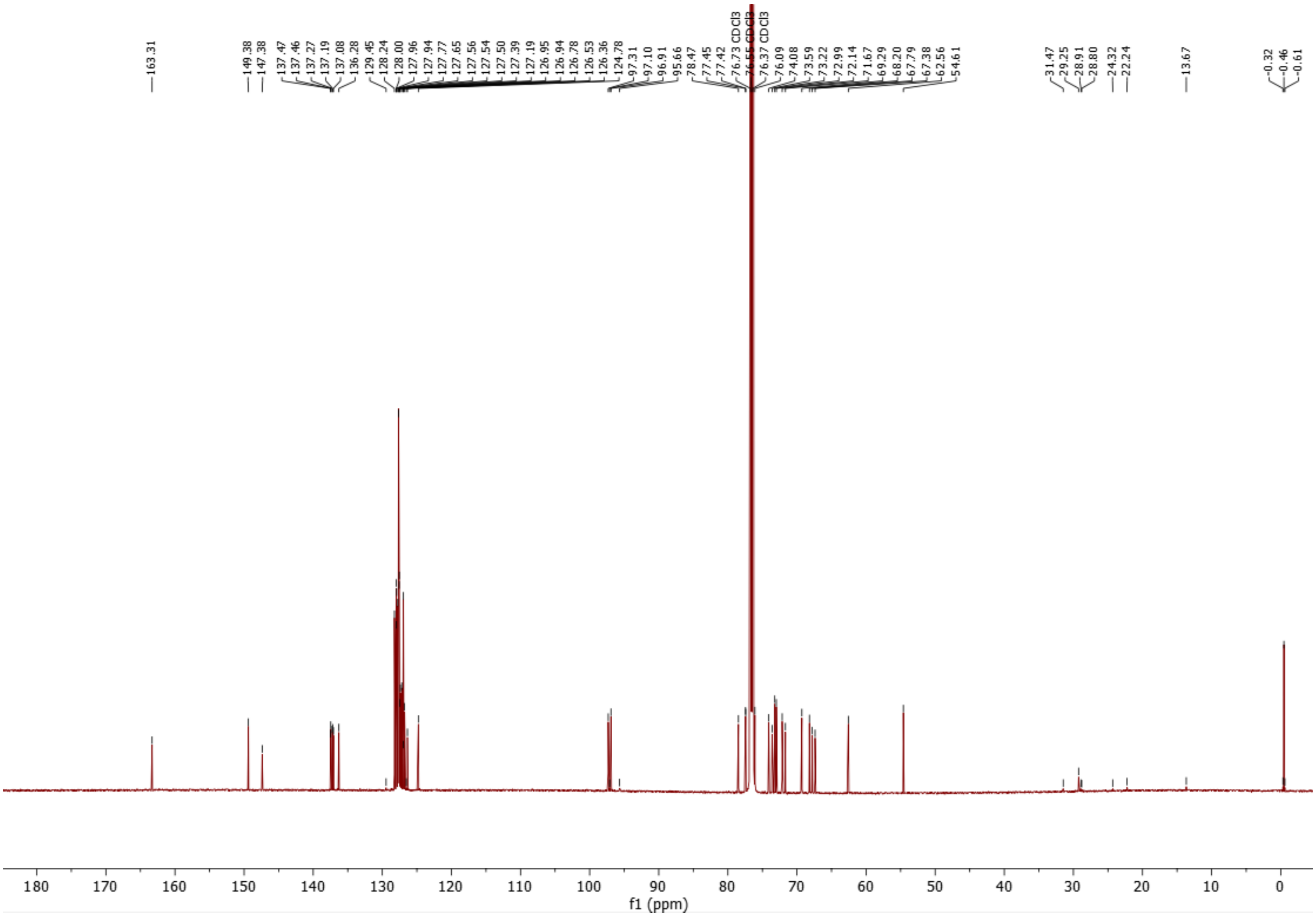

Figure S49: ^{13}C NMR (176 MHz, $CDCl_3$)

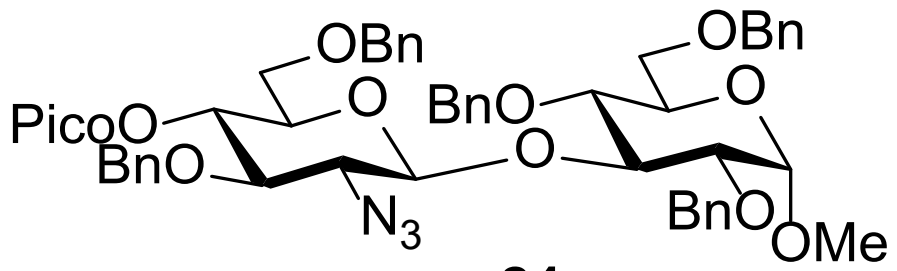

24

β-only

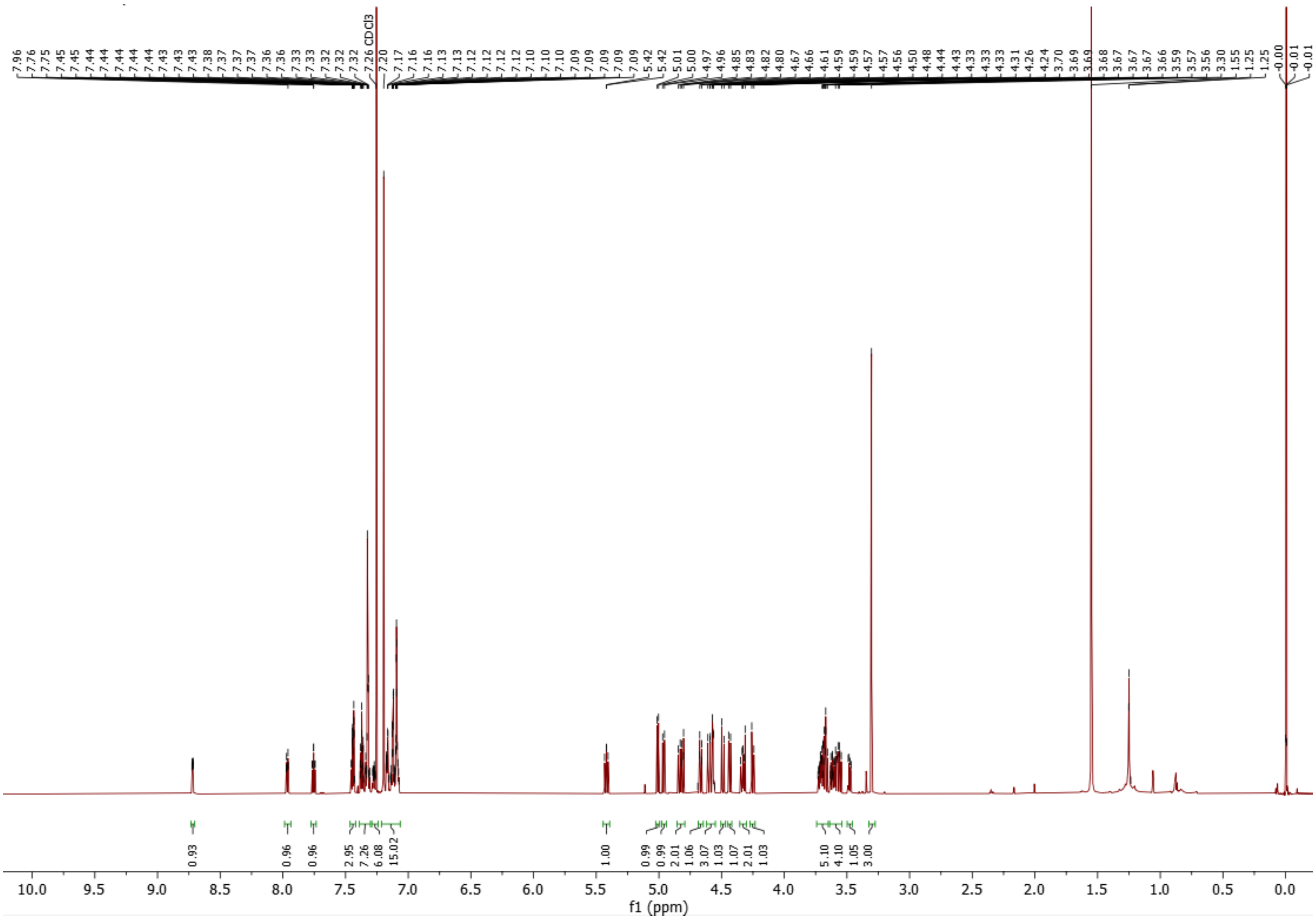

Figure S50: ^{1}H NMR (700 MHz, $CDCl_3$)

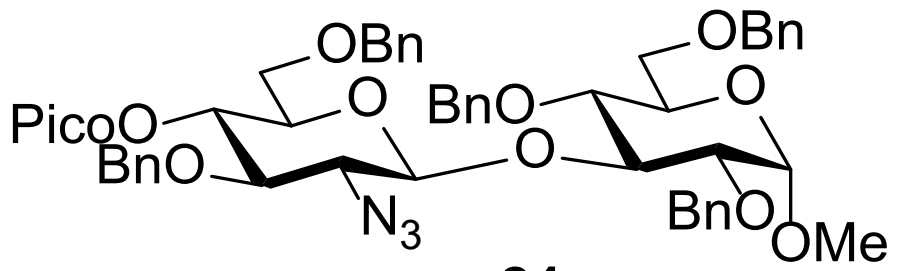

24

β-only

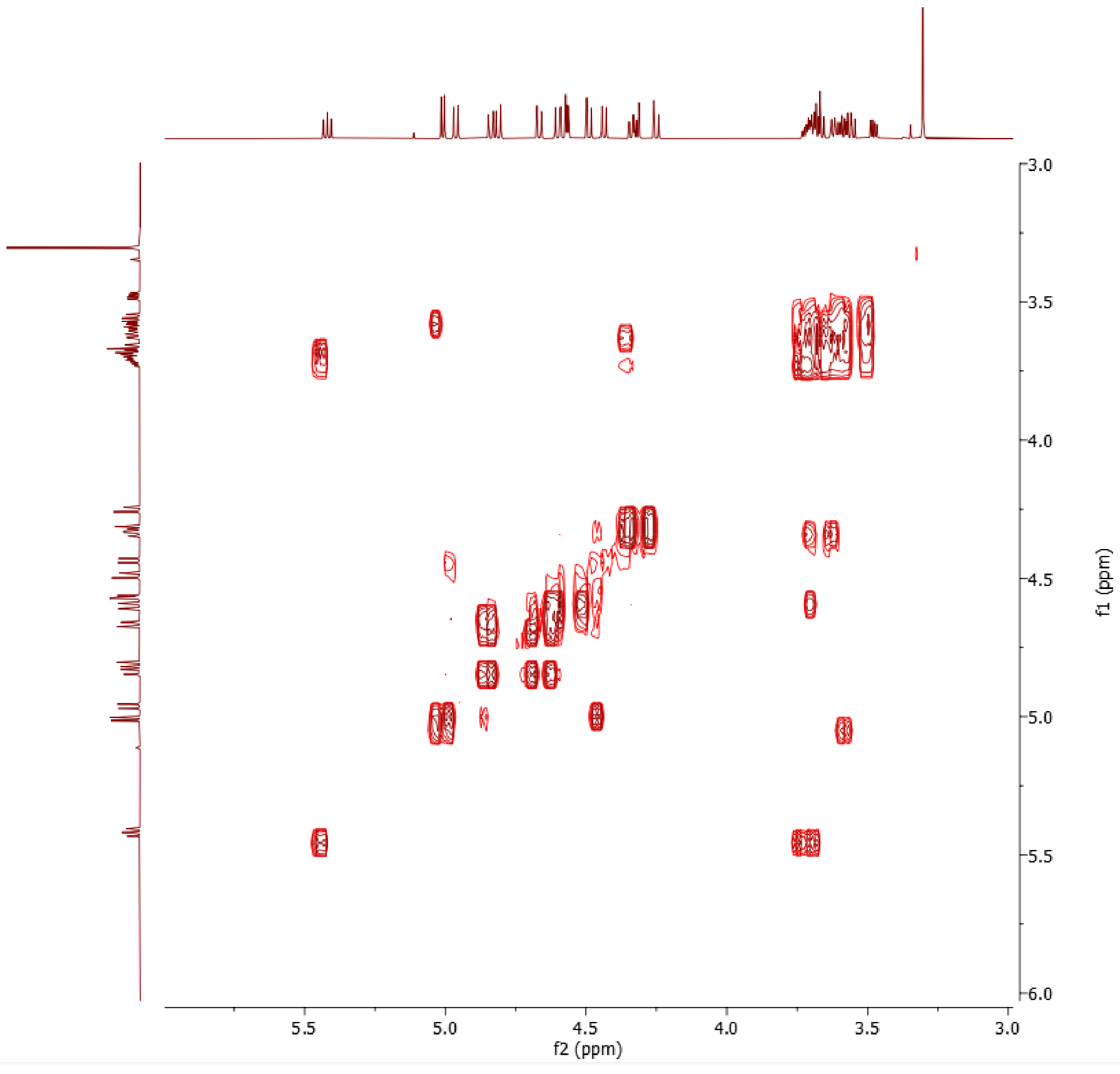

Figure S51: COSY NMR (700 MHz, $CDCl_3$)

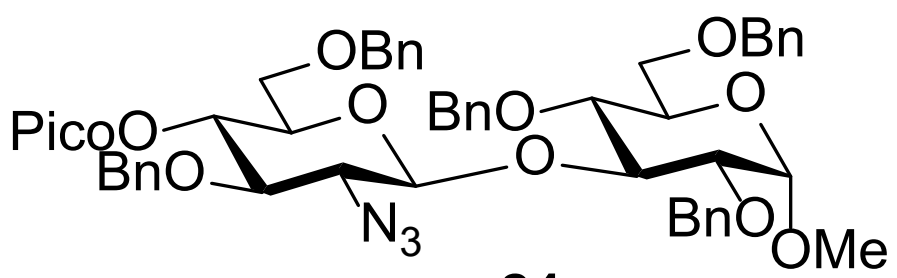

24

β-only

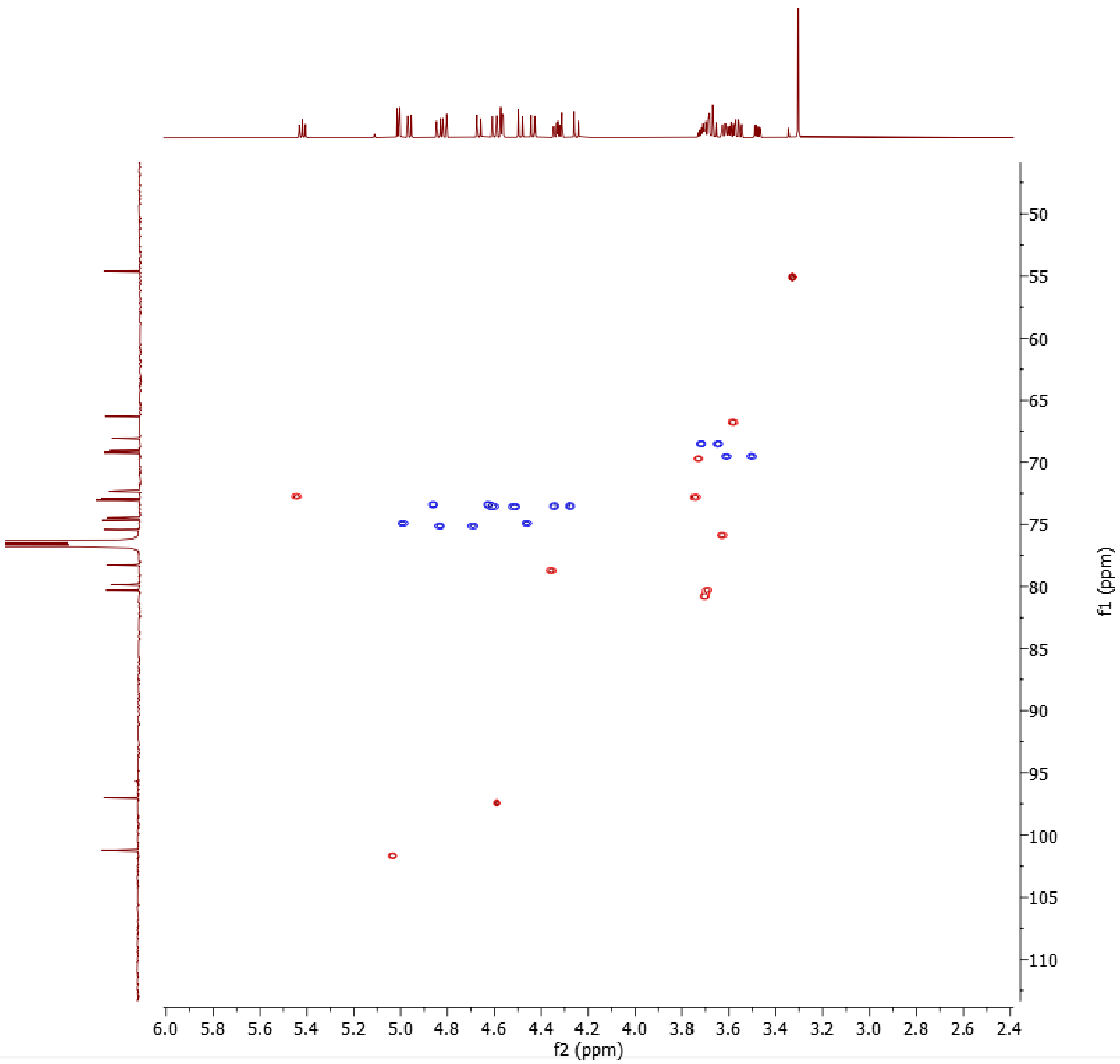

Figure S52: ^{1}H NMR and $^{13}C(^{1}H)$ NMR HSQC Spectrum (700 MHz, 176 MHz, $CDCl_3$)

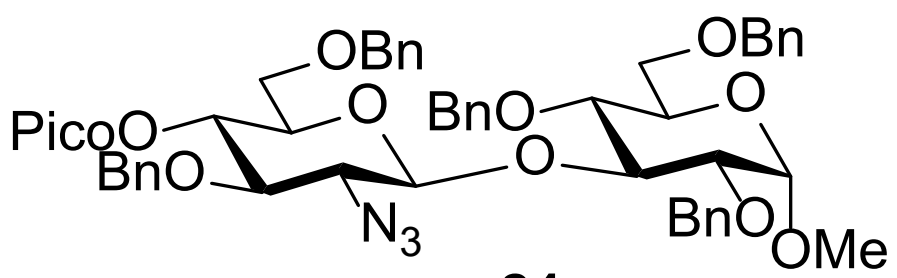

24

β-only

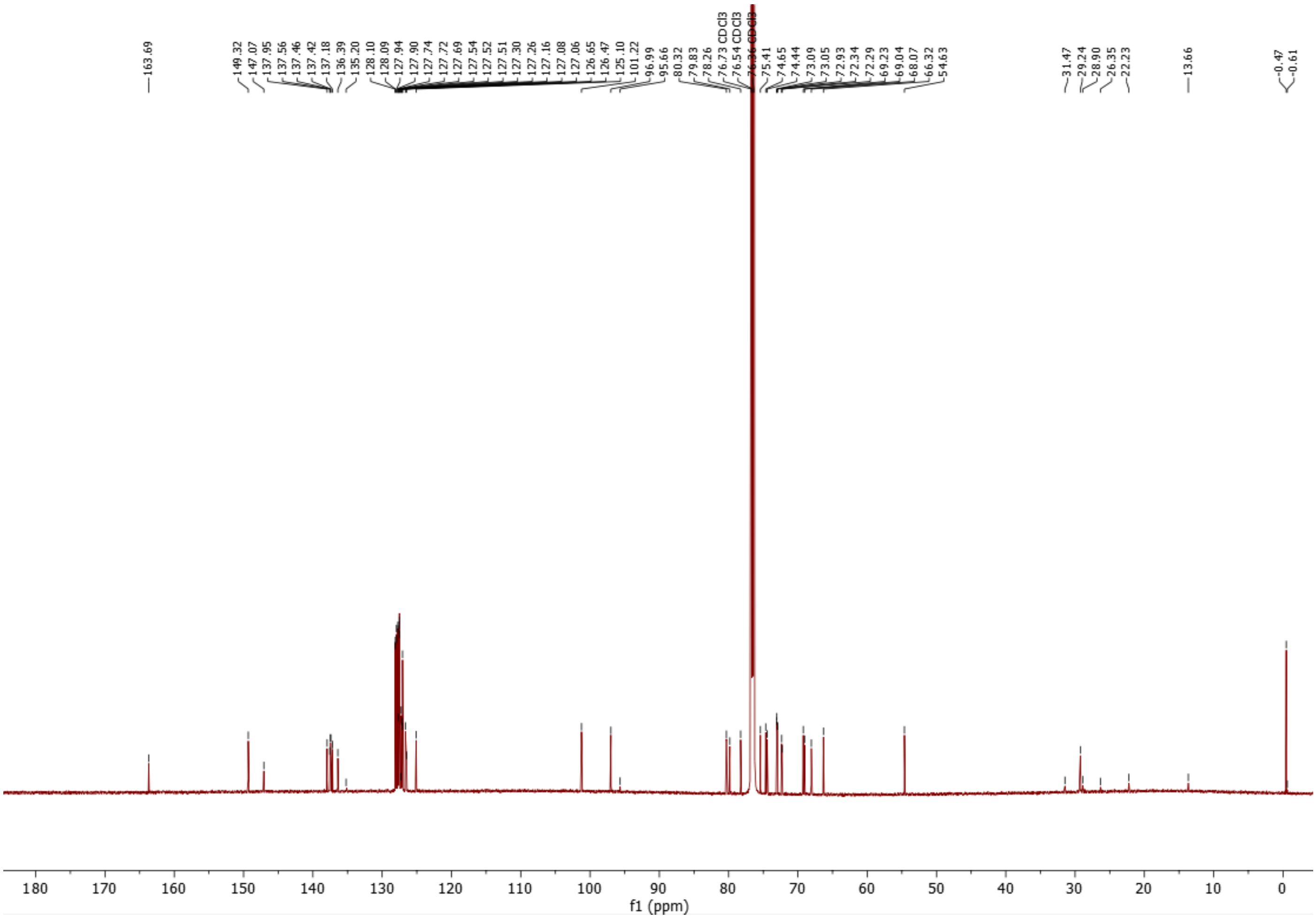

Figure S53: ^{13}C NMR (176 MHz, $CDCl_3$)

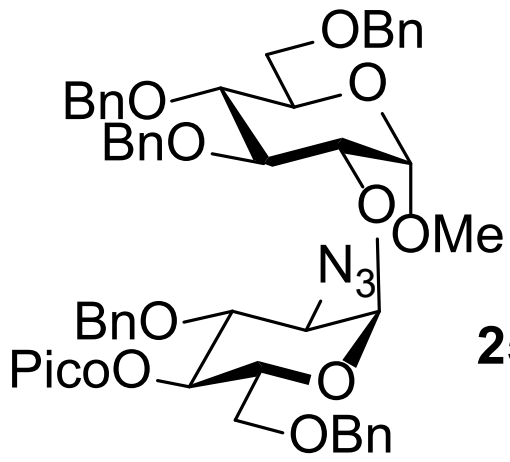

25

α-only

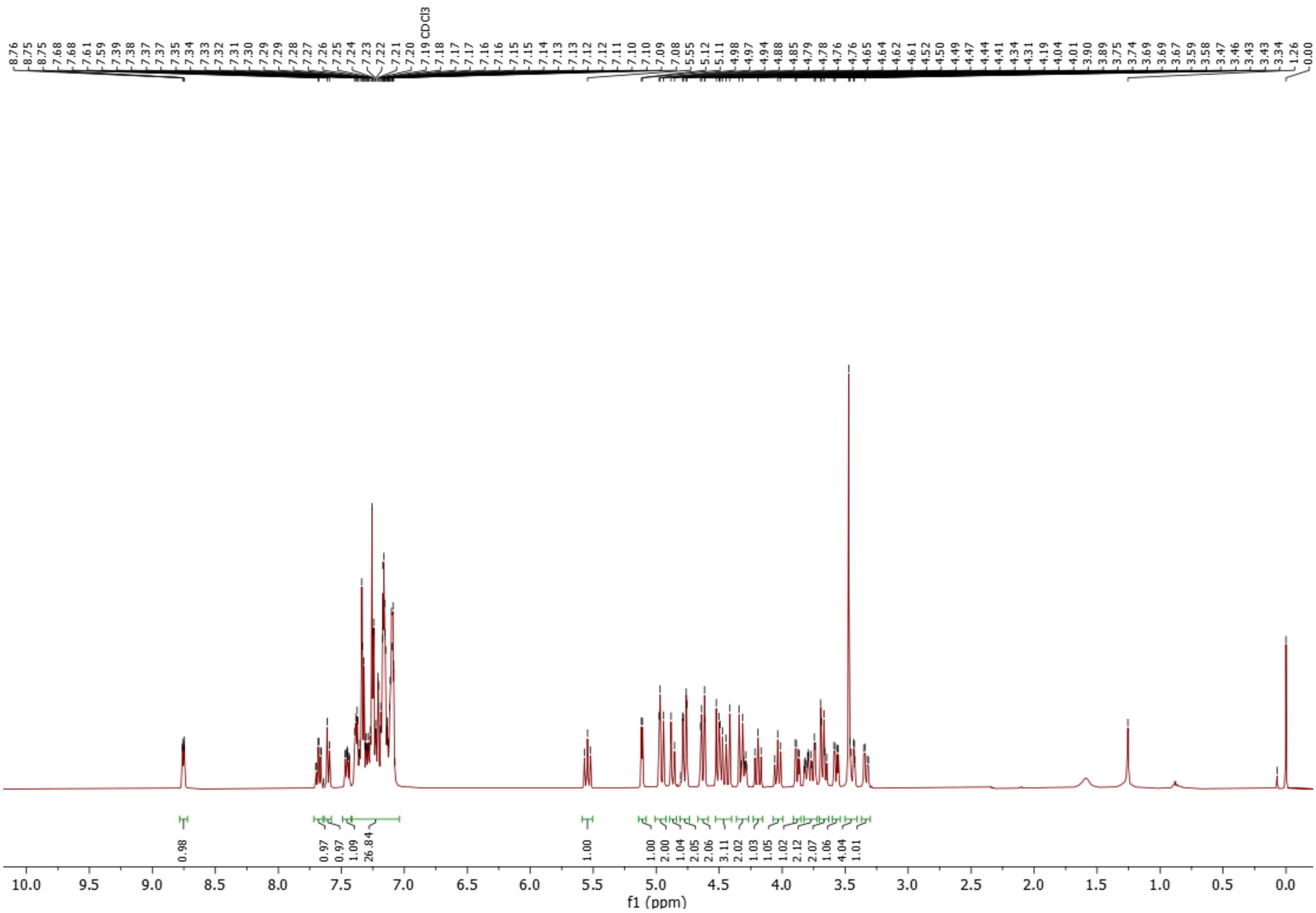

Figure S54: ^{1}H NMR (400 MHz, $CDCl_3$)

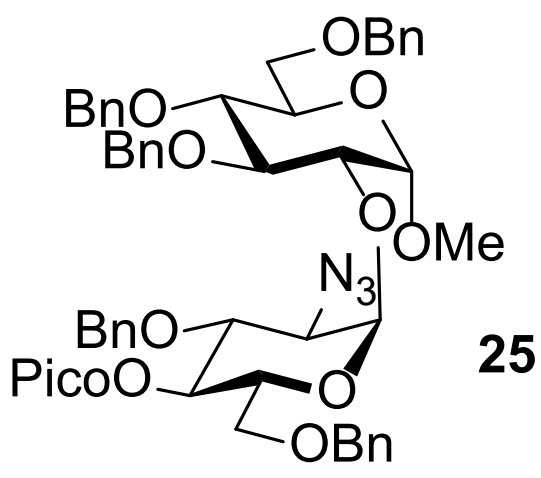

25

α-only

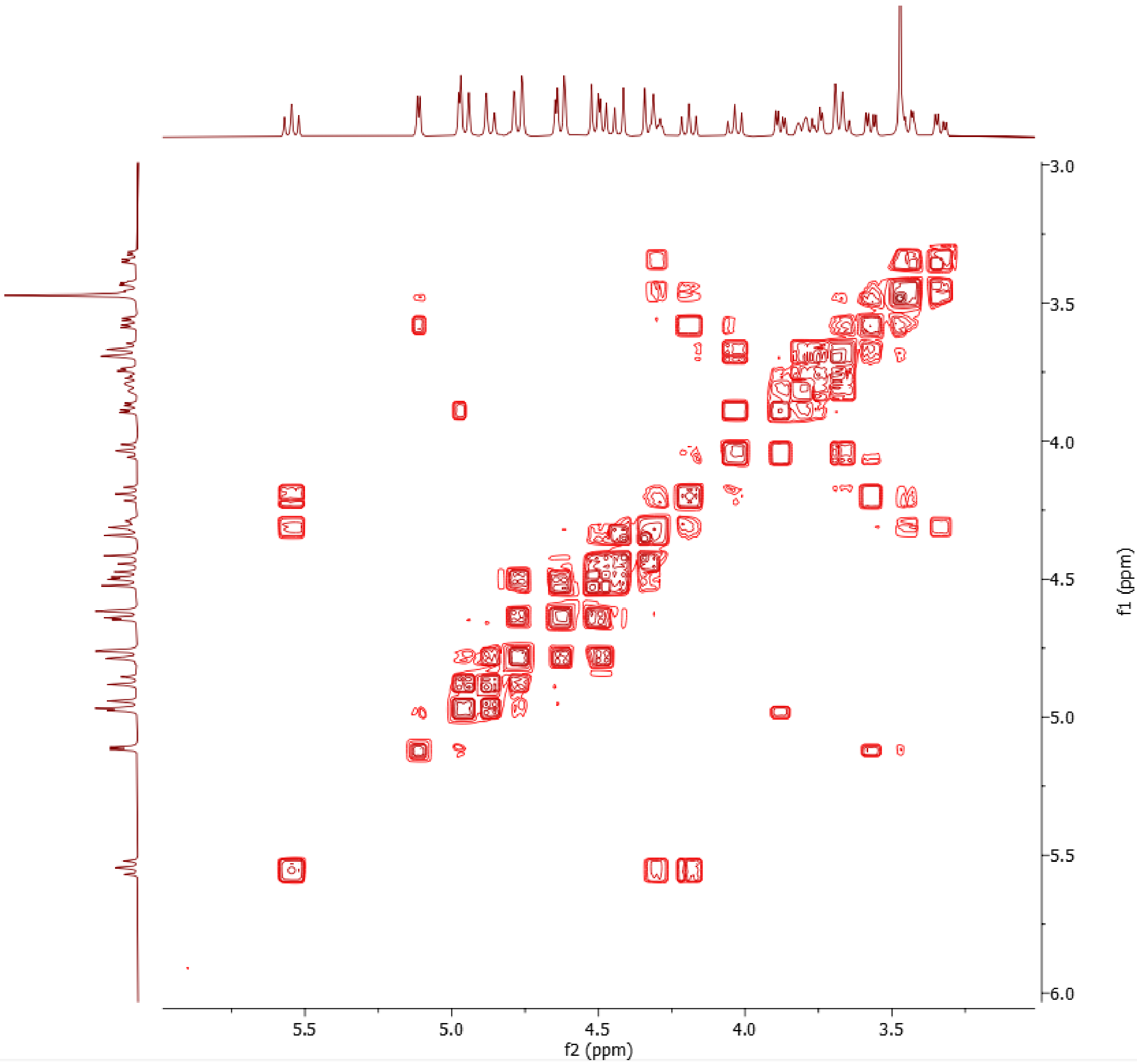

Figure S55: COSY NMR (400 MHz, $CDCl_3$)

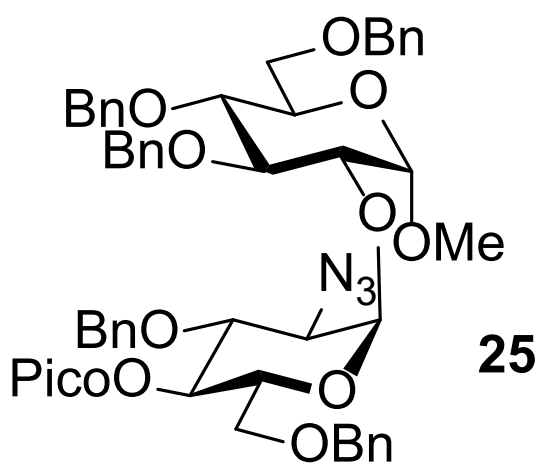

25

α-only

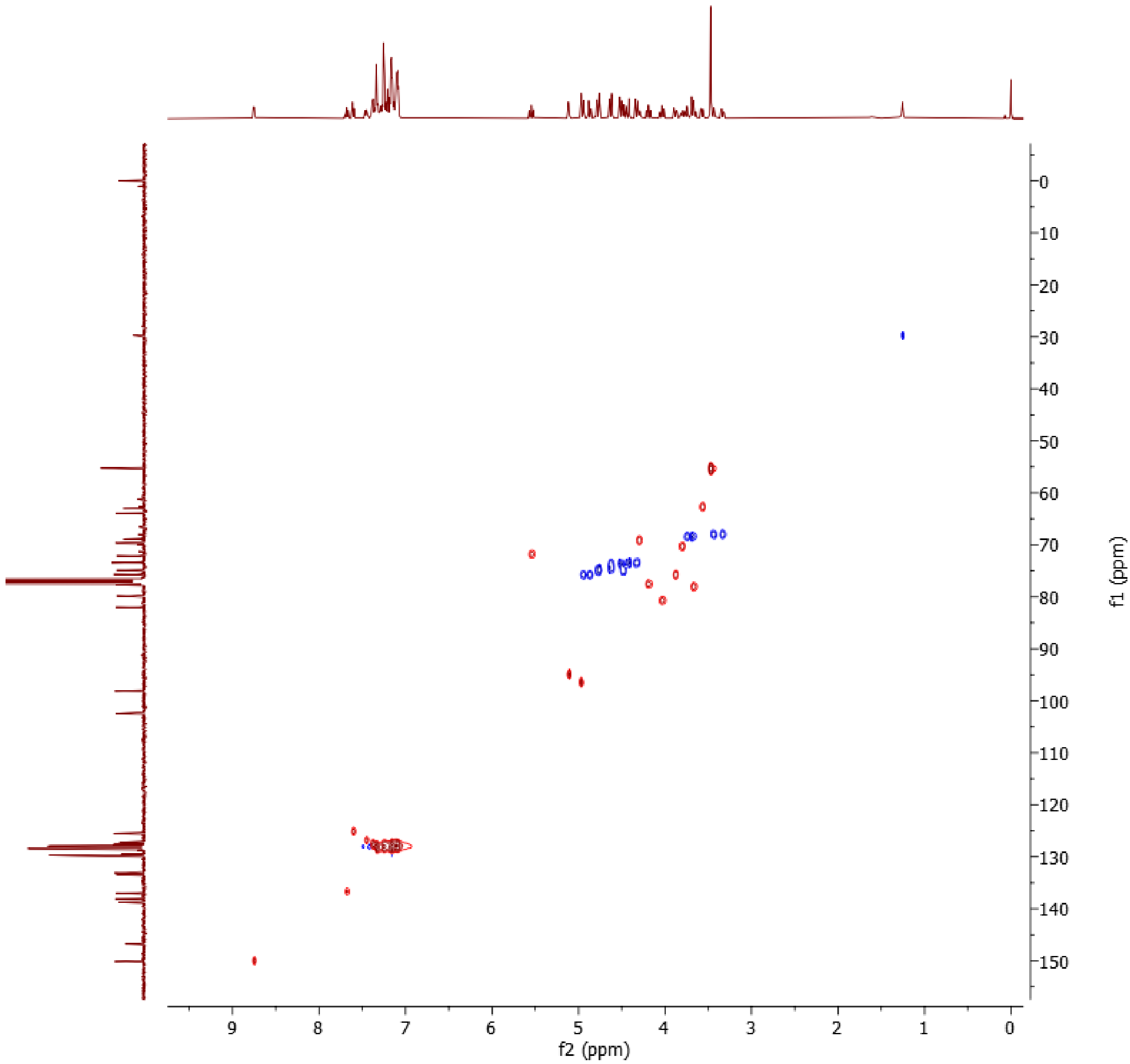

Figure S56: ^{1}H NMR and $^{13}C(^{1}H)$ NMR HSQC Spectrum (400 MHz, 101 MHz, $CDCl_3$)

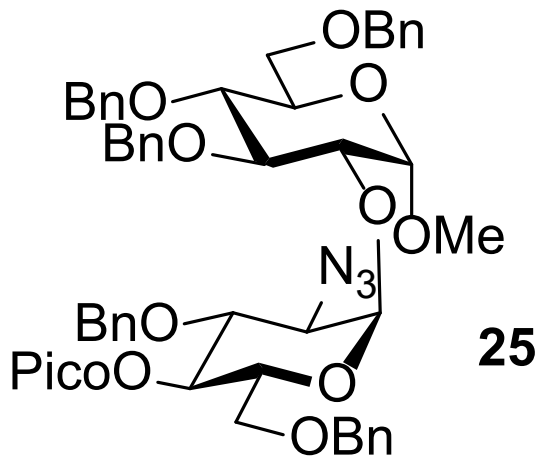

25

α-only

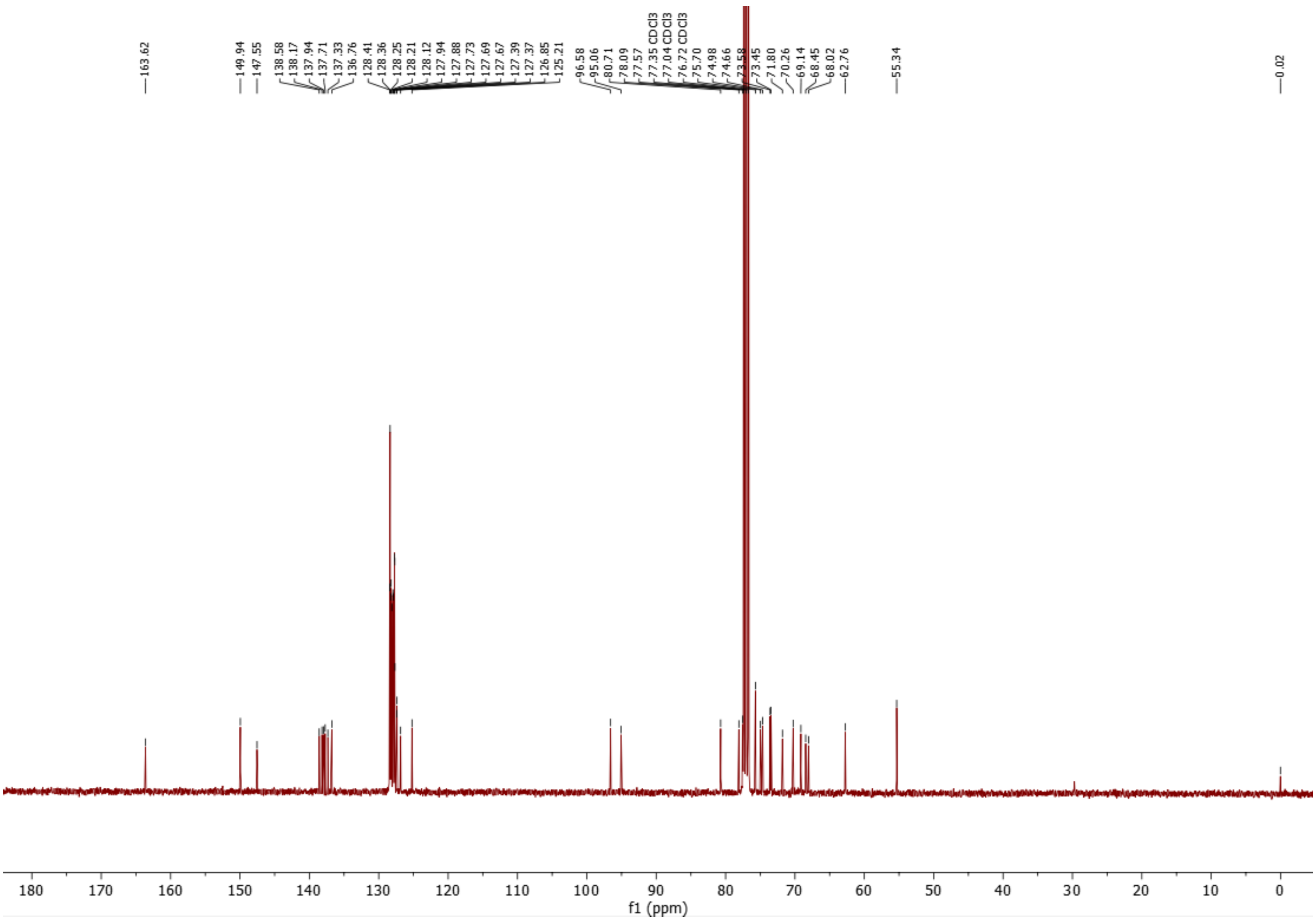

Figure S57: ^{13}C NMR (101 MHz, $CDCl_3$)

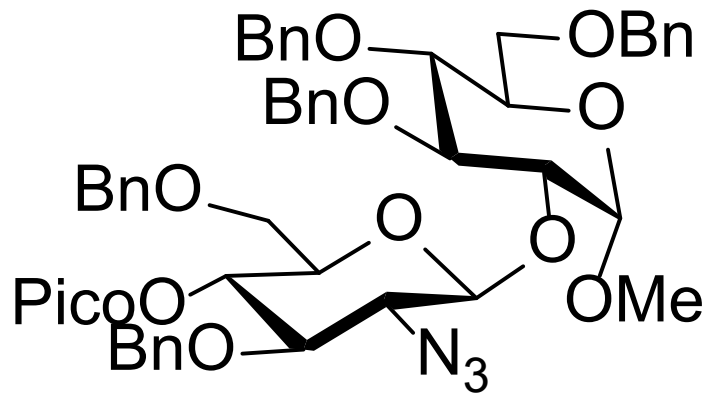

25

β-only

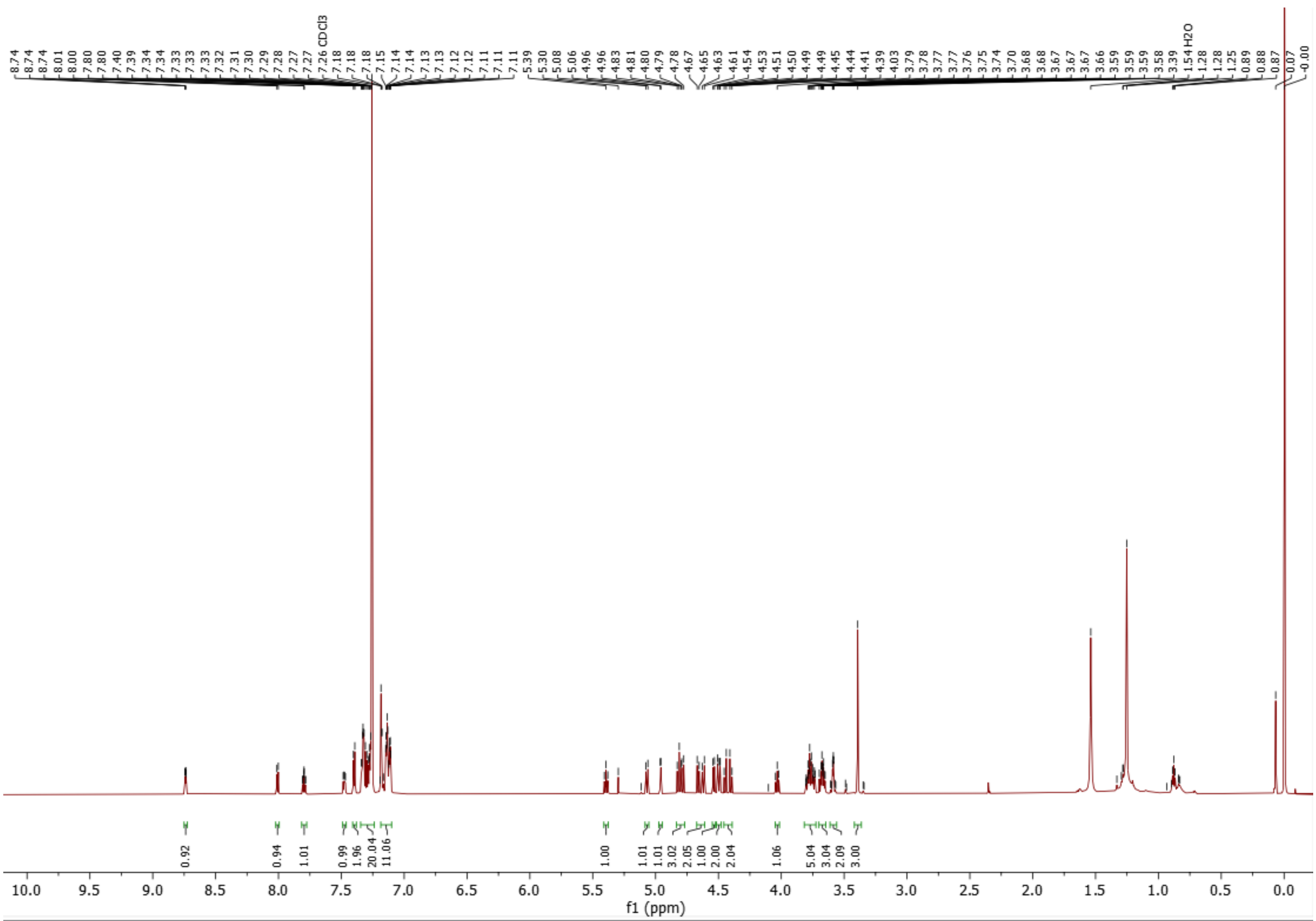

Figure S58: ^{1}H NMR (700 MHz, $CDCl_3$)

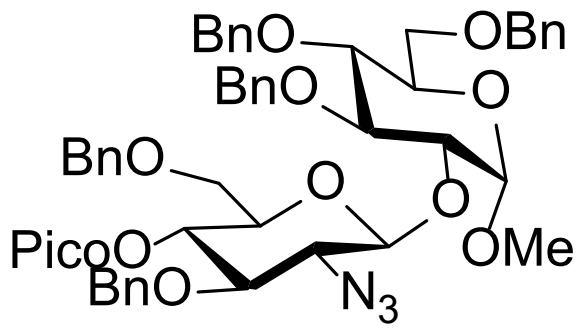

25

β-only

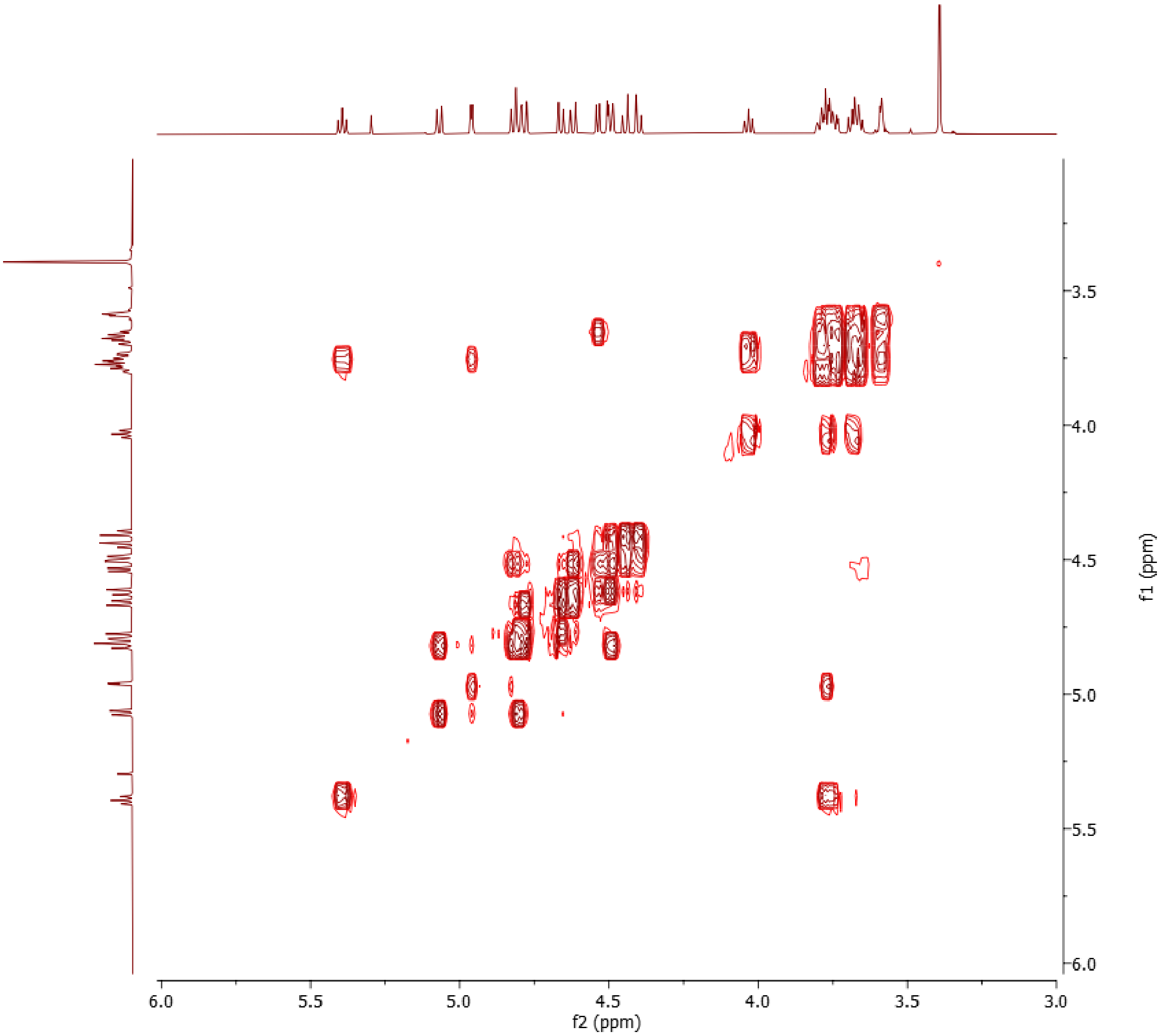

Figure S59: COSY NMR (700 MHz, $CDCl_3$)

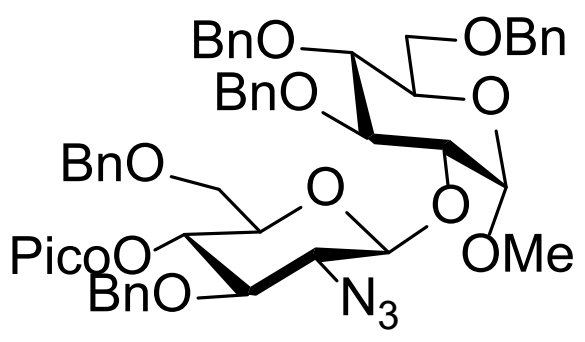

25

β-only

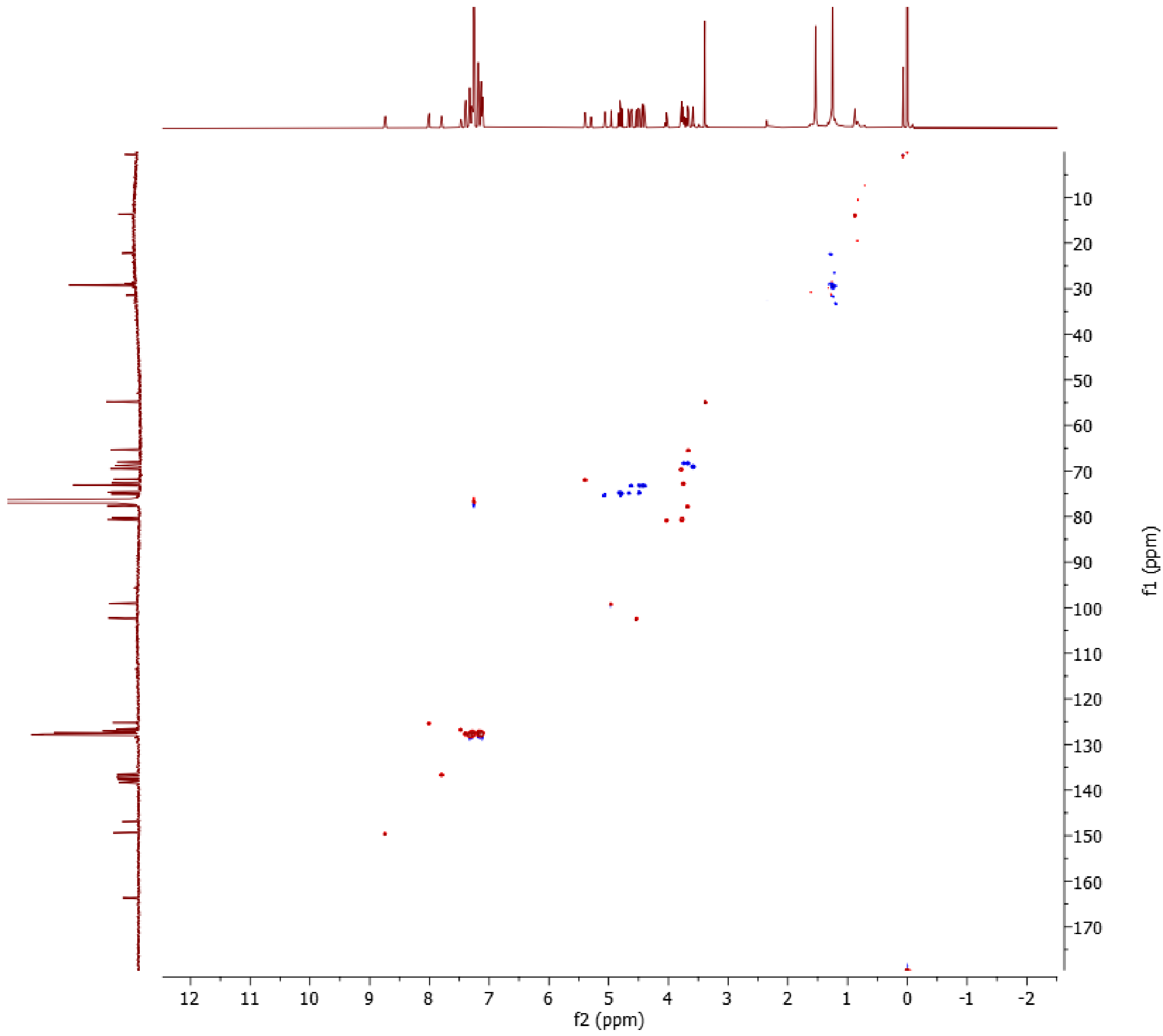

Figure S60: ^{1}H NMR and $^{13}C(^{1}H)$ NMR HSQC Spectrum (700 MHz, 176 MHz, $CDCl_3$)

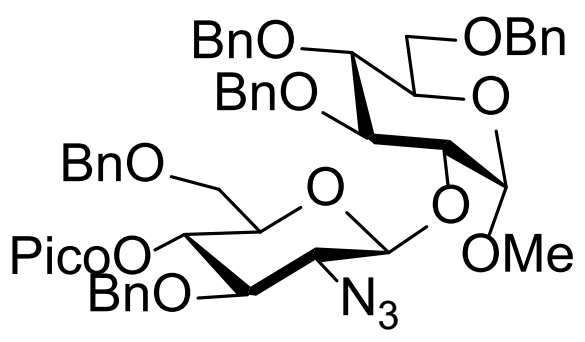

25

β-only

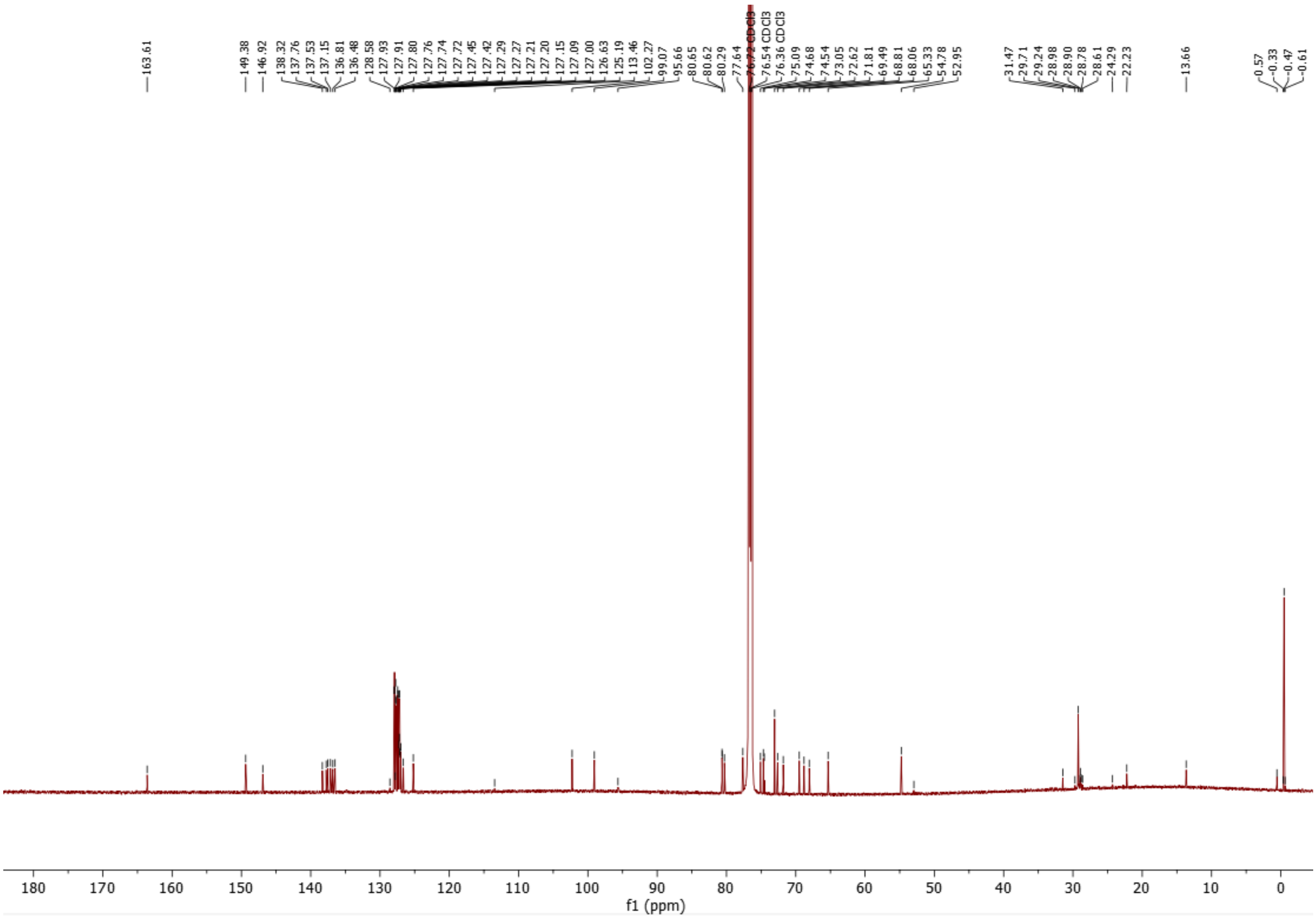

Figure S61: ^{13}C NMR (176 MHz, $CDCl_3$)

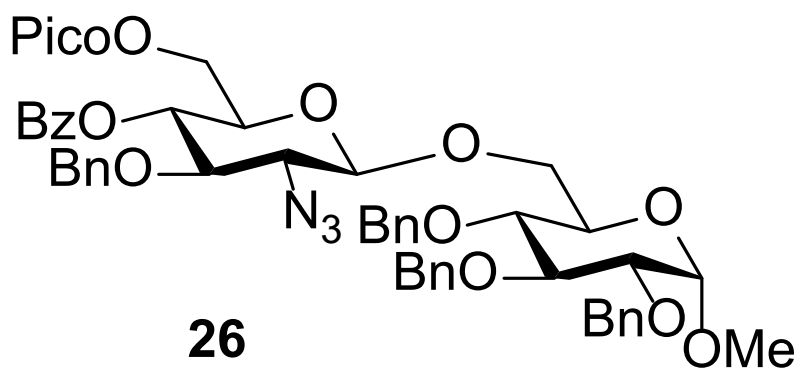

26

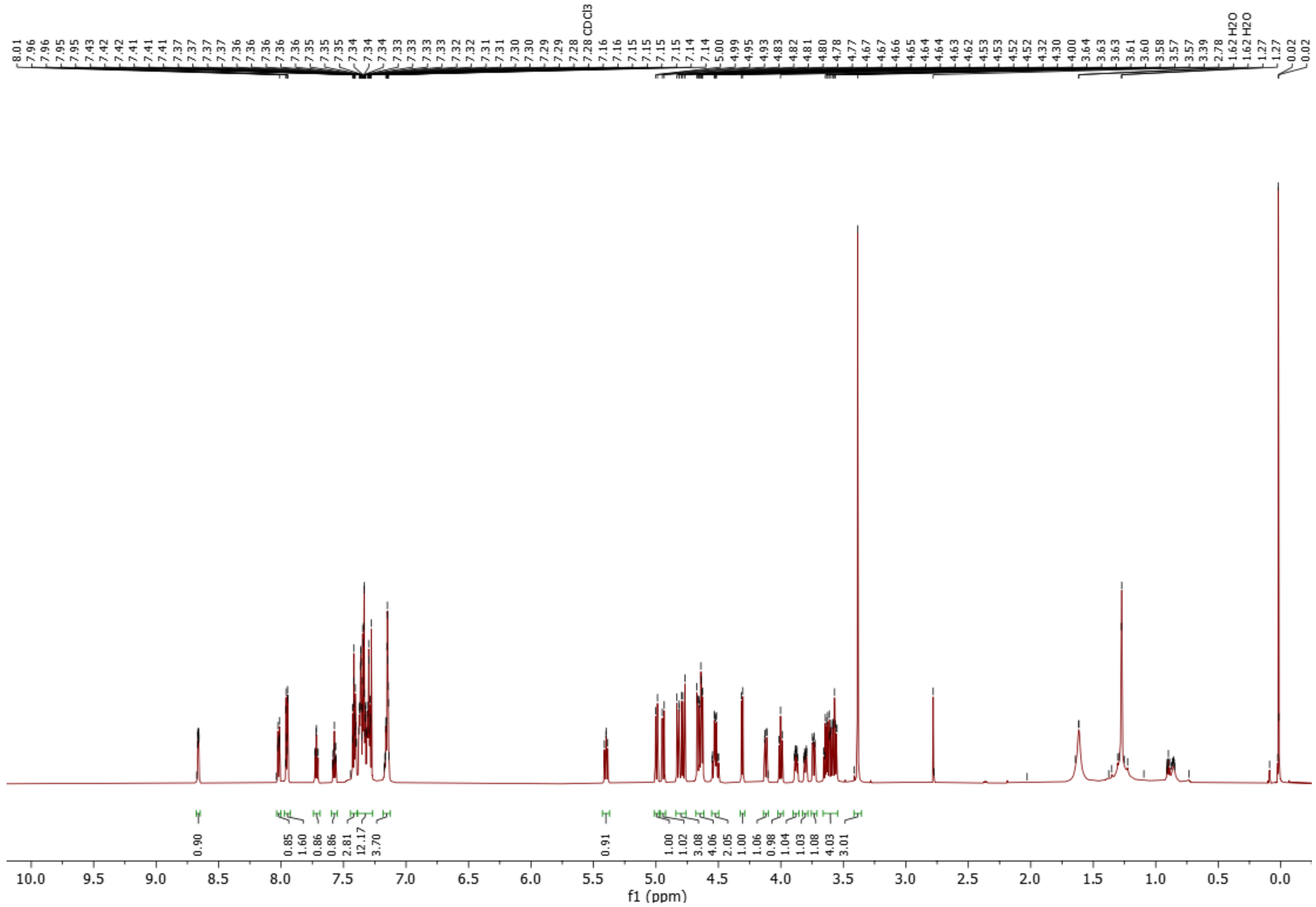

Figure S62: ^{1}H NMR (700 MHz, $CDCl_3$)

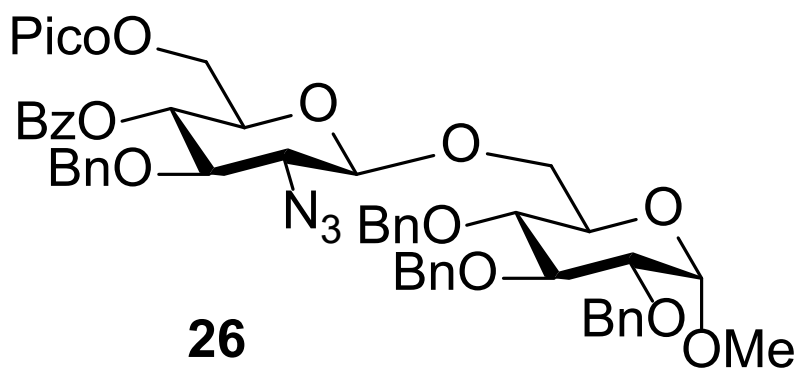

26

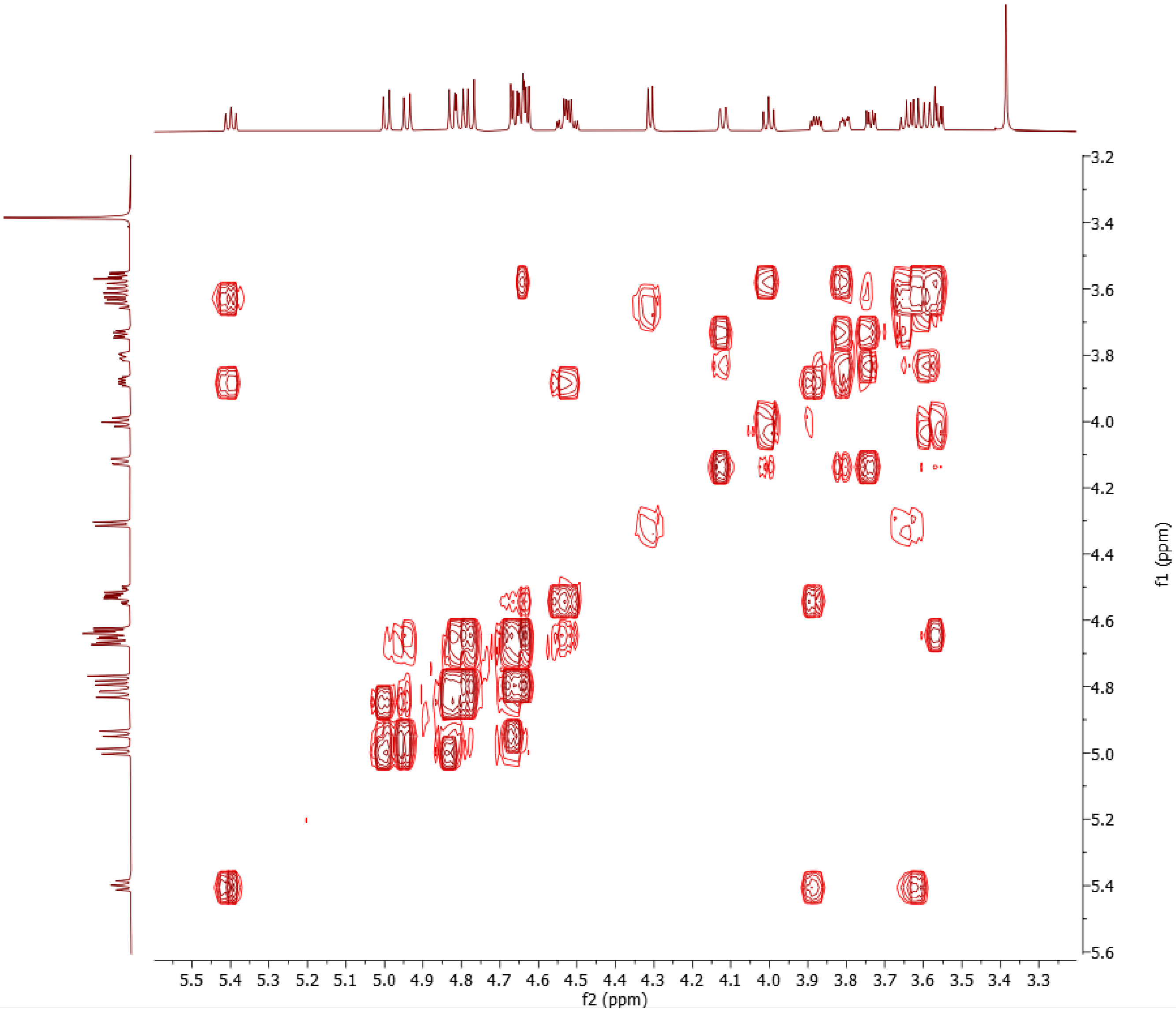

Figure S63: COSY NMR (700 MHz, $CDCl_3$)

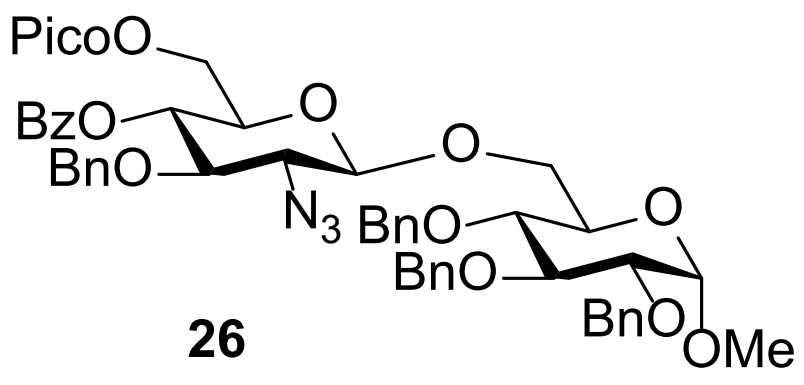

26

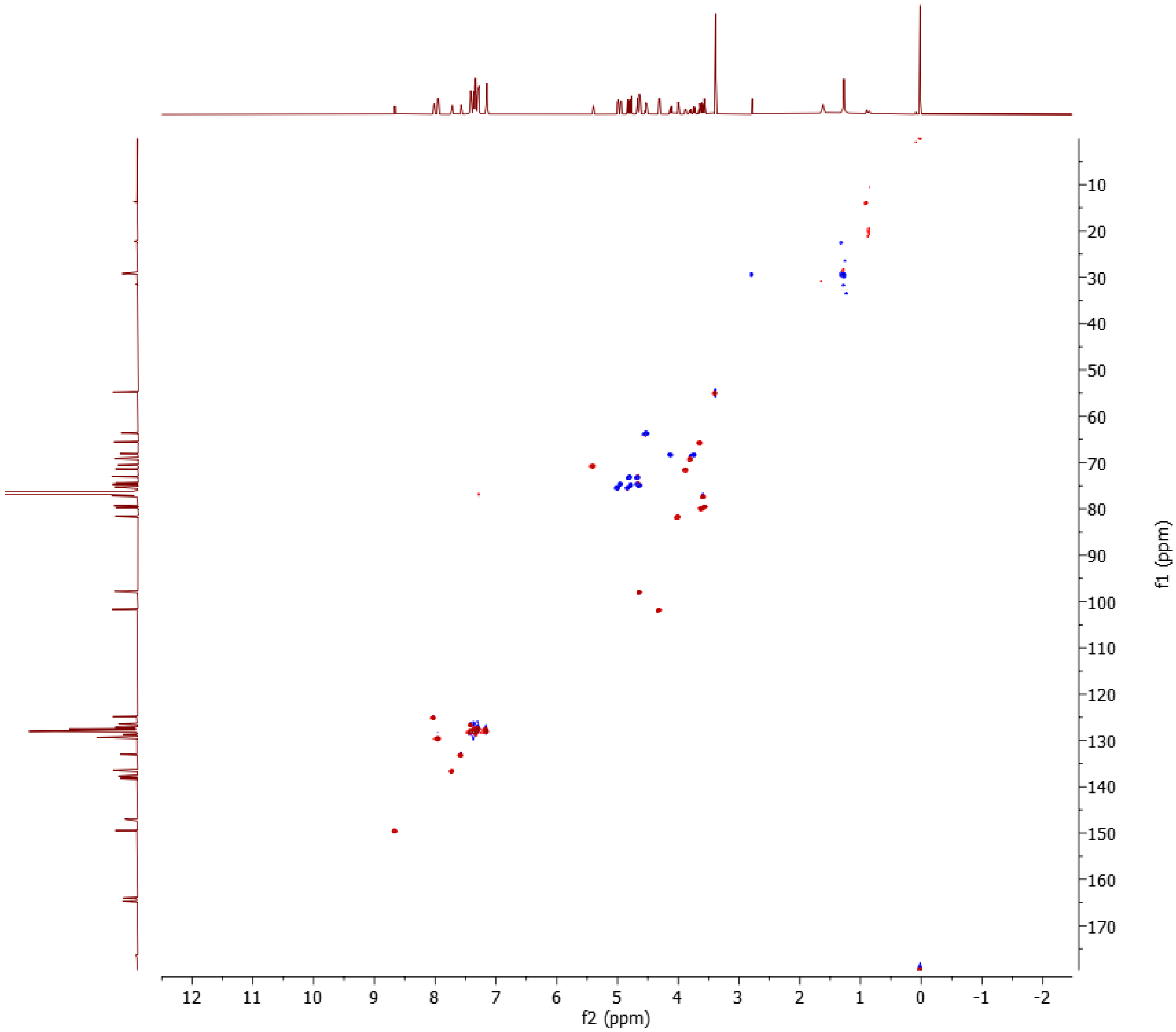

Figure S64: ^{1}H NMR and $^{13}C(^{1}H)$ NMR HSQC Spectrum (700 MHz, 176 MHz, $CDCl_3$)

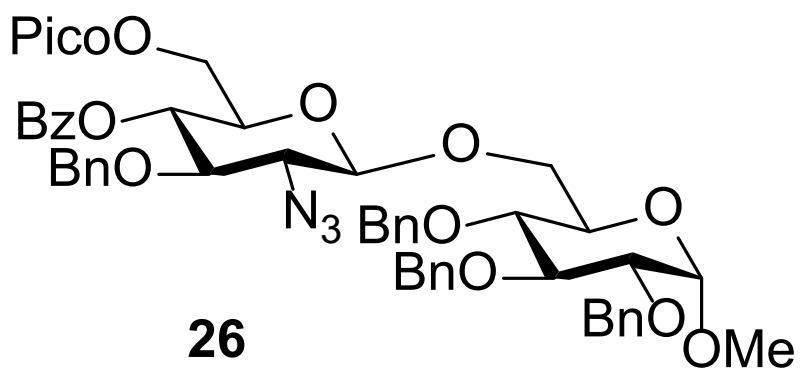

26

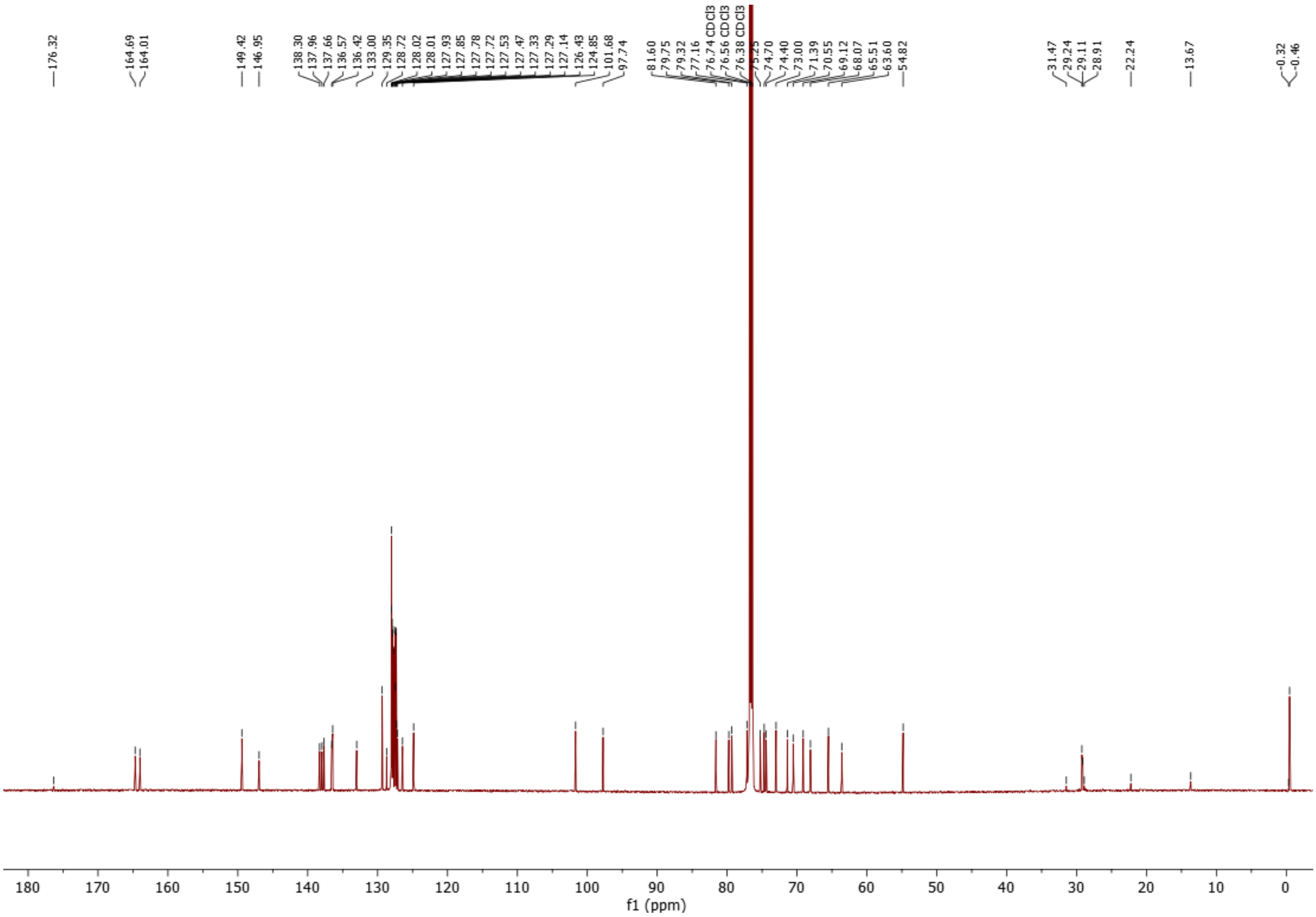

Figure S65: ^{13}C NMR (176 MHz, $CDCl_3$)

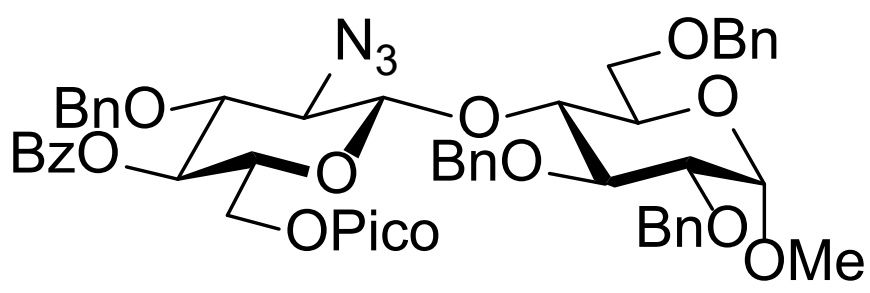

27

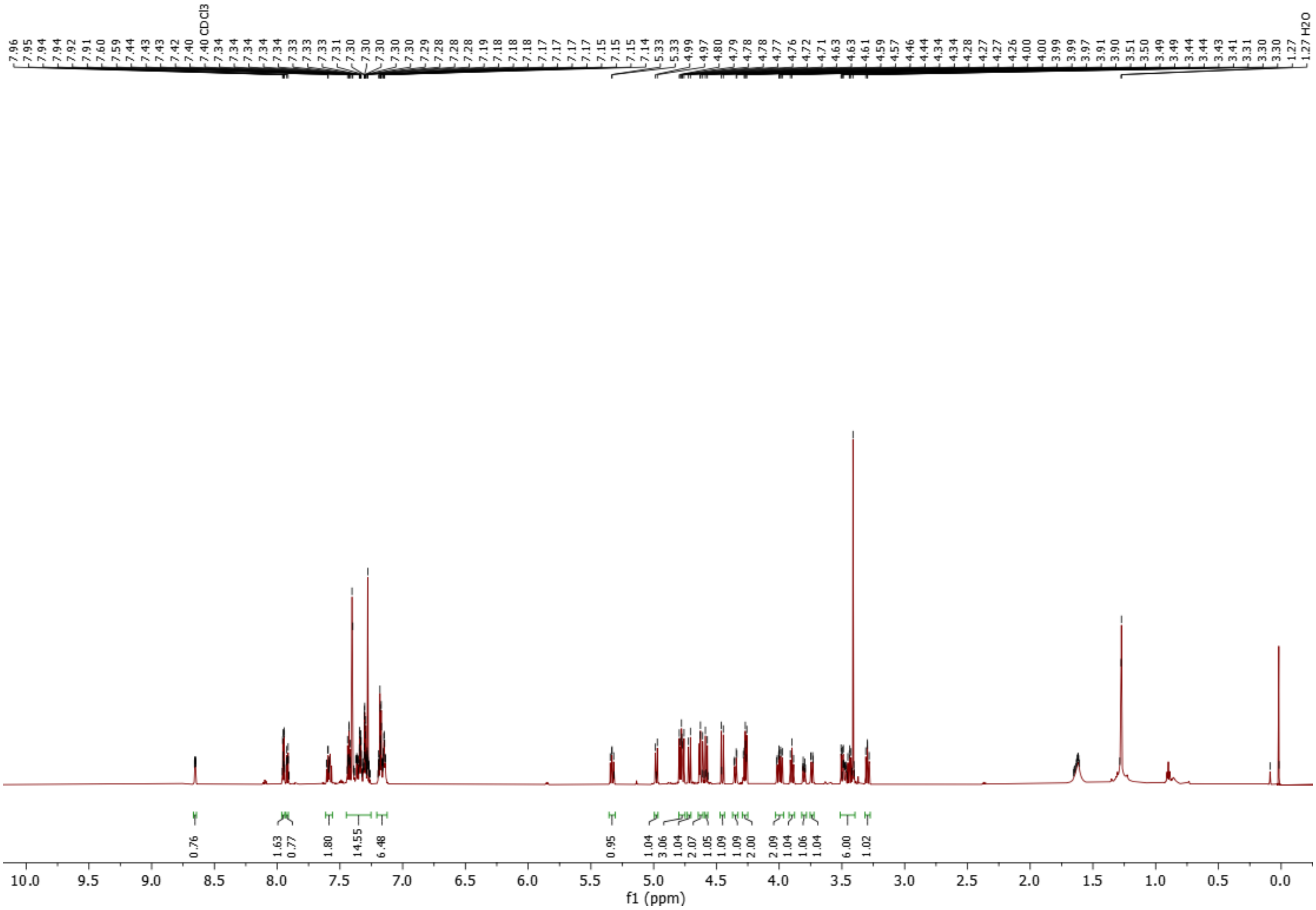

Figure S66: ^{1}H NMR (700 MHz, $CDCl_3$)

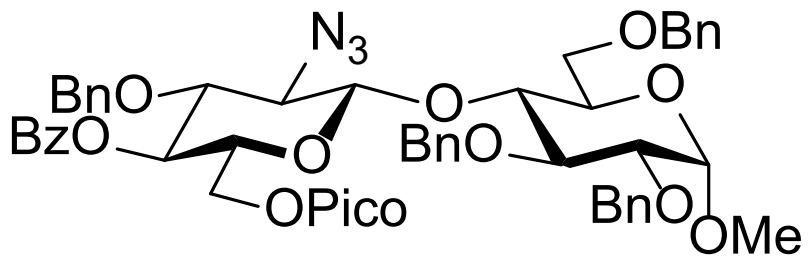

27

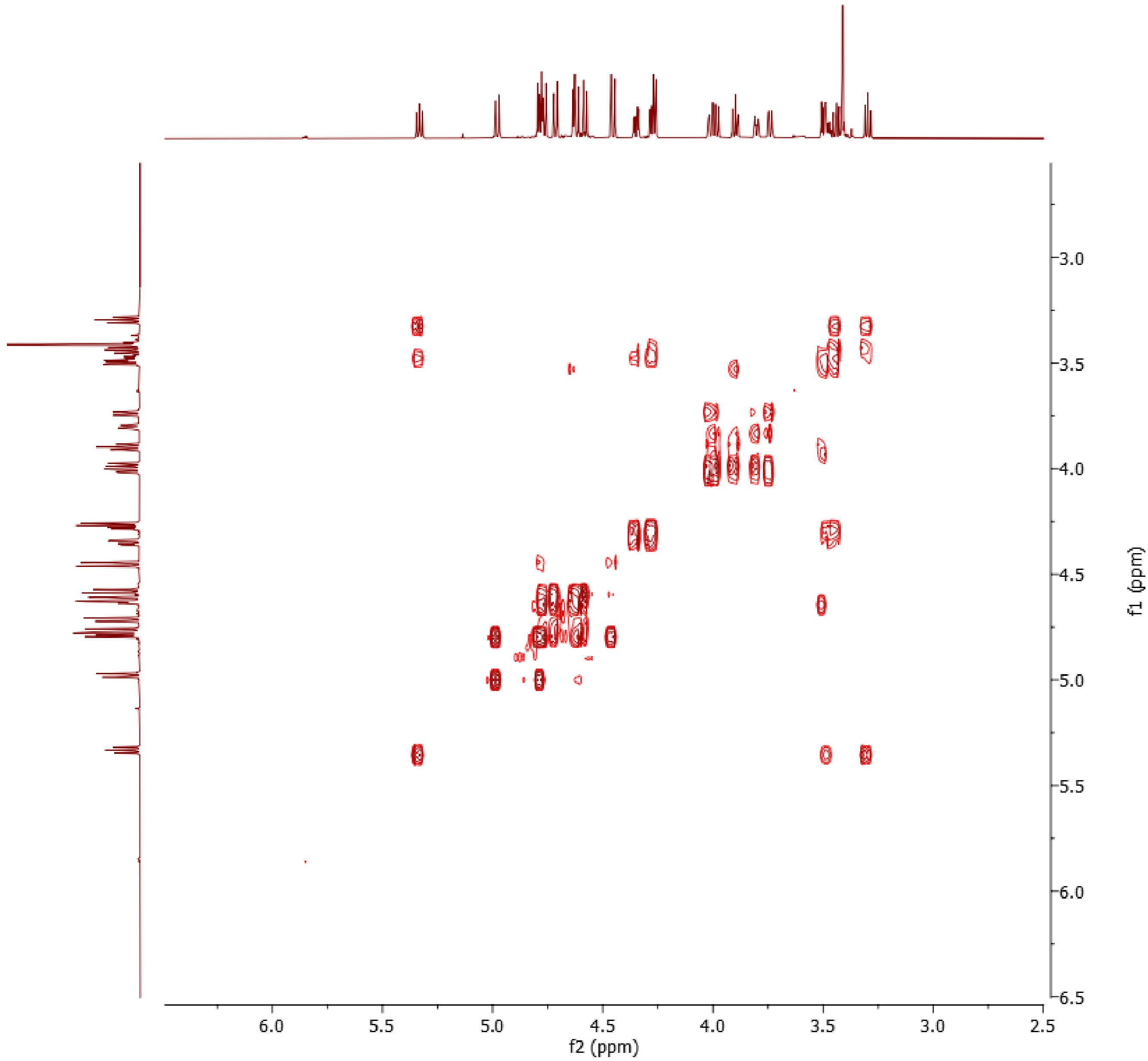

Figure S67: COSY NMR (700 MHz, $CDCl_3$)

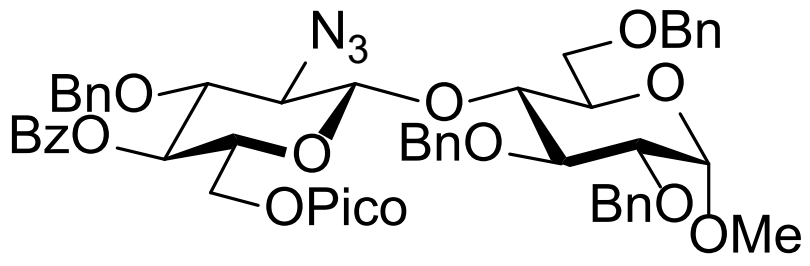

27

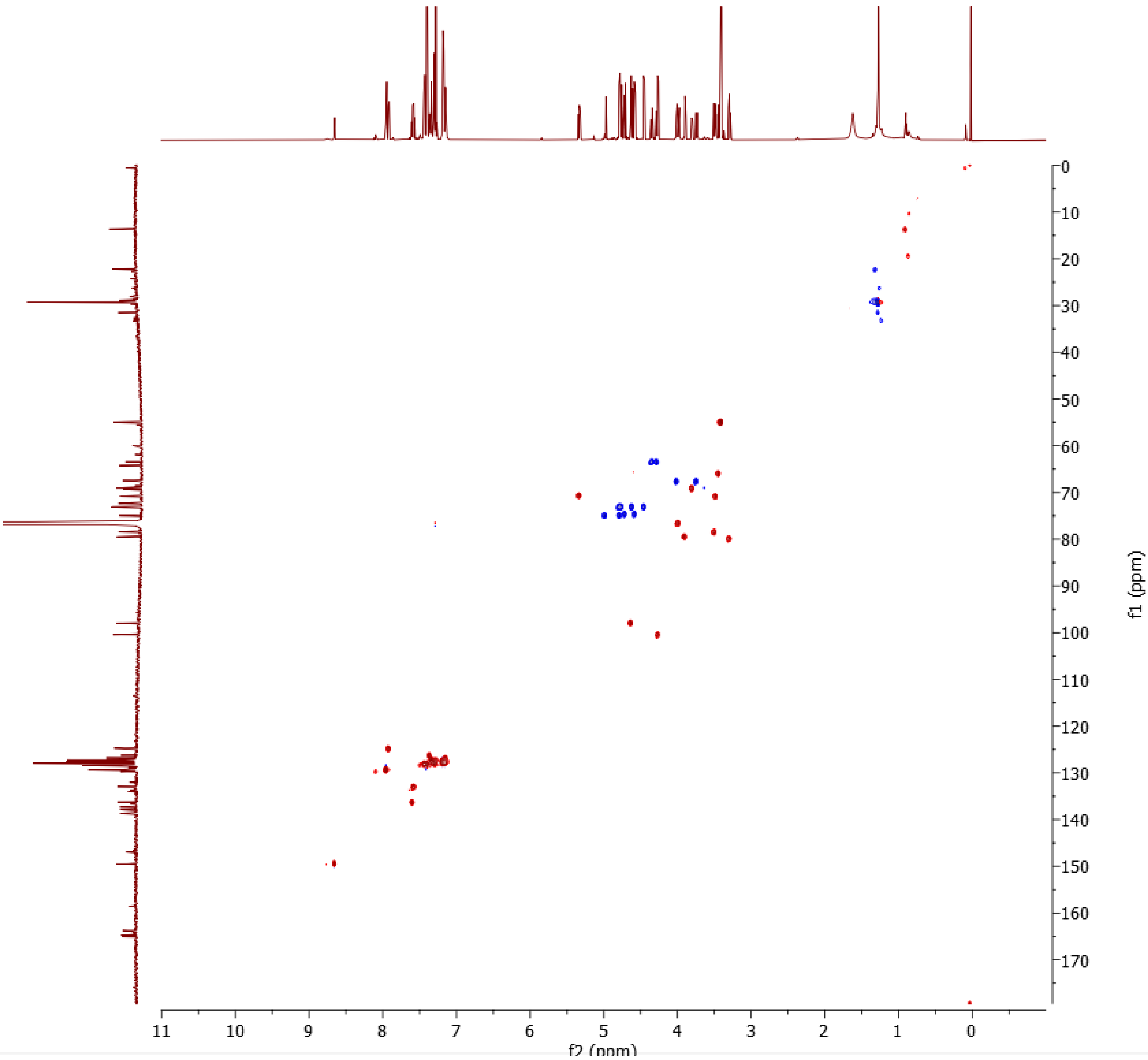

Figure S68: ^{1}H NMR and $^{13}C(^{1}H)$ NMR HSQC Spectrum (700 MHz, 176 MHz, $CDCl_3$)

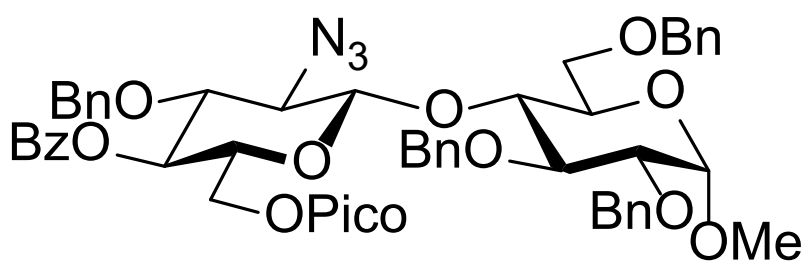

27

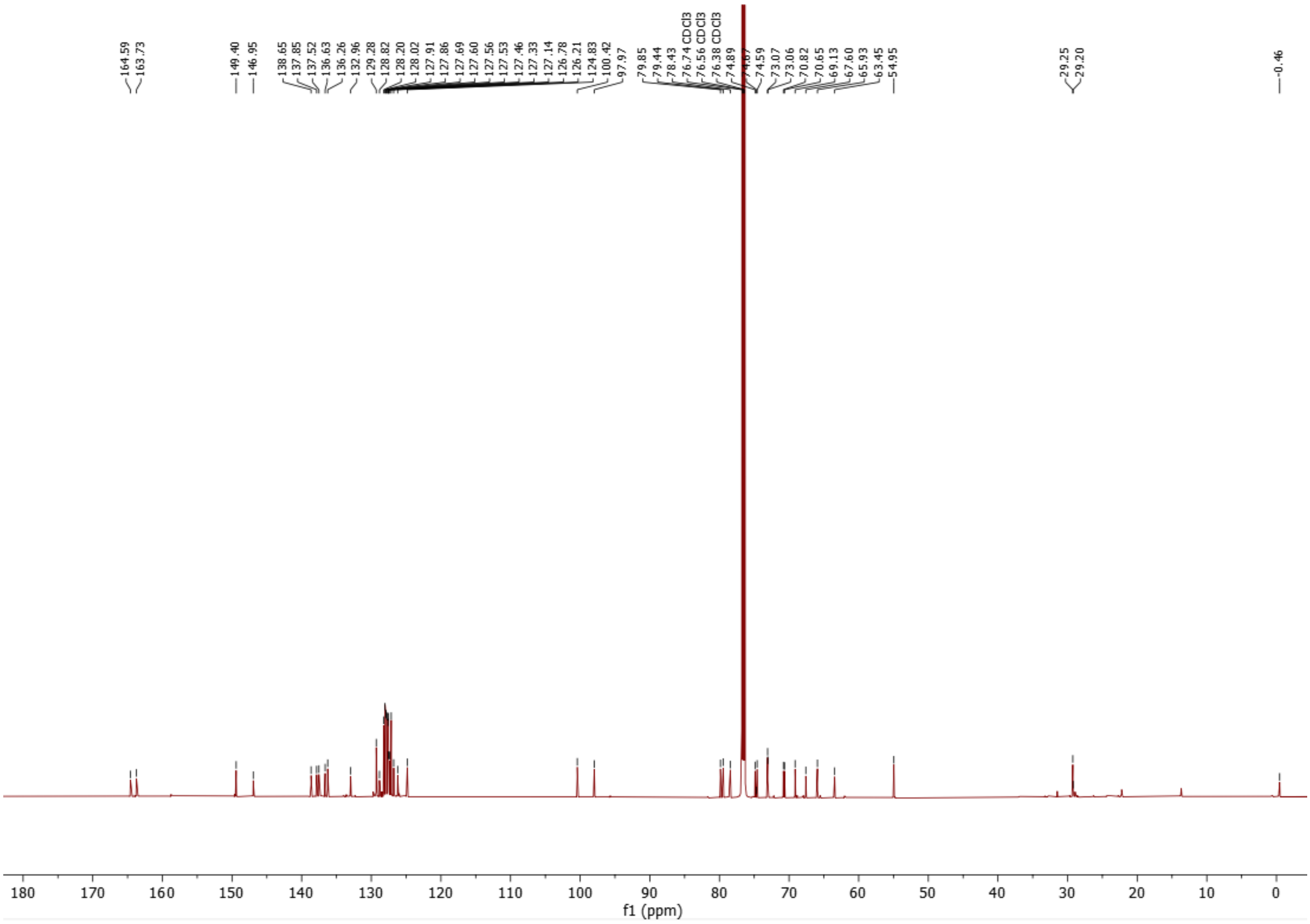

Figure S69: ^{13}C NMR (176 MHz, CDCl$_3$)

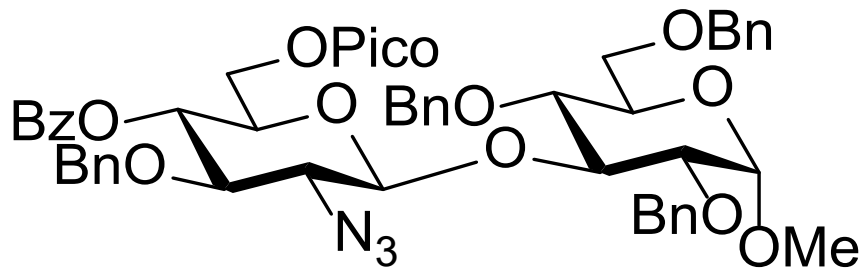

28

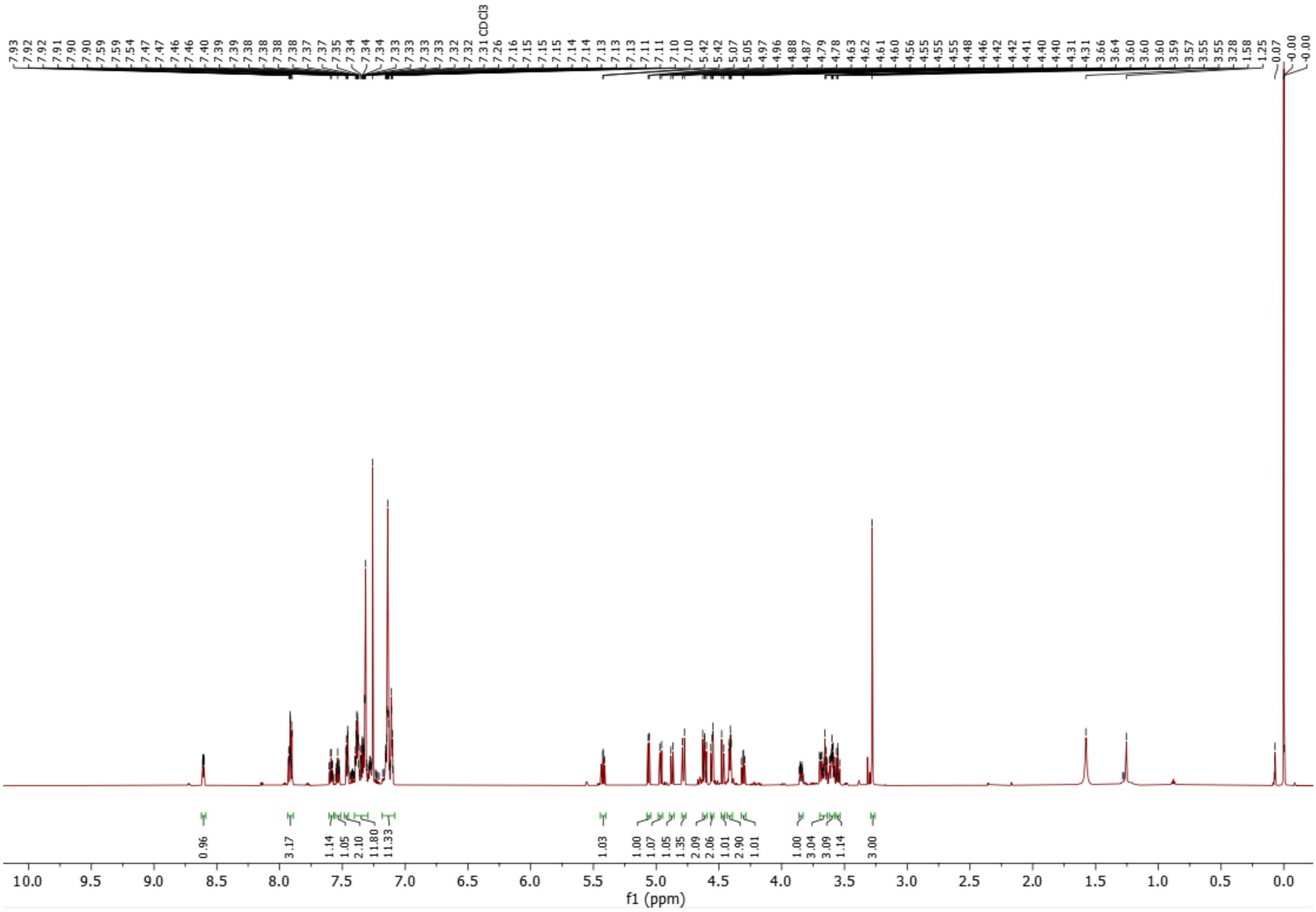

Figure S70: ^{1}H NMR (700 MHz, $CDCl_3$)

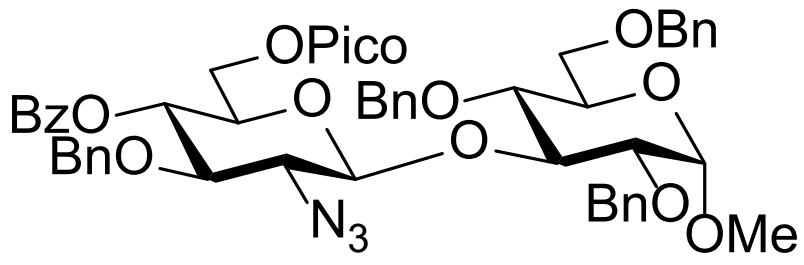

28

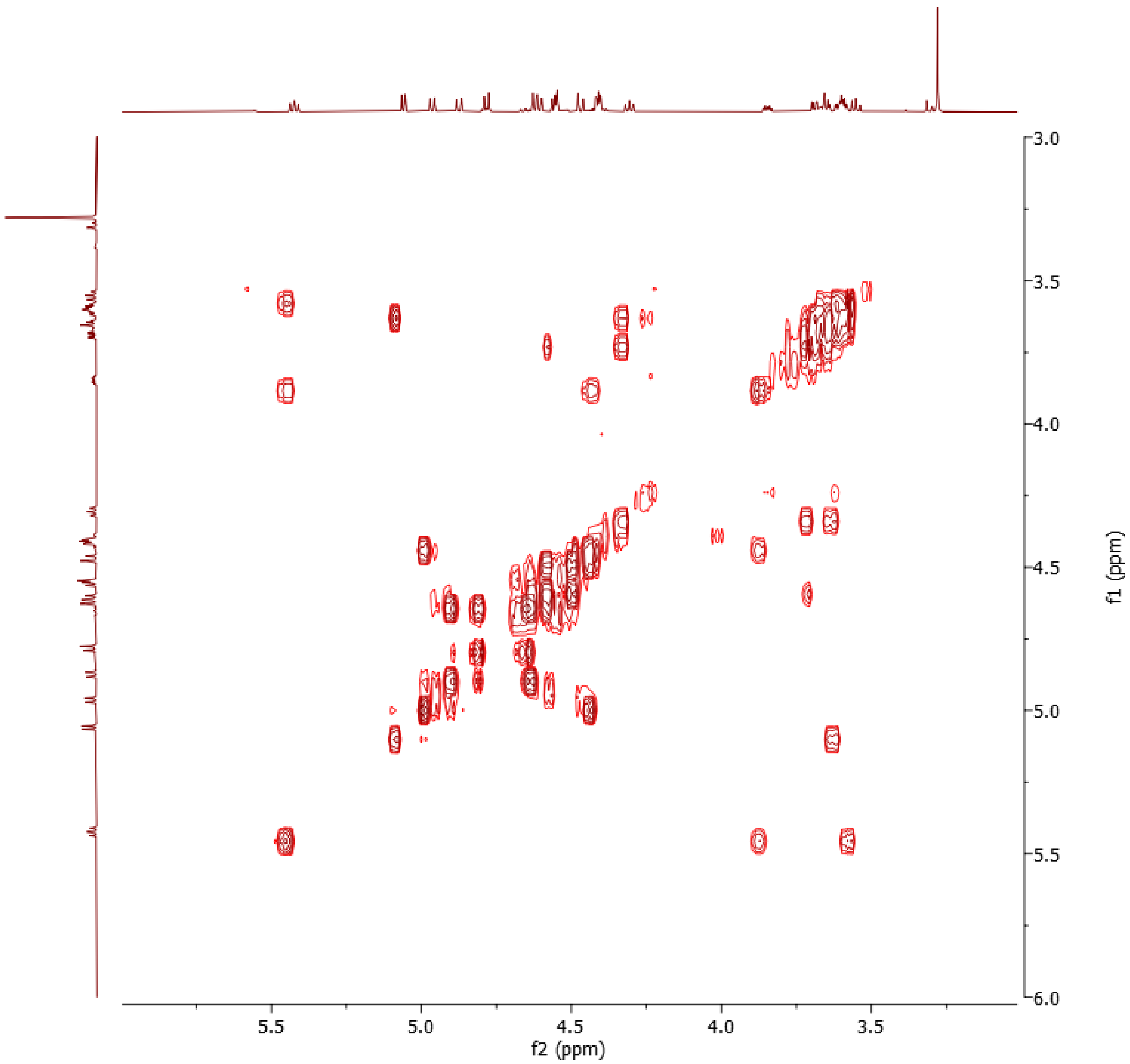

Figure S71: COSY NMR (700 MHz, $CDCl_3$)

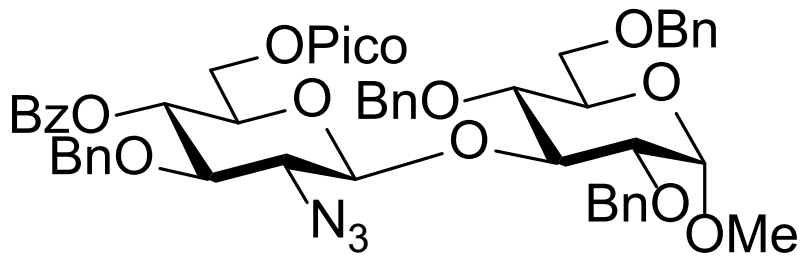

28

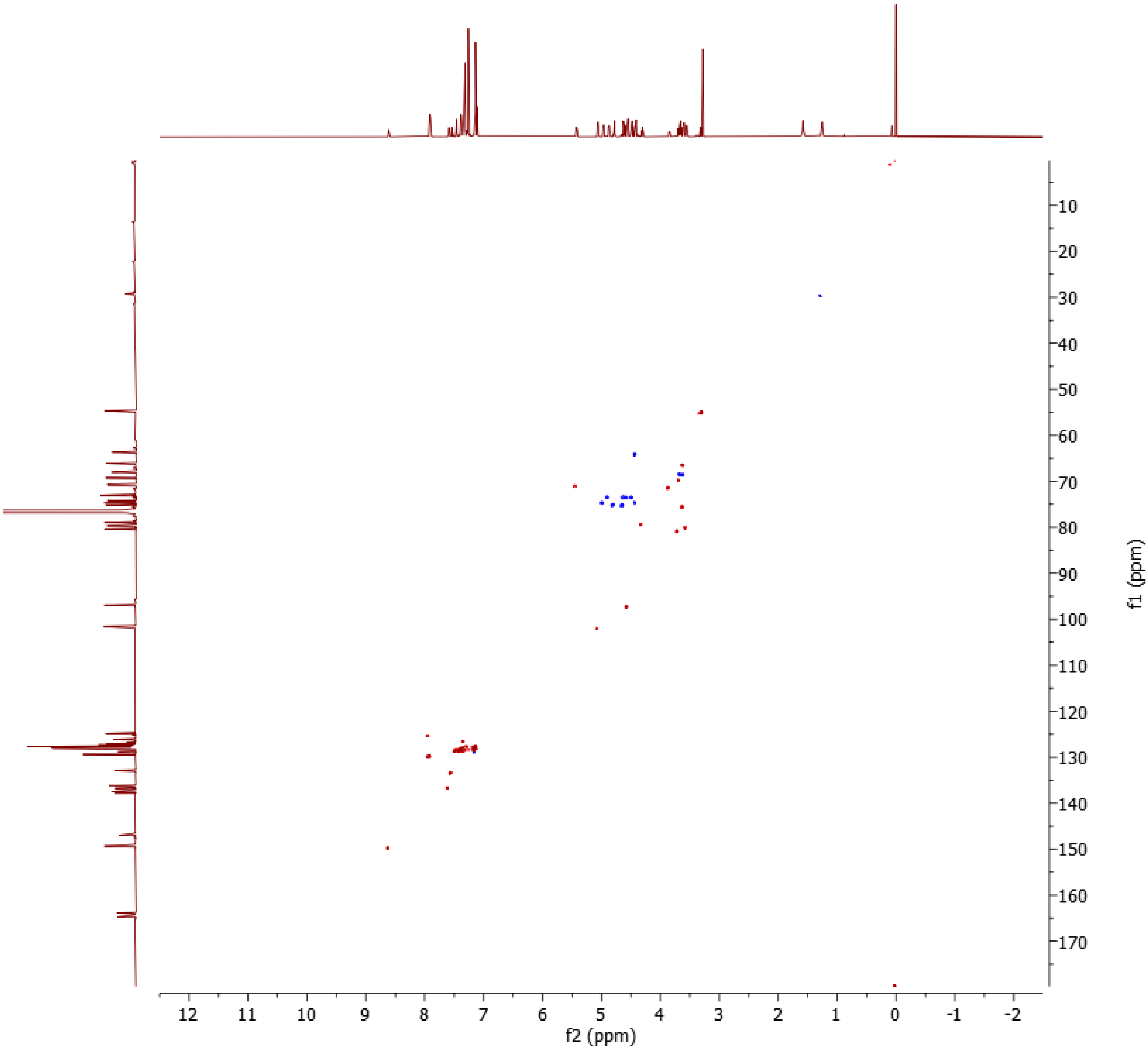

Figure S72: ^{1}H NMR and ^{13}C(^{1}H) NMR HSQC Spectrum (700 MHz, 176 MHz, $CDCl_3$)

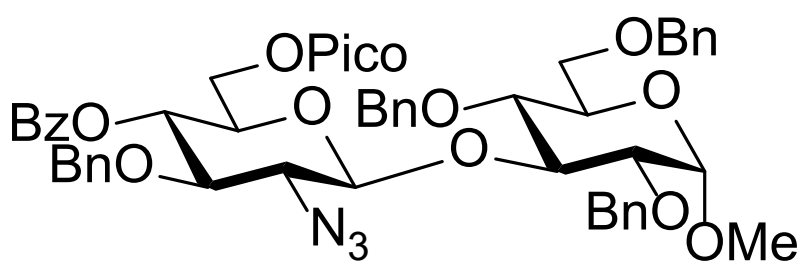

28

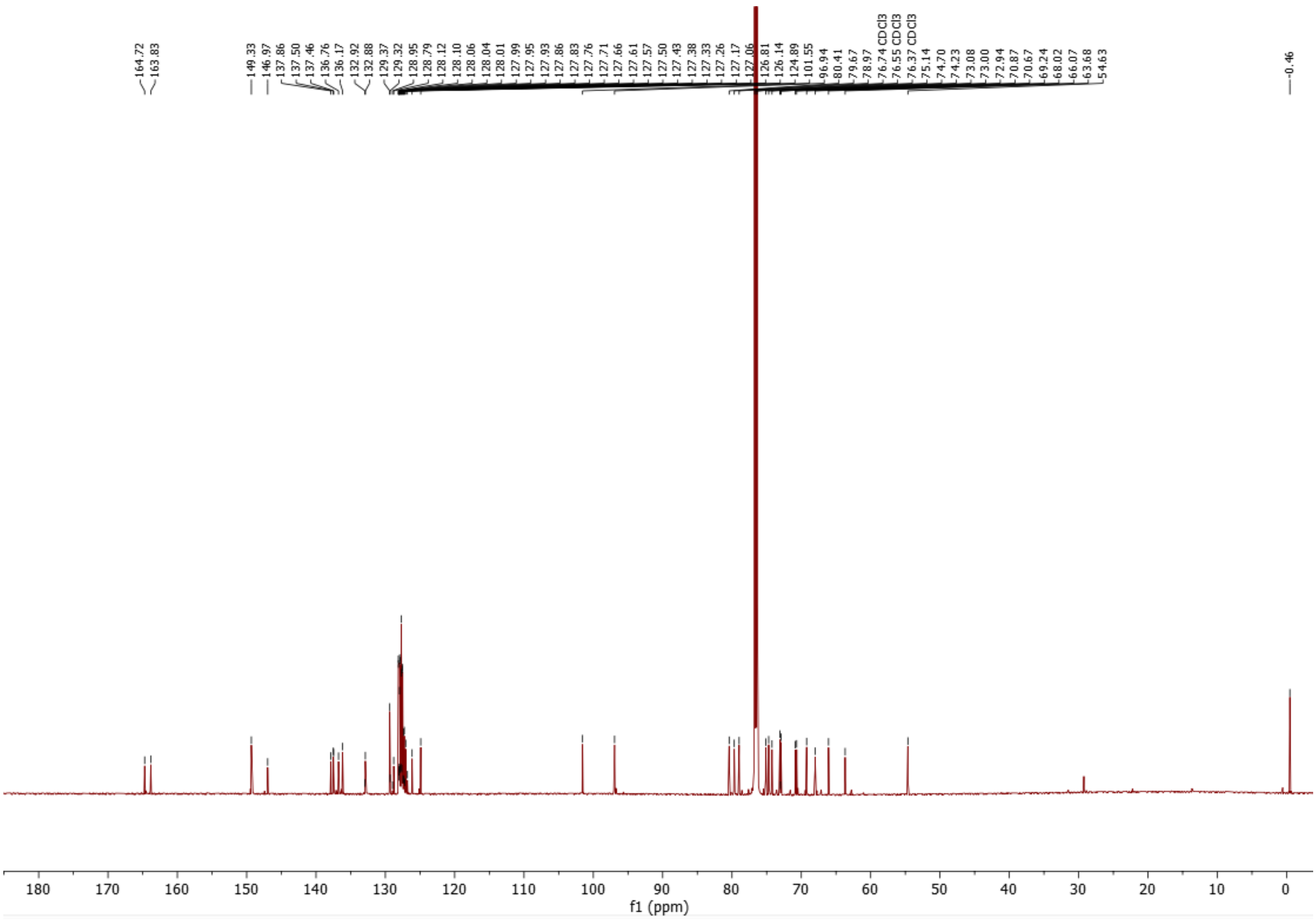

Figure S73: ^{13}C NMR (176 MHz, $CDCl_3$)

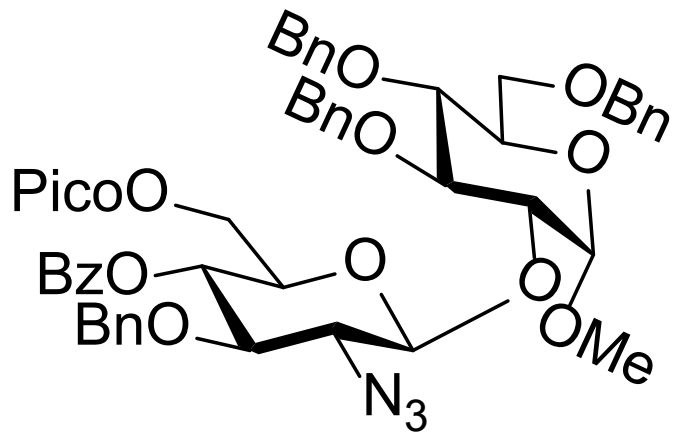

29

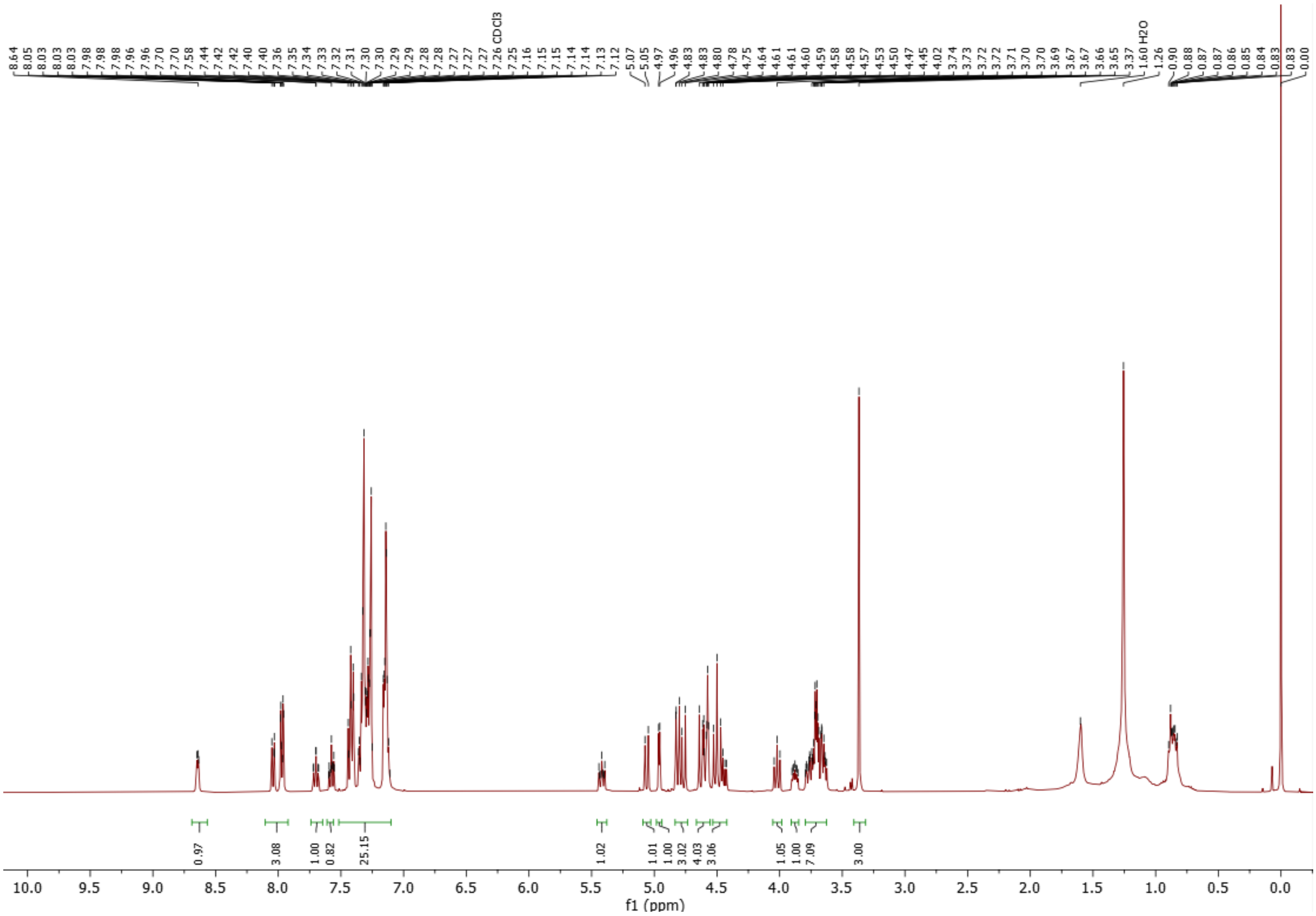

Figure S74: ^{1}H NMR (400 MHz, $CDCl_3$)

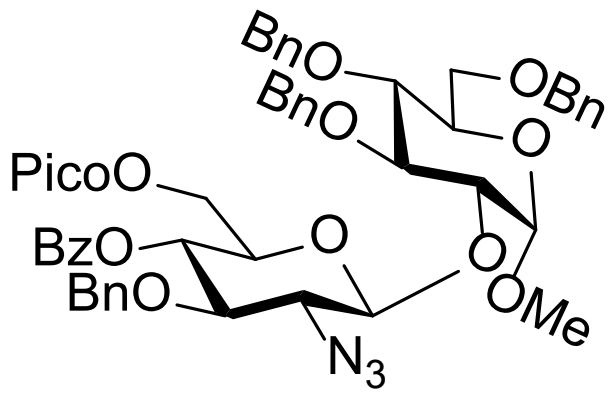

29

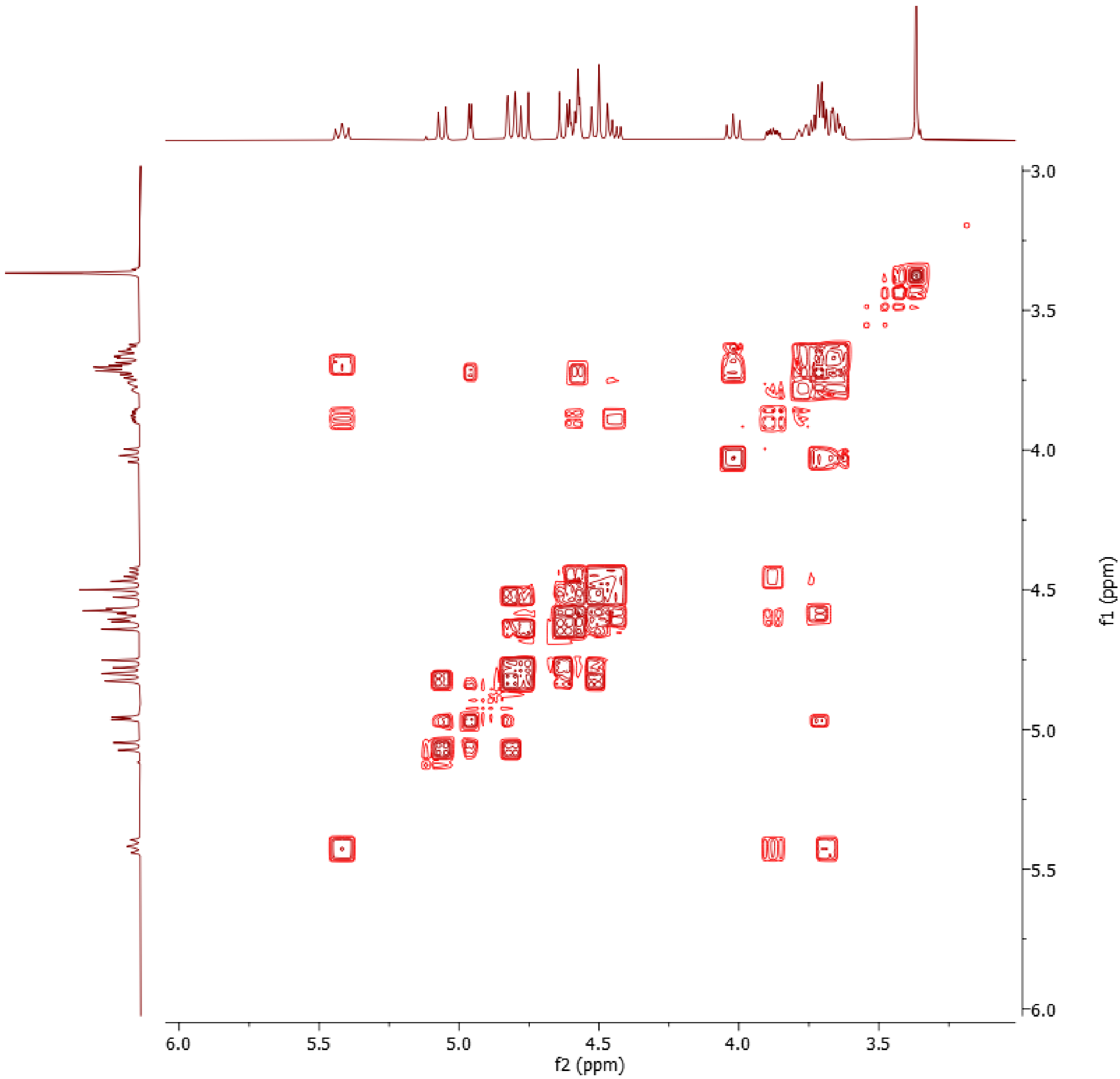

Figure S75: COSY NMR (400 MHz, $CDCl_3$)

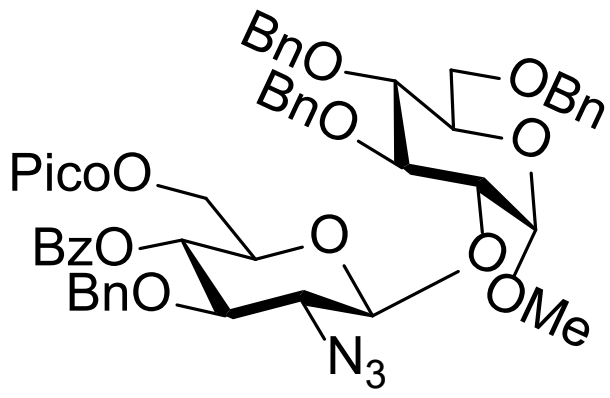

29

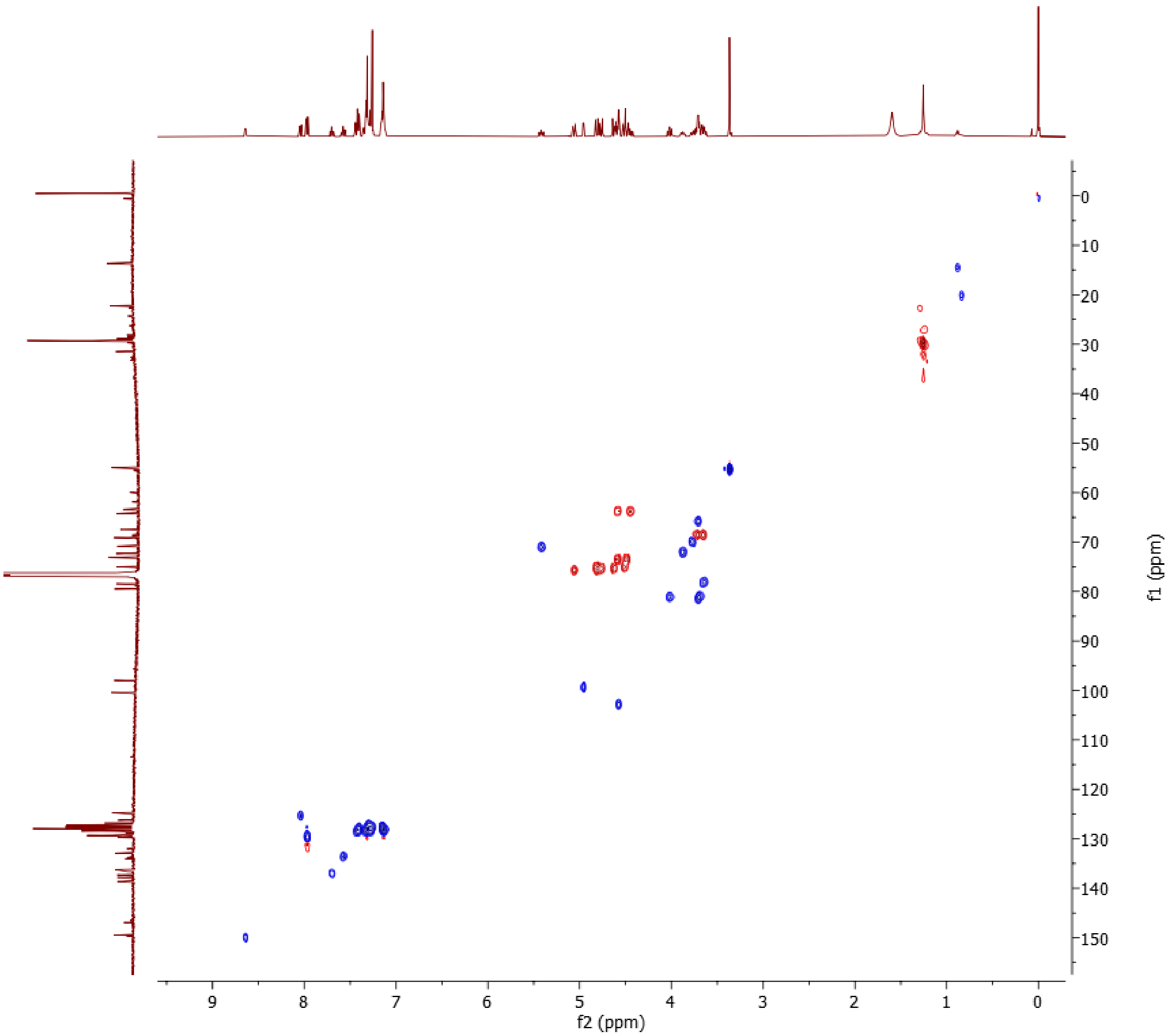

Figure S76: ^{1}H NMR and $^{13}C(^{1}H)$ NMR HSQC Spectrum (400 MHz, 101 MHz, $CDCl_3$)

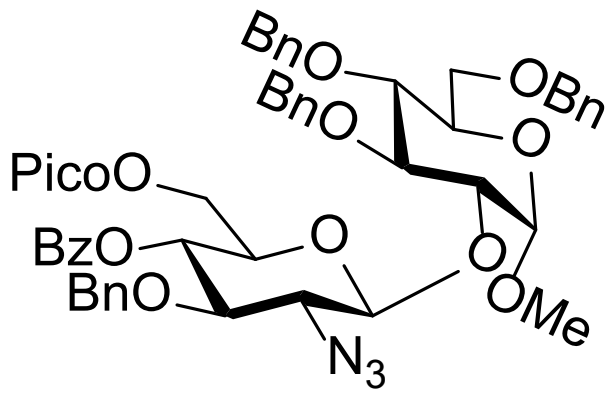

29

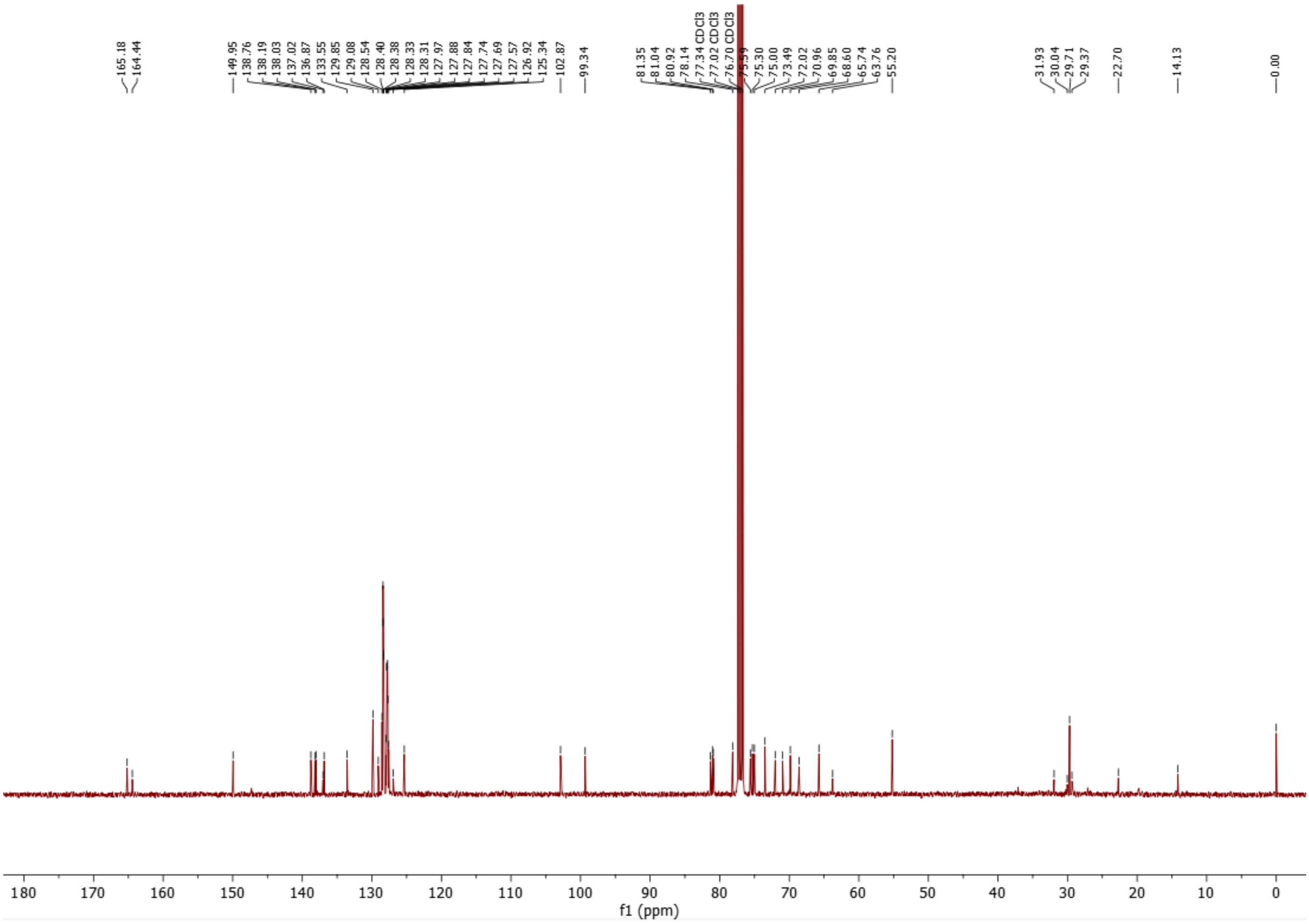

Figure S77: ^{13}C NMR (101 MHz, $CDCl_3$)

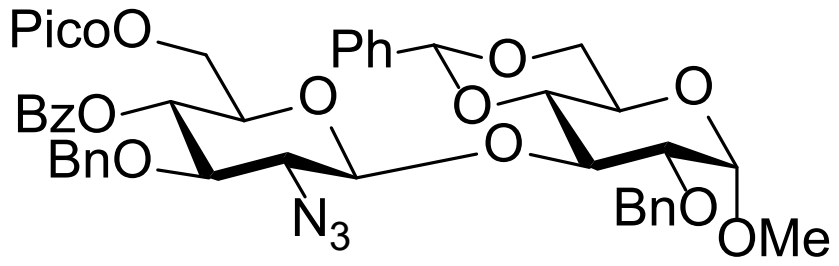

30

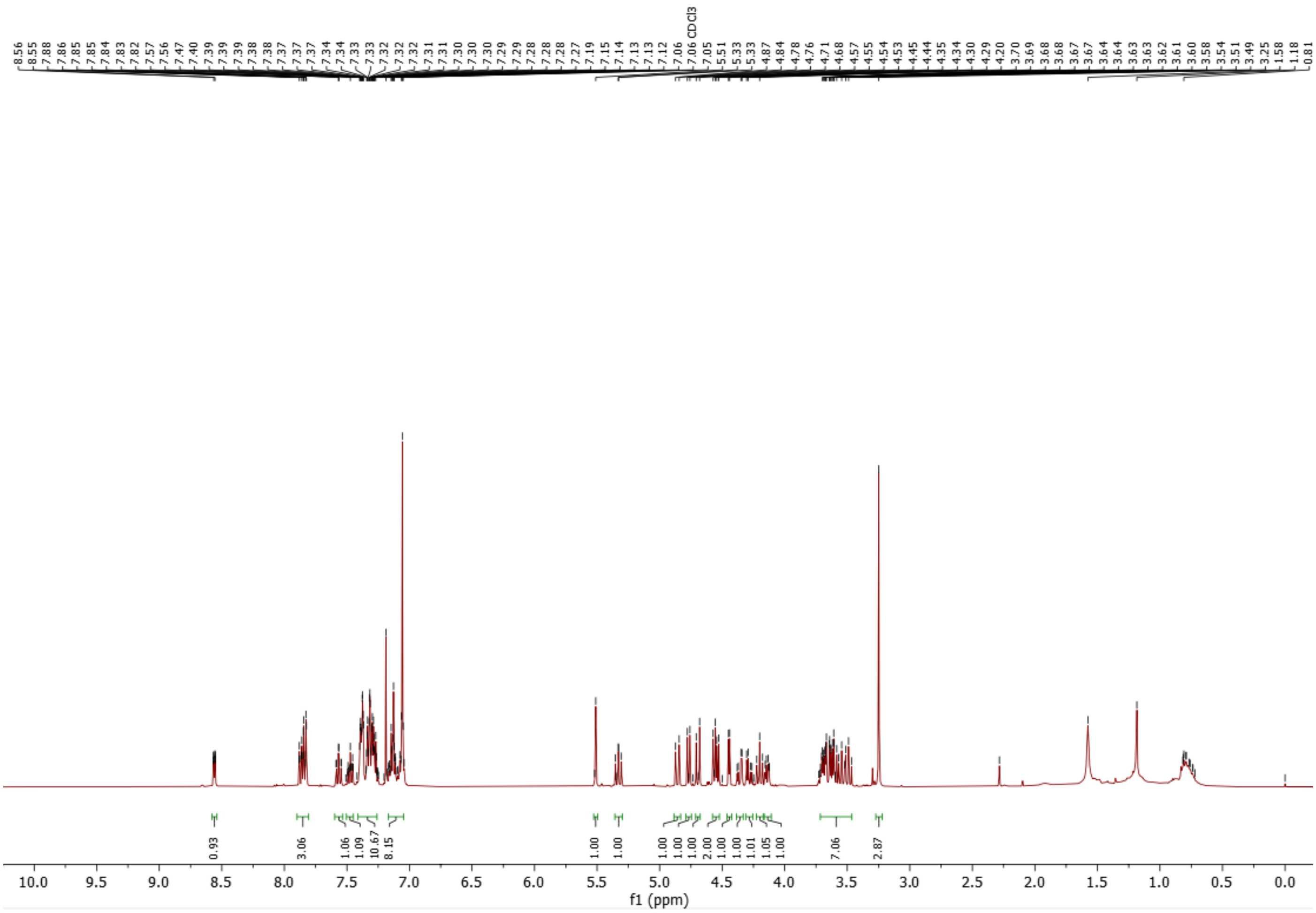

Figure S78: ^{1}H NMR (400 MHz, $CDCl_3$)

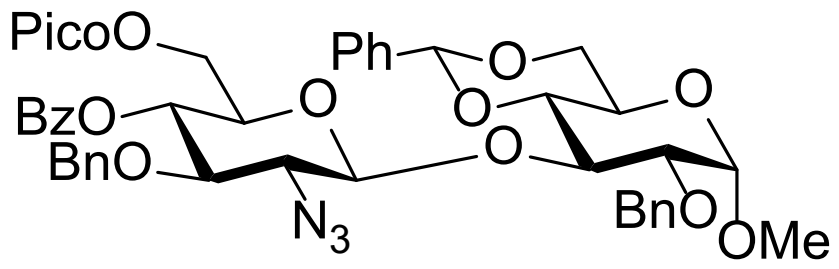

30

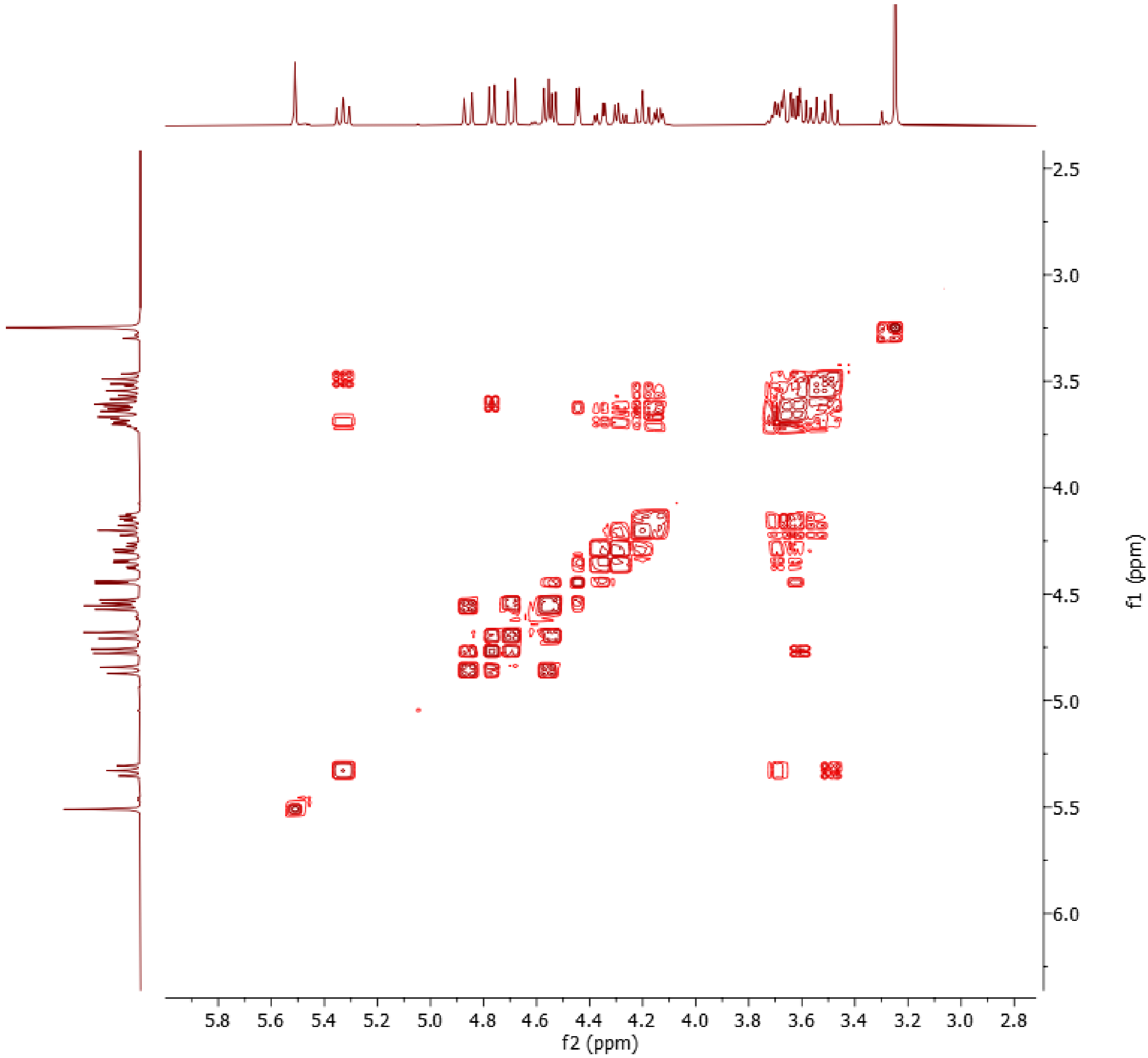

Figure S79: COSY NMR (400 MHz, $CDCl_3$)

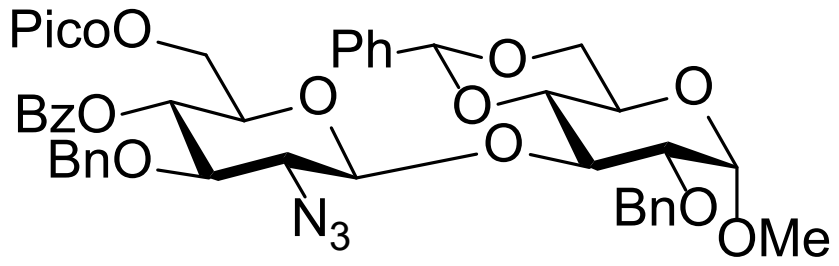

30

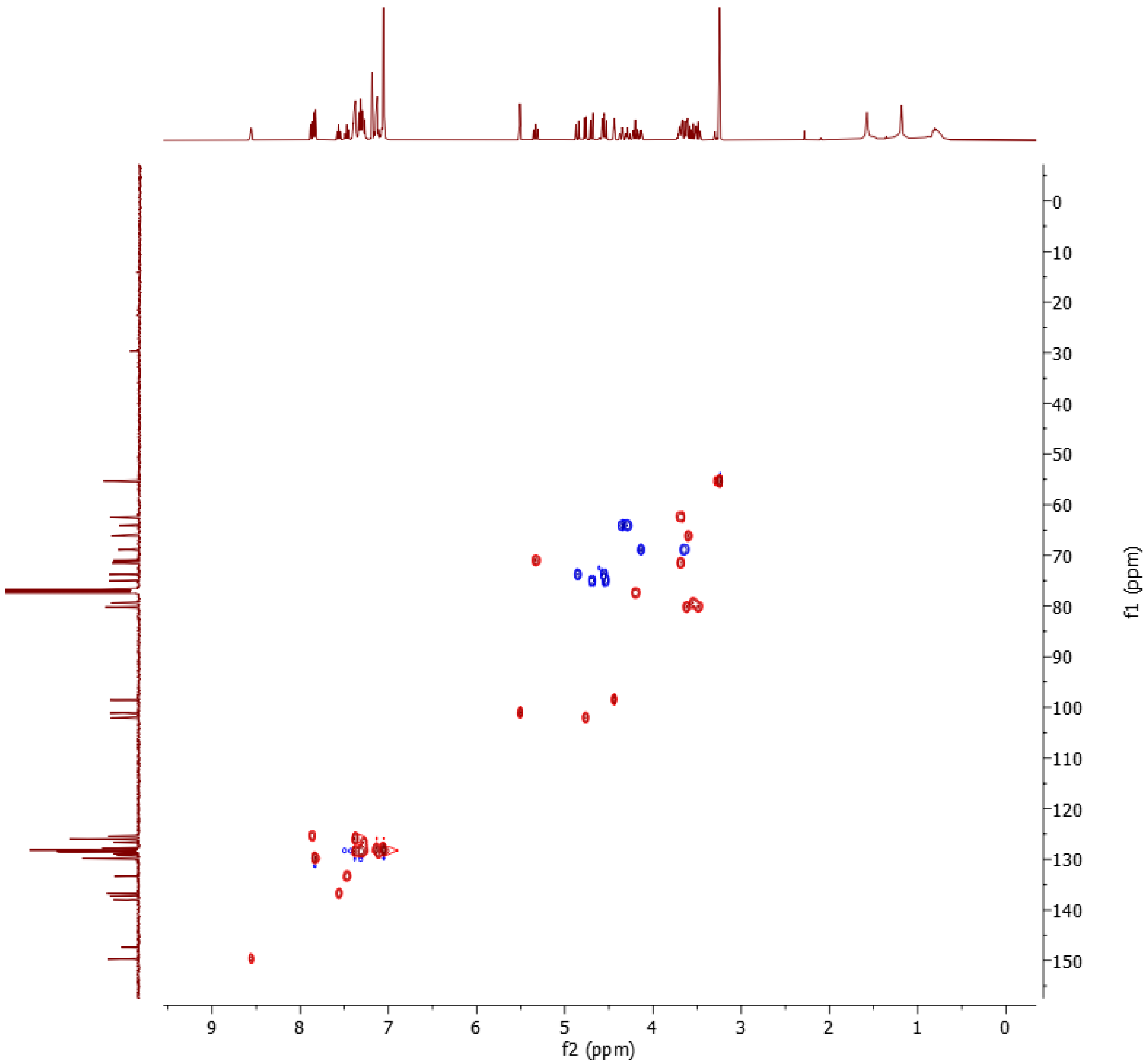

Figure S80: ^{1}H NMR and ^{13}C(^{1}H) NMR HSQC Spectrum (400 MHz, 101 MHz, $CDCl_3$)

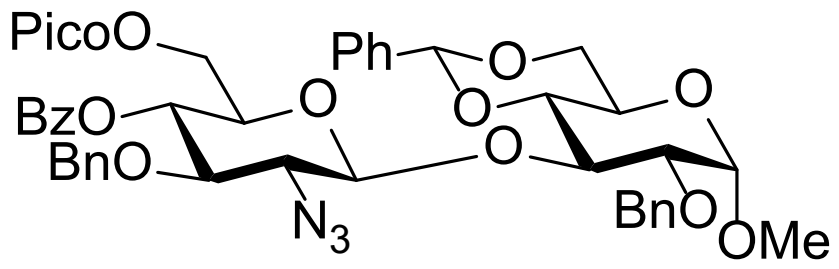

30

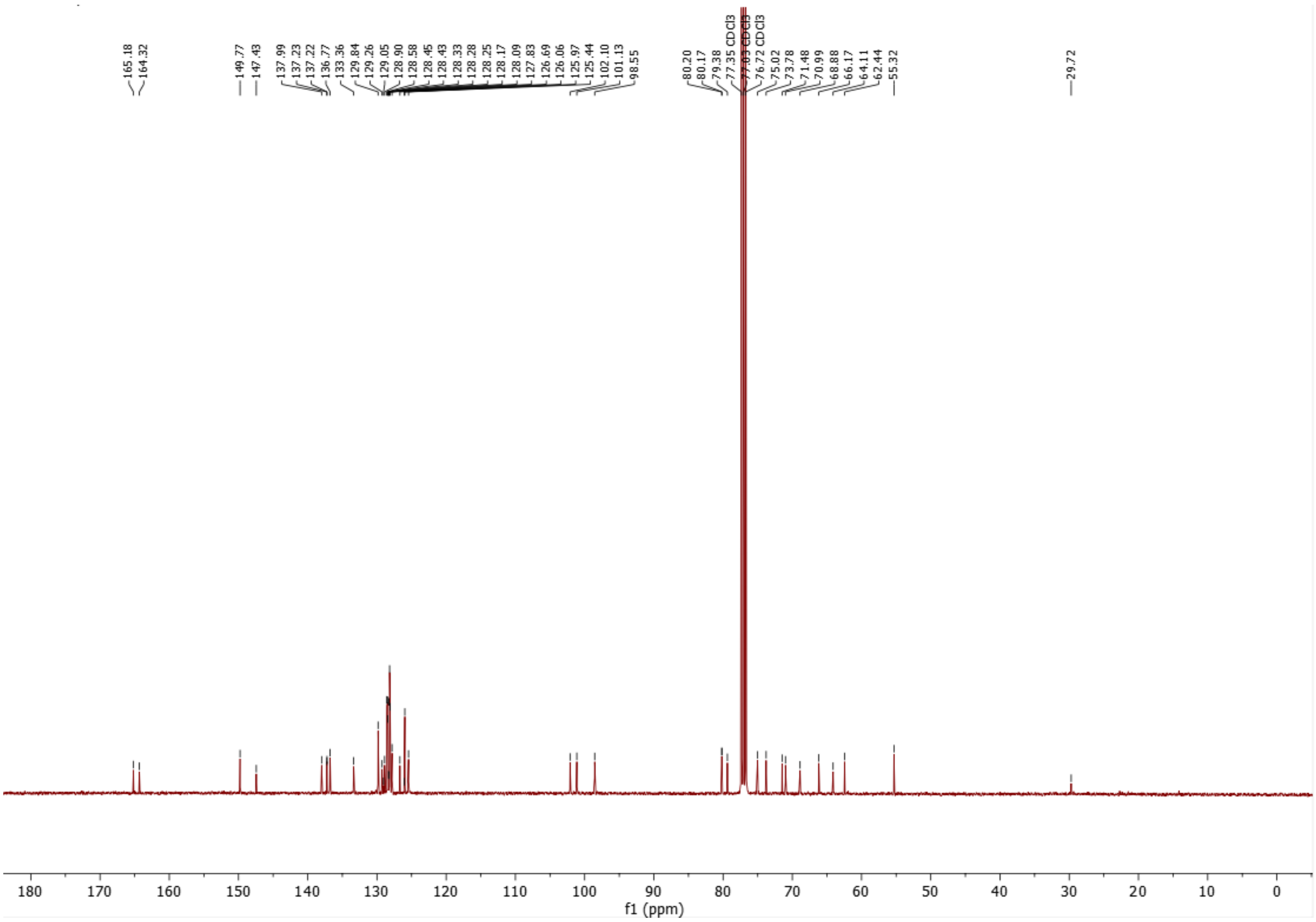

Figure S81: ^{13}C NMR (101 MHz, $CDCl_3$)

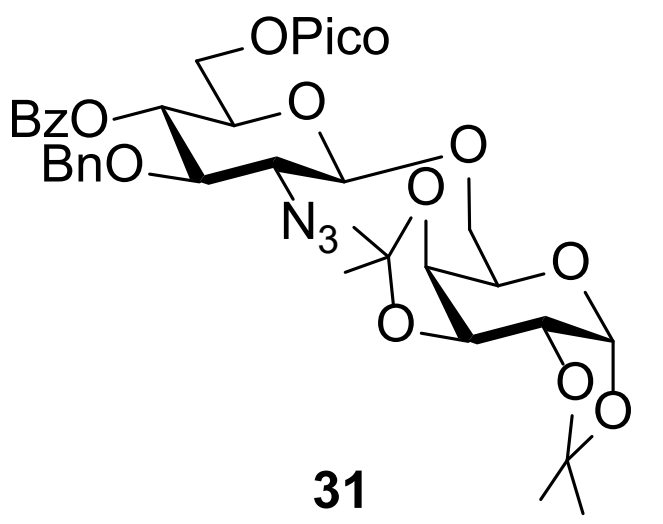

31

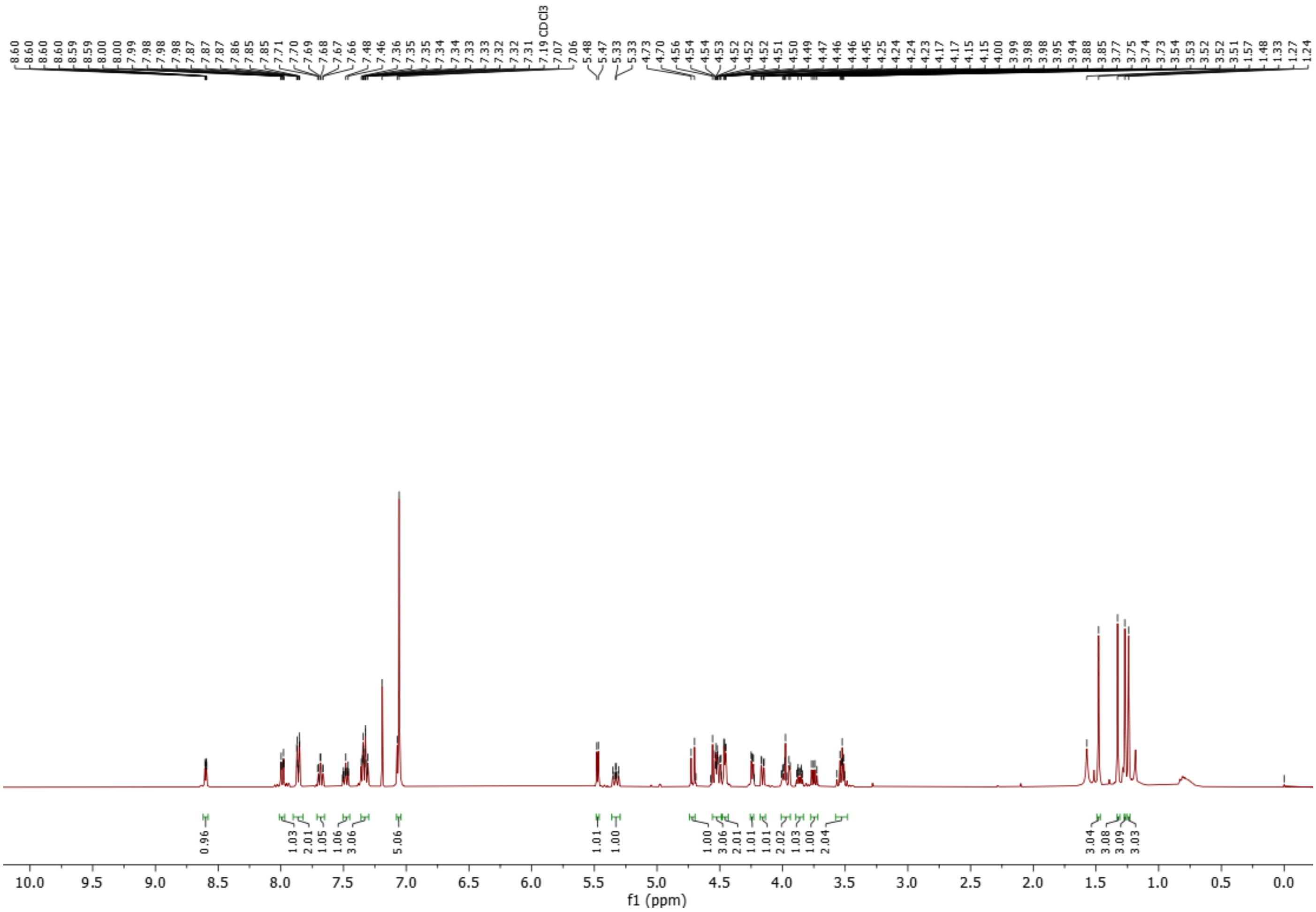

Figure S82: ^{1}H NMR (400 MHz, $CDCl_3$)

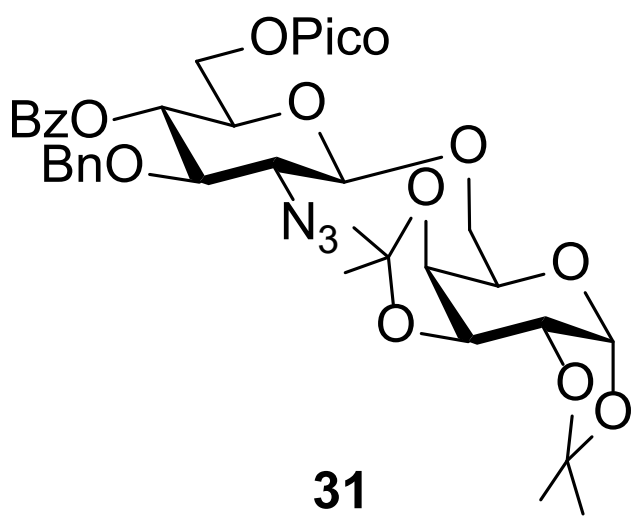

31

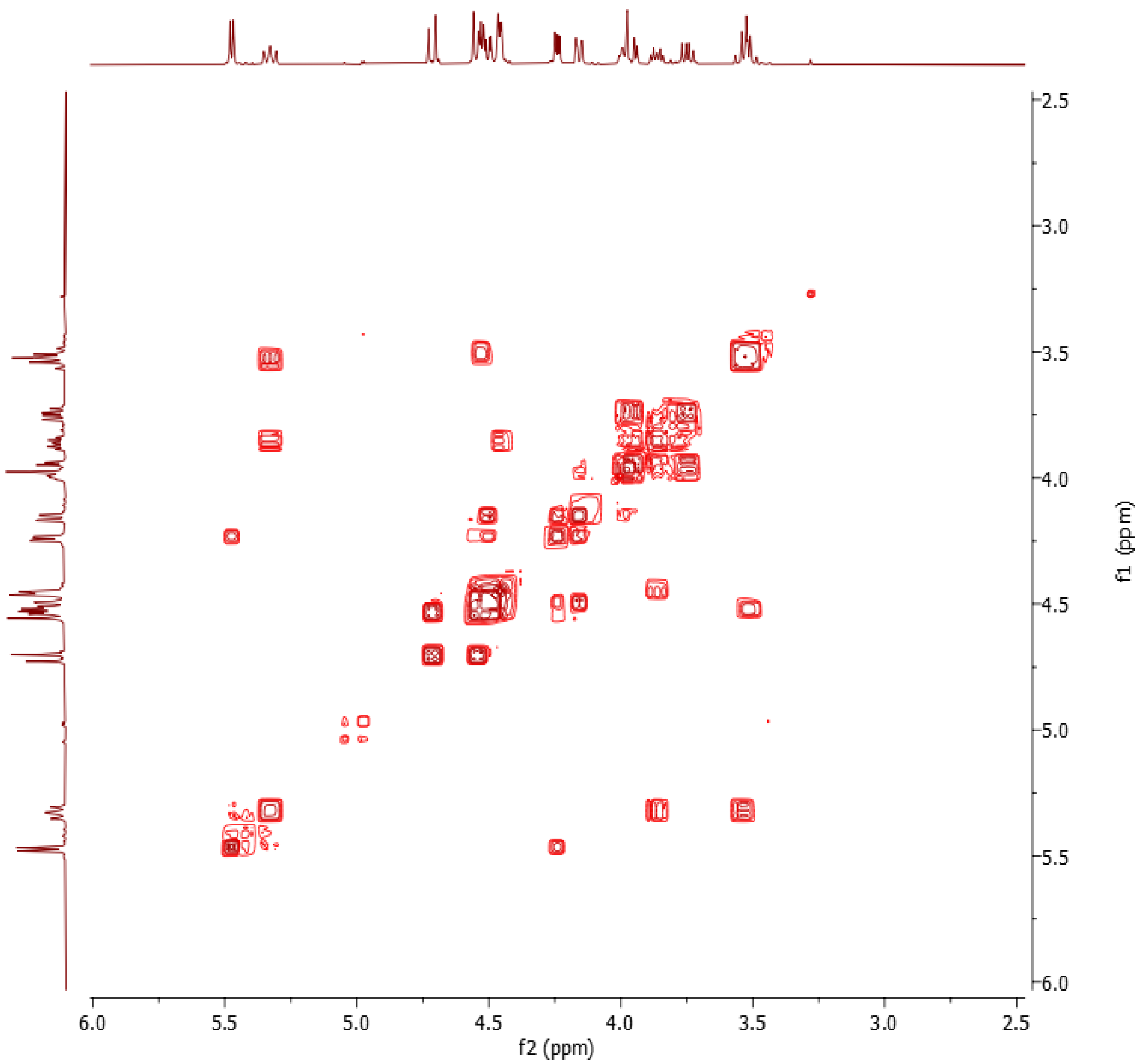

Figure S83: COSY NMR (400 MHz, $CDCl_3$)

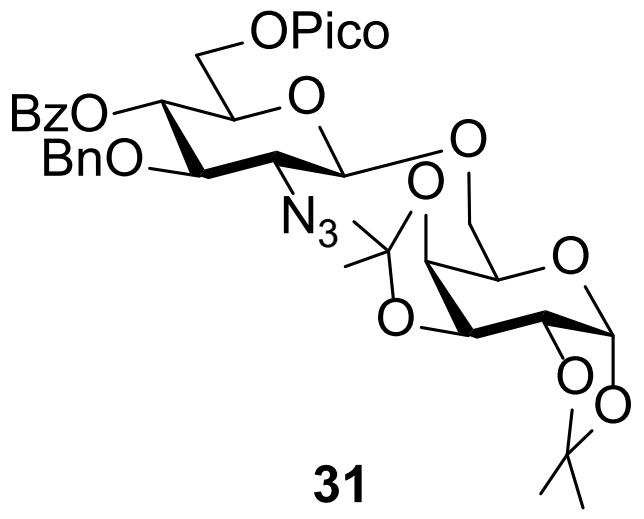

31

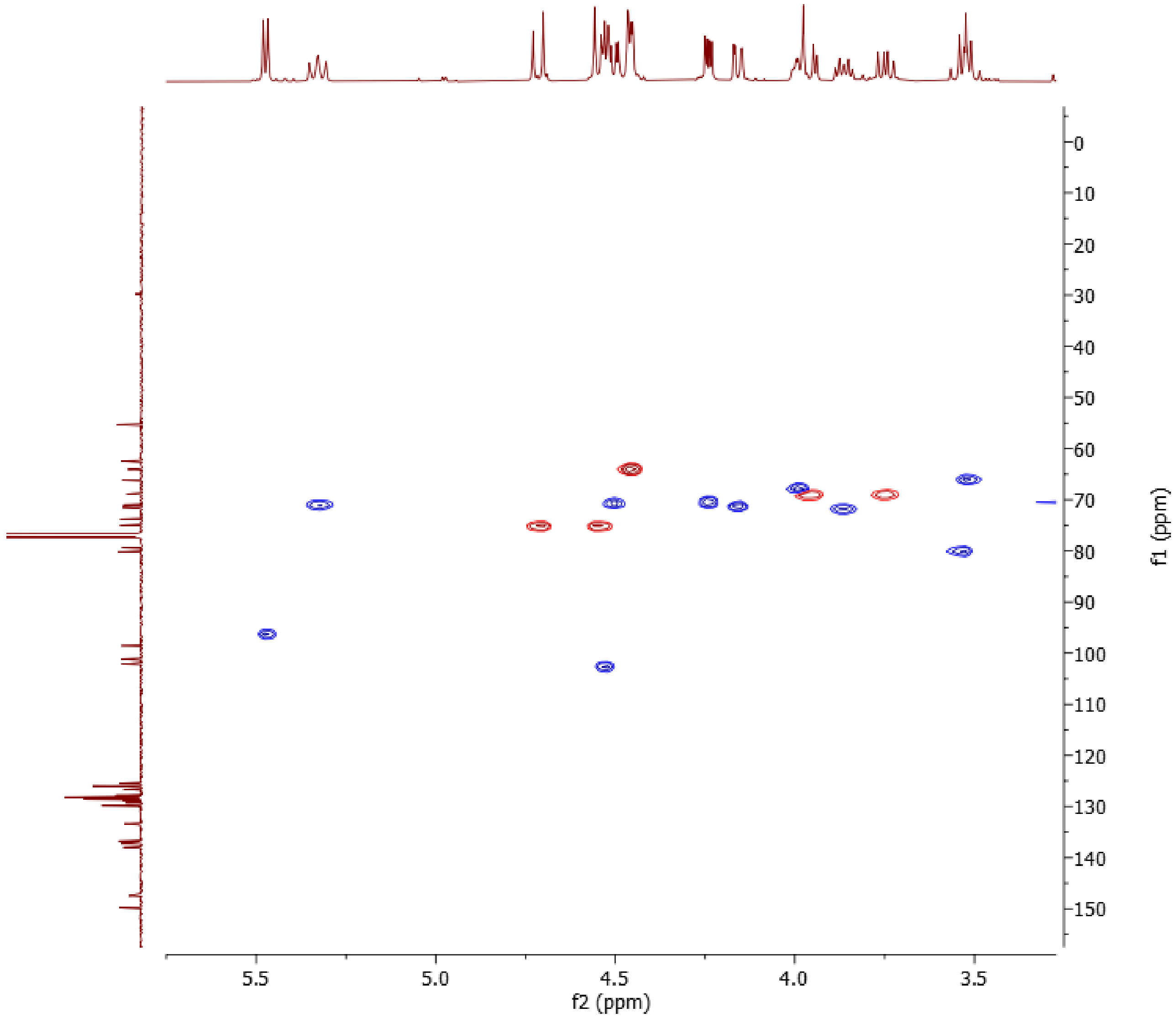

Figure S84: ^{1}H NMR and $^{13}C(^{1}H)$ NMR HSQC Spectrum (400 MHz, 101 MHz, $CDCl_3$)

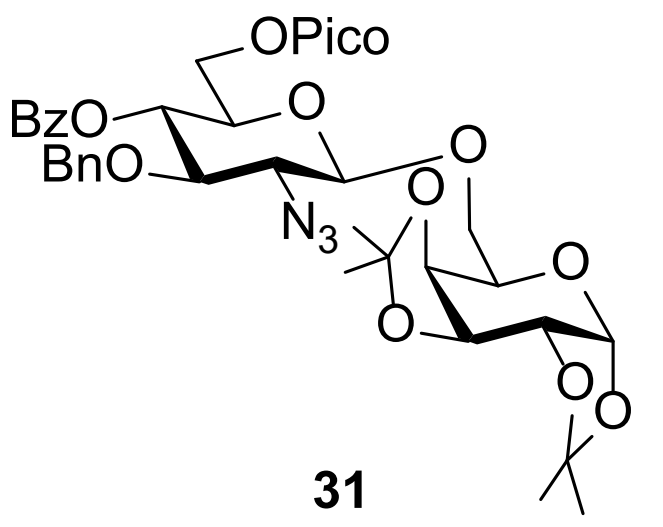

31

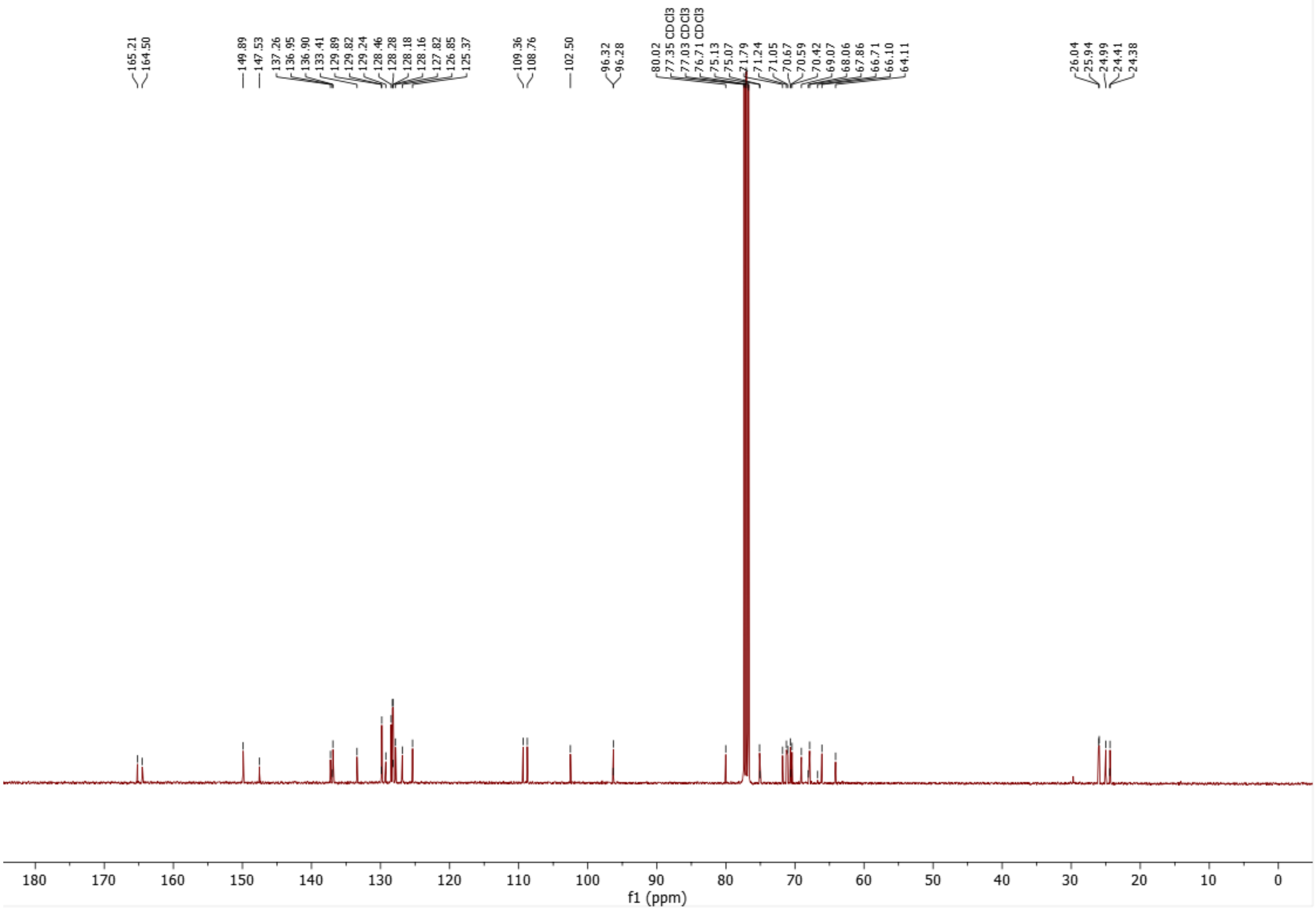

Figure S85: ^{13}C NMR (101 MHz, CDCl$_3$)

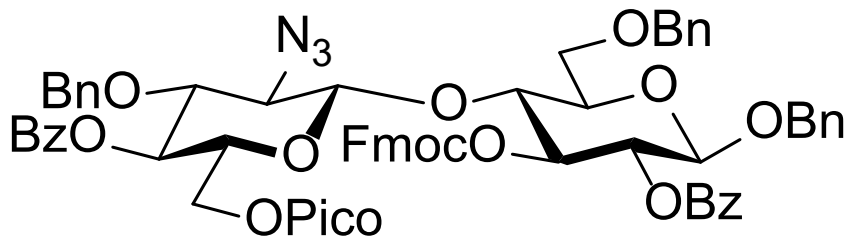

32

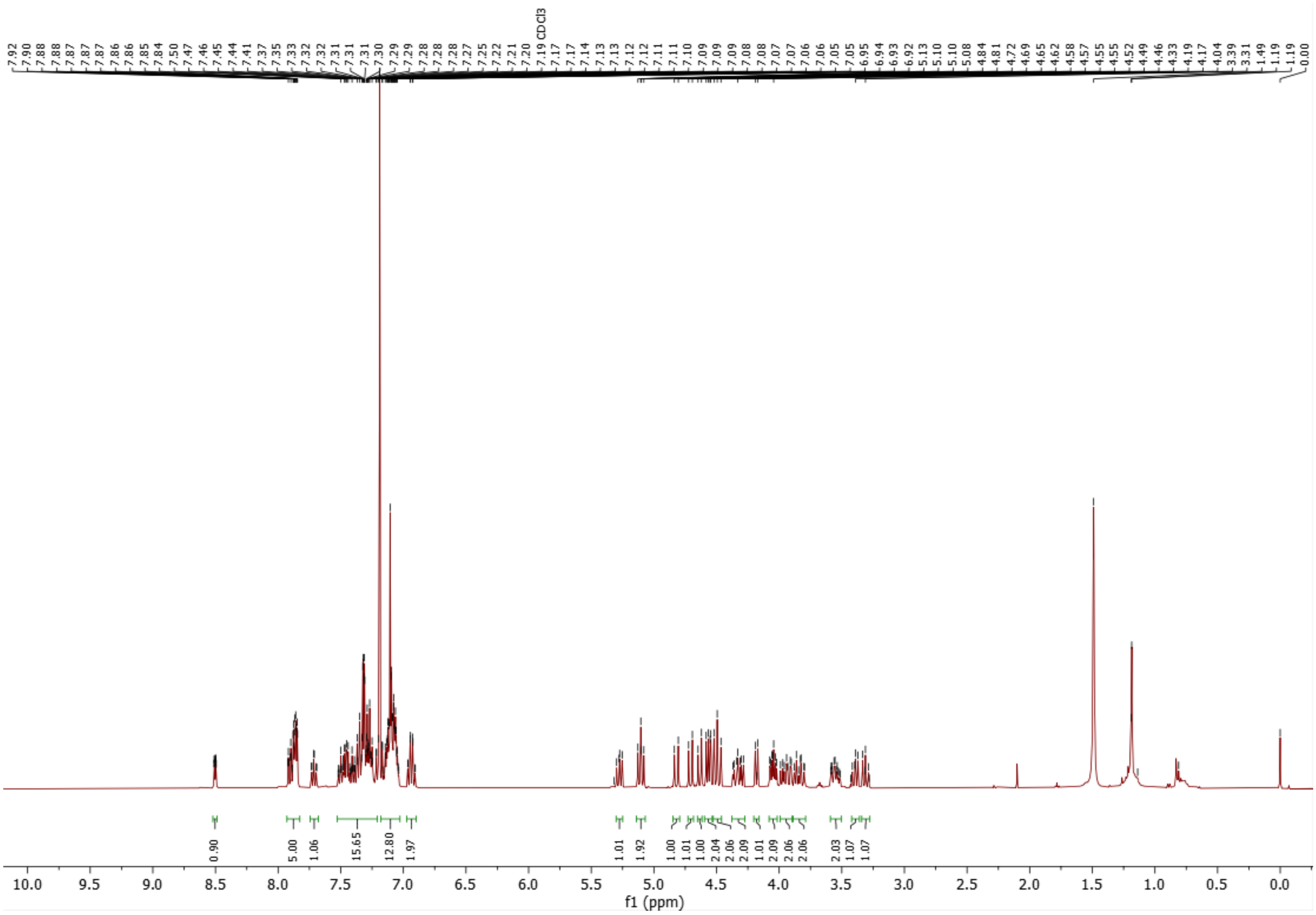

Figure S86: ^{1}H NMR (400 MHz, $CDCl_3$)

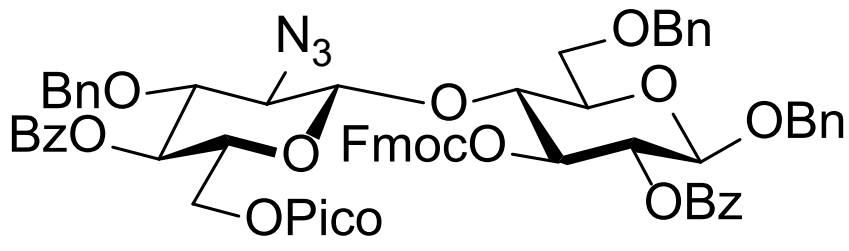

32

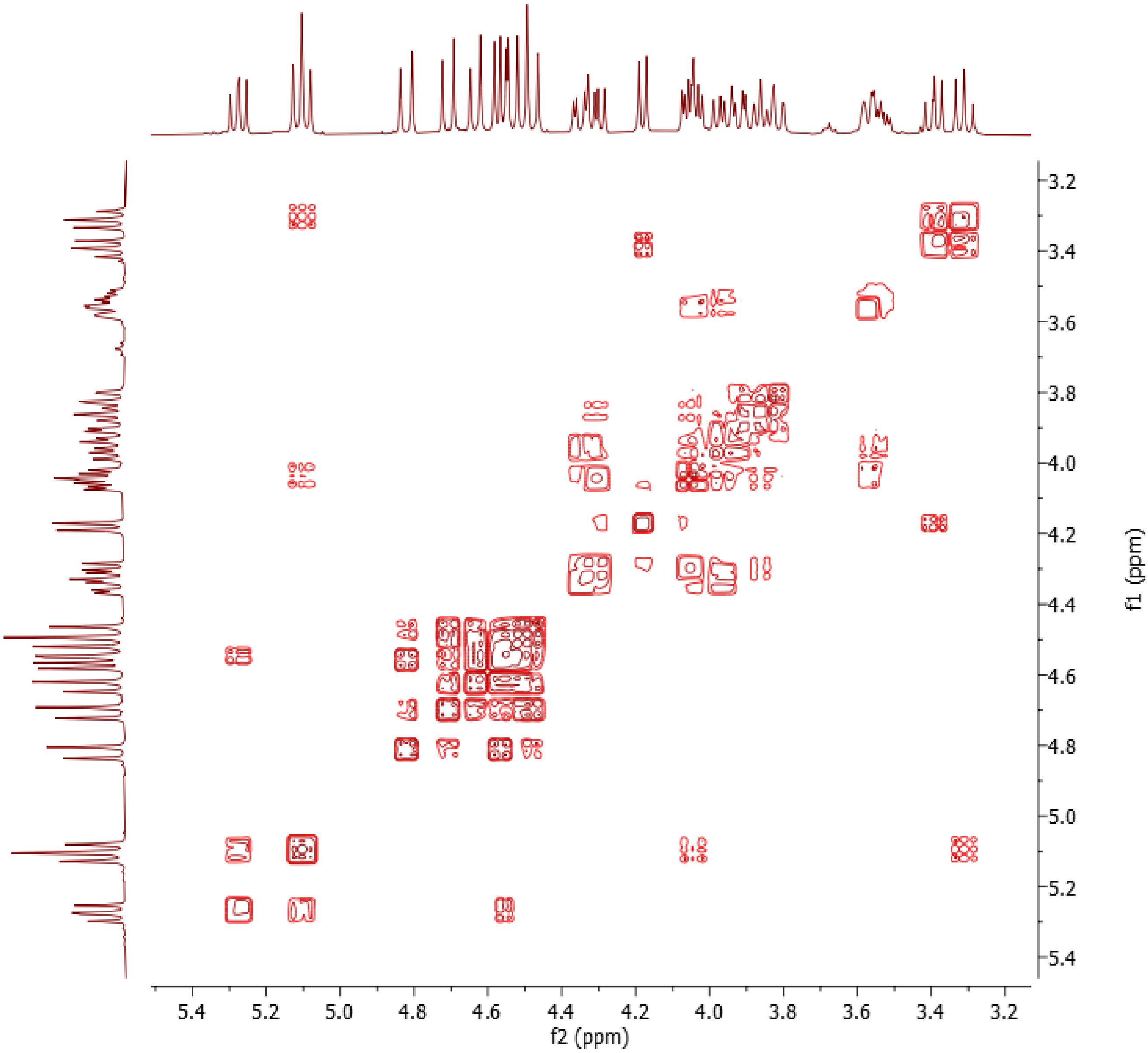

Figure S87: COSY NMR (700 MHz, $CDCl_3$)

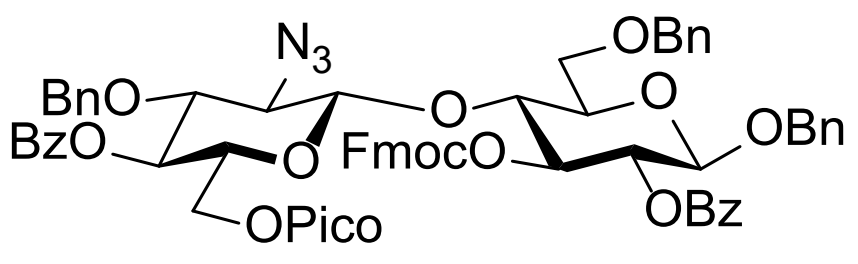

32

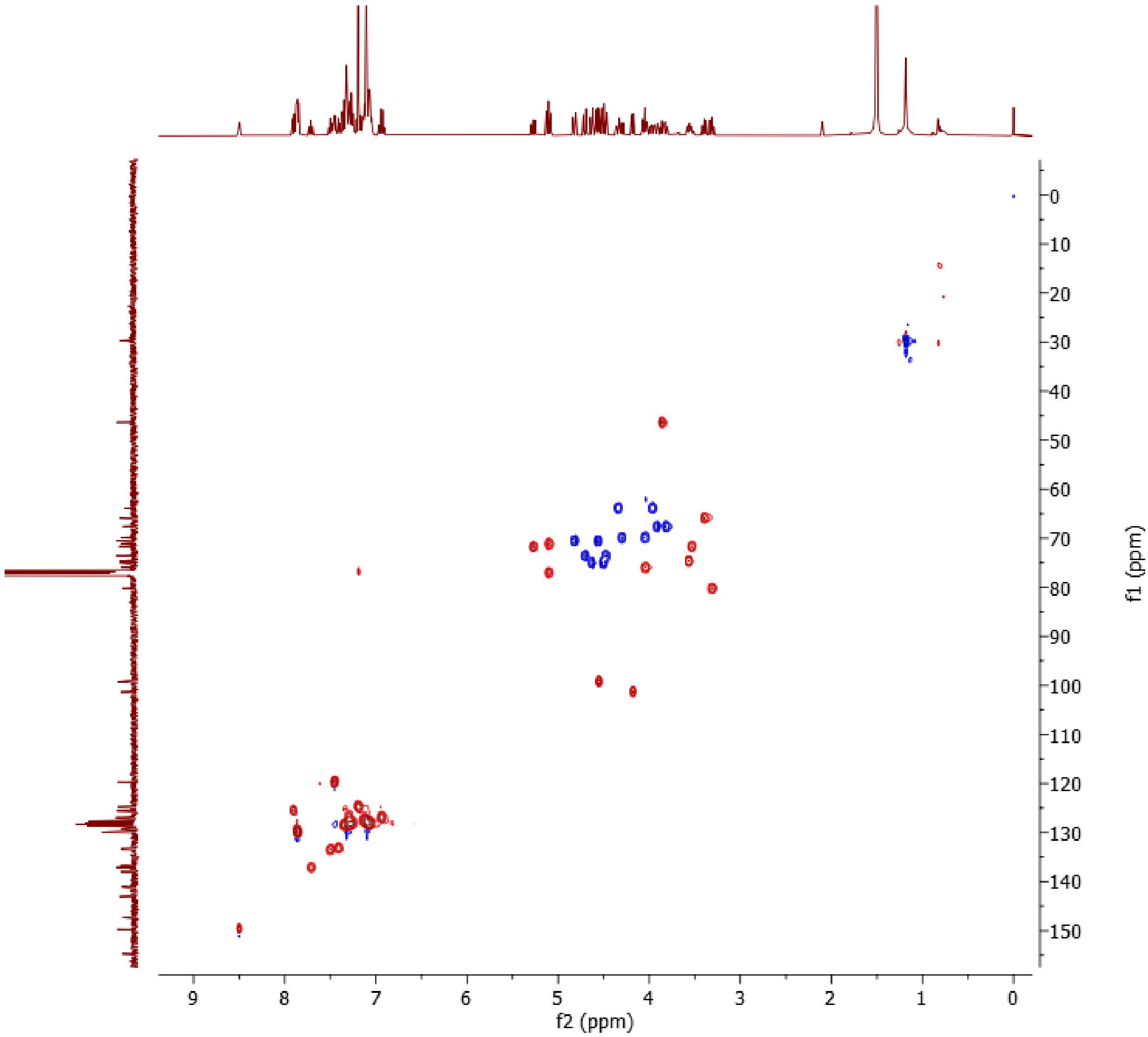

Figure S88: ^{1}H NMR and $^{13}C(^{1}H)$ NMR HSQC Spectrum (400 MHz, 101 MHz, $CDCl_3$)

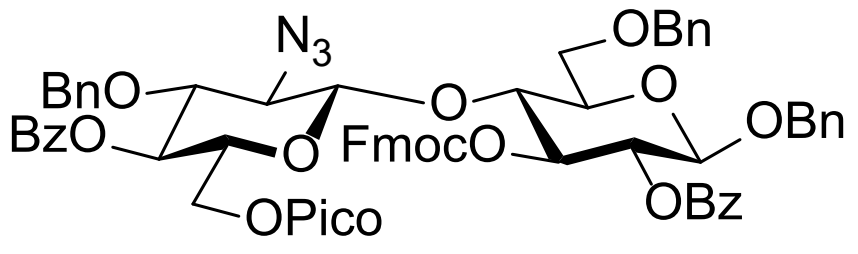

32

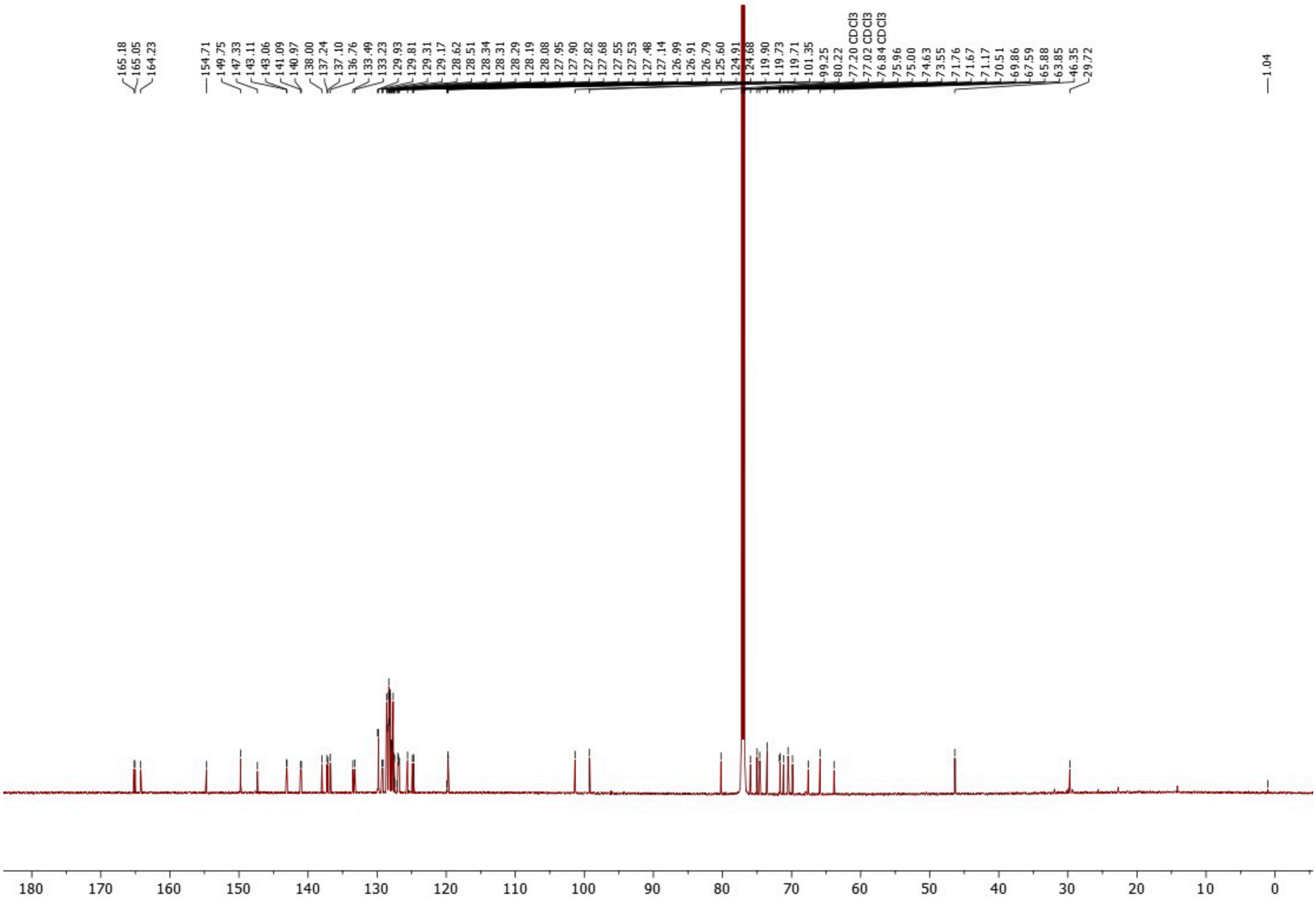

Figure S89: ^{13}C NMR (101 MHz, $CDCl_3$)

Spectra of PNAG tetrasaccharide fragment 36 and all intermediates

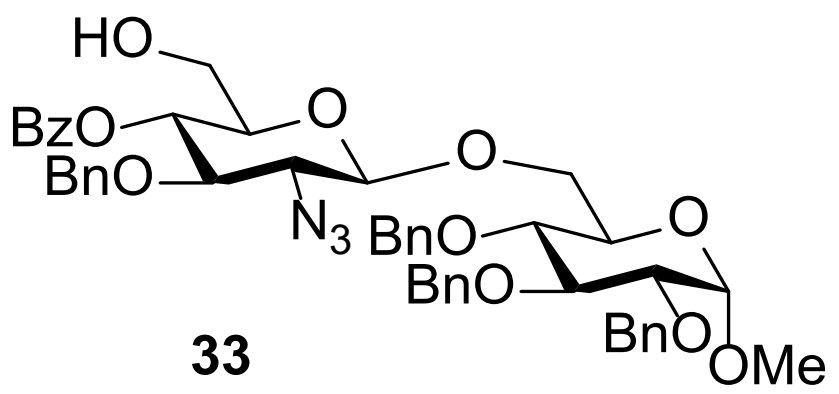

33

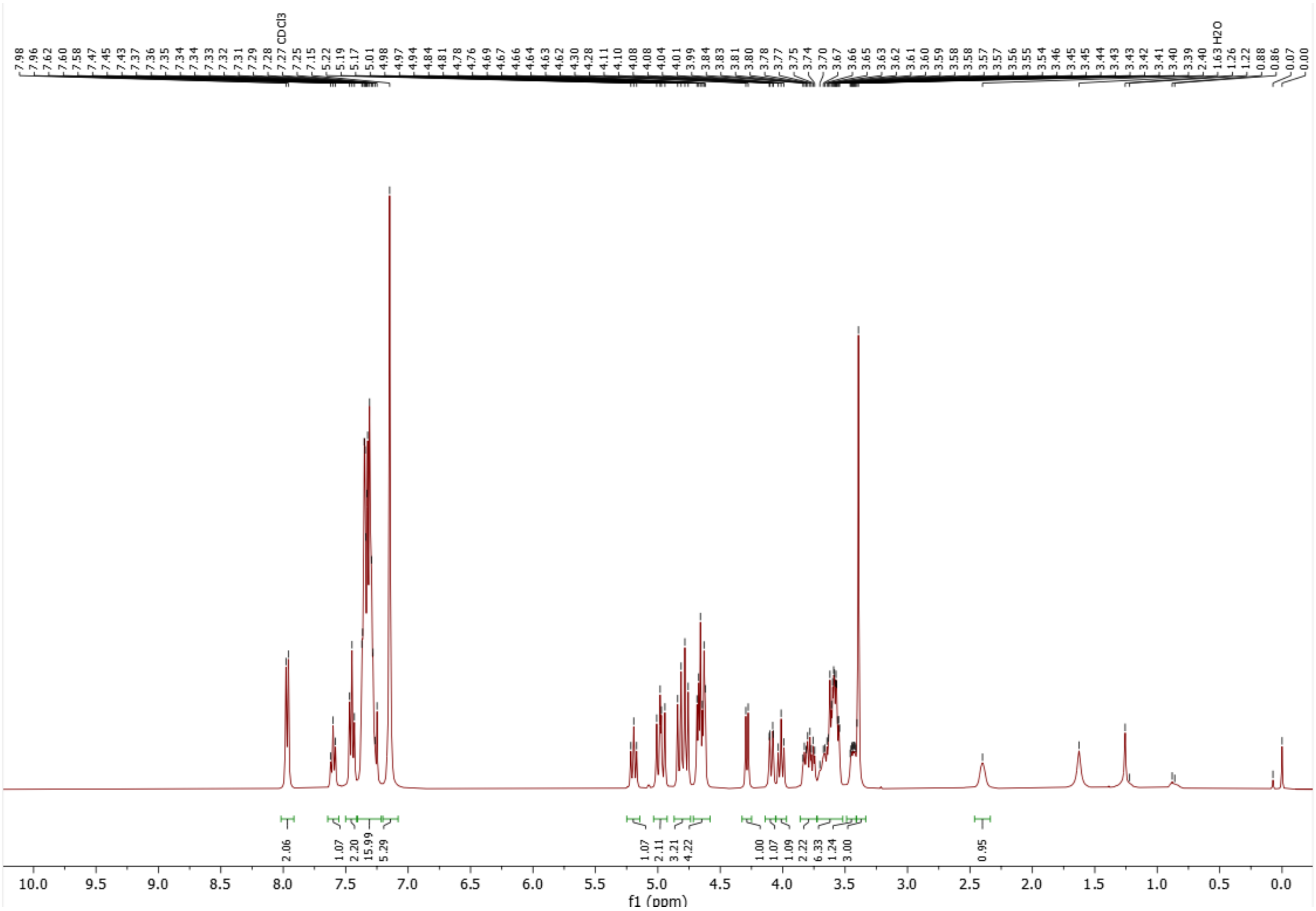

Figure S90: ^{1}H NMR (700 MHz, $CDCl_3$)

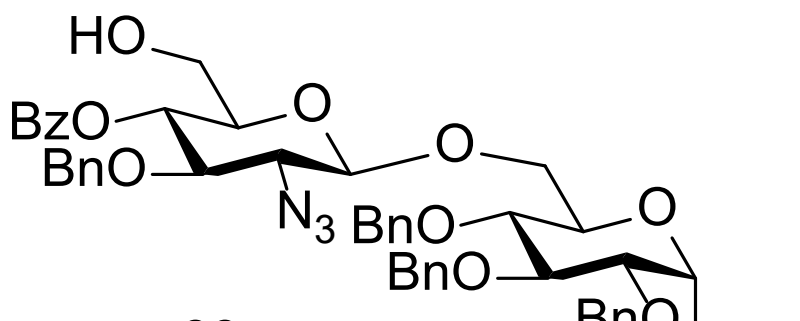

33

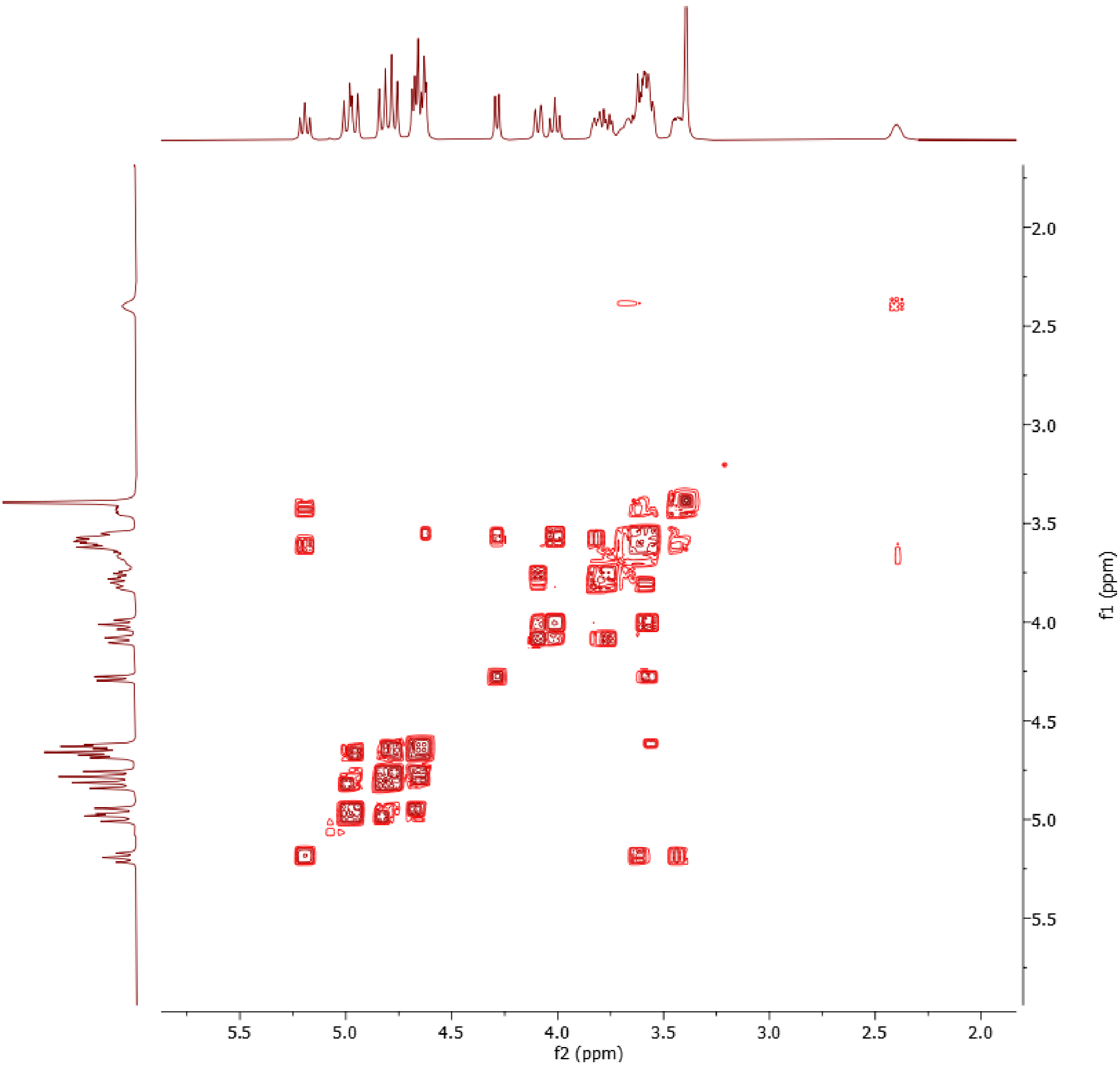

Figure S91: COSY NMR (700 MHz, $CDCl_3$)

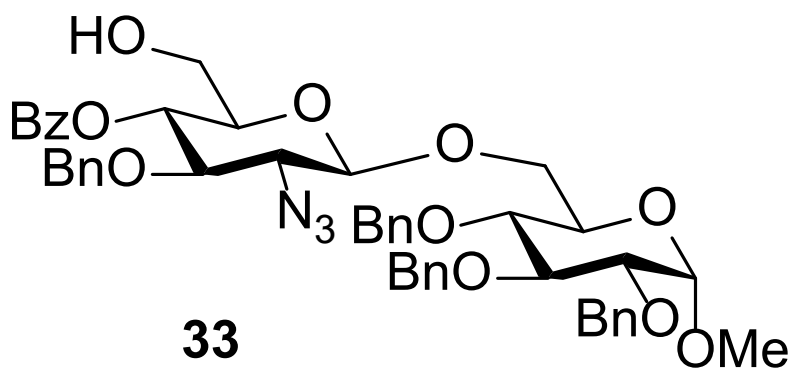

33

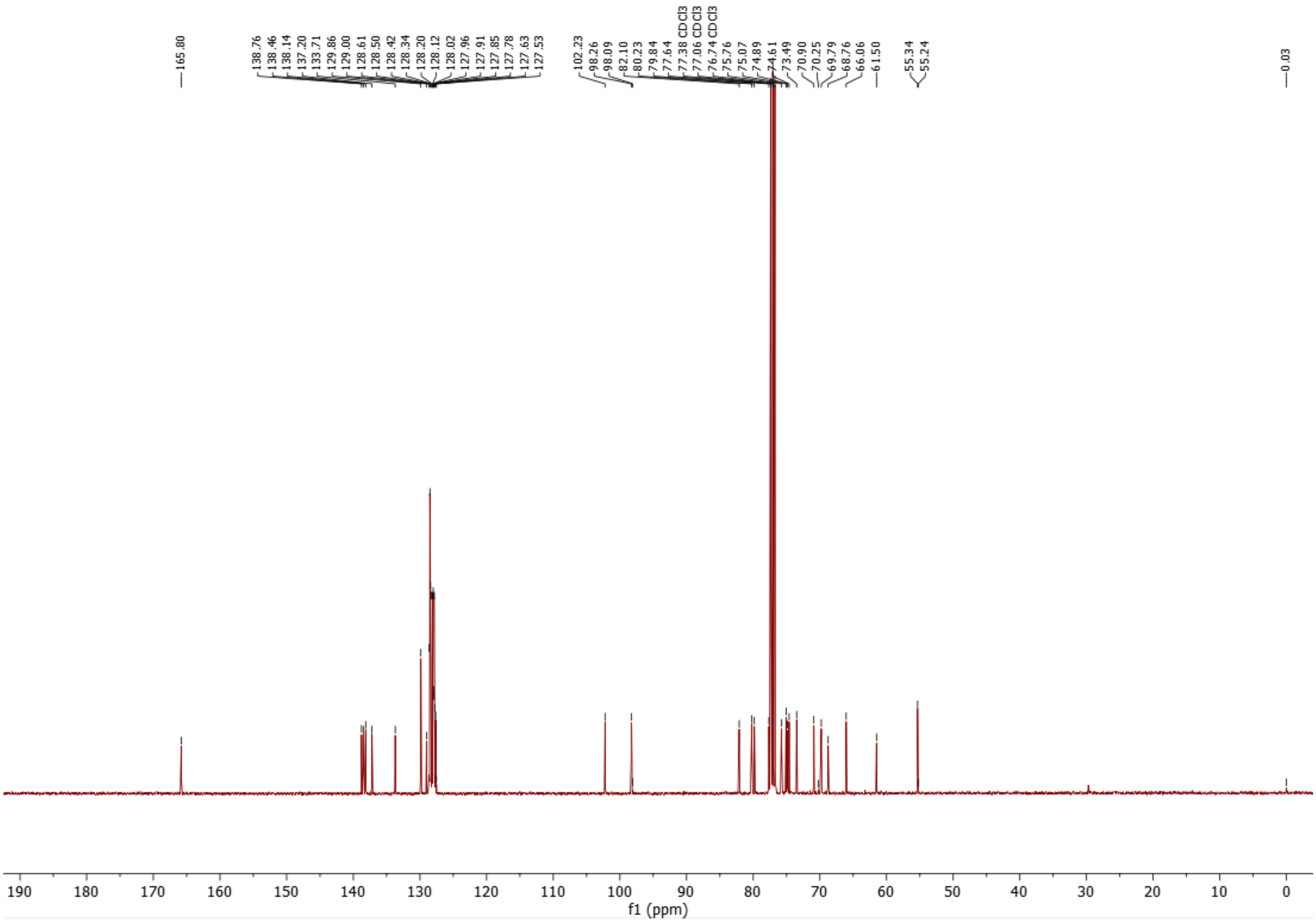

Figure S92: ^{13}C NMR (176 MHz, $CDCl_3$)

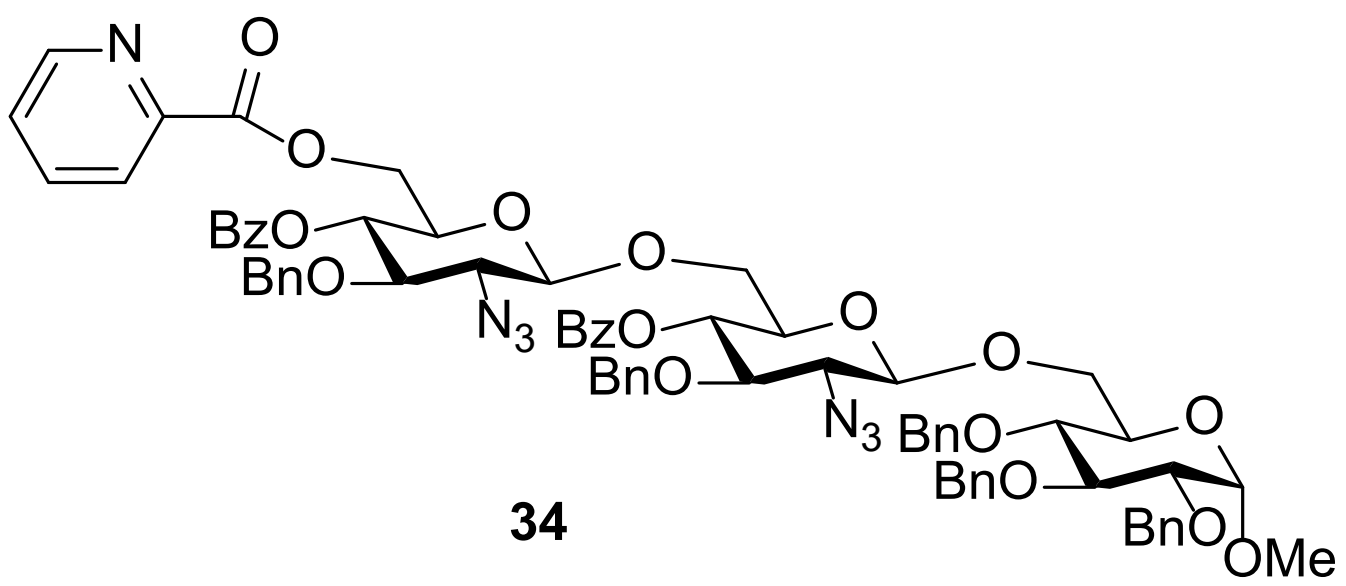

34

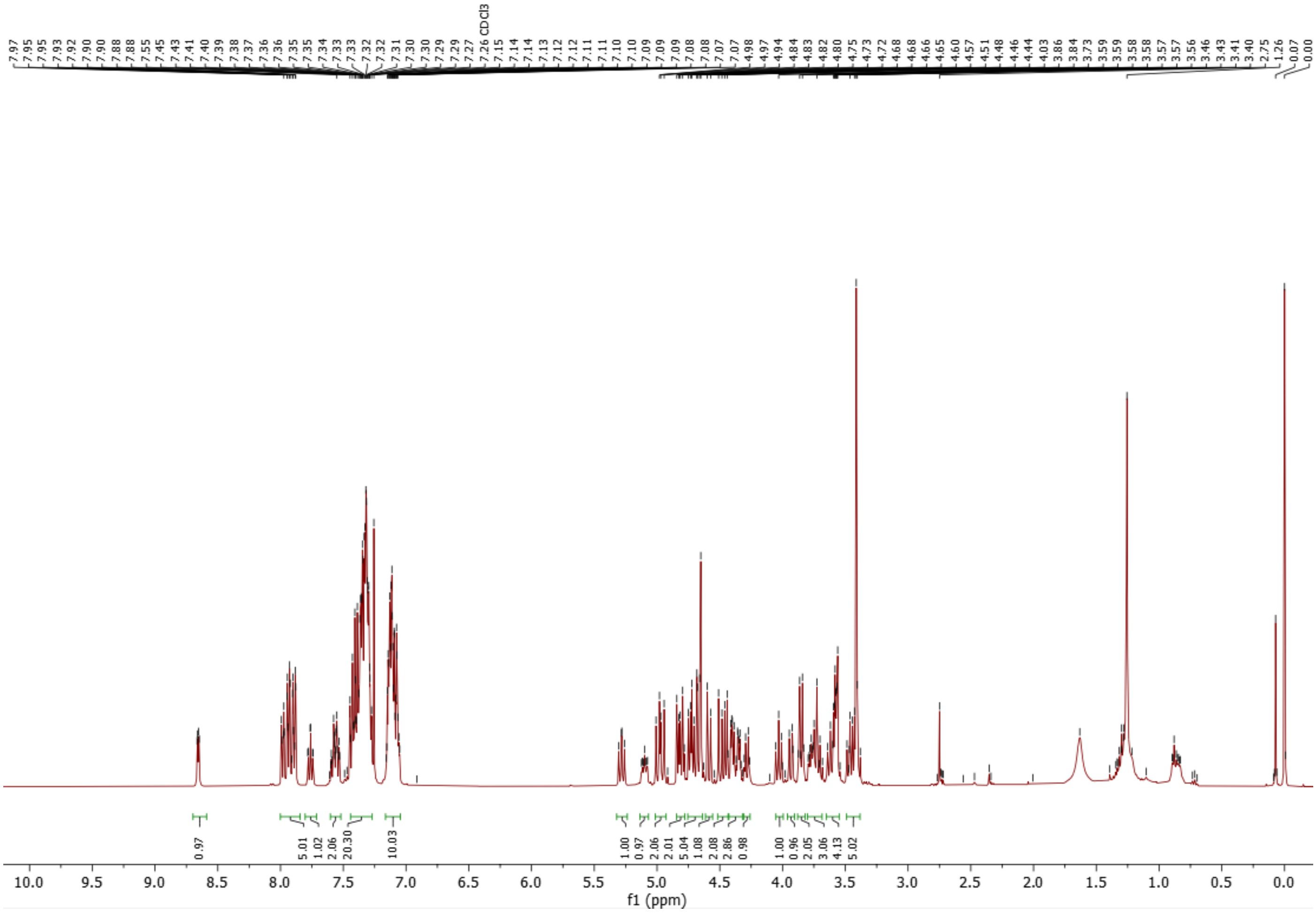

Figure S93: ^{1}H NMR (700 MHz, $CDCl_3$)

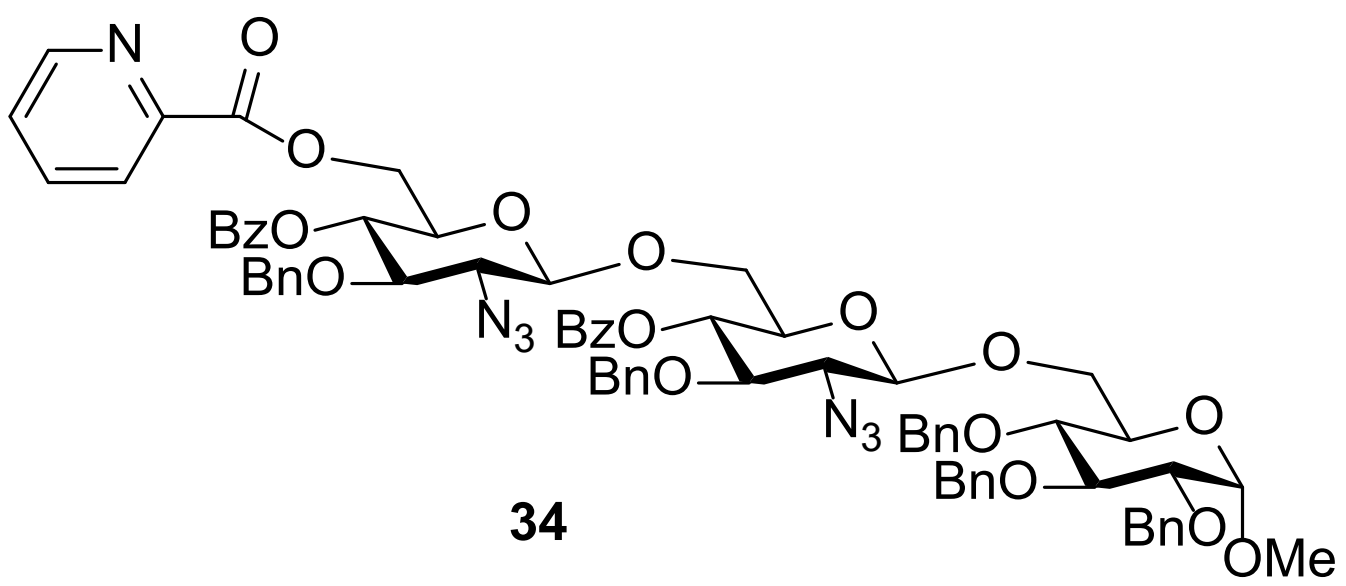

34

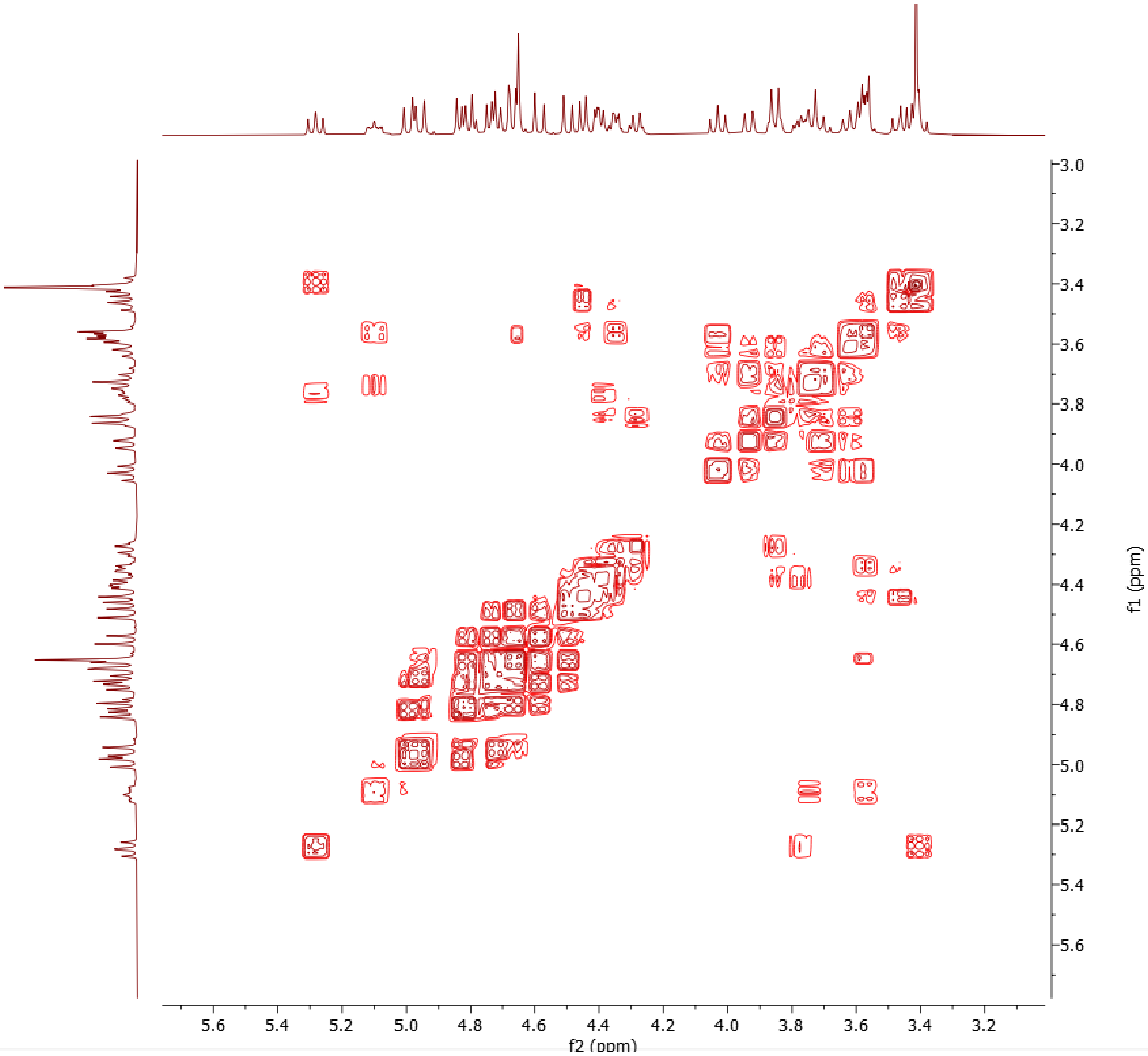

Figure S94: COSY NMR (700 MHz, $CDCl_3$)

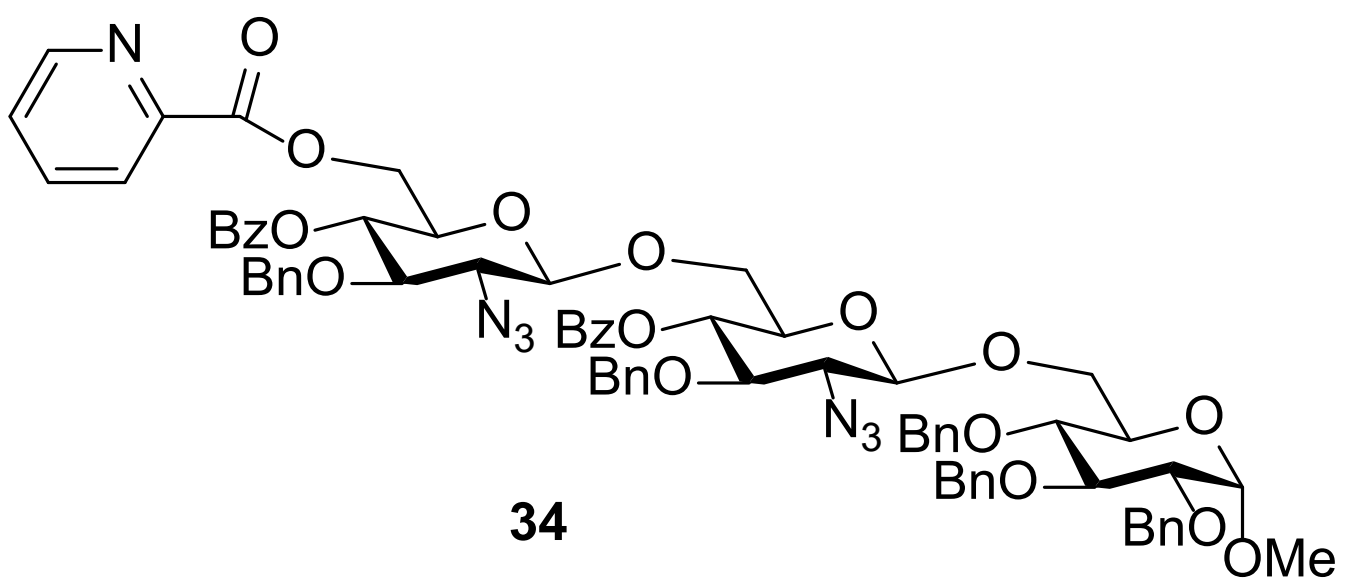

34

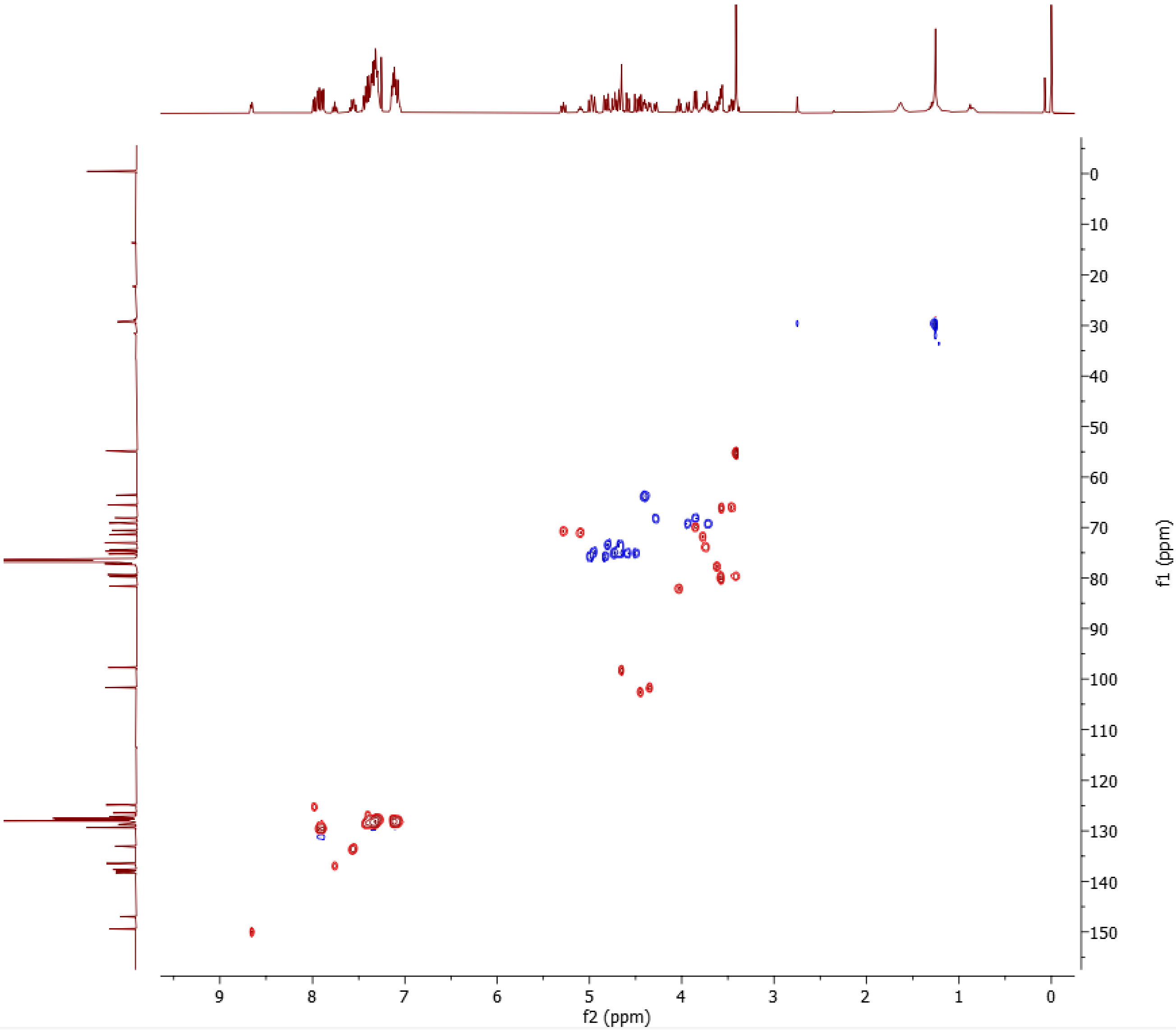

Figure S95: ^{1}H NMR and $^{13}C(^{1}H)$ NMR HSQC Spectrum (700 MHz, 176 MHz, $CDCl_3$)

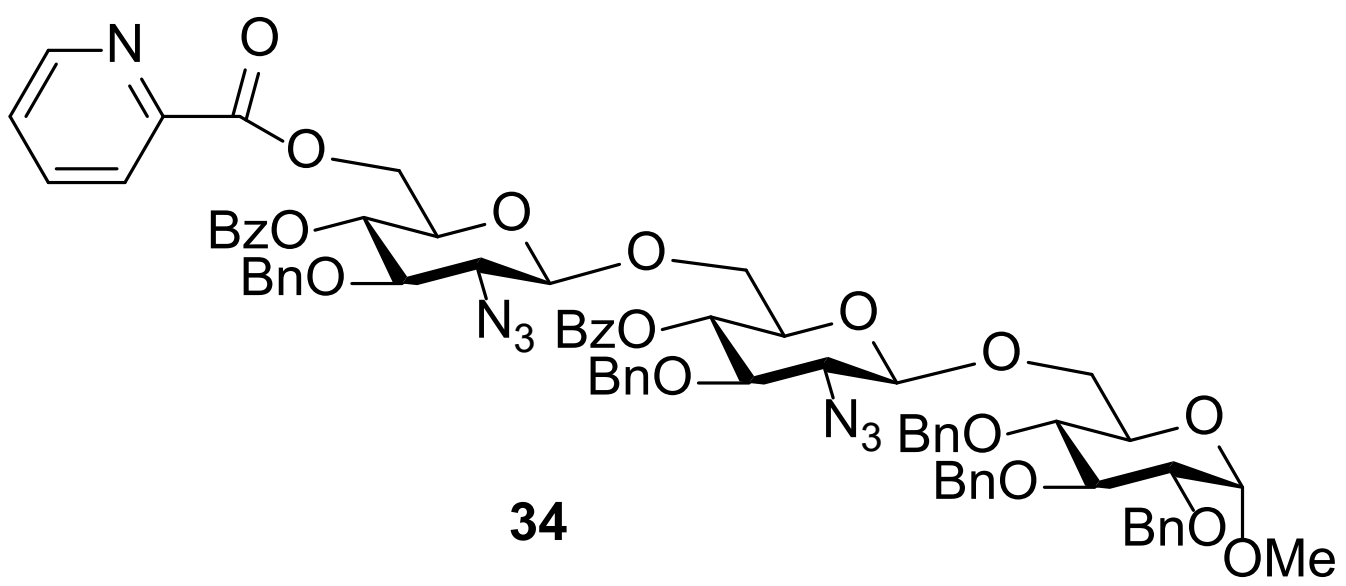

34

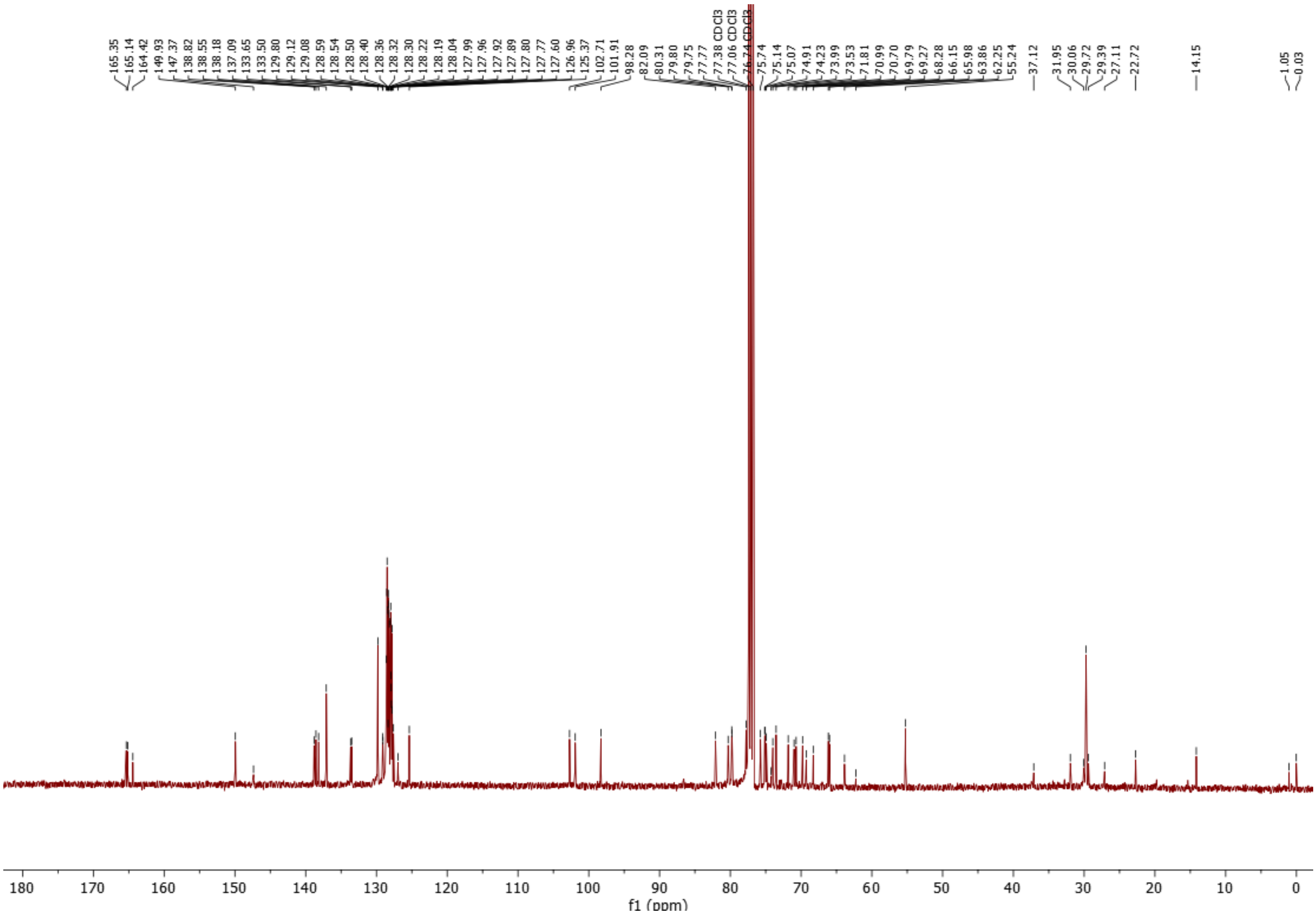

Figure S96: ^{13}C NMR (176 MHz, $CDCl_3$)

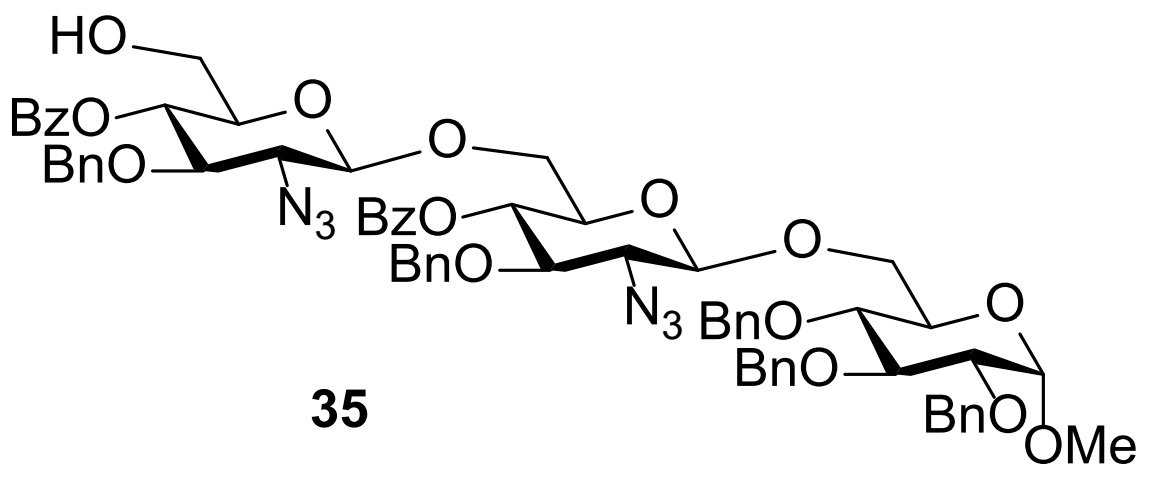

35

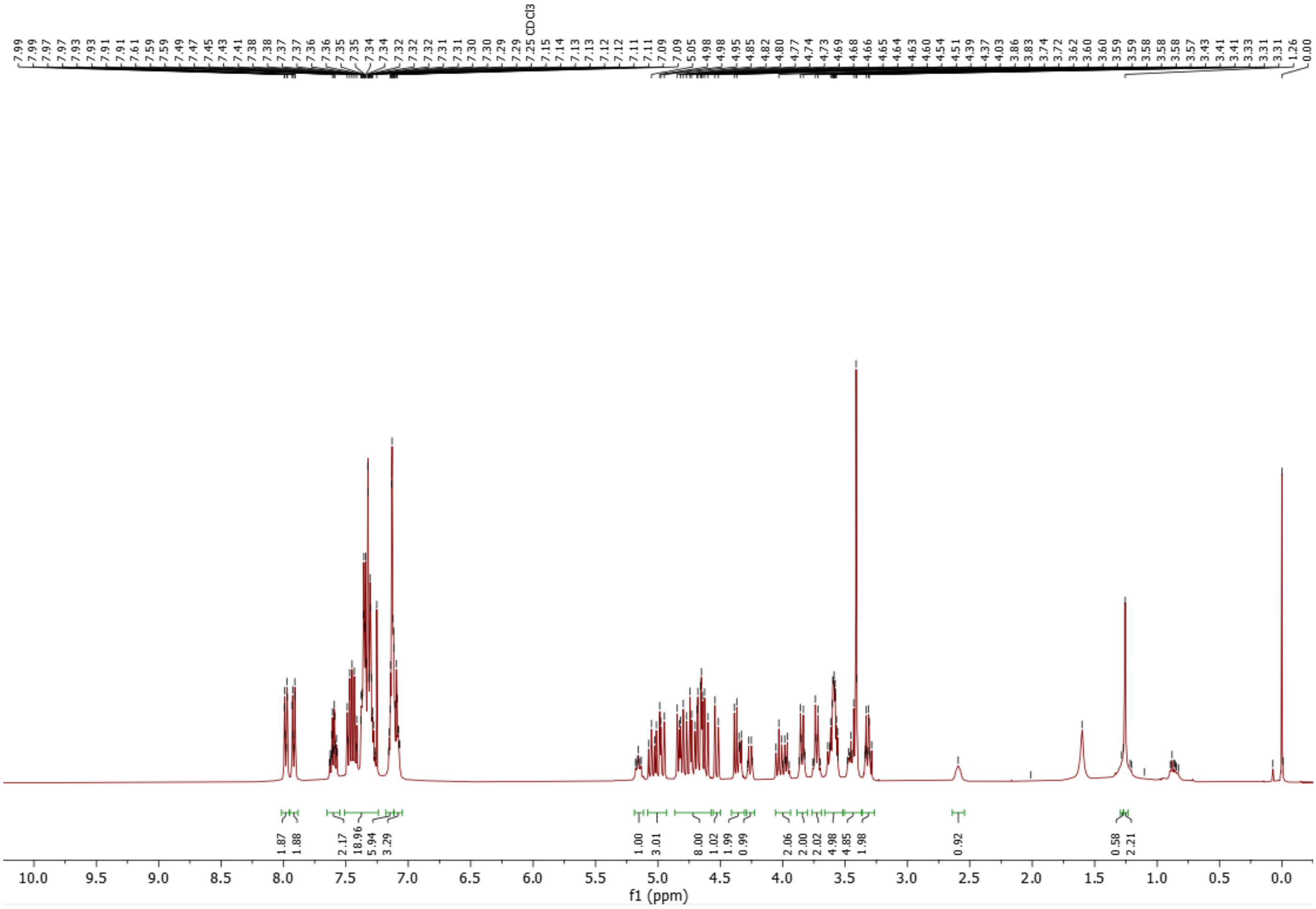

Figure S97: ^{1}H NMR (700 MHz, $CDCl_3$)

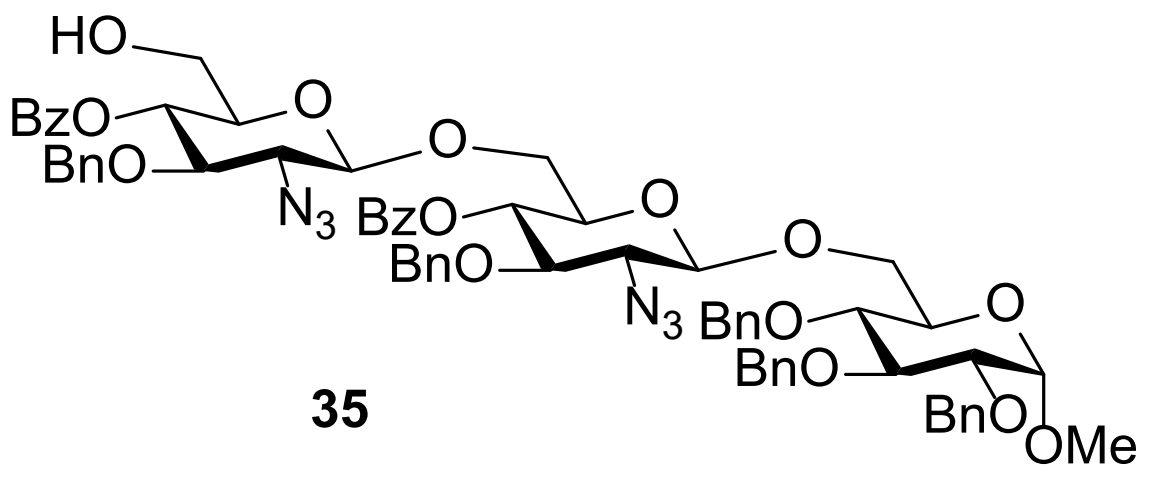

35

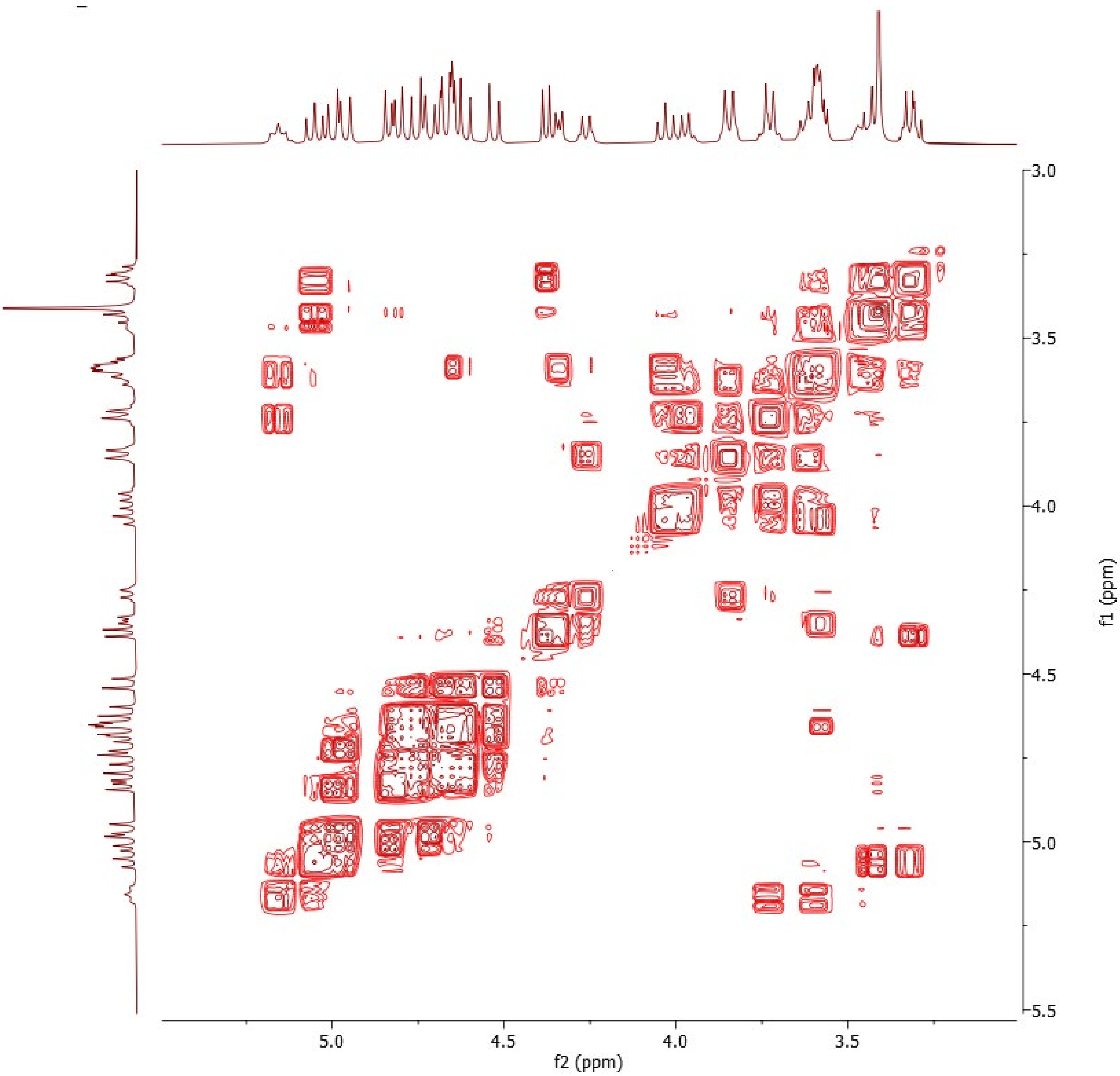

Figure S98: COSY NMR (700 MHz, $CDCl_3$)

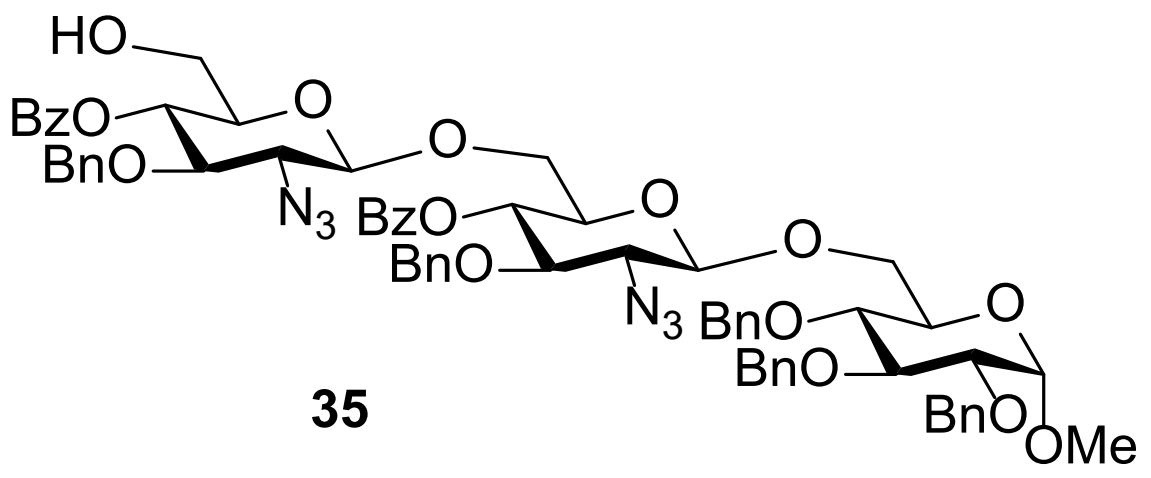

35

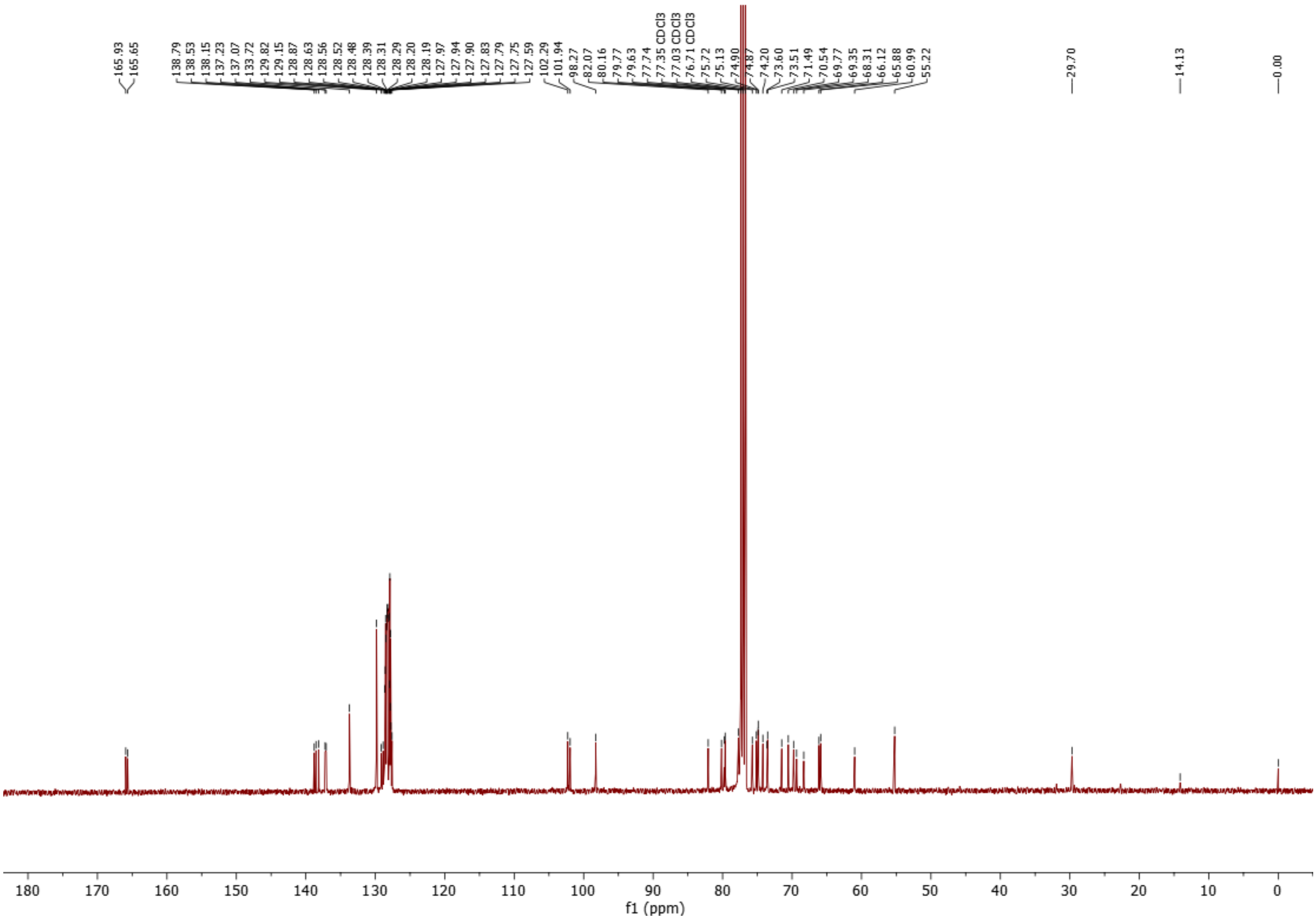

Figure S99: ^{13}C NMR (176 MHz, $CDCl_3$)

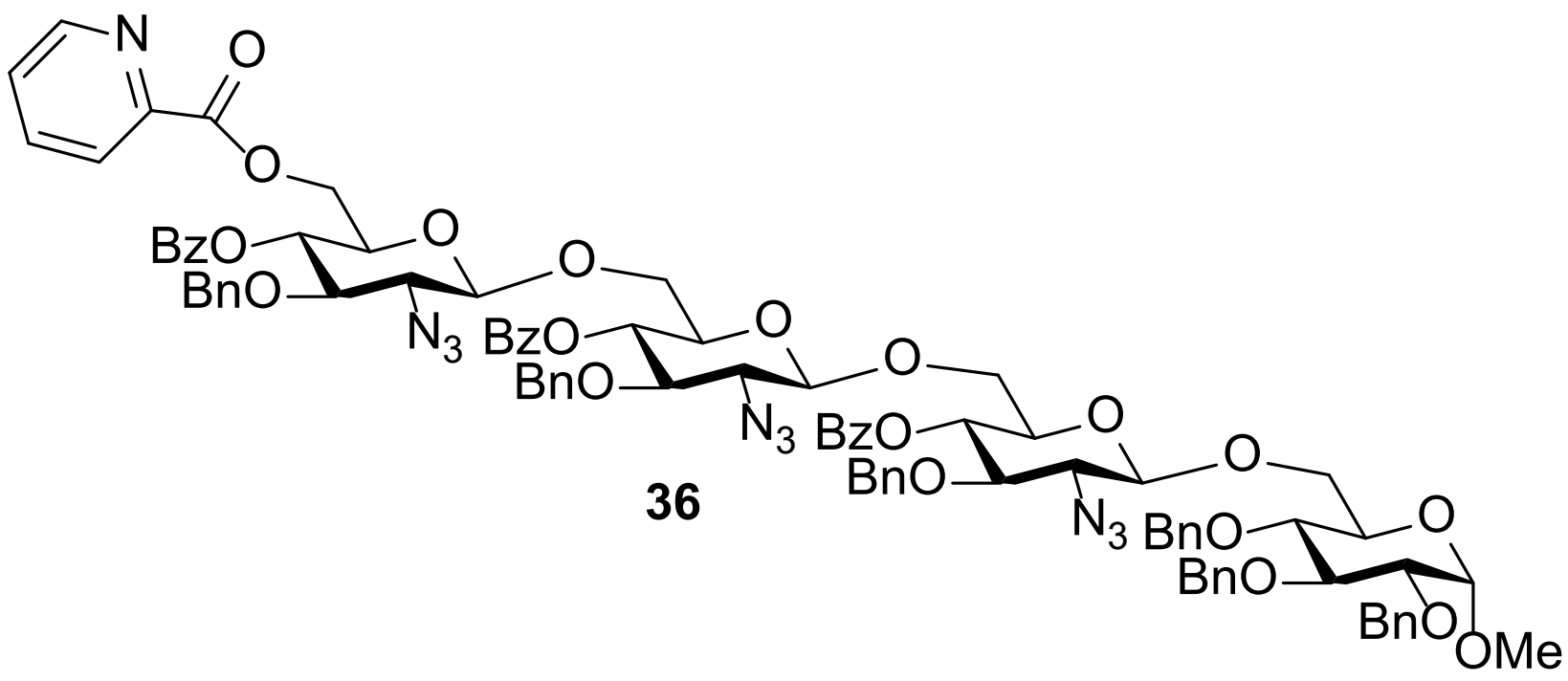

36

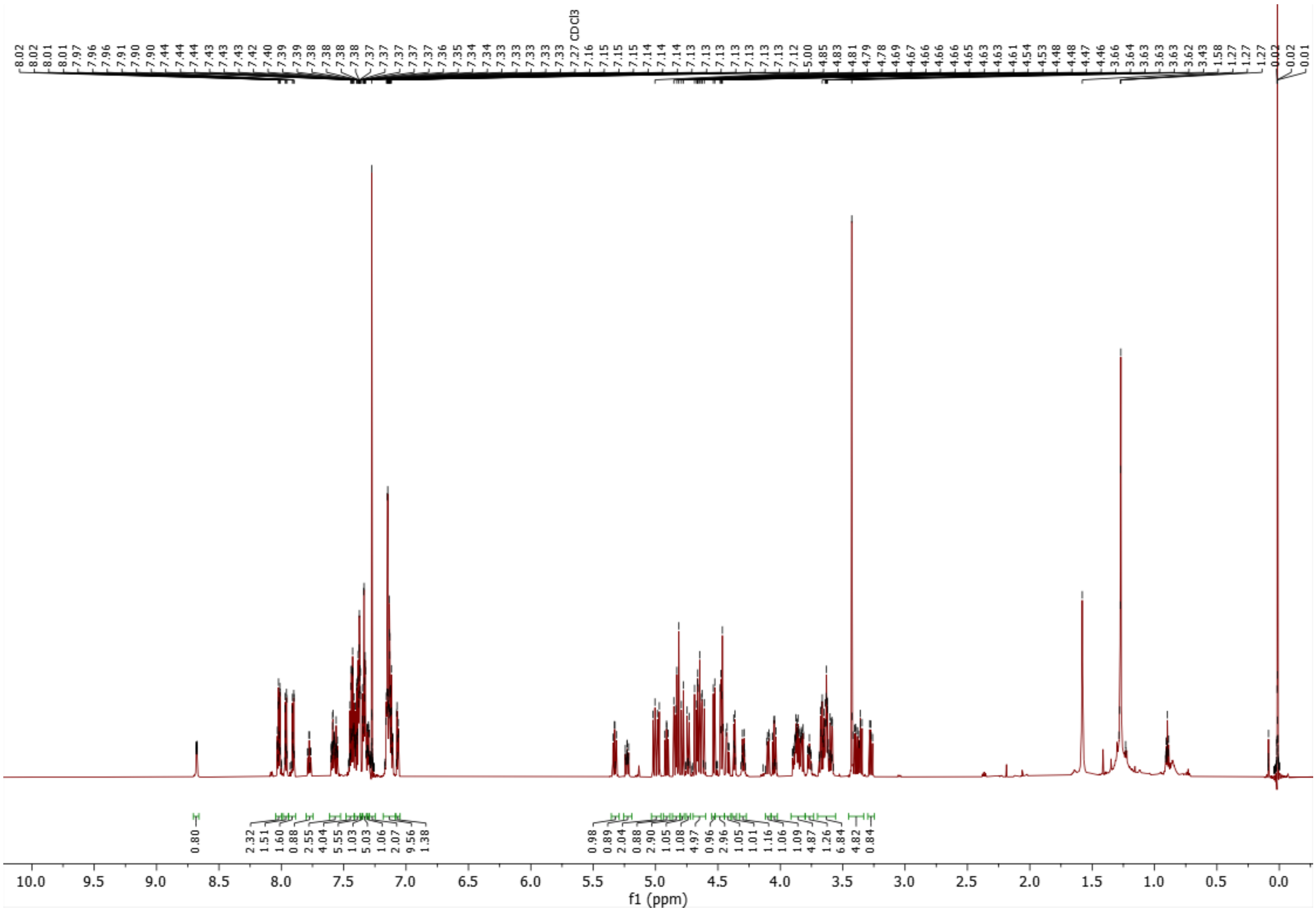

Figure S100: ^{1}H NMR (700 MHz, $CDCl_3$)

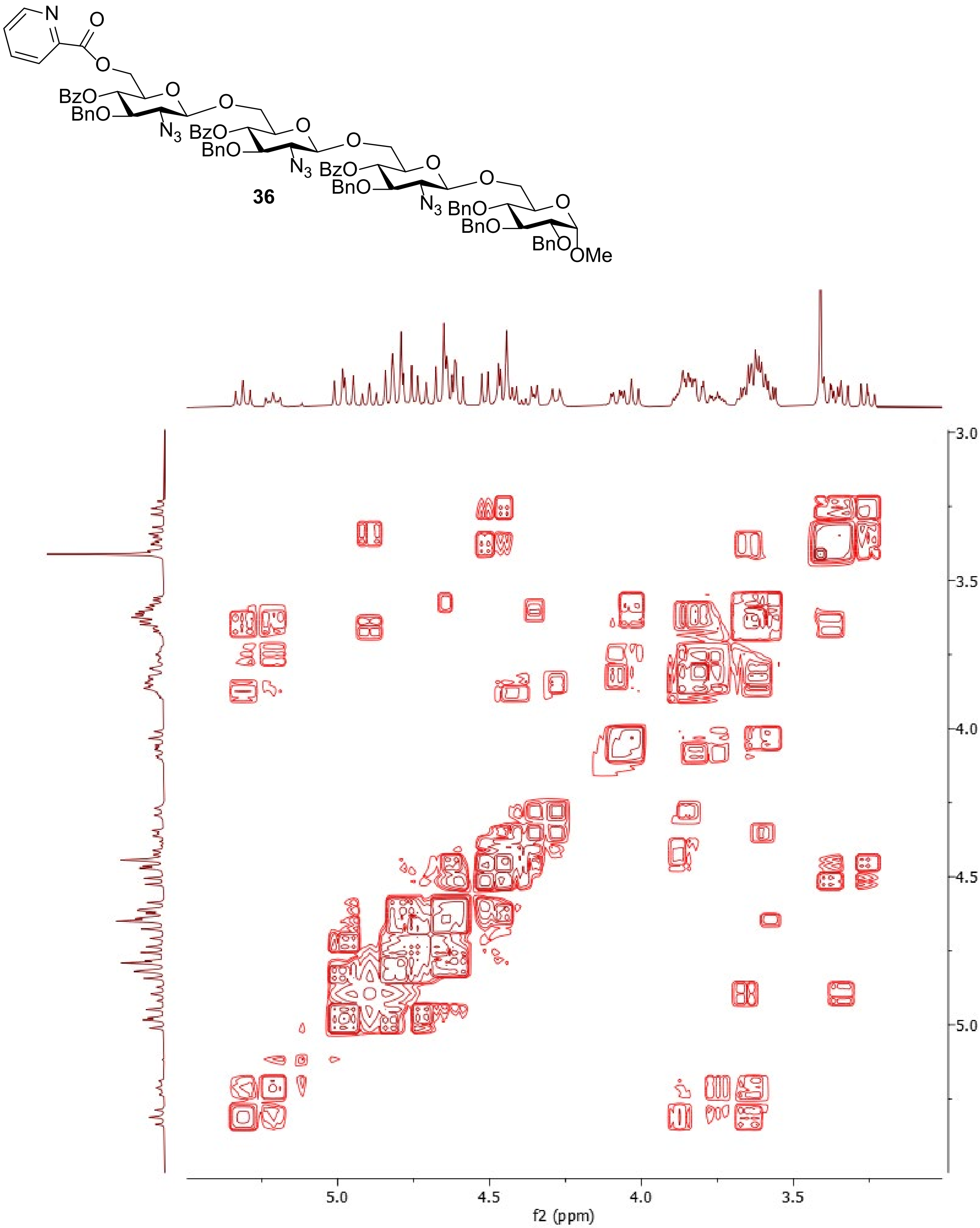

Figure S101: COSY NMR (700 MHz, $CDCl_3$)

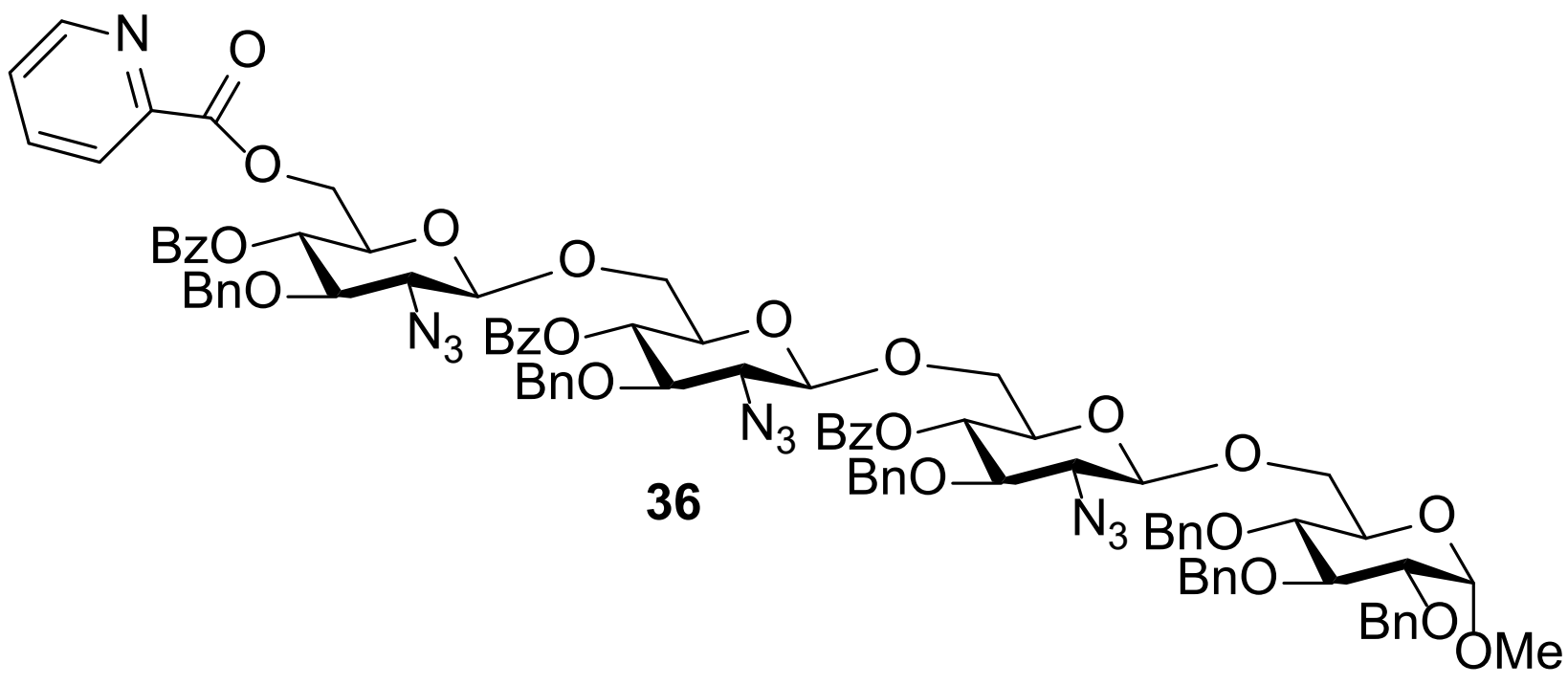

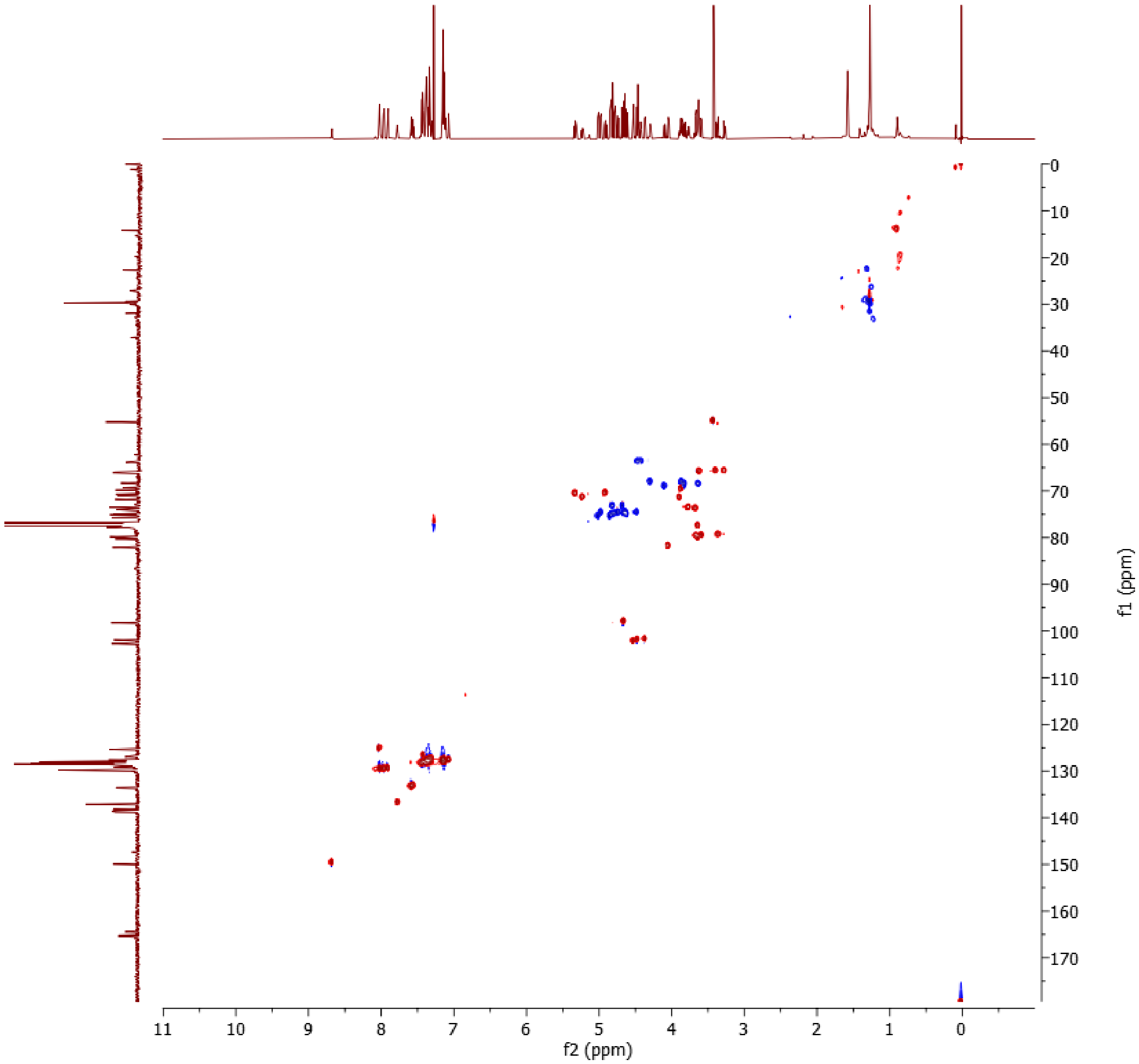

Figure S102: ^{1}H NMR and ^{13}C(^{1}H) NMR HSQC Spectrum (700 MHz, 176 MHz, $CDCl_3$)

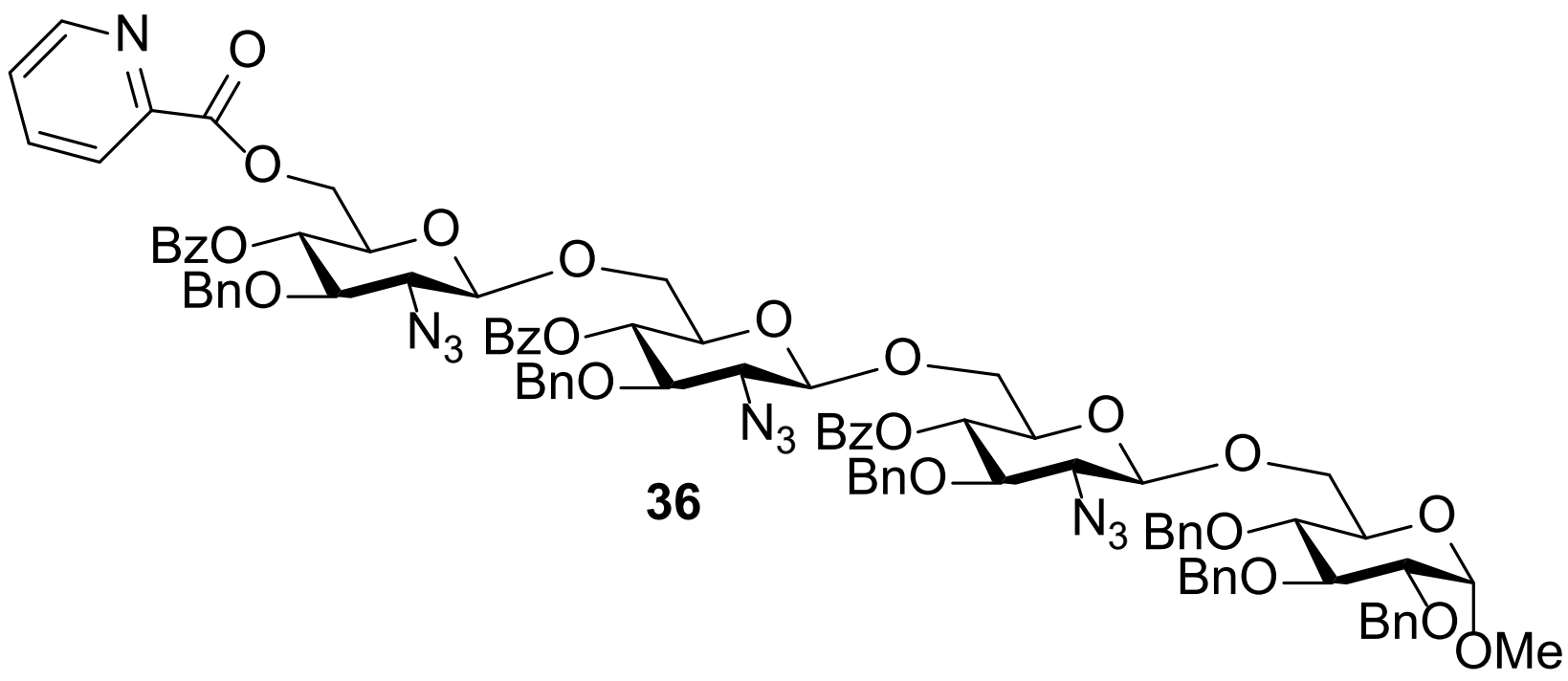

36

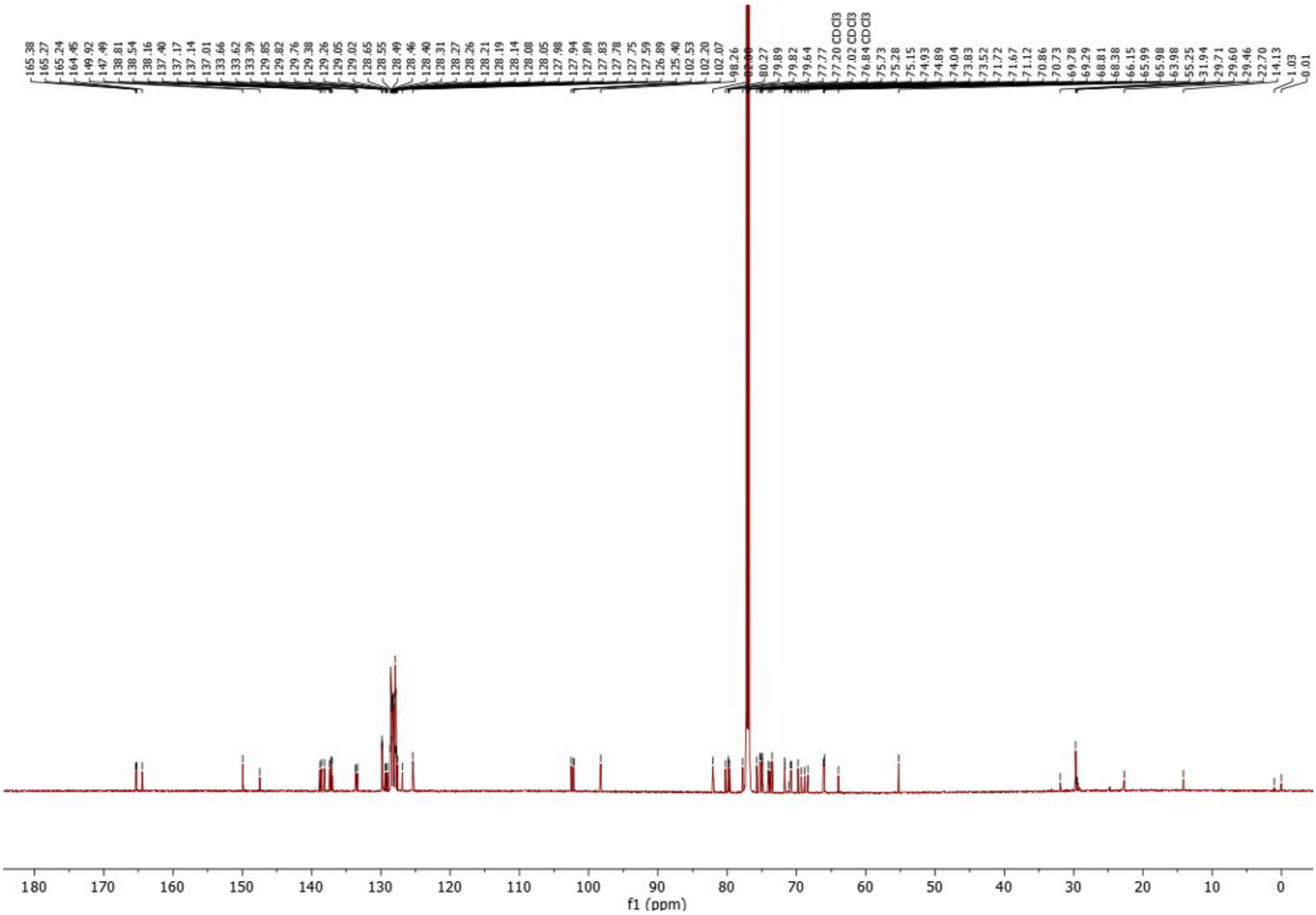

Figure S103: ^{13}C NMR (176 MHz, $CDCl_3$)
